# Supplementary material for: Temples and bats in a homogeneous agriculture landscape: Importance of microhabitat availability, disturbance and land use for bat conservation
Source: PLoS One. 2022 Jul 13;17(7):e0251771. doi: 10.1371/journal.pone.0251771 (PMC9278754; doi:10.1371/journal.pone.0251771)
Supplement: S1 Table — Model abbreviations-BLD: No.buildings, DR: Dark rooms, WAY: walkway, TWR: tower, REN: Renovation, VIS: visitors. (DOCX) [file pone.0251771.s001.docx]

| Temple model with disturbance | | | | | | | | | | |
| --- | --- | --- | --- | --- | --- | --- | --- | --- | --- | --- |
| Species richness | | | | | | | |  |  |  |
|  | Estimate | 2.500 | 97.500 | Std. Error | Models | df | logLik | AICc | delta | weight |
| (Intercept) | -0.052 | -0.291 | 0.187 | 0.122 | BLD+DR+REN | 7 | -171.900 | 358.270 | 0.000 | 0.600 |
| No. buildings | 0.674 | **0.587** | **0.761** | 0.044 | BLD+REN | 6 | -173.380 | 359.110 | 0.850 | 0.400 |
| Dark rooms | 0.246 | **0.044** | **0.448** | 0.103 |  | | | | | |
| Renovation | -0.364 | **-0.524** | **-0.204** | 0.081 |  |  |  |  |  |  |
|  |  |  |  |  |  |  |  |  |  |  |
| Bat Abundance | | | | | | | | | | |
| (Intercept) | 1.817 | -0.498 | 4.132 | 1.177 | BLD+DR+REN+TWR+WAY | 8 | -1118.670 | 2253.940 | 0.000 | 0.570 |
| No. buildings | 0.802 | **0.397** | **1.206** | 0.206 | BLD+DR+REN+VIS+WAY | 9 | -1117.880 | 2254.520 | 0.570 | 0.430 |
| Dark rooms | 1.597 | **0.541** | **2.654** | 0.536 |  | | | | | |
| walkway | 1.678 | -0.404 | 3.761 | 1.057 |  |  |  |  |  |  |
| Renovation | -0.319 | -1.016 | 0.378 | 0.354 |  |  |  |  |  |  |
| Visitors | -0.869 | **-1.473** | **-0.265** | 0.307 |  |  |  |  |  |  |
| Tower | 0.349 | -0.532 | 1.230 | 0.447 |  |  |  |  |  |  |
| *Hiposederous speoris* | | | | | | | |  |  |  |
| (Intercept) | -0.923 | -5.439 | 3.594 | 2.296 | BLD+DR+REN+VIS | 8 | -766.210 | 1549.020 | 0.000 | 0.400 |
| No. buildings | 0.769 | **0.090** | **1.448** | 0.345 | DR+VIS | 6 | -768.880 | 1550.120 | 1.090 | 0.230 |
| Dark rooms | 2.731 | **0.367** | **5.095** | 1.200 | BLD+DR+REN+TWR+VIS+WAY | 10 | -764.790 | 1550.510 | 1.480 | 0.190 |
| Renovation | -0.323 | -1.509 | 0.863 | 0.602 | BLD+DR+REN+TWR+VIS | 9 | -765.920 | 1550.600 | 1.580 | 0.180 |
| Visitors | -0.559 | -1.877 | 0.759 | 0.669 |  | | | | | |
| walkway | 3.631 | -0.948 | 8.209 | 2.324 |  |  |  |  |  |  |
| Tower | -0.748 | -2.566 | 1.069 | 0.922 |  |  |  |  |  |  |
| *Megaderma lyra* | | | | | | | |  |  |  |
| (Intercept) | -10.230 | -16.610 | -3.849 | 3.243 | BLD+REN | 6 | -176.560 | 365.460 | 0.000 | 0.660 |
| No. buildings | 0.982 | -0.162 | 2.126 | 0.581 | BLD+VIS+REN | 7 | -176.160 | 366.780 | 1.320 | 0.340 |
| Renovation | -3.462 | **-6.074** | **-0.850** | 1.326 |  | | | | | |
| Visitors | -1.769 | -6.113 | 2.575 | 2.205 |  |  |  |  |  |  |
| *Tadarida aegyptiaca* | | | | | | | | | | |
| (Intercept) | -6.086 | -12.775 | 0.603 | 3.413 | BLD+REN+VIS | 7 | -129.499 | 273.500 | 0.000 | 0.567 |
| Renovation | -3.723 | **-7.216** | **-0.230** | 1.782 |  | | | | | |
| visitors | -2.231 | -6.036 | 1.575 | 1.942 |  |  |  |  |  |  |
| No. buildings | 1.083 | **0.145** | **2.021** | 0.479 |  |  |  |  |  |  |
| *Taphozous melanopogon* | | | | |  |  |  |  |  |  |
| (Intercept) | -14.690 | -25.594 | -3.786 | 5.538 | BLD+REN+VIS | 7 | -265.480 | 545.420 | 0.000 | 0.680 |
| No. buildings | 0.758 | **0.132** | **1.384** | 0.318 | BLD+DR+REN+VIS | 8 | -265.140 | 546.890 | 1.460 | 0.320 |
| Renovation | -1.181 | -2.659 | 0.297 | 0.750 |  | | | | | |
| visitors | 2.312 | -1.594 | 6.217 | 1.983 |  |  |  |  |  |  |
| Dark rooms | 2.576 | -4.351 | 9.503 | 3.516 |  |  |  |  |  |  |
| *Rousettus leschenaultii* | | | | | | | | | | |
| (Intercept) | -9.636 | -16.172 | -3.100 | 3.318 | BLD+VIS | 6 | -190.460 | 393.270 | 0.000 | 0.440 |
| No. buildings | 1.163 | -1.296 | 3.622 | 1.248 | DR+REN | 6 | -191.100 | 394.550 | 1.290 | 0.230 |
| visitors | -0.913 | -3.988 | 2.161 | 1.561 | BLD+REN+VIS | 7 | -190.360 | 395.180 | 1.920 | 0.340 |
| Dark rooms | 0.687 | -4.871 | 6.245 | 2.822 |  | | | | | |
| Renovation | 0.667 | -2.645 | 3.979 | 1.681 |  |  |  |  |  |  |

**S1 Table2. Top models of species abundance response to microhabitat and disturbance. Model abbreviations-BLD: No.buildings, DR: Dark rooms, WAY: walkway, TWR: tower, REN: Renovation, VIS: visitors**
