# Supplementary material for: Temples and bats in a homogeneous agriculture landscape: Importance of microhabitat availability, disturbance and land use for bat conservation
Source: PLoS One. 2022 Jul 13;17(7):e0251771. doi: 10.1371/journal.pone.0251771 (PMC9278754; doi:10.1371/journal.pone.0251771)
Supplement: S2 Table — (DOCX) [file pone.0251771.s002.docx]

| species | Distance | Parameters | Estimate | 2.50% | 97.50% | SE | Model | df | logLik | AICc | delta | weight |
| --- | --- | --- | --- | --- | --- | --- | --- | --- | --- | --- | --- | --- |
| Species richness | 0 | Intercept | 0.829 | 0.503 | 1.155 | 0.166 | Water | 5 | -258.08 | 526.4 | 0 | 0.5 |
|  | 0 | Water | 0.029 | -0.144 | 0.202 | 0.088 | Trees | 5 | -258.09 | 526.42 | 0.01 | 0.5 |
|  | 0 | Trees | 0.006 | 0.002 | 0.010 | 0.002 |  |  |  |  |  |  |
|  | 500 | Intercept | 0.937 | 0.694 | 1.180 | 0.123 | (Null) | 4 | -256.61 | 521.39 | 0 | 0.86 |
|  | 500 | Grassland | -0.071 | -0.398 | 0.255 | 0.166 | Grass | 5 | -257.4 | 525.05 | 3.66 | 0.14 |
|  | 1000 | Intercept | 0.940 | 0.695 | 1.184 | 0.124 | (Null) | 4 | -256.61 | 521.39 | 0 | 0.72 |
|  | 1000 | Grassland | -0.197 | -0.982 | 0.588 | 0.399 | Grass | 5 | -256.5 | 523.24 | 1.85 | 0.28 |
|  | 3000 | Intercept | 0.793 | 0.541 | 1.071 | 0.128 | Grass | 5 | -253.56 | 517.4 | 0 | 0.817 |
|  | 3000 | Grassland | 0.653 | 0.190 | 1.120 | 0.235 |  |  |  |  |  |  |
|  | 5000 | Intercept | 0.861 | 0.548 | 1.173 | 0.159 | Grass | 5 | -255.49 | 521.22 | 0 | 0.52 |
|  | 5000 | Grassland | 0.618 | -0.205 | 1.441 | 0.418 | Null | 4 | -256.61 | 521.39 | 0.17 | 0.48 |
| Abundance | 0 | Intercept | 2.917 | 1.963 | 3.870 | 0.484 | Trees | 5 | -1132.44 | 2275.13 | 0 | 0.56 |
|  | 0 | Trees | 0.017 | 0.000 | 0.033 | 0.008 | Trees  +Water | 6 | -1132.29 | 2276.93 | 1.8 | 0.23 |
|  | 0 | Water | -0.174 | -0.804 | 0.456 | 0.320 | (Null) | 4 | -1134.48 | 2277.12 | 2 | 0.21 |
|  | 500 | Intercept | 3.307 | 2.587 | 4.027 | 0.366 |  |  |  |  |  |  |
|  | 500 | Trees | -0.032 | -0.097 | 0.032 | 0.033 | Trees | 5 | -1134 | 2278.25 | 0 | 0.34 |
|  | 500 | Grassland | 0.184 | -1.330 | 1.698 | 0.769 | Grass | 5 | -1134.44 | 2279.13 | 0.88 | 0.27 |
|  | 500 | Water | -0.001 | -0.061 | 0.059 | 0.030 | Water | 5 | -1134.48 | 2279.21 | 0.96 | 0.23 |
|  | 500 | Scrub | 0.142 | -0.088 | 0.371 | 0.116 | Grass  +Scrub | 6 | -1133.7 | 2279.75 | 1.5 | 0.16 |
|  | 1000 | Intercept | 3.229 | 1.964 | 4.495 | 0.643 |  |  |  |  |  |  |
|  | 1000 | Scrub | 0.176 | 0.057 | 0.294 | 0.060 | Trees  +Crop  +Urban  +Scrub | 8 | -1128.33 | 2273.26 | 0 | 0.58 |
|  | 1000 | Crop | 0.002 | -0.007 | 0.011 | 0.005 | Water+Trees+Grass+Scrub | 8 | -1128.65 | 2273.9 | 0.64 | 0.42 |
|  | 1000 | Trees | -0.144 | -0.237 | -0.051 | 0.047 |  |  |  |  |  |  |
|  | 1000 | Urban | 0.016 | -0.018 | 0.051 | 0.018 |  |  |  |  |  |  |
|  | 1000 | Water | -0.019 | -0.089 | 0.051 | 0.035 |  |  |  |  |  |  |
|  | 1000 | Grassland | -0.094 | -3.954 | 3.767 | 1.960 |  |  |  |  |  |  |
|  | 3000 | Intercept | 3.282 | 2.007 | 4.557 | 0.648 | Scrub | 5 | -1133.02 | 2276.29 | 0 | 0.23 |
|  | 3000 | Scrub | 0.066 | -0.004 | 0.136 | 0.036 | Trees | 5 | -1133.04 | 2276.33 | 0.04 | 0.23 |
|  | 3000 | Trees | -0.064 | -0.133 | 0.004 | 0.035 | Trees+Scrub+Grass | 7 | -1131.23 | 2276.93 | 0.64 | 0.17 |
|  | 3000 | Grassland | 0.291 | -1.387 | 1.968 | 0.851 | (Null) | 4 | -1134.48 | 2277.12 | 0.84 | 0.15 |
|  | 3000 | Water | -0.084 | -0.289 | 0.120 | 0.104 | Trees+Scrub+Water+Urban | 8 | -1130.52 | 2277.64 | 1.35 | 0.12 |
|  | 3000 | Urban | 0.035 | -0.059 | 0.129 | 0.048 | Trees+Scrub+Water+Grass | 8 | -1130.73 | 2278.06 | 1.77 | 0.1 |
|  | 5000 | Intercept | 7.065 | 3.306 | 10.824 | 1.913 |  |  |  |  |  |  |
|  | 5000 | Water | -0.498 | -0.864 | -0.132 | 0.186 |  |  |  |  |  |  |
|  | 5000 | Urban | -0.139 | -0.267 | -0.010 | 0.065 | Water+Trees+Crop+Urban | 8 | -1128.59 | 2273.78 | 0 | 0.35 |
|  | 5000 | Trees | -0.077 | -0.137 | -0.017 | 0.030 | Water+Trees+Grass+Urban | 8 | -1128.61 | 2273.82 | 0.04 | 0.34 |
|  | 5000 | Crop | 0.001 | -0.008 | 0.011 | 0.005 | Water+Trees+Grass+Scrub | 8 | -1128.73 | 2274.06 | 0.28 | 0.31 |
|  | 5000 | Grassland | -0.023 | -3.133 | 3.087 | 1.579 |  |  |  |  |  |  |
|  | 5000 | Scrub | 0.069 | 0.002 | 0.137 | 0.034 |  |  |  |  |  |  |
| Hipposideros | 0 | Intercept | 1.591 | 0.171 | 3.011 | 0.724 |  |  |  | 1554.2 |  |  |
|  | 0 | Trees | -0.004 | -0.032 | 0.025 | 0.015 |  |  |  |  |  |  |
|  | 500 | Intercept | 1.681 | -0.070 | 3.431 | 0.890 | Grass+Urban | 6 | -770.32 | 1552.98 | 0 | 0.26 |
|  | 500 | Grassland | 1.091 | -1.123 | 3.304 | 1.124 | Grass+Urban | 5 | -771.64 | 1553.53 | 0.55 | 0.22 |
|  | 500 | Urban | -0.036 | -0.080 | 0.008 | 0.022 | Grass+Scrub | 6 | -770.79 | 1553.93 | 0.94 | 0.19 |
|  | 500 | Scrub | 0.246 | -0.128 | 0.621 | 0.190 | Water | 5 | -772.07 | 1554.39 | 1.41 | 0.16 |
|  | 500 | Water | 0.017 | -0.085 | 0.119 | 0.052 | Trees | 5 | -772.12 | 1554.49 | 1.51 | 0.17 |
|  | 500 | Trees | -0.005 | -0.112 | 0.102 | 0.054 |  |  |  |  |  |  |
|  | 1000 | Intercept | 1.583 | -0.295 | 3.460 | 0.954 | Trees+Crop+Urban+Scrub | 8 | -767.06 | 1550.72 | 0 | 0.44 |
|  | 1000 | Scrub | 0.275 | 0.059 | 0.492 | 0.110 | Water+Trees+Grass+Scrub | 8 | -767.39 | 1551.38 | 0.66 | 0.31 |
|  | 1000 | Crop | 0.001 | -0.014 | 0.016 | 0.007 | Grass+Scrub | 6 | -769.75 | 1551.85 | 1.13 | 0.25 |
|  | 1000 | Trees | -0.173 | -0.333 | -0.013 | 0.081 |  |  |  |  |  |  |
|  | 1000 | Urban | -0.026 | -0.089 | 0.037 | 0.032 |  |  |  |  |  |  |
|  | 1000 | Water | 0.015 | -0.106 | 0.135 | 0.061 |  |  |  |  |  |  |
|  | 1000 | Grassland | 0.444 | -5.734 | 6.622 | 3.136 |  |  |  |  |  |  |
|  | 3000 | Intercept | 0.672 | -0.668 | 2.012 | 0.684 | Scrub | 5 | -769.857 | 1550 | 0 | 0.427 |
|  | 3000 | Scrub | 0.124 | 0.009 | 0.240 | 0.059 |  |  |  |  |  |  |
|  | 5000 | Intercept | 2.098 | -2.593 | 6.789 | 2.389 | Water | 5 | -772 | 1554.24 | 1.43 | 0.15 |
|  | 5000 | Grassland | -0.595 | -6.040 | 4.850 | 2.765 | Grass+Urban | 6 | -770.95 | 1554.25 | 1.45 | 0.15 |
|  | 5000 | Scrub | 0.112 | -0.007 | 0.231 | 0.060 | Grass | 5 | -772.05 | 1554.36 | 1.55 | 0.14 |
|  | 5000 | Water | -0.275 | -0.972 | 0.423 | 0.354 | Trees | 5 | -772.13 | 1554.5 | 1.69 | 0.13 |
|  | 5000 | Urban | -0.197 | -0.446 | 0.051 | 0.126 | Water+Grass+Urban | 7 | -770.16 | 1554.79 | 1.99 | 0.11 |
|  | 5000 | Trees | -0.002 | -0.101 | 0.097 | 0.050 |  |  |  |  |  |  |
| Megaderma | 0 | Intercept | -10.086 | -14.399 | -6.069 | 2.219 | Trees | 5 | -180.71 | 371.68 | 0 | 0.5 |
|  | 0 | Trees | 0.006 | -0.057 | 0.068 | 0.032 | Water | 5 | -180.73 | 371.71 | 0.03 | 0.5 |
|  | 0 | Water | -0.064 | -1.373 | 1.648 | 1.252 |  |  |  |  |  |  |
|  | 500 | Intercept | -9.165 | -12.658 | -5.673 | 1.773 |  |  |  |  |  |  |
|  | 500 | Grassland | -1.701 | -22.185 | 18.783 | 10.399 |  |  |  |  |  |  |
|  | 500 | Crop | -0.038 | -0.074 | -0.002 | 0.018 | Grass+Crop | 6 | -178.26 | 368.87 | 0 | 0.73 |
|  | 500 | Water | -0.053 | -0.387 | 0.282 | 0.170 | Water+Grass+Crop | 7 | -178.2 | 370.86 | 1.99 | 0.27 |
|  | 1000 | Intercept | -8.910 | -12.676 | -5.143 | 1.912 |  |  |  |  |  |  |
|  | 1000 | Grassland | -2.381 | -29.684 | 24.921 | 13.860 | Grass+Crop | 6 | -178.47 | 369.29 | 0 | 0.72 |
|  | 1000 | Crop | -0.031 | -0.060 | -0.001 | 0.015 | Water+Grass+Crop | 7 | -178.34 | 371.15 | 1.86 | 0.28 |
|  | 1000 | Water | -0.130 | -0.769 | 0.509 | 0.324 |  |  |  |  |  |  |
|  | 3000 | Intercept | -9.571 | -13.736 | -5.407 | 2.115 |  | 4 | -180.73 | 369.62 | 0 | 0.48 |
|  | 3000 | Grassland | -0.325 | -9.084 | 8.435 | 4.447 | Crop+Grass | 6 | -178.99 | 370.33 | 0.71 | 0.34 |
|  | 3000 | Crop | -0.028 | -0.059 | 0.003 | 0.016 | Water | 5 | -180.67 | 371.59 | 1.97 | 0.18 |
|  | 3000 | Water | -0.168 | -1.223 | 0.887 | 0.536 |  |  |  |  |  |  |
|  | 5000 | Intercept | -9.432 | -14.681 | -4.182 | 2.666 | Grass+Crop | 6 | -179.3 | 370.95 | 0 | 0.32 |
|  | 5000 | Grassland | -1.791 | -16.318 | 12.736 | 7.375 | Grass+Crop | 5 | -180.7 | 371.65 | 0.7 | 0.23 |
|  | 5000 | Crop | -0.027 | -0.061 | 0.006 | 0.017 | Water | 5 | -180.72 | 371.69 | 0.74 | 0.22 |
|  | 5000 | Water | -0.103 | -1.593 | 1.386 | 0.756 | Trees | 5 | -180.73 | 371.7 | 0.75 | 0.22 |
|  | 5000 | Trees | 0.008 | -0.220 | 0.236 | 0.116 |  |  |  |  |  |  |
|  | 0 | Intercept | -10.086 | -14.457 | -5.715 | 2.219 |  |  |  |  |  |  |
|  | 0 | Trees | 0.006 | -0.057 | 0.068 | 0.032 | Trees | 5 | -180.71 | 371.68 | 0 | 0.5 |
|  | 0 | Water | -0.064 | -2.531 | 2.403 | 1.252 | Water | 5 | -180.73 | 371.71 | 0.03 | 0.5 |
|  | 500 | Intercept | -9.441 | -13.064 | -5.819 | 1.839 | Trees | 5 | -190.96 | 392.17 | 0 | 0.38 |
|  | 500 | Trees | -0.354 | -1.823 | 1.115 | 0.746 | Grass | 5 | -191.14 | 392.52 | 0.34 | 0.32 |
|  | 500 | Grassland | -2.717 | -28.261 | 22.827 | 12.968 | Water | 5 | -191.16 | 392.57 | 0.39 | 0.31 |
|  | 500 | Water | 0.029 | -0.167 | 0.224 | 0.099 |  |  |  |  |  |  |
|  | 1000 | Intercept | -9.219 | -13.146 | -5.291 | 1.994 | Trees | 5 | -190.9 | 392.05 | 0 | 0.57 |
|  | 1000 | Trees | -0.381 | -1.805 | 1.043 | 0.723 | Water | 5 | -191.19 | 392.63 | 0.59 | 0.43 |
|  | 1000 | Water | 0.013 | -0.251 | 0.277 | 0.134 |  |  |  |  |  |  |
|  | 3000 | Intercept | -9.805 | -14.066 | -5.543 | 2.164 |  | 4 | -191.2 | 390.56 | 0 | 0.45 |
|  | 3000 | Trees | -0.159 | -0.928 | 0.610 | 0.391 | Trees | 5 | -191.05 | 392.35 | 1.79 | 0.19 |
|  | 3000 | Urban | 0.078 | -0.220 | 0.375 | 0.151 | Urban | 5 | -191.07 | 392.39 | 1.83 | 0.18 |
|  | 3000 | Grassland | 1.390 | -4.757 | 7.536 | 3.120 | Grass | 5 | -191.1 | 392.45 | 1.89 | 0.18 |
|  | 5000 | Intercept | -10.148 | -15.311 | -4.984 | 2.622 | Water | 5 | -191.12 | 392.48 | 0 | 0.27 |
|  | 5000 | Water | 0.241 | -0.913 | 1.395 | 0.586 | Grass | 5 | -191.12 | 392.49 | 0.01 | 0.27 |
|  | 5000 | Grassland | 2.023 | -8.862 | 12.908 | 5.526 | Trees | 5 | -191.14 | 392.53 | 0.05 | 0.26 |
|  | 5000 | Trees | -0.049 | -0.391 | 0.294 | 0.174 | Grass+Crop | 6 | -191.02 | 394.4 | 1.92 | 0.1 |
|  | 5000 | Crop | -0.010 | -0.054 | 0.035 | 0.023 | Grass+Scrub | 6 | -191.04 | 394.43 | 1.95 | 0.1 |
|  | 5000 | Scrub | 0.054 | -0.207 | 0.315 | 0.132 |  |  |  |  |  |  |
| Tadardia | 0 | Intercept | -9.608 | -14.070 | -5.146 | 2.266 | Waterer | 5 | -135.24 | 280.73 | 0 | 0.53 |
|  | 0 | Water | 0.555 | -1.653 | 2.763 | 1.121 | Treese | 5 | -135.36 | 280.97 | 0.24 | 0.47 |
|  | 0 | Trees | 0.001 | -0.057 | 0.059 | 0.030 |  |  |  |  |  |  |
|  | 500 | Intercept | -8.367 | -12.152 | -4.581 | 1.922 | Grass+Crop | 6 | -133.18 | 278.71 | 0 | 0.39 |
|  | 500 | Grassland | -1.819 | -19.858 | 16.221 | 9.158 | Trees+Crop+Urban | 7 | -133.01 | 280.49 | 1.78 | 0.16 |
|  | 500 | Crop | -0.023 | -0.046 | 0.000 | 0.012 | Grass+Water+Crop | 7 | -133.03 | 280.52 | 1.82 | 0.16 |
|  | 500 | Urban | -0.010 | -0.109 | 0.088 | 0.050 | Trees | 5 | -135.2 | 280.65 | 1.94 | 0.15 |
|  | 500 | Trees | -0.317 | -1.849 | 1.215 | 0.778 | Water | 5 | -135.22 | 280.69 | 1.98 | 0.14 |
|  | 500 | Water | -0.085 | -0.484 | 0.314 | 0.203 |  |  |  |  |  |  |
|  | 1000 | Intercept | -7.652 | -11.239 | -4.065 | 1.821 | Grass+Crop | 6 | -132.74 | 277.83 | 0 | 0.72 |
|  | 1000 | Grassland | -24.772 | -149.996 | 100.453 | 63.572 | Water+Grass+Crop | 7 | -132.64 | 279.75 | 1.92 | 0.28 |
|  | 1000 | Crop | -0.022 | -0.043 | -0.001 | 0.010 |  |  |  |  |  |  |
|  | 1000 | Water | -0.081 | -0.506 | 0.344 | 0.216 |  |  |  |  |  |  |
|  | 3000 | Intercept | -8.235 | -13.475 | -2.995 | 2.663 | Crop+Grass | 6 | -132.38 | 277.1 | 0 | 0.43 |
|  | 3000 | Grassland | 0.812 | -5.570 | 7.193 | 3.240 | Water+Crop+Grass | 7 | -131.94 | 278.35 | 1.24 | 0.23 |
|  | 3000 | Crop | -0.022 | -0.040 | -0.004 | 0.009 | (Null) | 4 | -135.36 | 278.89 | 1.79 | 0.18 |
|  | 3000 | Water | -0.487 | -1.728 | 0.755 | 0.630 | Trees+Urban+Crop | 7 | -132.3 | 279.07 | 1.96 | 0.16 |
|  | 3000 | Urban | 0.061 | -0.228 | 0.350 | 0.147 |  |  |  |  |  |  |
|  | 3000 | Trees | -0.031 | -0.344 | 0.283 | 0.159 |  |  |  |  |  |  |
|  | 5000 | Intercept | -8.291 | -14.453 | -2.129 | 3.130 | Grass+Crop | 6 | -132.35 | 277.05 | 0 | 0.56 |
|  | 5000 | Grassland | -0.488 | -12.427 | 11.452 | 6.061 | Water+Grass+Crop | 7 | -132.2 | 278.87 | 1.83 | 0.22 |
|  | 5000 | Crop | -0.024 | -0.044 | -0.004 | 0.010 | Trees+Crop+Urban | 7 | -132.22 | 278.9 | 1.85 | 0.22 |
|  | 5000 | Water | -0.368 | -1.794 | 1.058 | 0.724 |  |  |  |  |  |  |
|  | 5000 | Urban | 0.117 | -0.338 | 0.572 | 0.231 |  |  |  |  |  |  |
|  | 5000 | Trees | -0.022 | -0.264 | 0.220 | 0.123 |  |  |  |  |  |  |
| Taphazhus | 0 | Intercept | -8.476 | -12.411 | -4.542 | 1.998 | Water | 5 | -269.63 | 549.5 | 0 | 0.45 |
|  | 0 | Water | -0.997 | -3.967 | 1.973 | 1.508 | Trees | 5 | -269.83 | 549.91 | 0.41 | 0.37 |
|  | 0 | Trees | 0.010 | -0.051 | 0.071 | 0.031 | Waterer+Treese | 6 | -269.48 | 551.3 | 1.8 | 0.18 |
|  | 500 | Intercept | -9.273 | -12.846 | -5.699 | 1.815 | Trees | 5 | -269.77 | 549.8 | 0 | 0.26 |
|  | 500 | Trees | -0.096 | -0.837 | 0.645 | 0.376 | Water | 5 | -269.82 | 549.88 | 0.09 | 0.25 |
|  | 500 | Water | 0.029 | -0.176 | 0.233 | 0.104 | Grass | 5 | -269.84 | 549.92 | 0.12 | 0.25 |
|  | 500 | Grassland | 0.322 | -3.117 | 3.760 | 1.746 | Grass+Urban | 6 | -269.32 | 550.99 | 1.2 | 0.14 |
|  | 500 | Urban | 0.047 | -0.052 | 0.147 | 0.051 | Grass+Crop | 6 | -269.72 | 551.78 | 1.98 | 0.1 |
|  | 500 | Crop | -0.007 | -0.036 | 0.021 | 0.015 |  |  |  |  |  |  |
|  | 1000 | Intercept | -9.137 | -12.374 | -5.901 | 1.644 |  |  |  |  |  |  |
|  | 1000 | Grassland | 1.637 | -5.136 | 8.410 | 3.438 | Grass | 5 | -269.75 | 549.75 | 0 | 0.25 |
|  | 1000 | Water | 0.056 | -0.195 | 0.308 | 0.128 | Water | 5 | -269.76 | 549.77 | 0.01 | 0.25 |
|  | 1000 | Trees | -0.032 | -0.425 | 0.362 | 0.200 | Trees | 5 | -269.84 | 549.93 | 0.17 | 0.23 |
|  | 1000 | Crop | -0.012 | -0.030 | 0.006 | 0.009 | Grass+Crop | 6 | -269.02 | 550.39 | 0.63 | 0.18 |
|  | 1000 | Urban | 0.029 | -0.100 | 0.157 | 0.065 | Grass+Urban | 6 | -269.65 | 551.66 | 1.9 | 0.1 |
|  | 3000 | Intercept | -8.635 | -13.813 | -3.456 | 2.633 | (Null) | 4 | -269.86 | 547.87 | 0 | 0.31 |
|  | 3000 | Grassland | 7.892 | -1.886 | 17.670 | 4.964 | Urban+Grass | 6 | -268.34 | 549.04 | 1.16 | 0.17 |
|  | 3000 | Urban | -0.208 | -0.693 | 0.276 | 0.246 | Water | 5 | -269.42 | 549.09 | 1.22 | 0.17 |
|  | 3000 | Water | 0.302 | -0.337 | 0.940 | 0.324 | Urban | 5 | -269.74 | 549.73 | 1.85 | 0.12 |
|  | 3000 | Scrub | 0.050 | -0.162 | 0.261 | 0.107 | Scrub | 5 | -269.76 | 549.76 | 1.89 | 0.12 |
|  | 3000 | Trees | -0.059 | -0.502 | 0.384 | 0.225 | Trees | 5 | -269.8 | 549.85 | 1.97 | 0.11 |
|  | 5000 | Intercept | -9.437 | -15.329 | -3.546 | 2.994 |  |  |  |  |  |  |
|  | 5000 | Grassland | 5.918 | -7.637 | 19.473 | 6.883 | Grass | 5 | -269.47 | 549.18 | 0 | 0.25 |
|  | 5000 | Trees | -0.091 | -0.514 | 0.332 | 0.215 | Trees | 5 | -269.7 | 549.66 | 0.48 | 0.19 |
|  | 5000 | Water | 0.198 | -0.856 | 1.252 | 0.535 | Water | 5 | -269.79 | 549.83 | 0.65 | 0.18 |
|  | 5000 | Crop | -0.009 | -0.025 | 0.006 | 0.008 | Grass+Crop | 6 | -268.99 | 550.32 | 1.14 | 0.14 |
|  | 5000 | Urban | -0.262 | -0.930 | 0.405 | 0.339 | Grass+Urban | 6 | -269 | 550.35 | 1.17 | 0.14 |
|  | 5000 | Scrub | 0.081 | -0.171 | 0.333 | 0.128 | Grass+Scrub | 6 | -269.27 | 550.89 | 1.71 | 0.1 |

**S2 Table3. Top models of species response to land-use elements**.
