# Supplementary material for: Temples and bats in a homogeneous agriculture landscape: Importance of microhabitat availability, disturbance and land use for bat conservation
Source: PLoS One. 2022 Jul 13;17(7):e0251771. doi: 10.1371/journal.pone.0251771 (PMC9278754; doi:10.1371/journal.pone.0251771)
Supplement: Attachment — Submitted filename: PONE-D-21-13889_R1_ReviewerEdits.pdf [file pone.0251771.s008.pdf]

PLOS ONE

Manuscript Click here to access/download;Manuscript;Bats in ancient temples of South India.docx

--Manuscript Draft--

**Manuscript Number:**	PONE-D-21-13889R1
**Article Type:**	Research Article
**Full Title:**	Manuscript Click here to access/download;Manuscript;Bats in ancient temples of South India.docx
**Short Title:**	Temples and Bat conservation
**Corresponding Author:**	Ganesh T<br>Ashoka Trust for Research in Ecology and the Environment<br>Bangalore, INDIA
**Keywords:**	bats; microhabitat; Hindu temples; India; landscape,
**Abstract:**	Anthropogenic structures such as temples in south Asia are widely used by cave-dwelling bats as roosting and nursery sites. These roosts are constantly under threat as temples are being renovated and bats removed from such places even more so after the COVID19 pandemic. No detailed understanding exists on what makes temples favorable for bats and what factors are important for their persistence. Here we relate temple microhabitat characteristics and land use around ancient temples (>400 years) to bat species richness and abundance in the Tamiraparani river basin of south India. Temples were selected for sampling along the basin based on logistics and permission to access them. Bats were counted at the roost in the mornings and late afternoons from inside the temples. Temple characteristics such as dark rooms, corridors, crevices and presence of tower were recorded after the count along with disturbances to the roost. We also recorded land use such as crop, trees, scrub, grassland, urban areas and water availability within 5 km radius of the temple based on ESA landuse classifications. We used GLMM to relate the counts in temples with microhabitat and land use. We sampled 59 temples repeatedly across 5 years which yielded a sample of 256 survey events. The total number of bats counted was 20,211 of which Hipposideros speoris was the most common (9,715) followed by Rousettus leschenaulti (5,306), Taphozous melanopogon (3,196), Megaderma lyra (1,497) Tadarida aegyptiaca (303), Pipistrellus sp (144) and Rhinopoma hardwickii (50). About 39% of the abundance was accounted by dark rooms while 51% by walkways around the deity room. Species richness and total abundance were related to the number of building in the temple and the availability of dark rooms. Land use elements only had a weak effect but scrub and grassland even though were few are important for bats. We conclude that retaining undisturbed dark rooms with small exits in temples and other dimly lit areas and having natural areas around temples are critical for bats conservation in the area.
**Order of Authors:**	Ganesh T
	Saravanan Amaavasai
	Mathivanan M
**Response to Reviewers:**	All responses to reviewers are marked as authors response(AR) in red.<br><br>Editor<br><br>The reviewers agree and the AE concurs that there is a degree of opacity in the analytical approaches that require elucidation before the manuscript can be fully vetted (at least with respect to the analyses, their results, and the inferences drawn from them). The reviewers identify a number of areas in the analytical methods that require additional detail and clarification.<br>AR: We have re written the Material and methods section giving clarity in the methods used and the data used in the analysis<br>the manuscript requires substantial editing to correct considerable errors in grammar

Powered by Editorial Manager® and ProduXion Manager® from Aries Systems Corporation

and syntax.
AR: This has been addressed to the extent possible
Reviewer 1.

Review: Temples the last abode for bats in a homogeneous agriculture matrix: Importance of
microhabitat availability and land use factors for bat conservation.

Ganesh et al studies historic temples as roosting habitat for bats in India. This work highlights the importance of anthropogenic structures as essential roosts, particularly as temples are places where humans are actively working to remove bats. While focusing on one region in India, the authors conducted roost counts and identified species of bats over several years while also assessing each temple for several microhabitat and landscape factors. They conducted linear models to compare species richness and abundance to the landscape and microhabitat variables and calculated niche breadth and overlap. The authors found seven species of bat across forty seven out of fifty-four temples that they surveyed. Dark rooms and corridors were found to be the most important microhabitats for roosting, while landscape effects had little overall effect albeit with some species-specific instances. Bat abundance varied by species in season, but overall was lowest during the monsoon season. There was little species overlap in temples and little niche overlap. The authors attributed a lot of the microhabitat preferences to species-specific life history and roosting preferences. The seasonality differences correlated with the parturition and lactation of many of the species. While the landscape effect had a weak response, the importance of scrub and open landscape is still noted. Overall, this paper lays the foundation for further studies and emphasizes the importance of an anthropogenic roost for bats. This paper certainly merits publication with additional clarification and editing.
My first concern overall for the paper is some grammatical and formatting issues. I have listed specific examples of reoccurring problems throughout at the end of this review, however there are additional grammar edits that need to be fixed.
My second concern is with the landscape ecology analysis and discussion throughout the paper. The analysis of the landscape features was measuring presence/absence of landscape features over a significantly sized area. This broad measuring does not get at some of the heterogeneity of the landscape and may explain some of the lack of effect of the landscape features in the model. A more rigorous measurement of the landscape types could be better such as using the total amount or percentage of land cover type in the 5km buffer. As the remote sensing data may be unable to provide this level of detail (as mentioned in the methods), a mention of this drawback in the discussion would be warranted. Additionally, the discussion section touched on the landscape feature briefly but the middle of the paragraph still discussed temple characteristics such as festivals and renovations and the ending sentences focused on site fidelity. The importance of open spaces was stated but never explained or elaborated on. A deeper discussion with a paragraph focused solely on landscape characteristics while moving the still-relevant topics to more appropriate places would improve the analysis especially since the third hypothesis on landscape features was disproven.
Overall, this paper has significant importance for publishing by emphasizing the importance of temples as habitat worthy of conservation especially at a time when bats are at a high risk of exclusion from places because of the pandemic. Some modifications are necessary to clarify the paper and its findings, however they do not detract from the importance of the study.

Authors response(AR):
The landscape analysis has been completely revised. We have used recently released ESA landcover classes at 10m resolution for the analysis (https://tiledimageservices.arcgis.com/P3ePLMYs2RVChkJx/arcgis/rest/services/Esri_2020_Land_Cover_V2/ImageServer). However, this approach allows us to estimate the landcover class for a particular period and not across seasons because of cloud cover as mentioned in the MS earlier. We have therefore assumed, based on government literature that landcover has not drastically changed during the study period which is also substaintiated by authors observations. We analysis the % landcover under each class to species richness and abundance of bats at 3 spatial scales;500m, 1km and 3km. The variable in the landcover class is crop which we determine based on google earth images as earlier and interviews with farmers around temples. Some periods of

the sampling May and Sep do not have any crops and there fore we have considered crop area in such times as zero and considered it as fallow agriculture. More details in the MS.
Reviewers: A deeper discussion with a paragraph focused solely on landscape characteristics while moving the still-relevant topics to more appropriate places would improve the analysis especially since the third hypothesis on landscape features was disproven.
AR: Our discussion has changed and improved given the changes in the analysis. We have as suggested given more space for discussing landscape characterstics.

Grammatical and Formatting Comments:
Check the spacing throughout the article. There should be a space in between the last word and opening parentheses. On the last sentence of page 11, there should be a space after the comma and between each year. The same issues occur in the caption for Table 4 with the list of symbols. Lastly, there should be a space in between genus and species in the species names in the results - I.e. M. lyra, not M.lyra.
In the first two paragraphs of the introduction, there are a few words and several citations where the year is in blue. They should all be in black since they are not hyperlinks.
A w is bolded in the word “with” in the beginning of the full paragraph on page 10.
Throughout the entire paper, halls is followed by (corridors) or vice versa each time. I think saying “halls and corridors” is appropriate the first time and then use one consistently for the rest of the paper.
AR: We agree on the English and have tried our best to improve. Some of the issues have also arised due to formatting.
We have modified halls to a more appropriate word ‘walkway’
Figure 1 appears blurry, so I would recommend the resolution to be larger. Partially because of the resolution, the temples and town dots are hard to tell apart. It would be easier to read if one of the dots was hollow.
AR: We have redone the figure
Figure 3 does not have axis labels which helps the reader.
Corrected

In Figure 4, the dark room and hall patterns are very hard to tell apart between the bars. A different pattern would make the graph easier to read.
We have redone the figure

Figure 5 has extra ticks around the outside of the graph.
We have redone the figure

In Figure 8, the no-village bar is distracting and confusing as it overlaps with the error bars.
Changed the fig as we are using landcover maps

Reviewer 2

Reviewer Summary of Paper: This is a study of factors important for bats using ancient temples and overlap of roost use in bats using ancient temples. The authors investigate roost characteristics of temples (microhabitat) and surrounding land use (landscape-scale habitat at 5 km scale). They determine that roost characteristics (mainly presence of dark rooms and halls) influence bat richness/abundance in temples. Landscape variables at the 5 km scale were not important for bat abundance and bat presence.
Overall impression: The subject of managing historic/ancient buildings and bat conservation will appeal to many readers. The study would add to the literature of managing ancient/historic buildings for bats. However, the manuscript would need major revision before publication in PLoS One. The discussion is the strongest section of this paper, and other sections could be improved by incorporating more literature into the introduction to set up the hypotheses, clarifying the hypotheses, organizing the data analysis section by hypothesis, adding more information about which data were used in which analysis, and clarifying some of the figures.
My specific comments to the authors to improve the manuscript are outlined below:
Title:

	1.The term "matrix" implies a heterogenous landscape of multiple land use/cover types not a homogenous one (only one cover type). I suggest rewording or clarifying the title.<br>Author response AR: replaced "matrix" with "landscape"<br>Abstract:<br>2.Please specify which pandemic.<br>3.AR: Mentioned as COVID19 pandemic<br><br>4."We sampled 59 temples repeatedly across 5 years which yielded a sample of 256 temple." I believe that 59 temples sampled repeatedly is a sample size of 59 temples. I suggest saying "which yielded 256 survey events" or rephrasing another way. Was every temple sampled 5 times?<br><br>AR:Yes sample size is 59 temples and these were repeatedly sampled. Suitably modified in the abstract. The number of times each temple was sampled varied between 1 to 5. Have mentioned the methods.<br>Introduction:<br>The introduction should be supported with more literature to justify the research question. I suggest the authors incorporate the literature on bats using anthropogenic structures and introduce and explain the issues here for bats, humans managing structures, and bat-human conflicts. A good paper to read is Fagan et al. 2018. "Roost selection by bats in buildings, Great Smoky Mountains National Park" in the Journal of Wildlife Management 82: 424-434, as they evaluated bat roost selection of historic buildings in a non-urbanized area and also measured landscape variables. If the literature is limited for bats in buildings, the authors could draw on the literature of bats using bridges or other anthropogenic transportation structures that humans have to manage. One example is Meierhofer et al. 2019. "Structural and environmental predictors of presence and abundance of tri-colored bats in Texas culverts" in the Journal of Mammalogy 100: 1274-1281. One of the more interesting aspects of this subject is that bats using historical buildings or other human infrastructure makes it difficult to balance bat conservation with human-bat conflicts. And this could be applied beyond ancient temples.<br>More information on the types of bat species that use these temples, why we should care about them, and current conservation status would greatly improve this manuscript. For example, the authors could mention the importance of bats to agricultural pest suppression (if these bats are insectivores) or seed dispersal (if these bats are frugivores) or providing other examples of ecosystem services. The authors should explain what they mean by the pandemic negatively affecting bats. Future readers may not know which pandemic the authors are referring to, and why it affected bats. If any species are "data deficient" or of conservation concern on the IUCN Red List of Threatened Species (https://www.iucnredlist.org/), this should also be mentioned in this introduction. Additionally, if there have been previous studies involving these species, i.e., what they usually use to roost if not temples, what they eat, where they forage, etc. this would be helpful to include some basic background information to set up the hypotheses. Are they mostly fruit bats or are they insectivores? Some species background information is provided in the discussion but is really needed in the introduction to support the justification of these research questions.<br>Similarly, more information on temple structure, e.g., What materials are they made out of? How do bats get into the temples (through doors or cracks in the walls or unknown?) Are they air conditioned or are they about the same temperature/hotter/cooler than outside?) Additionally, introduce readers to the monsoon seasons briefly in the introduction and more in the study area section.<br>AR: More information on temple structure given in the MS itself<br><br>The authors should be careful about using the term "niche" when only referring to roosting habitat. A niche encompasses all needs of a species (roosting habitat, foraging habitat, all biotic and abiotic factors (climate), etc.). In some parts of this manuscript, I believe "niche" should be replaced with "roost habitat".<br>The objectives of this paper need to be clarified if they are investigating what microhabitat and landscape factors determine roost use and bat abundance in order to inform bat conservation in ancient temples, and/or if they are investigating which bats have roost overlap needs (what I think the authors are referring to when they say "niche overlap and breadth"). What is important about quantifying niche overlap/breadth?

Powered by Editorial Manager® and ProduXion Manager® from Aries Systems Corporation

	AR: we have removed niche analysis from the results.<br>5.“These are situated in a human-dominated agricultural matrix that is changing due to several economic reasons and climate change effects (Fischer et al. 2009, 2010; Jones et al. 2009).” Please describe the climate change effects affecting bats in this area. Do you mean the monsoons? I also suggest including literature on anthropogenic disturbance, agriculture, and habitat/fragmentation threats to support your justification for this research. I suggest developing those ideas more with Frick et al. 2019 “A review of the major threats and challenges to global bat conservation” paper (available at: https://www.researchgate.net/publication/332146982_A_review_of_the_major_threats_and_challenges_to_global_bat_conservation) or other studies<br>AR: The role of climate and habitat transformation in the region has been stated in the context of Frick et al 2019 paper. We have also added some available literature for the region.<br><br>6.“The recent pandemic has further made them more vulnerable to disturbance and direct persecution.” Please describe which pandemic and why this is an issue for bats.<br>AR: COVID 19, Done<br><br>7.“ 1. Given that the temple bats are mostly cave-dwelling species we expect larger Yinpterochiroptera occupying similar micro-habitat, show greater niche overlap and similar niche breadth.” Please introduce the species before stating the hypotheses. This is the first time the bats and some traits are mentioned. Please describe niche breadth and overlap in this context. Does this mean the authors are trying to determine overlap in roost needs/preferences of species using the temples and that they expect Yinpterochiroptera to have overlapping needs with each other? Why does this matter? Please clarify this hypothesis. Are Yinopterochiroptera the only group that use the temples? Are they all cave-dwelling?<br><br>AR: we have modified this hypothesis. We have removed niche overlap from the paper. Mention of species is done earlier and most of the species are insectivorous expect for one<br><br>8.“2. Microclimate conditions inside the temples are very stable and any influence on bat abundances could be a reflection of food availability or disturbance at roost and therefore we expect bats to show seasonal changes in niche overlap and breadth due to variability in crop and insect availability.”<br><br>This hypothesis is confusing to me. I’m not sure what you are hypothesizing. Would it not be better to test abundance changes seasonally rather than niche overlap and breadth? The climate/monsoon seasons could also be described before introducing this hypothesis. Additionally, it is unclear how disturbance at roost and food availability were measured.<br><br>AR: we have modified this hypothesis and have made a separate result for roost disturbance which is mentioned in the introduction and methods.<br>9.“3. Since the structure of ancient temples are all similar, we do not expect any change in microhabitat availability in temples with and without bats, instead we expect bat abundances driven by the availability of landscape features especially seasonal crops around the temples.” Please describe how the temples differ or are similar to each other and the seasonality of crops before introducing this hypothesis. Also, is it not true that the temples are structurally different? Some are smaller/larger and more complex than others?<br>AR:We have described the temples in detail and the type of crop and their availability in the section. Temple did vary in size but structurally they were more or less similar.<br><br>Materials and methods:<br><br>“The ancient temples(>400 years) are built of large granite stones and provide a niche for several bat species.” I think the authors mean that the temple structures provide roost habitat, not a full niche.<br><br>AR: Agreed and we have clarified in the text

Powered by Editorial Manager® and ProduXion Manager® from Aries Systems Corporation

	“The surveyed temples are situated in an agricultural matrix surrounded by rice paddies or bananas” Are the temples completely surrounding in the whole 5 km radius landscape or just mostly surrounded in the immediate area around the temple? What is the extent of the surrounding agriculture? An agricultural matrix implies mixed cover types so this sentence is somewhat confusing.<br>AR: The entire area even beyond 5km are dominated by paddy. We have given more information about the landscape in the introduction and study area section<br>“stone pillars that supported the stone roof was considered a separate habitat as they provided a niche for few species that roost in the gaps created between the stone joints this was categorized as crevices” This sentence is a little confusing as it reads. Were the stone joints in the pillars categorized as crevices? The stone pillars provided a roost, not a niche.<br>AR: Yes, they provide crevices and therefore a roost habitat<br>“In 2012 we measured temperature and humidity while sampling for bats and found no difference between temples and therefore not considered in the analysis.” What are the temperature and humidity conditions in these temples and is it similar to cave conditions used by these bats?<br>AR: We are not aware of temperature and humidity conditions inside caves in the region as caves are non-existent because the rocky hills have been quarried. Conditions inside the temples are mentioned though, as stated in the methods section, we could not monitor these continuously<br>“Since we did not have information on how far the different bat species forage in the landscape we took 5 km based on the study on the large bat Megaderma lyra (Audet et al 1991).” Did the authors consider testing multiple spatial scales? Many bat studies have determined that landscape scale factors are important at different scales. See the discussion in Bellamy et al. 2013 “Multiscale, presence-only habitat suitability models: fine resolution maps for eight bat species” in the Journal of Applied Ecology 50: 892-901 (available at: https://besjournals.onlinelibrary.wiley.com/doi/full/10.1111/1365-2664.12117) about importance of factors at multiple landscape scales. In the case of not knowing foraging distance for all species, it would improve this study to quantify landscape availability at more than one scale to determine if it is important (For example, maybe test 500 m, 1 km, and 5 km or landscape scales that are relevant to bats in other parts of the world). What kind of bat is the Megaderma lyra? Is it an insectivorous species?<br>AR: Thank you for the paper link. We have used one-time remote sensing classification of the land use available at ESA website and reanalyzed the data at 4 different spatial scales as suggested. Since such information was available only at one particular year for the entire region we considered the land use not to have changed during the sampling period and when the image was taken. This we justify based on government records of area under agriculture. We also tried to do a fine level classification by gridding the area around temples and finding the land use within the grid cells using google earth. This was a herculean task as the grid cells for each temple at 3km was over 1000 and was not possible to do that for each temple across years and is the reason we resorted to remote sensing analysis for fine scale analysis<br>.<br>AR: Megaderma lyra is an insectivorous species and we have mentioned the details of all the species in a separate Table 1..<br><br>“If a land use element is present we gave a value of 0.25 in each quadrant and calculated the frequency of each land use for the entire 5km radius area.” Is 25% (i.e, 0.25) sensitive enough to detect variation? Why 25% and not 10%? If a 5 km radius landscape only has a little bit of banana, but it is present, then it would still get a value comprising 25% of the landscape? This may be a limitation of the study to mention in your discussion. Would the results be different if the 5 km landscape was divided by 10% increments?<br>AR: This is a very valid point and we agree on the bias and as mentioned above we have done our best under the current conditions. Keeping all the comments related to landuse we have changed the analysis as mentioned before.<br>Did land use around each temple differ or was it correlated among the 5 sampling years?<br>AR: Yes, the land use did not change much across years<br>“The best model was selected using.” Please finish this sentence and indicate what AICc weights and delta AIC value criteria were used to determine plausible models.<br>AR: done

“We constructed a linear model to explain the species richness and abundance of bats in the temples based on the microhabitat and landscape features. We based the variables used in our linear models on our observations made in 2012.” Does this mean that only the landscape features from 2012 and the bat counts from 2012 were used? I think it would be statistically incorrect if all survey years at the same temples were treated as independent temples in the models.

AR:We have use mixed model approach to repeated sampling of temples across years following Bolker et al. 2009 wherein they say “Repeated measurements on the same individual, the same location, or observations taken at the same point in time are often correlated; this correlation can be accounted for by using random effects in generalized linear mixed models”. Since there were variations both within the sampled temples and across years, making temple and years as random factors accounts for this variation and gives a more robust relationship between the response variable (species richness and abundance of bats) with explanatory variables like temple features and land use elements.

Please organize the data analysis section to be more clear which analysis you are doing to answer which question/hypothesis and clarify which data were used to examine microhabitat and landscape factor influence on abundance and richness. In other words, did you use only data from 2012 or did you treat each repeated survey as a separate “temple”? If only used 2012 data, what were the monsoon conditions at that time?

AR: Temples were sampled for its features in 2012 and if any new temples were added in later years their features was recorded then. Temple features are subjected to modifications and therefore we have included renovation of temples and visitors to the temple as variables that could affect bats. Renovation is a dynamic variable that can differ between years and therefore we have measured it in all sampled years in all temples compared to more static ones like number of buildings, tower etc which do not change across years.
We have now tried to bring in more clarity in the data analysis section as suggested

Results:

“The total number of roosts sampled was 351 across 246 temples in 5 years.” This sentence is a little confusing. There were only 59 temples. Were any of the roosts that were repeatedly sampled the same roosts as previous years?

AR: We have modified the sentence and yes, same roosts were sampled across years and some temples also had multiple roosts

“None of these bat species is threatened as per IUCN category.” Are any of them considered “data deficient”? Please move this species information to the introduction to introduce the species that use the temples.
AR: Done. No bat was data deficient

“The lmer model explained 67% of the variation in species richness of which 55% were explained by the fixed factors.” Do you mean the model averaged mixed model comprising all models with AICc <5 or the top model or something else?
AR: The % values pertain to the top model. The models have changed after reanalysis. The delta AICc was kept at <2 unless mentioned specifically. We have corrected for any discrepancies

Discussion:
The authors are effective at relating the results to the previous literature and this is the strongest section of the paper.
Mention the limitations of the study (e.g., temples were chosen based on logistics rather than randomly, only one landscape scale was considered, land use in the surrounding 5 km radius was considered at a course resolution (0.25))
AR: The landscape section has been reanalyzed completely as mentioned earlier
From your results, are their certain species that may be more impacted by temple renovation/disturbance?

Powered by Editorial Manager® and ProduXion Manager® from Aries Systems Corporation

	AR: We have now made renovation as an objective as mentioned earlier and identify species that are affected, most species are.<br><br>Figures:<br>Fig 1 map is a bit blurry. I suggest increasing dpi/resolution. Did there appear to be any spatial pattern of temples that did not have bats vs. those that did? If so, mention in discussion.<br>AR: The fig has been redone. We did not test for spatial correlations<br><br>Fig 4 bar graph gray colors are difficult to differentiate between dark room and hall.<br>AR: Corrected<br>Fig 5 is this a bar graph of all temples, including the ones that were resampled? If so, perhaps plot the average species richness across the 5 years against the no. of buildings in each temple. It is difficult to interpret as is.<br><br>AR: These are not bar graphs, they are effect plots from the mixed model showing the relationship between number of buildings and species richness keeping other factors constant in the model. Such effect plots are routinely used to plot the results of mixed models and are considered valid to predict the relationships between variables.<br>We have corrected the graph and included datapoints along with the prediction line.<br><br>Same comment as last figure (which is also labeled Fig 5. Is that intentional?) What is DR? Define in figure or in parentheses in caption.<br>AR: expanded and clarified<br>Fig 6 I suggest the axis title say "Proportion of rice paddy in 5 km radius" or similar<br>AR: After the new analysis using remote sensing categorization we could not do the analysis at individual crop level so this is excluded<br>Fig 8 This figure is difficult to read with No. villages plotted on top of the land use classes.<br>AR: Changed completely<br>Table 1: Nicely presented. Please indicate in the caption at what values are there considered an overlap? Higher values mean more overlap?<br>AR: We have removed this analysis<br>Table 4: Missing a variable after the "+" under Hiposederous speoris? Did you mention that you also had species-specific models in the main text?<br>AR: Modified<br><br><br>Reviewer 3<br><br><br>Made comments in the MS itself which is addressed therein
**Additional Information:**	
**Question**	**Response**
**Financial Disclosure**<br><br>Enter a financial disclosure statement that describes the sources of funding for the work included in this submission. Review the submission guidelines for detailed requirements. View published research articles from *PLOS ONE* for specific examples.<br><br>This statement is required for submission and **will appear in the published article** if the submission is accepted. Please make sure it is accurate.	The work was partly supported by a grant from Bat Conservation International Global Grassroots Conservation Fund. We thank Maria,Lise and K.S.Seshadri for help in the field. We are grateful to Dr.R.Ganesan, Dr.M.Soubadra Devy for their help during the initial stages of the work. We also tank Rajkamal, Seshadri, Prashanth and Soubadra for their comments on the MS. We thank Thamizhazhagan for helping in capturing bats and Muthupandi for assistance in the field. Abhishek helped us with landuse analysis and Thalavaipandi drew the map. The Temple authorities and temple staffs in both Tirunelveli and Thoothukudi districts permitted us to do the study. Jermiah Rajanesan, director Dhonavur Fellowship and Maria Joseph, Head Master, Christuraja Hr., Sec., School, Palayamkottai helped us with logistics and we are grateful to them for their help. There was no additional external funding received for this study

Powered by Editorial Manager® and ProduXion Manager® from Aries Systems Corporation

**Unfunded studies**<br>Enter: *The author(s) received no specific funding for this work.*<br><br>**Funded studies**<br>Enter a statement with the following details:<br>• Initials of the authors who received each award<br>• Grant numbers awarded to each author<br>• The full name of each funder<br>• URL of each funder website<br>• Did the sponsors or funders play any role in the study design, data collection and analysis, decision to publish, or preparation of the manuscript?<br>• **NO** - Include this sentence at the end of your statement: *The funders had no role in study design, data collection and analysis, decision to publish, or preparation of the manuscript.*<br>• **YES** - Specify the role(s) played.<br><br>* typeset	
**Competing Interests**<br><br>Use the instructions below to enter a competing interest statement for this submission. On behalf of all authors, disclose any competing interests that could be perceived to bias this work—acknowledging all financial support and any other relevant financial or non-financial competing interests.<br><br>This statement is required for submission and **will appear in the published article** if the submission is accepted. Please make sure it is accurate and that any funding sources listed in your Funding Information later in the submission form are also declared in your Financial Disclosure statement.<br><br>View published research articles from *PLOS ONE* for specific examples.	The authors have declared that no competing interests exist.

**NO authors have competing interests**<br><br>Enter: *The authors have declared that no competing interests exist.*<br><br>**Authors with competing interests**<br><br>Enter competing interest details beginning with this statement:<br><br>*I have read the journal's policy and the authors of this manuscript have the following competing interests: [insert competing interests here]*<br><br>* typeset	
**Ethics Statement**<br><br>Enter an ethics statement for this submission. This statement is required if the study involved:<br><br>• Human participants<br>• Human specimens or tissue<br>• Vertebrate animals or cephalopods<br>• Vertebrate embryos or tissues<br>• Field research<br><br>Write "N/A" if the submission does not require an ethics statement.<br><br>General guidance is provided below. Consult the submission guidelines for detailed instructions. **Make sure that all information entered here is included in the Methods section of the manuscript.**	No specific permit required

**Format for specific study types**<br><br>**Human Subject Research (involving human participants and/or tissue)**<br>• Give the name of the institutional review board or ethics committee that approved the study<br>• Include the approval number and/or a statement indicating approval of this research<br>• Indicate the form of consent obtained (written/oral) or the reason that consent was not obtained (e.g. the data were analyzed anonymously)<br><br>**Animal Research (involving vertebrate animals, embryos or tissues)**<br>• Provide the name of the Institutional Animal Care and Use Committee (IACUC) or other relevant ethics board that reviewed the study protocol, and indicate whether they approved this research or granted a formal waiver of ethical approval<br>• Include an approval number if one was obtained<br>• If the study involved *non-human primates*, add *additional details* about animal welfare and steps taken to ameliorate suffering<br>• If anesthesia, euthanasia, or any kind of animal sacrifice is part of the study, include briefly which substances and/or methods were applied<br><br>**Field Research**<br><br>Include the following details if this study involves the collection of plant, animal, or other materials from a natural setting:<br>• Field permit number<br>• Name of the institution or relevant body that granted permission	
**Data Availability**<br><br>Authors are required to make all data underlying the findings described fully available, without restriction, and from the time of publication. PLOS allows rare exceptions to address legal and ethical concerns. See the PLOS Data Policy and FAQ for detailed information.	Yes - all data are fully available without restriction

A Data Availability Statement describing where the data can be found is required at submission. Your answers to this question constitute the Data Availability Statement and **will be published in the article**, if accepted.<br><br>**Important:** Stating 'data available on request from the author' is not sufficient. If your data are only available upon request, select 'No' for the first question and explain your exceptional situation in the text box.<br><br>Do the authors confirm that all data underlying the findings described in their manuscript are fully available without restriction?	
**Describe where the data may be found in full sentences. If you are copying our sample text, replace any instances of XXX with the appropriate details.**<br><br>• If the data are **held or will be held in a public repository**, include URLs, accession numbers or DOIs. If this information will only be available after acceptance, indicate this by ticking the box below. For example: *All XXX files are available from the XXX database (accession number(s) XXX, XXX.)*.<br>• If the data are all contained **within the manuscript and/or Supporting Information files**, enter the following: *All relevant data are within the manuscript and its Supporting Information files.*<br>• If neither of these applies but you are able to provide **details of access elsewhere**, with or without limitations, please do so. For example:<br><br>*Data cannot be shared publicly because of [XXX]. Data are available from the XXX Institutional Data Access / Ethics Committee (contact via XXX) for researchers who meet the criteria for access to confidential data.*<br><br>*The data underlying the results presented in the study are available from (include the name of the third party*	Data are available from the ATREE Institutional Data Access / Ethics Committee (contact via tganesh@atree.org) for researchers who meet the criteria for access to confidential data.

and contact information or URL).<br>• This text is appropriate if the data are owned by a third party and authors do not have permission to share the data.<br><br>* typeset	
Additional data availability information:	Tick here if the URLs/accession numbers/DOIs will be available only after acceptance of the manuscript for publication so that we can ensure their inclusion before publication.

Powered by Editorial Manager® and ProduXion Manager® from Aries Systems Corporation

Manuscript

Click here to access/download;Manuscript;Manuscript.docx
Click here to download Manuscript Bats in temples of South India.docx

Temples the last abode for bats in a homogeneous agriculture landscape: Importance of microhabitat availability, disturbance and land use factors for bat conservation.

Authors: T.Ganesh, A.Saravanan and M.Mathivanan

Ashoka Trust for Research in Ecology and the Environment (ATREE),
Royal Enclave, Sriramapura, Jakkur Post, Bangalore 560064,
Karnataka state, India

Abstract

Anthropogenic structures such as temples in south Asia are widely used by cave-dwelling bats as roosting and nursery sites. These roosts are constantly under threat as temples are being renovated and bats removed from such places even more so after the COVID19 pandemic. No detailed understanding exists on what makes temples favorable for bats and what factors are important for their persistence. Here we relate temple microhabitat characteristics and land use around ancient temples (>400 years) to bat species richness and abundance in the Tamiraparani river basin of south India. Temples were selected for sampling along the basin based on logistics and permission to access them. Bats were counted at the roost in the mornings and late afternoons from inside the temples. Temple characteristics such as dark rooms, corridors, crevices and presence of tower were recorded after the count along with disturbances to the roost. We also recorded land use such as crop, trees, scrub, grassland, urban areas and water availability within 5 km radius of the temple based on ESA landuse classifications. We used GLMM to relate the counts in temples with microhabitat and land use. We sampled 59 temples repeatedly across 5 years which yielded a sample of 256 survey events. The total number of bats counted was 20,211 of which *Hipposideros speoris* was the most common (9,715) followed by *Rousettus leschenaulti* (5,306), *Taphozous melanopogon* (3,196), *Megaderma lyra* (1,497) *Tadarida aegyptiaca* (303), *Pipistrellus sp* (144) and *Rhinopoma hardwickii* (50). About 39% of the abundance was accounted by dark rooms while 51% by walkways around the deity room. Species richness and total abundance were related to the number of building in the temple and the availability of dark rooms. Land use elements only had a weak effect but scrub and grassland even though were few are important for bats. We conclude that retaining undisturbed dark rooms with small exits in temples and other dimly lit areas and having natural areas around temples are critical for bats conservation in the area.

Key words: Bats; microhabitat; Hindu temples; India; landscape,

Introduction

Many studies suggest that human-modified landscapes with a heterogeneous mosaic of different land-uses can preserve species-rich bat assemblages (Medellín et al. 200[illegible]oreno and Halffter 2001; Estrada and Coates-Estrada 2002; Harvey et al. 2006; Medina et al. 2007; Barragan et al. 2010; Mendenhall et al. 2014). This is possible with the concurrent availability of microhabitats that bats use as roosting sites ranging from caves, crevices, tree cavities, foliage, and several types of anthropogenic structures including road bridges and buildings (Voigt and Kingston 2016, Meierhofer et al. 2019). These microhabitats being important features of a bat's environment, the selection of specific roost sites can strongly impact the survival and fitness of bats (Vonhof and Barclay, 1996, Humphrey, 1975; Stebbings, 1995). However, emerging challenges such as changing agricultural practices and climate change pose major threats to bat assemblages and populations in human-dominated landscapes (Jones et al.2009, Frick et al 2019) and could negatively impact human populations by altering the ecosystem services that bats provide (Kunz et al. 2011, Williams-Guillén et al 2016). Moreover, bats that roost in anthropogenic structures are considered vulnerable to disturbance since just like cave roosting species they often form large, concentrated aggregations attracting attention (Furey, et al. 2016).

Most studies on the use of anthropogenic structures by bats have come from urban areas and under temperate conditions (Kunz et al., 2011; Jenkins et al., 1998; Clem, 1993, Fagan et al 2017,Meierhofer et al. 2019). While it is accepted that bats have been poorly studied in the Paleotropics (Meyer et al 2016), the selection of microhabitats and landscape-level influences on their population remains poorly known (McNab, 1982; Ho and Lee, 2003, Kerth et al., 2001). Most studies on bat–habitat relationships are done at fixed spatial scales (Rainho & Palmeirim 2011) but recent studies have shown the importance of scale for bats (Razgour, Hanmer & Jones (2011), Bellamy et al 2013. In tropical South Asia, several species of both insectivorous and frugivorous bats use temples as roosting sites (Bates and Harrison, 1997, Korad 2007). In south India numerous very old (>400y) temples dot the landscape and form important bat habitats (Swamidoss et al. 2012). These temples are made of granite stones and the entrance of the temple has towers (*Gopuram*) usually made of bricks. The temples are not airconditioned and the temperature inside is stable and warm (32^0 - 33^0 C) and fairly constant across the day (personal obs T.Ganesh). Bats enter and leave the roost through numerous vents that are left open for air to circulate inside the

temple.

Bat use of anthropogenic structures such as temples also makes it difficult to balance bat conservation with human-bat conflicts. The temples are situated in a human-dominated agricultural matrix where intensive paddy and banana cultivation is done. Agriculture is considered a major threat to bats (Frick et al. 2019) and with irrigated agriculture spreading to dry areas in the region, threats to bat population can be expected to rise in the future along with a predicted increase in rains(Tamil Nadu State Action Plan for Climate Change). Populations of bats in many temples have also dwindled (Vanitharani 2004, Mahandran et al.2015) and many are also directly killed during temple renovation (Addline et al 2004). The changing attitude of people towards bats has also led many temples to be made "bat-proof" by placing nets and power washing roost areas with chlorinated chemicals to prevent bats from colonizing. The recent COVID-19 pandemic has further made them more vulnerable to disturbance and direct persecution. In India, only two out of 120 species of bats ~~comes~~ under the strict Wildlife Protection Act 1972 while others ~~even though~~ are ~~threatened~~ under IUCN category, remain unprotected.

Management decisions for bats and buildings, including the timing of maintenance activities, restriction of human use, and bat deterrence or exclusion, require information on the roosting habits of temple-roosting bats. Studies in temperate regions have shown how features of bridges and buildings can help identify sites for conservation and thereby reduce bat-human conflict (Fagan et al 2017, Meierhofer et 2019) and such efforts are needed for conserving bats in tropical regions. ~~It's~~ therefore essential to understand what attributes of temples are important for bats and how such information can help articulate bat conservation efforts in ~~such~~ temple landscapes.

More than 50% of the 35 bat species found in Tamil Nadu state dwell in caves and old buildings (Bates and Harrison 1997). Except one *Rousettus leschenaultia*, a frugivore, all are insectivores. In the paddy agriculture-dominated landscape of south India, ancient temples are often the only habitat for ~~several~~ bat species. They provide critical ecosystem services for agriculture and in south Asia can save up to US$1.2 million per year from insect depredation (Wagner et al. 2014) and consume large quantities of mosquitoes thus providing human health benefits (Sophia 2010). Despite the numerous threats and the importance of bats in providing key ecosystem services in human-dominated landscapes (Wagner et al. 2014, Thet and Mya 2015), there is no systematic study on the ecology of bats in temples while behavioural studies have been carried out on them in South India (Audet et al. 1991, Leippert et al.2000).

Moreover**,** **w**ith populations of bats dwindling no attempt to monitor bats in the temples or how important are temples in an agricultural matrix to bats has been investigated. We initiated a bat monitoring program in 2012 in temples of south India to understand why bats use temples and how the population of species is responding to disturbance and land-use change. In this study, we identify temples used by bats for roosting in a semi-arid region characterized by the winter monsoon (Oct to Dec) and agriculture dominated by irrigated rice paddy and banana for 6-8 months a year. The rest of the ~~time~~ the land is fallow in most places. We record the species composition, rarity, and abundance of bats in temples and identify factors that promote or dissuade bat colonization such as microhabitat availability, disturbance in temples and landscape features surrounding the temples. While much of the bat diversity in human-dominated landscapes can be attributed to a combination of habitat diversity and roost site availability in an area, in tropical monoculture areas with rice paddies and banana and low habitat heterogeneity, it would be interesting to compare the role of land use elements and availability of microhabitats on bat species richness and abundances. In this paper, we test three major hypotheses 1.Disturbance at the roost negatively affects species richness and abundance of bats, 2. Roost characteristics are more important than landscape features for bats to colonize temples and 3. In a homogeneous monoculture cropping, species occurrence and abundance are independent of spatial availability of habitats around roosts.

Materials and Methods

Study area

The study was carried out in Tirunelveli & Thoothukudi Districts of Tamil Nadu state in South India.. The region is semi-arid and experiences the northeast winter monsoons between Oct-Dec which bring in 70% of the annual rainfall. The south west monsoon which is obstructed by the Western Ghats mountains brings in strong winds and light rain during June – Sep. The rest of the months are dry. Several ancient temples built during the Chola and Pandya dynasties 500 to 1000 years back exist in the districts along the perennial Tamiraparani river and its tributaries. The river and the numerous interconnected village ponds are the lifelines for Paddy and Banana dominated agriculture supporting a dense human population in the semi-arid landscape. The ancient temples are built of large granite stones and provide a habitat for several bat species. There are over 4041 temples (HR&CE, 2020) in the study area with 2-3 villages having one ancient temple. The typical structure of the temples includes a tower that stands at the four entrances and one over the deity, a deity chamber lit by an oil lamp and an electric bulb, a walkway surrounding the deity chamber for walking and prayer by devotees, and several rooms including storerooms and a kitchen which are dark and rarely frequented by people. The temples are usually located towards the corner of a village or town.

Temple selection

We selected 59 temples from the Tamiraparani basin and surveyed them with 2-4 personnel between 06:00-12:00 h and again between 16:00 to 17:00h to detect the presence of bats. The survey was done from May to Dec and the same temples were visited across the years 2012,2013,2014,2018,2019. for a total of 246 surveys
All species of both *Yinpterochiroptera* and *Yangochiroptera* bats found roosting inside the temples were recorded. The presence of bats in the temple was identified by several signs such as droppings and smell, interviewing local people, temple priests, and caretakers. ~~Later~~ dark corners, temple roof, abandoned ~~&~~ isolated rooms inside the temple and the temple tower were all scanned for chiropteran presence with a red LED light. Once bat colonies were detected, we counted the number of individuals and in some cases, especially in closed towers, we took photographs to estimate them.

Temple features

a. Microhabitat

There were multiple locations inside temples where bats could roost: Closed rooms with few small openings, very low natural light and not frequently used by people ~~were Dark Rooms;~~ stone pillars ~~that support the stone roof is a separate habitat as they provide a niche for few species that roost in the~~ gaps created between the stone joints ~~this was crevices;~~ temple towers which were conical structures built over the entrance or the deity and wide walking space around the deity room covered by a stone roof are walkways. We also counted the number of independent buildings in the temple complex as bats could move between them during the day due to disturbance. In 2012 we measured temperature and humidity while sampling for bats and found no difference between temples and therefore not considered in the analysis. We could not set dataloggers for continuous monitoring because of objections by the temple authorities to placing any devices inside temples. ~~Temple size estimated based on~~ visual observation; those with deity rooms and walkways were considered as small, those with deity rooms, walkways and towers considered as medium-sized, and those having deity rooms, multiple towers, and two walkways (inside and outside) were considered as large. We also recorded the number of trees in the temple complex and open wells used by bats. The basic structure of the temple remained the same with more walkways and towers included as they became big.

b. **Disturbance**

Two factors that directly impact bats at the roost are structural impacts like renovation of the temple premises and human presence. Renovation of temples like power washing the walls, reconstructing parts of the building or any other maintenance work was on a scale from 0 to 1. Zero being no renovation activities in the temple and 1 when it was present. Information on visitors to the temple, obtained from temple authorities and scaled from 1 to 3. Three pertains to large crowds and 1 attracting few people.

Land-use features

Land-use features can be easily measured from satellite-based land-use classification however in this case we could not do that for each sampling period because of high cloud cover during that time. We, therefore, used the composite analysis of land cover available using high-resolution(10m) Esri data for 2020 (Karra et al.2021) to identify major land uses in the region

around temples. We could not obtain such information for each sampling year. The land uses were already categorized as water, scrub, grassland, bare ground, flooded area, crop, urban and tree-covered areas (Karra et al.2021). We combined, bare ground and grassland as grassland habitat and water and flooded area as water habitat after verifying the actual land-use on the ground and using Google Earth. Crop availability each season was obtained from Google Earth images when available and further substantiated by informal interviews with farmers around each temple. If crops were present we used the Esri calculated value of crop area in the analysis and if not we considered it as zero. The extent of other land uses has not changed much during the sampling period based on the data available with the district administration (Statistical handbook of Tirunelveli district 2009-2019).

Since the spatial scale is important for bat foraging (Bellamy and Scot 2013) we categorized land use at 500m, 1km,3km and 5km from the temple based on the foraging distance of bats from their roost (Audet et al 1991). We also recorded trees and water availability (open wells) which are often used by bats in the temple complex, for land-use analysis at 0km level.

Species identification and abundance

Bats were identified to species based on detailed photographs taken in the temple using the bat identification guide by Bates and Harrison (2001).

Bats were counted at the roost when they were least active (late morning and early evenings) using a flashlight with red filters. Where possible photographs were taken as soon as bat aggregations were noticed in a particular microhabitat inside the temple and later counted to estimate abundance (Carpenter, 2014). Several pictures were taken in quick succession to estimate and avoid bias in counting. Binoculars were used to see the species characteristics for identification. We could not do a roost exit count in the evening as it was not logistically possible to be in each temple at the right time for the count.

Data analysis

If more than one roost was found in a temple, we pooled roost counts and recorded total species richness for the temple. We constructed a linear model to explain the species richness and abundance of bats in the temples based on the microhabitat and landscape features. We based the variables used in our linear models on our observations made in 2012. We eliminated variables that had were strongly correlated (>0.70) with other variables to reduce variable redundancy and

retained ones that are biologically meaningful. We related the total species richness and abundance(counts) of bats at the temple with temple variables and landscape elements using mixed models. Since temples were sampled more than once and repeated measurements on the same temple are often correlated we accounted for this by using random effects in the mixed models (Bolker et al., 2009). We used lmer package in R for species richness as it was normally distributed, and since the bat counts were overdispersed, we used a negative binomial with glmmadmb package in R (Bolker et al 2012).We considered temple ID and year as a random factor in the model and temple characteristics, disturbance and land use elements as fixed factors. We used the function *dredge* in MuMIn package(version 1.43.17) in R to obtain all possible combinations of models using the select variables. We fitted two models one for temple characteristics with disturbance and the other for land use elements at 5 spatial scales; 0m,500m, 1km,3km and 5km. The models were ranked using Akaike's Information Criterion corrected for sample size and based on calculated Akaike weights of each model. Model averaging was done for closely related models following(Mazerolle 2020) using model selection criteria in MuMIn package(version 1.43.17) in R. We calculated model-averaged parameter estimates and unconditional standard errors to assess the relative importance of each variable and account for model uncertainty (Burnham and Anderson 2002). We considered only parameters with 95% confidence intervals and not overlapping with 0 to be informative (Payton et al. 2003). We tested for differences in microhabitats and land-use between species using Kruskal Wallis and Mann Whitney tests. Data analysis and statistical tests were carried out using Microsoft Excel®, PAST® and R version 4.

Results

Species richness and abundance

Forty-seven of the 59 temples surveyed housed bats and 51 of these had multiple roosts and only 8 had a single roost across years. The total number of roosts sampled was 351 across 246 surveys in 5 years. Seven species of bats were recorded of which six were Yinpterochiroptera and one *Rousettus leschenaulti* was a Yangochiroptera **(Table 1)**. Among the Yinpterochiroptera, *Hipposideros speoris* was the most frequently seen species found in 135 (38%) roosts sampled followed by *Taphozous melanopogon (58), Megaderma lyra (29) Tadarida aegyptiaca (26) Rousettus leschenaulti (22), Pipistrellus sp (9) and Rhinopoma hardwickii (7)*. Forty-five % of the temples had only one species, 34% had two species, 15% had three species and 6% had four species of bats. In all 20,211 bats were counted of which

Hipposideros speoris(HS) was the most common (9715, Range 2-1150) across temples followed by *Rousettus leschenaultia(RL)* (5,306, 1-2946), *Taphozous melanopogon* (TM) (3,196, 1-1165), *Megaderma lyra* (ML)(1,497, range 20-605) *Tadarida aegyptiaca (TE)* (303, 3-84), *Pipistrellus sp (PS)* (144) and *Rhinopoma hardwickii* (RH)(50). None of the bat species recorded ~~is~~ "threatened" as per the International Union for Conservation of Nature (IUCN).

Table 1. Species characteristics and IUCN status of bats recorded in temples. The breeding season in parenthesis is based on the present study.

Species name	IUCN	Diet	Breeding season	Roosting habitat
Rousettus leschenaultii	LC	Flowers and fruits	Nov-March	Temples, old buildings, caves
Rhinopoma hardwickii	LC	Moths, neuropteran insects and beetles	Feb-Mar	Crevices, roof, houses, temples, boulders
Hipposideros speoris	LC	Beetles, termites, low flying insects and flies	Dec-Mar (Dec)	crevices in hills, caves, caverns, disused buildings, tunnels and temples
Megaderma lyra	LC	Insects, reptiles, fishes and birds	Nov-Apr	caves, temples, forts, dilapidated old buildings, underground tunnels, old cow sheds, grain godowns, shallow soap stone mines and attics of houses
Taphozous melanopogon	LC	Insects	Jan-June (Dec-June)	ruins temples and caves, dark dungeons in the old fort
Tadarida aegyptiaca	LC	I Caterpillar, spider, water beetle, ground-dwelling insects	June-Sep	Crevices in cliff faces, crevices in large piles of rocks and boulders, narrow spaces formed by slabs of stone leanings against walls, the expansion joints at the top of supporting pillars in modern grain store, narrow cracks in a pillar of old mosques and crevices in buildings and old forts
Pipistrellus tenuis	LC	Small insects, beetle, cockroach, termite, grasshopper	Feb-march, July-Aug (Dec)	Roofs of bungalows, holes and crevices in walls, hollow branches of trees, dead leaves of trees

Bats and Roost characteristics

We distinguished four separate microhabitats inside the temple that were used for roosting by the seven species (**Fig 1**). About 39% of the total abundance was in dark rooms while 51% of bats were in walkways around the deity room which are the main microhabitats for 5 out of 7 species. *Tadarida aegyptiaca*, *T. melanopogon* and *Pipistrellus sp* Crevices preferred pillars.

Fig 1. Percentage of microhabitats used by the different species of bats for roosting.

In terms of species occupancy, *H. speoris* occupied three out of four microhabitats, but most commonly dark rooms; walkways were used only when darkrooms were not available or disturbed. Likewise *M. lyra* and *T. melanopogon*, *R. hardwickii* were found in two microhabitats (walkways and crevices) while *T. aegyptiaca* was found only between pillars(crevices), and *Pipistrellus sp* found only in roof crevices. The fruit bat *R. leschenaulti* preferred walkways.

Temple and disturbance.

The number of buildings and Dark room availability in the temple complex significantly affected species richness and bat abundance (counts) in temples **(Fig 2, Table 2).** The top lmer model explained 69% of the variation in species richness of which 58% were explained by the fixed factors.

Fig 2. Effect plots showing the relationship between species richness, abundance and number of buildings and dark rooms in temples

Table 2: Effects of Temple characteristics on species richness (lmer model) and bat abundances(glmmadmb model). Temple id and year are included as random factors.

Temple model with disturbance										
**Species richness**										
	Estimate	2.500	97.500	Std. Error	Models	df	logLik	AICc	delta	weight
**(Intercept)**	-0.052	-0.291	0.187	0.122	BLD+DR+REN	7	-171.90	358.27	0.000	0.600
**No. buildings**	0.674	**0.587**	**0.761**	0.044	BLD+REN	6	-173.38	359.11	0.850	0.400
**Dark rooms**	0.246	**0.044**	**0.448**	0.103						
**Renovation**	-0.364	**-0.524**	**-0.204**	0.081						
**Bat Abundance**										
**(Intercept)**	1.817	-0.498	4.132	1.177	BLD+DR+REN+TWR+WAY	8	-1118.67	2253.94	0.000	0.570
**No. buildings**	0.802	**0.397**	**1.206**	0.206	BLD+DR+REN+VIS+WAY	9	-1117.88	2254.52	0.570	0.430
**Dark rooms**	1.597	**0.541**	**2.654**	0.536						
**walkway**	1.678	-0.404	3.761	1.057						
**Renovation**	-0.319	-1.016	0.378	0.354						
**Visitors**	-0.869	**-1.473**	**-0.265**	0.307						
**Tower**	0.349	-0.532	1.230	0.447						
Hiposederous speoris										
**(Intercept)**	-0.923	-5.439	3.594	2.296	BLD+DR+REN+VIS	8	-766.21	1549.02	0.000	0.400
**No. buildings**	0.769	**0.090**	**1.448**	0.345	DR+VIS	6	-768.88	1550.12	1.090	0.230
**Dark rooms**	2.731	**0.367**	**5.095**	1.200	BLD+DR+REN+TWR+VIS+WAY	10	-764.79	1550.51	1.480	0.190
**Renovation**	-0.323	-1.509	0.863	0.602	BLD+DR+REN+TWR+VIS	9	-765.92	1550.60	1.580	0.180
**Visitors**	-0.559	-1.877	0.759	0.669						
**walkway**	3.631	-0.948	8.209	2.324						
**Tower**	-0.748	-2.566	1.069	0.922						
Megaderma lyra										
**(Intercept)**	-10.230	-16.610	-3.849	3.243	BLD+REN	6	-176.56	365.46	0.000	0.660
**No. buildings**	0.982	-0.162	2.126	0.581	BLD+VIS+REN	7	-176.16	366.78	1.320	0.340
**Renovation**	-3.462	**-6.074**	**-0.850**	1.326						
**Visitors**	-1.769	-6.113	2.575	2.205						
Tadarida aegyptiaca										
**(Intercept)**	-6.086	-12.775	0.603	3.413	BLD+REN+VIS	7	-129.49	273.50	0.000	0.567
**Renovation**	-3.723	**-7.216**	**-0.230**	1.782						
**visitors**	-2.231	-6.036	1.575	1.942						
**No. buildings**	1.083	**0.145**	**2.021**	0.479						
Taphozous melanopogon										
**(Intercept)**	-14.690	-25.594	-3.786	5.538	BLD+REN+VIS	7	-265.48	545.42	0.000	0.680

**No. buildings**	0.758	**0.132**	**1.384**	0.318	BLD+DR+REN+VIS	8	-265.14	546.89	1.460	0.320
**Renovation**	-1.181	-2.659	0.297	0.750						
**visitors**	2.312	-1.594	6.217	1.983						
**Dark rooms**	2.576	-4.351	9.503	3.516						
Rousettus leschenaulti										
**(Intercept)**	-9.636	-16.172	-3.100	3.318	BLD+VIS	6	-190.46	393.27	0.000	0.440
**No. buildings**	1.163	-1.296	3.622	1.248	DR+REN	6	-191.10	394.55	1.290	0.230
**visitors**	-0.913	-3.988	2.161	1.561	BLD+REN+VIS	7	-190.36	395.18	1.920	0.340
**Dark rooms**	0.687	-4.871	6.245	2.822						
**Renovation**	0.667	-2.645	3.979	1.681						

Renovation of temple structures had a strong negative influence on bat species richness while the scale of visitors to the temple negatively affected abundance (**Fig 3**). Species wise response to microhabitat and disturbance varied (**Table 2**). *Hiposederous speoris* abundance was positively influenced by dark rooms and the number of buildings but not by any disturbance factors. Counts of *T.melanopogon* and *T.aegyptica* were also positively related to the number of buildings in the temple but were also affected negatively by renovation. *M.lyra* also showed a negative response to renovation while *R.leschenaulti* was not affected by any temple characteristics or disturbance.

Fig 3. Effects plots showing the relationship of disturbance on species richness and abundance.

Landscape

The percentage of land-use around the temples changed with distance. At 5km from the temple crops (63%) dominate followed by urban areas (13%), scrub (12%), trees (7%) water (5%), grass and bare areas (0.23%) ~~and at~~ 500m; crop (54%) continued to dominate followed by urban areas (36%), scrub (0.7%), trees (3%) water (6%), grass and bare (0.09%). Since temples are close to settlements urban area is high near the temple and crop, trees, scrub and grass areas are less (**Fig 5**). Grassland around the temples especially at 3km positively affected species richness and scrub availability positively affected the abundance while urban areas had a negative effect at 5km. Trees in the temple complex had a positive effect on species richness (**Table 3**). Scrub availability also had a positive effect on *Hiposederous speoris* abundance across the distance interval. Trees had a negative effect and were strongest at 1km (**Table 3**). M.lyra and

T.aegyptica both were negatively affected by crops up to 5km while. *T.melanopogon* and *R.leschenaulti* did not show any effect of land use on their roost abundance.

Fig 4. High resolution (10m) land-use around the temples sampled. Land-use classification obtained from Karra et al 2021. In the analysis grass and bare areas, and water and flooded areas were combined. See data analysis for more details.

Table 3: Species response to land-use elements. Model averaged parameter estimates of top models (<2 ΔAICc) with non-overlapping confidence intervals shown in the last column*

Species/Abundance	Distance (m)	Parameters	Estimate	2.50%	97.50 %	Std.Err	Strong association
Species richness	0	Intercept	0.829	0.503	1.155	0.166	
		Water	0.029	-0.144	0.202	0.088	
		Trees	0.006	**0.002**	**0.010**	0.002	*
	500	Intercept	0.937	0.694	1.180	0.123	
		Grassland	-0.071	-0.398	0.255	0.166	
	1000	Intercept	0.940	0.695	1.184	0.124	
		Grassland	-0.197	-0.982	0.588	0.399	
	3000	Intercept	0.793	0.541	1.071	0.128	
		Grassland	0.653	**0.190**	**1.120**	0.235	*
	5000	Intercept	0.861	0.548	1.173	0.159	
		Grassland	0.618	-0.205	1.441	0.418	
Abundance	0	Intercept	2.917	1.963	3.870	0.484	
		Trees	0.017	**0.000**	**0.033**	0.008	*
		Water	-0.174	-0.804	0.456	0.320	
	500	Intercept	3.307	2.587	4.027	0.366	
		Trees	-0.032	-0.097	0.032	0.033	
		Grassland	0.184	-1.330	1.698	0.769	
		Water	-0.001	-0.061	0.059	0.030	
		Scrub	0.142	-0.088	0.371	0.116	
	1000	Intercept	3.229	1.964	4.495	0.643	
		Scrub	0.176	0.057	0.294	0.060	
		Crop	0.002	-0.007	0.011	0.005	
		Trees	-0.144	-0.237	-0.051	0.047	*
		Urban	0.016	-0.018	0.051	0.018	
		Water	-0.019	-0.089	0.051	0.035	
		Grassland	-0.094	-3.954	3.767	1.960	
	3000	Intercept	3.282	2.007	4.557	0.648	
		Scrub	0.066	-0.004	0.136	0.036	
		Trees	-0.064	-0.133	0.004	0.035	
		Grassland	0.291	-1.387	1.968	0.851	
		Water	-0.084	-0.289	0.120	0.104	
		Urban	0.035	-0.059	0.129	0.048	
	5000	Intercept	7.065	3.306	10.824	1.913	
		Water	-0.498	-0.864	-0.132	0.186	*
		Urban	-0.139	-0.267	-0.010	0.065	*
		Trees	-0.077	-0.137	-0.017	0.030	*
		Crop	0.001	-0.008	0.011	0.005	
		Grassland	-0.023	-3.133	3.087	1.579	
		Scrub	0.069	0.002	0.137	0.034	*
Hiposederous speoris	0	Intercept	1.591	0.171	3.011	0.724	
		Trees	-0.004	-0.032	0.025	0.015	
	500	Intercept	1.681	-0.070	3.431	0.890	
		Grassland	1.091	-1.123	3.304	1.124	

		Urban	-0.036	-0.080	0.008	0.022	
		Scrub	0.246	-0.128	0.621	0.190	
		Water	0.017	-0.085	0.119	0.052	
		Trees	-0.005	-0.112	0.102	0.054	
	1000	Intercept	1.583	-0.295	3.460	0.954	
		Scrub	0.275	0.059	0.492	0.110	*
		Crop	0.001	-0.014	0.016	0.007	
		Trees	-0.173	-0.333	-0.013	0.081	*
		Urban	-0.026	-0.089	0.037	0.032	
		Water	0.015	-0.106	0.135	0.061	
		Grassland	0.444	-5.734	6.622	3.136	
	3000	Intercept	0.672	-0.668	2.012	0.684	
		Scrub	0.124	0.009	0.240	0.059	*
	5000	Intercept	2.098	-2.593	6.789	2.389	
		Grassland	-0.595	-6.040	4.850	2.765	
		Scrub	0.112	-0.007	0.231	0.060	
		Water	-0.275	-0.972	0.423	0.354	
		Urban	-0.197	-0.446	0.051	0.126	
		Trees	-0.002	-0.101	0.097	0.050	
Megaderma lyra	0	Intercept	-10.086	-14.399	-6.069	2.219	
		Trees	0.006	-0.057	0.068	0.032	
		Water	-0.064	-1.373	1.648	1.252	
	500	Intercept	-9.165	-12.658	-5.673	1.773	
		Grassland	-1.701	-22.185	18.783	10.399	
		Crop	-0.038	-0.074	-0.002	0.018	
		Water	-0.053	-0.387	0.282	0.170	
	1000	Intercept	-8.910	-12.676	-5.143	1.912	
		Grassland	-2.381	-29.684	24.921	13.860	
		Crop	-0.031	-0.060	-0.001	0.015	
		Water	-0.130	-0.769	0.509	0.324	
	3000	Intercept	-9.571	-13.736	-5.407	2.115	
		Grassland	-0.325	-9.084	8.435	4.447	
		Crop	-0.028	-0.059	0.003	0.016	
		Water	-0.168	-1.223	0.887	0.536	
	5000	Intercept	-9.432	-14.681	-4.182	2.666	
		Grassland	-1.791	-16.318	12.736	7.375	
		Crop	-0.027	-0.061	0.006	0.017	
		Water	-0.103	-1.593	1.386	0.756	
		Trees	0.008	-0.220	0.236	0.116	
Rousettus leschenaulti	0	Intercept	-10.086	-14.457	-5.715	2.219	
		Trees	0.006	-0.057	0.068	0.032	
		Water	-0.064	-2.531	2.403	1.252	
	500	Intercept	-9.441	-13.064	-5.819	1.839	
		Trees	-0.354	-1.823	1.115	0.746	
		Grassland	-2.717	-28.261	22.827	12.968	

		Water	0.029	-0.167	0.224	0.099	
	1000	Intercept	-9.219	-13.146	-5.291	1.994	
		Trees	-0.381	-1.805	1.043	0.723	
		Water	0.013	-0.251	0.277	0.134	
	3000	Intercept	-9.805	-14.066	-5.543	2.164	
		Trees	-0.159	-0.928	0.610	0.391	
		Urban	0.078	-0.220	0.375	0.151	
		Grassland	1.390	-4.757	7.536	3.120	
	5000	Intercept	-10.148	-15.311	-4.984	2.622	
		Water	0.241	-0.913	1.395	0.586	
		Grassland	2.023	-8.862	12.908	5.526	
		Trees	-0.049	-0.391	0.294	0.174	
		Crop	-0.010	-0.054	0.035	0.023	
		Scrub	0.054	-0.207	0.315	0.132	
Tadarida aegyptiaca	0	Intercept	-9.608	-14.070	-5.146	2.266	
		Water	0.555	-1.653	2.763	1.121	
		Trees	0.001	-0.057	0.059	0.030	
	500	Intercept	-8.367	-12.152	-4.581	1.922	*
		Grassland	-1.819	-19.858	16.221	9.158	
		Crop	-0.023	-0.046	0.000	0.012	*
		Urban	-0.010	-0.109	0.088	0.050	
		Trees	-0.317	-1.849	1.215	0.778	
		Water	-0.085	-0.484	0.314	0.203	
	1000	Intercept	-7.652	-11.239	-4.065	1.821	
		Grassland	-24.772	-149.996	100.453	63.572	
		Crop	-0.022	-0.043	-0.001	0.010	
		Water	-0.081	-0.506	0.344	0.216	
	3000	Intercept	-8.235	-13.475	-2.995	2.663	
		Grassland	0.812	-5.570	7.193	3.240	
		Crop	-0.022	-0.040	-0.004	0.009	
		Water	-0.487	-1.728	0.755	0.630	
		Urban	0.061	-0.228	0.350	0.147	
		Trees	-0.031	-0.344	0.283	0.159	
	5000	Intercept	-8.291	-14.453	-2.129	3.130	
		Grassland	-0.488	-12.427	11.452	6.061	
		Crop	-0.024	-0.044	-0.004	0.010	*
		Water	-0.368	-1.794	1.058	0.724	
		Urban	0.117	-0.338	0.572	0.231	
		Trees	-0.022	-0.264	0.220	0.123	
Taphozous melanopogon		Intercept	-8.476	-12.411	-4.542	1.998	
		Water	-0.997	-3.967	1.973	1.508	
		Trees	0.010	-0.051	0.071	0.031	
	500	Intercept	-9.273	-12.846	-5.699	1.815	

		Trees	-0.096	-0.837	0.645	0.376	
		Water	0.029	-0.176	0.233	0.104	
		Grassland	0.322	-3.117	3.760	1.746	
		Urban	0.047	-0.052	0.147	0.051	
		Crop	-0.007	-0.036	0.021	0.015	
	1000	Intercept	-9.137	-12.374	-5.901	1.644	
		Grassland	1.637	-5.136	8.410	3.438	
		Water	0.056	-0.195	0.308	0.128	
		Trees	-0.032	-0.425	0.362	0.200	
		Crop	-0.012	-0.030	0.006	0.009	
		Urban	0.029	-0.100	0.157	0.065	
	3000	Intercept	-8.635	-13.813	-3.456	2.633	
		Grassland	7.892	-1.886	17.670	4.964	
		Urban	-0.208	-0.693	0.276	0.246	
		Water	0.302	-0.337	0.940	0.324	
		Scrub	0.050	-0.162	0.261	0.107	
		Trees	-0.059	-0.502	0.384	0.225	
	5000	Intercept	-9.437	-15.329	-3.546	2.994	
		Grassland	5.918	-7.637	19.473	6.883	
		Trees	-0.091	-0.514	0.332	0.215	
		Water	0.198	-0.856	1.252	0.535	
		Crop	-0.009	-0.025	0.006	0.008	
		Urban	-0.262	-0.930	0.405	0.339	
		Scrub	0.081	-0.171	0.333	0.128	

The availability of roost microhabitats across temples with and without bats was not significantly different (Kruskal Wallis test; D=0.4, p=0.689), **Fig 5**). Of the total temples sampled, 80% of them had bats across all the years and in only 4/57 temples we found bats abandoning the temple. Renovation levels were higher in temples with no bat 44% (15/34) compared with temples having bats 35%(16/46) ~~similarly~~, visitor levels were also marginally higher in no bat (2.11+0.79) compared to temples with bats(1.89+.71). Land use between bat occupied and not occupied temples showed no differences except for trees in the vicinity of temples which was higher in bat occupied sites (Mann-Whitney U=3917 $p<0.01$) (**Fig 6**).

Fig 5.Proportional availability of habitats in temples with and without bats.

Fig 6. Mean land-use elements around temples with and without bats at various distances. * indicate significant differences at p< 0.05.

Discussion

Why do bats prefer some temples? Bats were found in about 80% of the temples and over 20,211 individuals were counted. Though we recorded only 7 species the most common species *Hipposideros speoris* (9715) accorded for nearly half of the population and also found in more than 50% of bat inhabited temples. *Hipposideros speoris* is a very common species across India and Srilanka and occurs in large congregations and shows high fidelity to roost sites (Selvarathinam and Marimuthu, 2013). The other 6 species are also not rare or threatened and use a multitude of anthropogenic structures to roost (Bates and Harrison, 1997). Except for *T. aegyptiaca*, *Pipistrellus sp* and *Rhinopoma hardwickii* species counts of all others ranged between 450 to 200 in each temple and appear to follow the general tendency of bats that roosts in caves and manmade structures, to be highly gregarious (Gaisler, 1979). Crevice roosting *T. aegyptiaca* and *Pipistrellus sp* species may have been underestimated because they are hard to count inside the narrow crevices.

Several species that roost and breed in temples across India are cave-dwelling species that have found temples as suitable alternative habitats(Bates and Harrison, 1997; Leippert et al., 2000). Our results show that temples offer dark, undisturbed roosts and crevices similar to caves and these harbours 6-7 species accounting for 80% of the abundance of bats in the temples. While we have pooled different types of dark spaces available in the temples, the one behind the main door of the temple and through which the temple tower can be accessed is occupied whenever there was no disturbance. These dark areas in old temples have several openings through which bats can easily fly out, a reason for their selection by most bat species. Granite stones used to build the ancient temples are stacked together and offers crevices for some species to roost such as the free-tailed bats (*T. aegyptiaca*) which often roost at eye level between pillars. Walkways surrounding the temple *sanctum* where the idol of the god is often long and dark during the day with a high roof which is a preferred place for the bats like *H. speoris* that remain unnoticed and therefore undisturbed during the morning hours when there is a movement of people. A similar response of cave-dwelling bats from genus *Hipposideros* has been found in Malaysia where they roost in very dark corners and high places (Rajasegarn et al 2018). We did not take species-wise roost height measurements so it’s not evident if species segregate roost according to height but unlike in a cave, many species that roost in dark corners seem to segregate spatially and can vary between temples. This requires some additional work.

This study demonstrates that disturbance to roosts is a major factor affecting roosting bats as many other similar studies have demonstrated in old buildings under temperate conditions (Kunz and Reynolds 2003, Fagan et al 2017). The effect was less impactful on the abundant *H.speoris* while other species are negatively affected by renovation and visitor levels. Large temples usually have more visitors but also more buildings and the possibility of harbouring more roosts and therefore even when disturbance levels increase bats might persist by moving to other buildings in the temple temporarily. While there could be several other factors affecting bats in temples like noise levels which can be of very high decibel levels during festivals, overlighting during festivals, prayer related smoke and day-long activities during festivals we could not measure these but needs to be considered when making management decisions for bat conservation. However, in many cases, these disturbance activities are routine in temples and bats have likely ~~got~~ accustomed to these threats but the recent spurt in the renovation of old temples where dark areas are lit, pressure cleaned to remove the odour of bats, painted and 'bat proofed' to keep away bats and pigeons from entering the premises is a major ~~one~~ and can effectively prevent bats from colonizing temples (Ganesh et al 2020). With current coronavirus and bat association being misreported by media bats have come under even greater threat and surveillance. Our surveys of bats in the same temples have shown that they have deserted some temples completely because of disturbances such as renovations (Selvarathinam and Marimuthu 2013).

While temple characteristics and disturbances are major drivers of bat abundance and occupancy, land use seems to play a less important role across species. Several studies have shown the importance of landscape elements for the presence of bats (Ford et al., 2006; Neubaum et al., 2007, Bellamy and Scot, 2013, Treitler et al. 2016) but in our case, there seems to be no single habitat effect. Fine-scale (10m) mapping of land-use around temples revealed differential use of habitat elements by species but these were either weak effects or had high variations in the effect. The landscape as mentioned earlier is highly homogeneous; crops and urban areas together constitute about 70-80% with scrub, grasslands, water bodies and trees comprising the rest in small proportions. Even though crops were everywhere they had a low negative effect on *M.lyra* and *T.aegyptiaca* while trees and water availability seem to have a consistent negative influence across species. The availability of scrub and grasslands is critical for species richness and the overall abundance of bats. These are the only two natural habitats available the rest being tree plantations, invasive *Prosopis juliflora* and artificial wetlands. Even though scrub and grasslands are less than 5% of the area it has a disproportionate and mostly positive effect on all the species except *M.lyra.* The radio-tracking study in south India of the insectivorous *M.lyra* showed it has a very flexible diet and can take prey both from air and ground which could enable it to use multiple habitats but prefers to forage mostly within 500m from temples(Audet et al 1991). The importance of natural habitat is emphasized for many species including bats(Razgour et al 2011) and this is evident in this study. Effect of water was variable with species like *T. aegyptiaca* seen to prefer water points in the vicinity of the temple but this may not be for water as large open wells in some temples are active sites for foraging. Water availability is important for bats and may drive bat movement and activity across the landscape (Geluso and Geluso, 2012; Rainho and Palmeirim, 2011) and studies in Thailand on similar species have shown insect diversity is less influential than water availability (Suksai and Bumrungsri 2018).

However, in this study, the effect was weak and negative for most species. This could be because there is no constraint for water availability in the region because of the perennial river and extensive irrigation ponds and excessive water can have a negative effect especially when the sampling was done during the cropping season.

Scale issues are important for many species including bats(Bellamy and Scot, 2013) and in this study at 5km from the roost most of the land-use effects became negligible or the direction of effects changed from positive to negative or vice versa. The extent of natural habitat increases with distance and this may influence bats to forage at larger spatial scales. Temples are located mostly close to human habitations and rivers while natural open dry habitats are further away and bats may be making regular forays as observed in UK bats (Razgour et al 2011). This corroborates with less preference for water and urban areas with distance when more suitable habitats are available.

This study provides a very broad and therefore limited view of land-use effect on bats. More nuanced study and analysis will need to use radio telemetry and acoustic sampling to see how land use elements influence bats. Foraging bats are most strongly associated with variables measured at small spatial scales and distance measures (Bellamy and Scot 2013). For instance, the only study on radio telemetry of temple bats shows 41% of the time the bats foraged 500m from the temples but ranged up to 4km over the night during the summer months. This may be different in rainy months when the general productivity of the habitat is more and larger use of the landscape may happen as scrub and grassland habitats also become richer in insect fauna during the rains. Further, the land-use change has to be included along with seasonal changes in bat occurrence which have not been entirely possible in this study but with more advanced remote sensing tools this is possible. A recent study in the same study area using radar images has shown a greater grassland area than by conventional means used here(Samrat et al 2021). Such an approach will also enable mapping across seasons irrespective of cloud cover.

The current study relies on the most recent high-resolution land-use analysis (Karra et al.2021) but the study was done in 2012-2019. This mismatch as mentioned earlier may not be very high given the land-use has remained largely the same over this period. However, for small-sized land-use such as natural habitats with less than 5% of the area and which are critical for bats, even small changes can result in large differences for a foraging animal. Further, the dynamics of wetlands has not been incorporated in this study but since it was mostly in the wet seasons we assume it to be stable across years but a more nuanced classification of these dynamics around

each temple might be important as irrigation systems and water availability are driven not entirely by monsoon patterns in the region (Nikil 2020).

The presence of bats is generally linked to microhabitat or stand-level structural conditions, however, Lewis (1995) also found that roost-site fidelity for bats varied depending on the availability and permanency of the roosts used and suggested that fidelity was high at buildings for these reasons. Supporting this a study on banded *H.speoris* bats from the study area showed 82% roost fidelity throughout the year (Selvarathinam, G Marimuthu 2013). Therefore the temples that have remained unchanged for centuries provide conditions for high roost fidelity despite changes in the landscape and therefore are more important for bats to persist in the area.

The importance of open uncultivated natural areas such as scrub, grasslands and lightly wooded areas are important for almost all the species of bats and this can have significant conservation importance as natural areas are rapidly diminishing.

Conservation of bats in temples

The study has shown that microhabitats within temples such as dark corners that are relatively less disturbed and rooms that were rarely used are essential for the persistence of bats. Temples with numerous buildings increase the possibility of having dark rooms. The negative effect of disturbance on bats is strong and can override the availability of microhabitats with temples having similar microhabitats not harbouring bats because of disturbance. This effect of disturbance is species-specific with the frugivores r.leschenaulti and insectivorous H.speoris are not affected by them which is due to their overall ability to return to roost sites after disturbance while others are more sensitive. Therefore conservation of bats would mean not just protecting the microhabitat in the temples from excessive disturbance but also allowing bats to use such spaces and not bat-proofing the temples. Most of the landscape factors were surprisingly not important though the availability of natural areas around temples are crucial but are also limited. While bats are known to provide important pest control services in paddy agriculture the role of paddy in sustaining the bat populations is not clear as crops did not have any significant effect on bat species richness and abundance. This calls for a more nuanced study using radio telemetry and acoustic sampling to understand the use of the landscape by bats since natural areas are likely to be converted in the future with a greater push for irrigated agriculture in the area. To make bat conservation a win-win situation bats roosting in public

and historical buildings need to be managed to promote their persistence, mitigate human-wildlife conflict, prevent potential zoonotic disease transmission and prevent bat guano and urine from damaging the historical integrity of structures like temples. This can be done with faith groups, temple management and by building awareness among the public to develop a comprehensive plan to let bat persist on the landscape and continue to ~~benefit from the~~ ecosystem services ~~they provide rather than ad-hoc elimination of bats in temples.~~

Acknowledgement

We thank Maria, Lise and K.S.Seshadri for help in the field. We are grateful to Dr.R.Ganesan, Dr.M.Soubadra Devy for their help during the initial stages of the work. We also tank Rajkamal, Seshadri, Prashanth and Soubadra for their comments on the MS. We thank Thamizhazhagan for helping in capturing bats and Muthupandi for assistance in the field. Abhishek helped us with land use analysis and Thalavaipandi drew the map. The Temple authorities and temple staffs in both Tirunelveli and Thoothukudi districts permitted us to do the study. Jermiah Rajanesan, director Dhonavur Fellowship and Maria Joseph, Head Master, Christuraja Hr., Sec., School, Palayamkottai helped us with logistics and we are grateful to them for their help.

Literature cited

Addline, EPD, J Vanitharani., S.Rajkumar, and R.T.Sundara. 2004. Government, law and temple bat management, pp-297-301. In: Muthuchelion,K (eds). Biodiversity Resources Management and Sustainable use. Centre for biodiversity and forest studies, school of energy, environment and natural resources, Madurai Kamaraj University, Madurai.

Audet, D., D.Krull, G. Marimuthu, S. Sumithran, and J. B. Singh. 1991. Foraging Behaviour of the Indian False Vampire Bat, *Megaderma lyra* (Chiroptera: Megadermatidae). Biotropica, 23: 63-67.

Barragan, F., C. Lorenzo, A.Moron, M.A. Briones-Salas, and S.Lopez. 2010. Bat and rodent diversity in a fragmented landscape on the Isthmus of Tehuantepec, Oaxaca, Mexico. *Tropical Conservation Science*, *3*(1), pp.1-16.

Bates, P.J.J, and D.L.Harrison. 1997. Bats of the Indian Subcontinent. Harrison Zoological Museum Press, England.

Bolker,B.M, H.Skaug, A. Magnusson, A.Nielsen 2012. Getting started with the glmmADMB package. http://admb-project.org.

Bolker, B.M, M. E. Brooks, C. J. Clark, S. W. Geange, J. R. Poulsen, M. Henry, H. Stevens, J.S S. White. 2009.Generalized linear mixed models: a practical guide for ecology and evolution, Trends in Ecology & Evolution, Volume 24, Issue 3,Pages 127-135, https://doi.org/10.1016/j.tree.2008.10.008.

Chandrasekaran, M. K. 2005. Time in the living world. Universities Press, Hyderabad, 197 pp.

Carpenter,E.R Gomez,D.L.Waldein and R.E.Sherwin. 2014. Photographic estimation of roosting density of Geoffroys Rousette Fruit Bat *Rousettus amplexi caudatus* (Chiroptera: Pteropodidae) at Monfort Bat Cave, Philippines. Journal of Threatened Taxa*, 6*: 5838-5844.

Cauglin,T, T.Ganesh and M.Lowman. 2012. Sacred fig trees promote frugivore visitation and tree seedling abundance in South India. Current science.102:918-922

Clem, P. D. 1993. Foraging patterns and the use of temporary roosts in female evening bats, *Nycticeius humeralis*, at an Indian maternity colony. Proceedings of the Indian Academy of Science, 102:201- 206.

Deshpande, K. 2012. Assessing diversity and distribution of bats in relation to land-use and anthropogenic threats in the southern Western Ghats, India. *Final Report Submitted to the Rufford Small Grants for Nature Conservation, 30pp*.

Estrada, A., and R. Coates- Estrada. 2002. Bats in continuous forest, forest fragments and in an agricultural mosaic habitat-island at Los Tuxtlas, Mexico. *Biological Conservation*, *103*(2), pp.237-245

Fischer, J., J. Stott, B.S. Law, M.D. Adams, and R.I. Forrester. 2009. Designing effective habitat studies: quantifying multiple sources of variability in bat activity. *Acta Chiropterologica*, *11*(1), pp.127-137.

Ford, W. M., J.M. Menzel, M.A. Menzel, J.W. Edwards, and J.C. Kilgo. 2006. Presence and absence of bats across habitat scales in the upper coastal plain of South Carolina. Journal of Wildlife Management, *70*: 1200-1209.

Furey, N.M. & P.A. Racey. 2016. Conservation ecology of cave bats. In *Bats in the Anthropocene: Conservation of Bats in a Changing World*. C. Voigt & T. Kingston, Eds.: 463–500. Springer International Publishing)

Gaisler. J. 1979. Ecology of Bats. In Ecology of Small Mammals (D M Stoddard.ed.) *Chapman and Hall,* London, Pp 2B1-342. *386* pp.

Ganesh, T., A.Saravanan and M.Mathivanan. 2020. Let's Protect the Bats Where They Live – Including in Our Temples (https://science.thewire.in/environment/bats-covid-19-temples-south-india/)

Geluso, K.N., Geluso, K., 2012. Effects of environmental factors on capture rates of
579 insectivorous bats, 1971–2005. J. Mammal. 93, 161–169. doi:10.1644/11-MAMM-A-107.1

Harvey, C.A., A. Medina, D.M. Sanchez, S. Vilchez, B. Hernandez, J.C. Saenz, J.M. Maes, F. Casanoves, and F.L. Sinclair. 2006. Patterns of animal diversity in different forms of tree cover in agricultural landscapes. *Ecological applications*, *16*(5), pp.1986-1999.

Ho, Y.Y., and L.L. Lee. 2003. Roost selection by Formosan leaf-nosed bats (Hipposideros armiger terasensis). *Zoological Science*, *20*(8), pp.1017-1024.

Humphrey, S.R. 1975. Nursery roosts and community diversity of Nearctic bats. *Journal of mammalogy*, *56*(2), pp.321-346

HR&CE 2020 https://tnhrce.gov.in/hrcehome/temple_list.php

Inger, R. F., H. B. Shaffer, M. Koshy, and R. Bakde. 1987. Ecological structure of a herpetological assemblage in South India. Amphibia-Reptilia, 8: 189–202.

Jenkins, E. V., T. Laine, S. E. Morgan, K. R. Cole, and J. R. Speakman. 1998. Roost selection in the pipistrelle bat, *Pipistrellus pipistrellus* (Chiroptera: Vespertilionidae), in northeast Scotland. Animal Behaviour, 56:909-917

Jones, G., D.S. Jacobs, T.H. Kunz, M.R. Willig, and P.A. Recay. 2009. Carpe noctem: the importance of bats as bioindicators. *Endangered species research*, *8*(1-2), pp.93-115.

Karra,K, C.Kontgis, Z. Statman-Weil, J.Mazzariello, M. Mathis, S. Brumby 2021. Global land use/land cover with Sentinel-2 and deep learning." IGARSS 2021-2021 IEEE International Geoscience and Remote Sensing Symposium. IEEE, 2021.

Kerth, G., M. Wagner, and B. Konig. 2001. Roosting together, foraging apart: information transfer about food is unlikely to explain sociality in female Bechstein's bats (Myotis bechsteinii). *Behavioral Ecology and Sociobiology*, *50*(3), pp.283-291.

Korad, V., K. Yardi, and R. Raut. 2007. Diversity and distribution of bats in the Western Ghats of India. *Zoos' Print Journal*, *22*(7), pp.2752-2758.

Kunz, T. H., E. Braun, De Torrez, D. Bauer, T. Lobova, and T.H. Fleming. 2011. Ecosystem services provided by bats. Annals of the New York Academy of Sciences, 1223: 1-38.

Leippert, D., W. Goymann, H. Hofer, G. Marimuthu, and J. Balasingh. 2000. Roost-mate communication in adult Indian false vampire bats (*Megaderma lyra*): an indication of individuality in temporal and spectral pattern. Animal Cognition, 3: 99-106.

Lewis, S.E. 1995. Roost fidelity of bats: a review. Journal of Mammal, 76, 481–496.

Lianne C. Ball 2002.A Strategy for Describing and Monitoring Bat Habitat. The Journal of Wildlife Management, Vol. 66(4): 1148-1153.

Mazerolle, MJ (2011). AlCcmodavg: Model selection and multimodel inference based on (Q) AIC (c). *R package version*, *1*.

McNab, B.K. 1982. Evolutionary alternatives in the physiological ecology of bats. In *Ecology of bats* (pp. 151-200). Springer, Boston, MA.

Medellin, R.A., M. Equihua, and M.A. Amin. 2000. Bat diversity and abundance as indicators of disturbance in Neotropical rainforests. *Conservation biology, 14*(6), pp.1666-1675.

Medina, A., C.A. Harvey, D.S. Merlo, S. Vilchez, and B. Hernandez. 2007. Bat diversity and movement in an agricultural landscape in Matiguás, Nicaragua. *Biotropica*, *39*(1), pp.120-128

Meyer, C.F., M.J. Struebig, and M.R. Willig. 2016. Responses of tropical bats to habitat fragmentation, logging, and deforestation. In *Bats in the Anthropocene: Conservation of bats in a changing world* (pp. 63-103). Springer, Cham.

Moreno, C.E, and G. Halffter. 2001. On the measure of sampling effort used in species accumulation curves. *Journal of Applied Ecology*, pp.487-490.

Mahandran, V.,C. M. Murugan and P. T. Nathan, 2015. An unusual roosting behaviour of Schneider's leaf-nosed bat, *Hipposideros speoris* at a cave-temple roost in Tamil Nadu. *Current Science*,109(5), pp 858:859.

Nikil.K.2020. Assessment of seasonal changes in total open water extent of tanks of Tirunelveli and Thoothukudi districts of Tamil Nadu using geomatics. Masters dissertation thesis, Pondicherry Central University, India. pp 44.

Payton, M. E., M. H. Greenstone, and N. Schenker. 2003. Overlapping confidence intervals or standard error intervals: what do they mean in terms of statistical significance? Journal of Insect Science 3:1–6.

Rainho, A., Palmeirim, J.M., 2011. The importance of distance to resources in the spatial 701 modelling of bat foraging habitat. PLoS One 6, e19227. doi:10.1371/journal.pone.0019227

Rajasegarn, P., N. Shazali, and F.A.A. Khan. 2018. Microclimate and Physiological Effects in the Roosts of Cave Dwelling Bats: Implications in Roost Selection and Conservation in Sarawak, Malaysian Borneo. *Zoological science*, *35*(6), pp.521-527.

Razgour, O., Hanmer, J. & Jones, G. 2011. Using multi-scale modeling to predict habitat suitability for species of conservation concern: the grey log-eared bat. Biological Conservation, 144, 2922–2930.

Samrat.A, M. S. Devy & T. Ganesh. 2021. Delineating fragmented grassland patches in the tropical region using multi-seasonal synthetic aperture radar (SAR) and optical satellite images, International Journal of Remote Sensing, 42:10, 3938-3954, DOI: 10.1080/01431161.2021.1881181, IF:2.976

Selvarathinam, T, and G. Marimuthu. 2013. Roost selection and fidelity of leaf-nosed bat, *Hipposideros speoris*. International Journal Current Trends in Research, 2: 62-65

Sophia,E. 2010.Foraging behaviour of the microchiropteran bat, *Hipposideros ater* on chosen insect pests. Journal of Biopesticides 3(1 Special Issue) 068 – 073.

Sophia,E, and J.Vanitharani.2006. *Hipposideros ater* in the ecosystem of Tirunelveli, Tamilnadu. Journal of Theoretical and Experimental Biology. Vol. 3 Pp: 27-34 ISSN: 0972-9720.

Stebbings, R.E. 1995. Why should bats be protected? A challenge for conservation. *Biological Journal of the Linnean Society*, *56*(suppl_1), pp.103-118.

Suksai, P., and S. Bumrungsri. 2019. Water bodies are a critical foraging habitat for insectivorous bats in tropical agricultural landscapes of central Thailand. *Songklanakarin Journal of Science and Technology*.

Swamidoss, P.D., M.R. Sudhakaran, and P. Parvathiraj. 2012. Habitat preference of microchiropteran bats in three districts of Tamilnadu, South India. *International Research Journal of Biological Sciences*, *1*(5), pp.24-30.

Thet.T and K. M. Mya 2015. Harvesting the guano of insectivorous bats: is it sustainable? Journal of Threatened Taxa 7(6): 7296–7297.

Treitler, J.T., Heim, O., Tschapka, M. and Jung, K. (2016), The effect of local land use and loss of forests on bats and nocturnal insects. Ecol Evol, 6: 4289-4297. https://doi.org/10.1002/ece3.2160).

Vanitharani, J., 2004. The Emerging Trends in the Bio-Diversity of Bats in Tamil Nadu. *Mapana Journal of Sciences*, *2*(2), pp.14-27.

Voigt, C.C., and T. Kingston. 2016. *Bats in the Anthropocene: conservation of bats in a changing world* (p. 606). Springer Nature.

Vonhof, M.R., and Barclay. 1996. Roost-site selection and roosting ecology of forest-dwelling bats in southern British Columbia. Canadian Journal of Zoology, 74: 1797-1805.

Williams-Guillen, K., E. Olimpi, B. Mass, P.J. Taylor, and R. Arlettaz. 2016. Bats in the anthropogenic matrix: challenges and opportunities for the conservation of Chiroptera and their ecosystem services in agricultural landscapes. In *Bats in the Anthropocene: Conservation of bats in a changing world* (pp. 151-186). Springer, Cham.

Wanger, T.C., K. Darras, S. Bumrungsri, T. Tscharntke & A.M. Klein (2014). Bat pest control contributes to food security in Thailand.Biological Conservation 171: 220–223; http://dx.doi.org/10.1016/j.biocon.2014.01.030

Figure 1

Click here to access/download;Figure;Figure 1.jpg

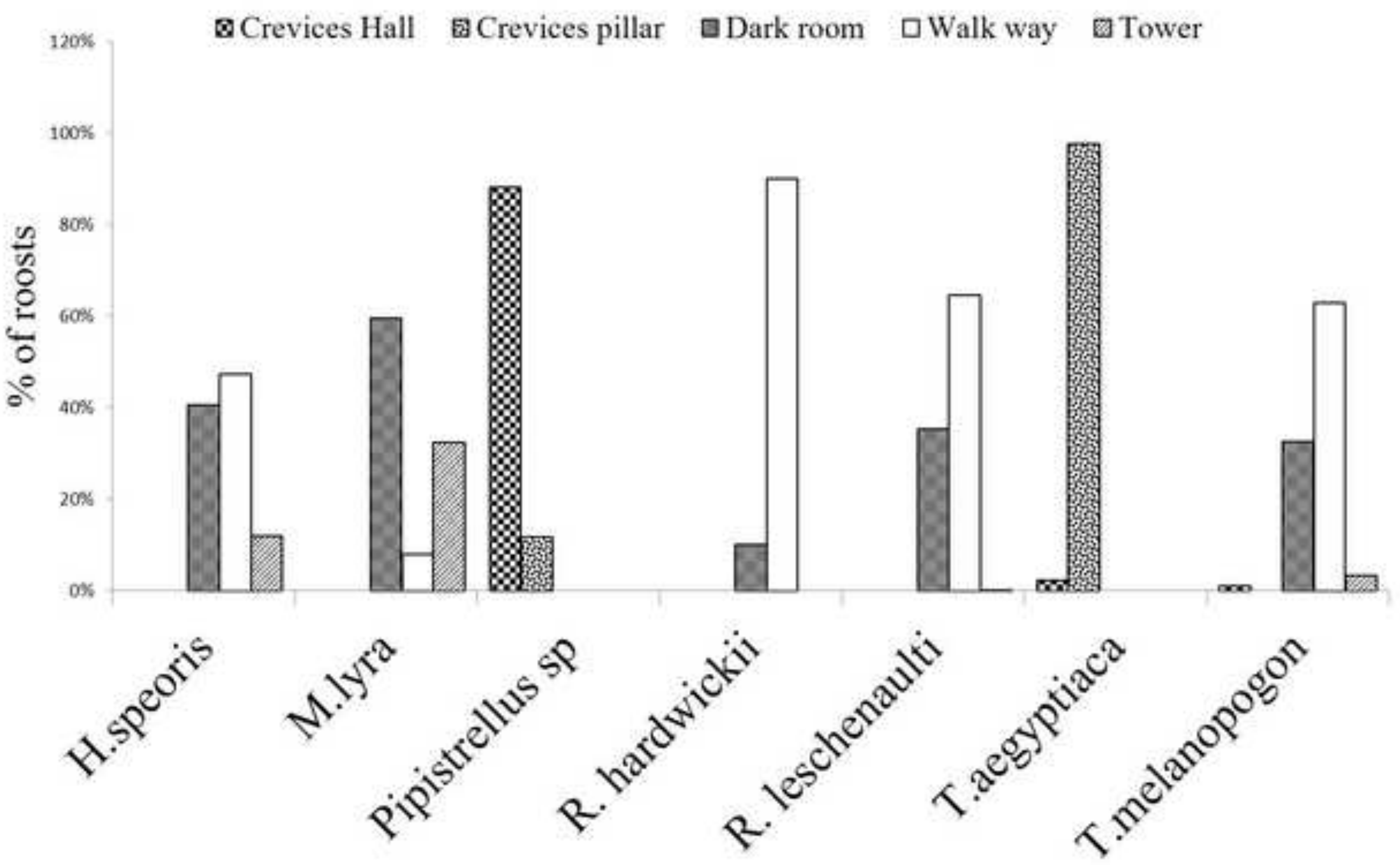

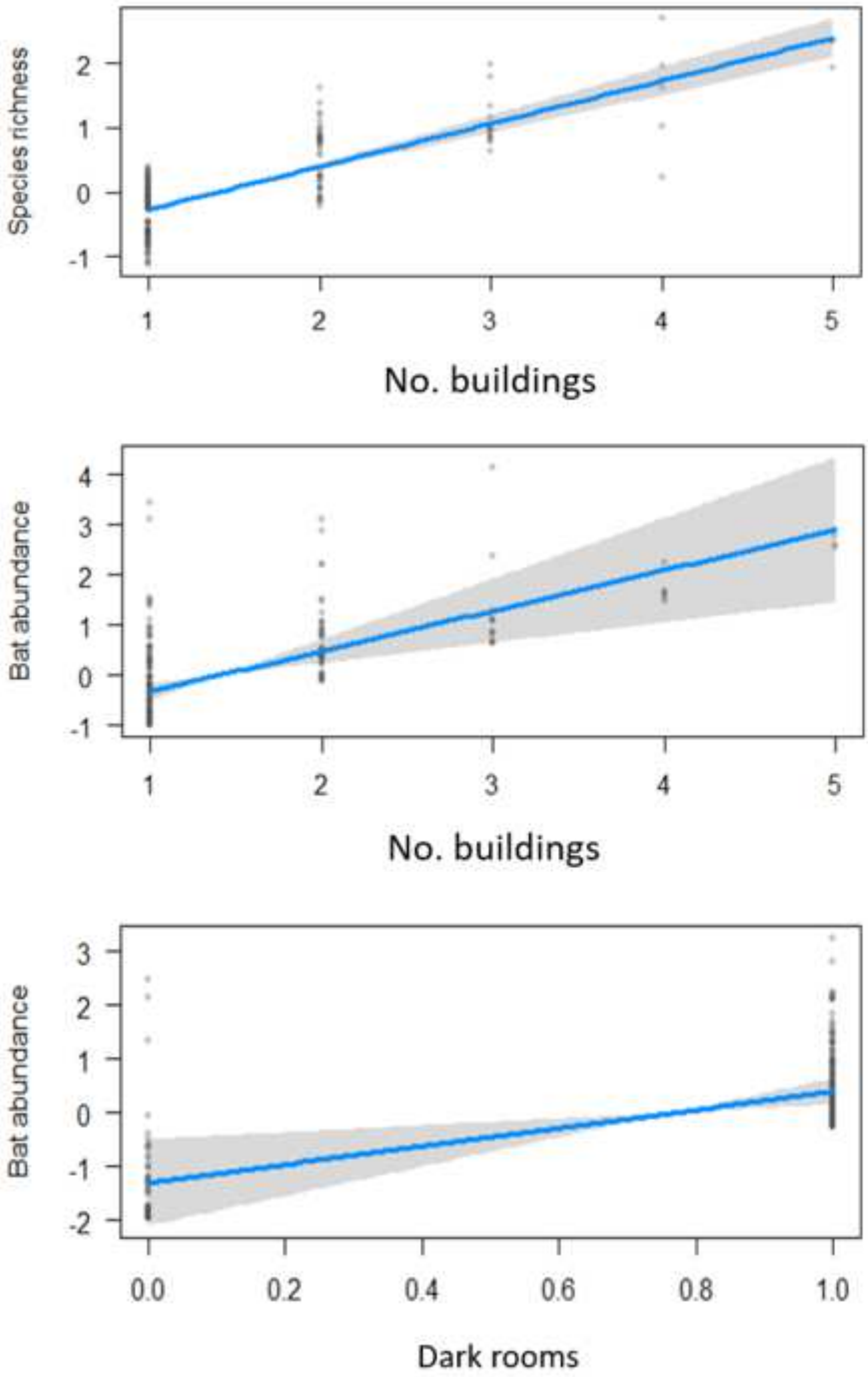

Species richness
2
1
0
-1
1
2
3
4
5
No. buildings
Bat abundance
4
3
2
1
0
-1
1
2
3
4
5
No. buildings
Bat abundance
3
2
1
0
-1
-2
0.0
0.2
0.4
0.6
0.8
1.0
Dark rooms

Figure 3

Click here to access/download;Figure;Figure 3.tif

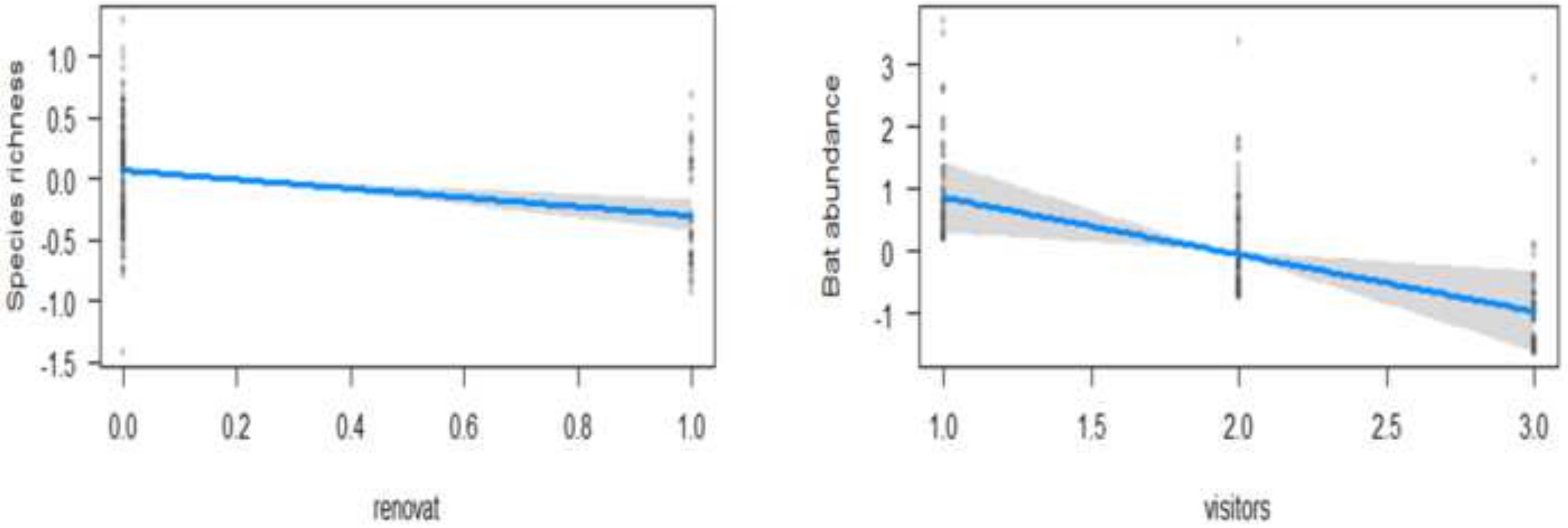

Figure 4

Click here to access/download;Figure;Figure 4.jpeg

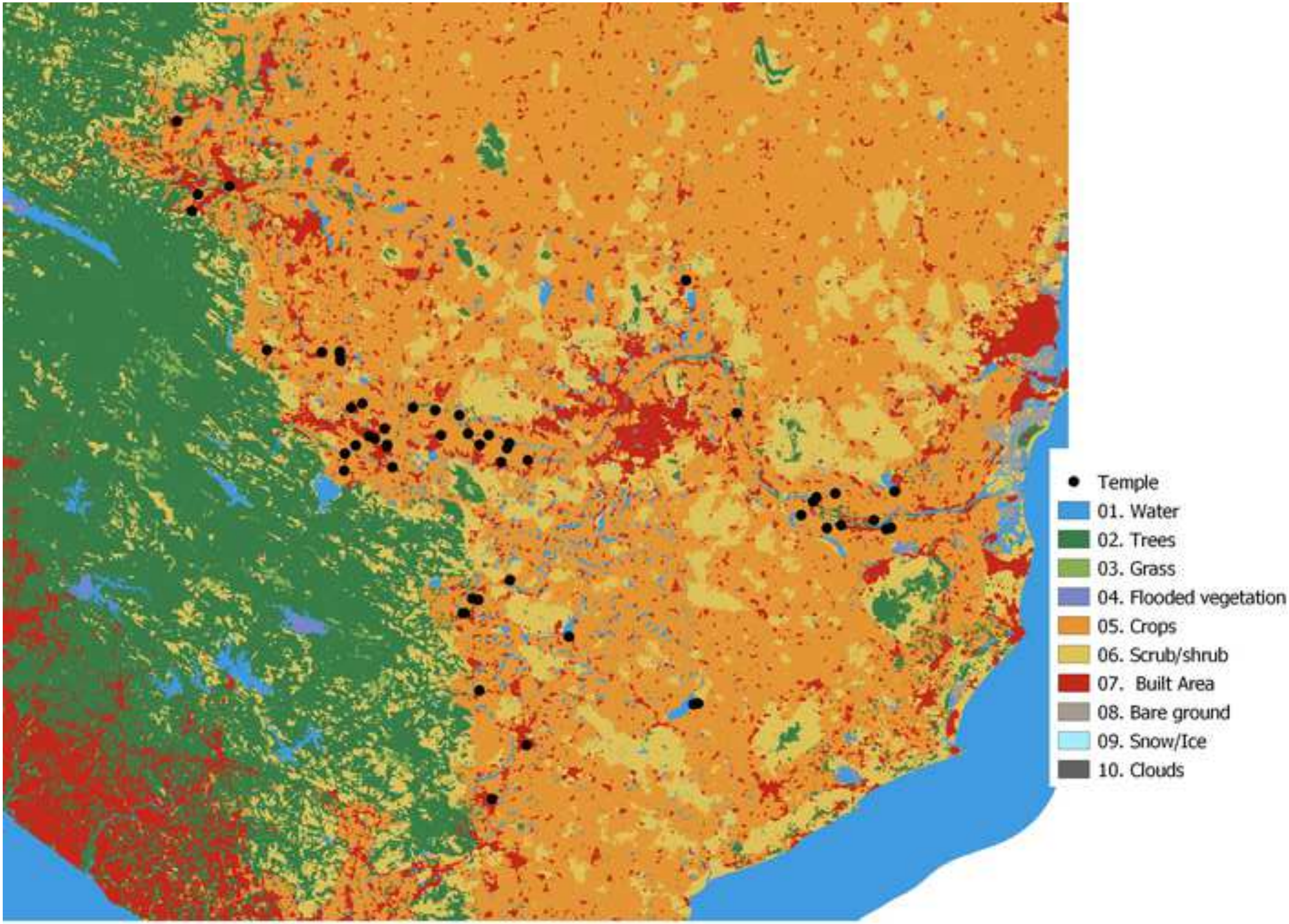

Figure 5 Click here to access/download;Figure;Figure 5.jpg

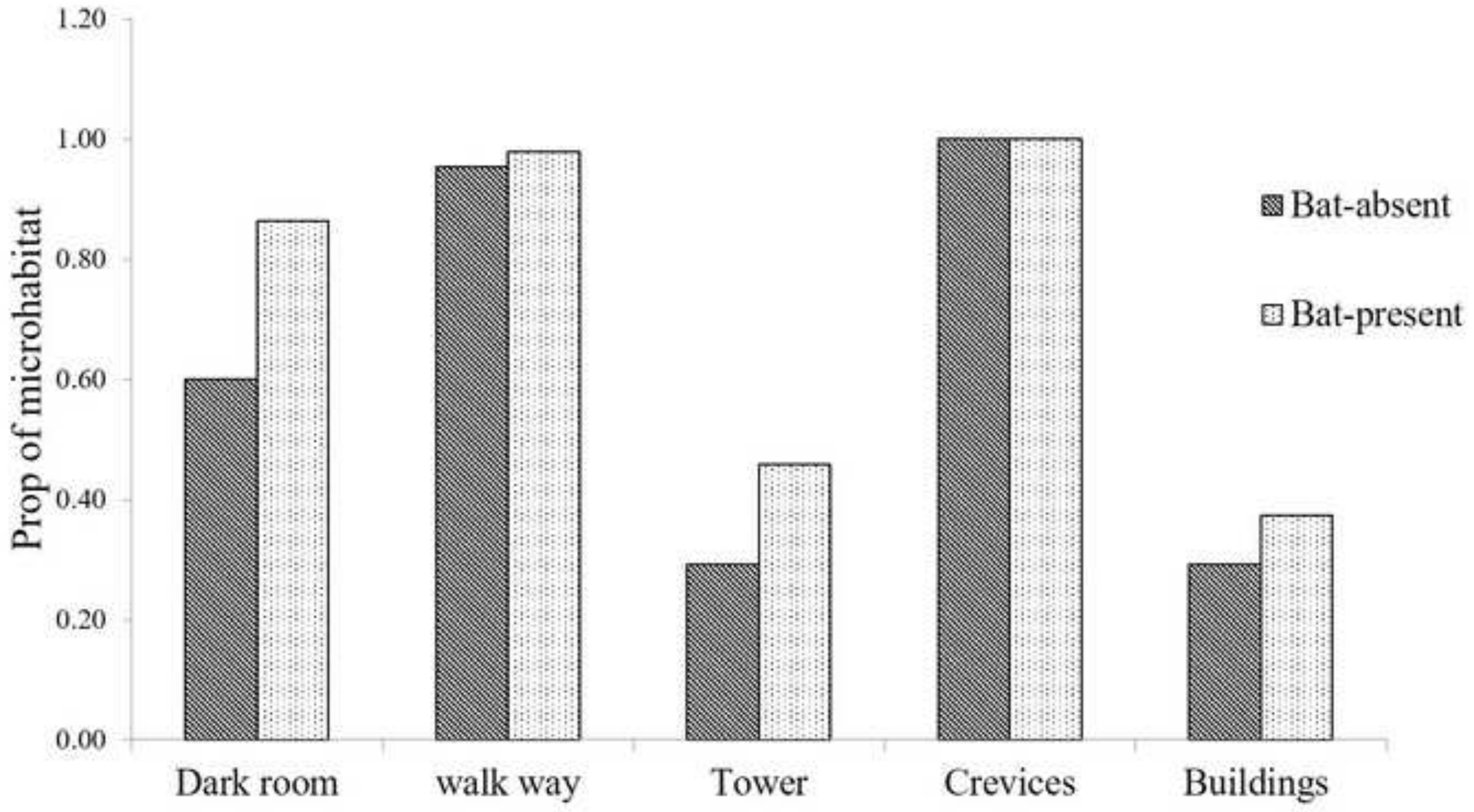

Figure 6

Click here to access/download;Figure;Figure 6.tif

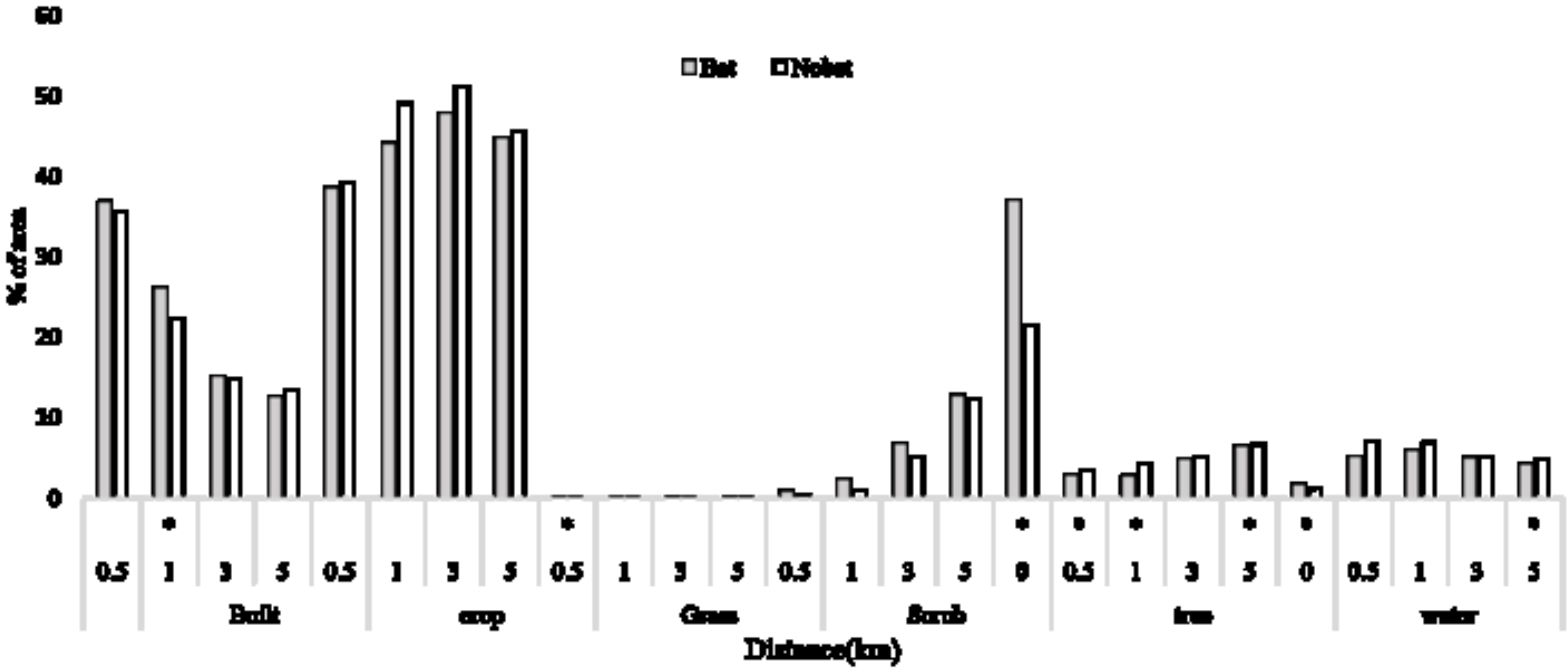

Click here to access/download
Supporting Information
Sup1 Table.docx

Revised Manuscript with Track Changes

Manuscript

Click here to access/download;Manuscript;Bats in ancient temples of South India.docx

Temples the last abode for bats in a homogeneous agriculture landscapematrix: Importance of microhabitat availability, disturbance and land use factors for bat conservation.

Authors: T.Ganesh, A.Saravanan and M.Mathivanan

Ashoka Trust for Research in Ecology and the Environment (ATREE),
Royal Enclave, Sriramapura, Jakkur Post, Bangalore 560064,
Karnataka state, India

// Abstract, body paragraphs
Abstract~~BSTRACT~~

Anthropogenic structures such as temples in south Asia are widely used by ~~mostly~~ cave-dwelling bats as roosting and nursery sites. These roosts are constantly under threat as t~~T~~emples are being renovated and bats ~~are~~ removed from such places ~~on a regular basis~~regularly even more so after the COVID19 pandemic. ~~However,~~ No ~~there is no~~ detailed understanding exists on~~f~~ what makes temples favorable for bats and what factors are important for their persistence~~conservation~~. Here we relate temple microhabitat characteristics and land use around ancient temples (>400 years) to bat species richness and abundance in the Tamiraparani river basin of south India. Temples were selected for sampling along the basin based on logistics and permission to access them. Bats were counted at the roost in the mornings, and late afternoons, from inside the temples. Temple characteristics such as dark rooms, corridors, crevices and presence of tower were recorded after the count along with disturbances to the roost. ~~was made~~ We also recorded land use such as crop, trees ~~plantations~~, scrub, grassland, urban areas and water availability within 5 ~~3~~5km radius of the temple based on ESA landuse classifications. We used GLMM to relate the counts in temples with microhabitat and land use. We sampled 59 temples repeatedly across 5 years which yielded a sample of 256 ~~temples~~survey events.

The total number of bats counted was 20,211 of which *Hipposideros speoris* was the most common (9,715) followed by *Rousettus leschenaulti* (5,306), *Taphozous melanopogon* (3,196), *Megaderma lyra* (1,497) *Tadarida aegyptiaca* (303), *Pipistrellus sp* (144) and *Rhinopoma hardwickii* (50). About 39% of the abundance was accounted by dark rooms while 51% by walkways ~~halls(corridors)~~ around the ~~diety~~deity room. Species richness and total abundance ~~was~~ were related to the number of building in the temple and the availability of dark rooms. Land use elements only had a weak effect but scrub and grassland even though were few are~~open natural habitats were~~wereas, important for bats~~most species~~. ~~The bats were also seasonal with the monsoons having the lowest number of bats which we atribute~~attribute ~~to the breeding behaviour~~behavior ~~of the species.~~ We conclude that retaining undisturbed dark rooms with small exits in temples and other dimly lit areas and having natural areas around temples are critical for bats conservation in the area.

Key words: Bats; microhabitat; Hindu temples; India; landscape,

Formatted: Font: 18 pt

Formatted: Font: 20 pt

Formatted: Font color: Text 1

Formatted: Font color: Text 1

Formatted: Font color: Text 1

Formatted: Font color: Text 1

Formatted: Font color: Text 1

Formatted: Font color: Text 1

Formatted: Font color: Text 1

Formatted: Font color: Text 1

Formatted: Font color: Text 1

Formatted: Font color: Text 1

Formatted: Font color: Text 1

Formatted: Font color: Text 1

Formatted: Right: 0.07", Space Before: 0.05 pt

Formatted: Font color: Text 1, Not Highlight

Formatted: Font color: Text 1

Formatted: Font color: Text 1, Not Highlight

Formatted: Font color: Text 1

Formatted: Font color: Text 1, Not Highlight

Formatted: Font color: Text 1

Formatted: Font color: Red

Introduction

Many studies suggest that human-modified landscapes with a heterogeneous mosaic of different land-uses can preserve species-rich bat assemblages (Medellín et al. 2000; Moreno and Halffter 2001; Estrada and Coates-Estrada 2002; Harvey et al. 2006; Medina et al. 2007; Barragan et al. 2010; Mendenhall et al. 2014). This is possible with the concurrent availability of microhabitats that bats use as roosting sites ranging from caves, crevices, tree cavities, foliage, and several types of anthropogenic structures including road bridges and buildings (Voigt and Kingston 2016, Meierhofer et al. 2019)). These microhabitatThese microhabitats being important features of a bat's environment, the selection of specific roost sites can strongly impact the survival and fitness of bats (Vonhof and Barclay, 1996, Humphrey, 1975; Stebbings, 1995). However, emerging challenges such as changing agricultural practices and climate change pose major threats to bat assemblages and populations in human-dominated landscapes (Jones et al. 2009, Frick et al 2019) and could negatively impact human populations by altering the ecosystem services that bats provide (Kunz et al. 2011, Williams-Guillén et al 2016). Moreover, bats that roost in anthropogenic structures are considered vulnerable to disturbance since just like cave roosting species they often form large, concentrated aggregations attracting attention (Furey, et al. 2016).

Most studies on the use of anthropogenic structures by bats have come from urban areas and under temperate conditions (Kunz et al., 2011; Jenkins et al., 1998; Clem, 1993, Fagan et al 2017,Meierhofer et al. 2019). While it is accepted that bats have been poorly studied in the Paleotropics (Meyer et al 2016), the selection of microhabitats and landscape-level influences on their population remains poorly known (McNab, 1982; Ho and Lee, 2003, Kerth et al., 2001). Most studies on bat–habitat relationships are done at fixed spatial scales (Rainho & Palmeirim 2011) but recent studiesy haves shown the -importance of scale for bats (Razgour, Hanmer & Jones (2011), Bellamy et al 2013. In tropical South Asia, several species of both insectivorous and frugivorous bats use temples as roosting sites (Bates and Harrison, 1997, Korad 2007). In south India numerous very old (>400y) temples dot the landscape and form important bat habitats (Swamidoss et al. 2012). These temples are made of granite stones Walls, pillars and roof of the temples are made of granite stones and t. The entrance of the temple has towers (*Gopuram*) usually made of bricks. which provide habitats for bats as they are undisturbed and removed from the buzz of

Formatted: Font: 18 pt

Formatted: Font color: Text 1

Formatted: Font: Times New Roman, 12 pt, Font color: Text 1

Formatted: Font color: Text 1

Formatted: Right: 0.09", Space Before: 0.05 pt

Formatted: Font: Times New Roman, 12 pt, Font color: Text 1

Formatted: Font color: Text 1

Formatted: Font: Times New Roman, 12 pt, Font color: Text 1

Formatted: Font color: Text 1

Formatted: Font: 12 pt, Font color: Text 1

Formatted: Font color: Text 1

Formatted: Space Before: 0 pt, Line spacing: 1.5 lines

Formatted: Font: Times New Roman, 12 pt, Font color: Text 1

Formatted: Font color: Text 1

Formatted: Font: Times New Roman, 12 pt, Font color: Text 1

Formatted: Font color: Text 1

Formatted: Font: Times New Roman, 12 pt, Font color: Text 1

Formatted: Font color: Text 1

Formatted: Font: Times New Roman, 12 pt, Font color: Text 1

Formatted: Font color: Text 1

Formatted: Font: Times New Roman, 12 pt, Font color: Text 1

Formatted: Font color: Text 1

Formatted: Font: Times New Roman, 12 pt, Font color: Text 1

Formatted: Font color: Text 1

temple activities. The temperature inside temples are usually warm and are not airconditioned and the temperature inside iswas stable and is usually warm (32^0 - 33.66^0 C), personal obs T.Ganesh) and fairly constant across the day (personal obs T.Ganesh). -Bats enter and leave the roosttemple through numerous ventsgaps that are left open for air to circulate inside the temple.

Bat use of anthropogenic structures such as temples also makes it difficult to balance bat conservation with human-bat conflicts. The templesse are situated in a human-dominated agricultural matrix where intensive paddy and banana cultivation is done. Agriculture is considered a major threat to bats (Frick et al. 2019) and with irrigated agriculture spreading to dry areas in the region, threats to bat population can be expected to rise in the future along with a predicted increase in rains(Tamil Nadu State Action Plan for Climate Change) that is changing due to several economic reasons and climate change effects (Fischer et al. 2009, 2010; Jones et al. 2009).

Walls, pillars and roof of the temples are made of granite stones. The entrance of the temple has towers (*Gopuram*) which provide habitats for bats as they are undisturbed and removed from the buzz of temple activities. Bats enter and leave the temple through numerous gaps that are left open for air to circulate inside the temple. Bat use of anthropogenic structures such as temples also makes it difficult to balance bat conservation with human-bat conflicts. Bats that roost in caves are considered vulnerable to disturbance because they form large, concentrated aggregations (Furey, et al. 2016). However, studies in temperate regions have shown how features of bridges can help identify sites for conservation and thereby reduce bat-human conflict (Meierhofer et 2019) and such efforts are needed for conserving bats in tropical regions. Populations of bats in many temples have also dwindled (Vanitharani 2004, Mahandran et al.2015) and many are also directly killed during temple renovation (Addline et al 2004). The changing attitude of people towards bats has also led many temples to be made "bat-proof" by placing nets and power washing roost areas with chlorinated chemicals to prevent bats from colonizing the place. The recent COVID 19 pandemic has further made them

Formatted: Font color: Text 1, Superscript

Formatted: Font color: Text 1

Formatted: Space Before: 0 pt, Line spacing: 1.5 lines

Formatted: Right: 0", Space Before: 3.55 pt

Formatted: Font: Italic, Font color: Text 1

Formatted: Font color: Text 1

Formatted: Font: Times New Roman, 12 pt, Font color: Text 1

Formatted: Font color: Text 1

Formatted: Font: Times New Roman, 12 pt, Font color: Text 1

Formatted: Font color: Text 1

more vulnerable to disturbance and direct persecution. In India, only two ~~species~~ out of 120 species of bats comes under the strict Wildlife Protection Act 1972 while others even though are threatened under~~by~~ IUCN category, remain unprotected.

Management decisions for bats and buildings, including the timing of maintenance activities, restriction of human use, and bat deterrence or exclusion, require information on the roosting habits of temple ~~building~~ roosting bats.

~~However,~~ S~~s~~tudies in temperate regions have shown how features of bridges and buildings can help identify sites for conservation and thereby reduce bat-human conflict (Fagan et al 2017,Meierhofer et 2019) and such efforts are needed for conserving bats in tropical regions.

~~.~~ It's therefore essential to understand what attributes of temples are important for bats and how such information can help articulate bat conservation efforts in such temple landscapes.

More than 50% of the 35 bat species found in Tamil Nadu state dwell ~~There are xx~~ in caves and old buildings (Bates and Harrison 1997) ~~dwelling bat species found in the plains of south India and all of them are known to use anthropogenic structures~~. Except one *Rousettus leschenaultia*, a frugivore, all are~~wereare~~ ~~Majority of these bats are~~ insectivores~~ous~~ ~~and only xx is~~ ~~a frugivorous species using built structures~~. In the paddy agriculture dominated landscape of south India, ancient temples are often the only ~~an important~~ habitat for several ~~of species~~ bats species. They provide critical ecosystem services for agriculture ~~Bats play a major role not only in forest ecosystems but also~~ and in south Asia ~~in agricultural areas in South Asia and~~ can save up to US$1.2 million per year from insect depredation ~~in south Asia~~ (Wagner et al. 2014) and consume large quantities of mosquitoes thus providing human health benefits ~~. Similarly from a human health considerations, they and consume a high number of mosquitoes~~ (Sophia 2010) ~~that could be beneficial to human health. in the landscape~~. -
~~These bats are known to provide important ecosystem services ranging from pollination, seed dispersal and pest control(Sopia . of~~ Despite the numerous threats and the importance of bats in providing key ecosystem services in human-dominated landscapes (Wagne~~rt~~ et al. 2014, Thet and Mya 2015), there is no systematic study on the ecology of bats in temples while behavioural studies have been carried out on them ~~species that occupy temples~~ in South India (Audet et al. 1991, Leippert et al.2000).

Formatted: Font: Times New Roman, 12 pt, Font color: Text 1
Formatted: Font: Times New Roman, 12 pt, Font color: Text 1
Formatted: Line spacing: 1.5 lines
Formatted: Font color: Text 1
Formatted: Font color: Text 1
Formatted: Font: Times New Roman, 12 pt, Font color: Text 1
Formatted: Font color: Text 1
Formatted: Font: Times New Roman, 12 pt, Font color: Text 1
Formatted: Font color: Text 1
Formatted: Font color: Text 1, Highlight
Formatted: Font color: Text 1
Formatted: Font color: Text 1, Not Highlight
Formatted: Font color: Text 1

Moreover, with populations of bats dwindling no attempt to monitor bats in the temples or how important are temples in an agricultural matrix to bats have has not been investigated. We initiated a bat monitoring program in 2012 in temples of south India to understand why bats use temples and how the population of species is responding to disturbance and land land-use change. In this study, we identify temples used by bats for roosting in the rice paddy-dominated agricultural landscape in South India. a semi-arid region characterized by the winter monsoon (Oct to Dec) unlike rest of the subcontinent which experiences summer monsoon (June-Sep) and, with agriculture dominated by extensively irrigated rice paddy and banana agriculture active for 6-8 months a year. The rest of the time the land is fallow in most places. We record the species composition, rarity, and abundance of bats in temples and identify factors that promote or dissuade bat colonization such as microhabitat availability, disturbance in temples and, disturbance in temples and landscape features surrounding the temples. While much of the bat diversity in human-dominated landscapes can be attributed to a combination of habitat diversity and roost site availability in an area, in tropical monoculture tropical cropping areas with rice paddies and banana andwith low habitat heterogeneity, it would be interesting to compare the role of land use elements and availability of microhabitats on bat species richness and abundances. In this paper, we test threewothree major hypotheses 1. Given that the temple bats are mostly cave-dwelling species we expect larger Yinpterochiroptera occupying similar micro-habitat, show greater habitatniche overlap and similar niche breadth of habitat use, 12. Microhabitat overlap among species is high for large *Yinpterochiroptera* Yinchiropteran bats but but they in temples vary seasonally anad is and is related to their breeding traits.Bat abundance and species richness in temples are a function of temple features dependent ofn the diversity of mMicrohabitat availabileity inside the temple and but are and in dependent ofn surrounding habitats. 2. Response to Ddisturbance at the roost negatively affects species richness and abundance of bats, 2. Roost characteristics are more important than landscape features for bats to colonizeoccupy temples is variable across sSpecies and 33. Bat abundance and species richness in temples are a function of temple features and independent of surrounding habitats.In a homogeneous monoculture cropping, species occurrence and abundance are independent ofnf spatial habitat availability of habitats around roosts. overlap of Mmicrohabitat overlap among species use in temples vary seasonally and vary seasonally is related to their breeding traitsBat climate conditions inside the temples are very stable and any influence on bat abundances arecould be a functionreflection of food availability (habitat heterogeneity) or disturbance at roost (temple renovation) rather than availability of and therefore we expect bats to show seasonal changes in microhabitatniche alone in the temple and 3. Monsoons because of greater productivity (insect abundance) would increase

Formatted: Indent: Left: 0"

Commented [A1]: This wasn't really mentioned in the abstract

Formatted: Font color: Text 1

Formatted: Font color: Text 1

Formatted: Font: Italic, Font color: Text 1

Formatted: Font color: Text 1

enhance bat abundance in temples. overlap and breadth due to variability in landscape level changes in crop and insect availability. 23. We, therefore, would expect a greater abundance of bats when the landscape around is most productive possibly and during the monsoonsSince the structure of ancient temples are all similar, we do not expect any change in microhabitat availability in temples with and without bats, instead we expect bat abundances driven by the availability of landscape features, especially seasonal crops around the temples.

Materials and Mmethods

Formatted: Font color: Red

Formatted: Font: 18 pt, Font color: Text 1

Formatted: Font: 18 pt, Font color: Text 1

Formatted: Indent: Left: 0"

Study area

The study was carried out in Tirunelveli & Thoothukudi Districts of Tamil Nadu state in South India (**Fig 1**.). The region is semi-arid and experiences the north~~-~~east winter monsoons between Oct-Dec which bring~~s~~ in 70% of the annual rainfall. The south monsoon which ~~are~~is obstructed by the Western Ghats mountains brings in strong winds and light rain during June – Sep. The rest of the months are dry. Several ancient temples built during the Chola and Pandya dynasties 500 to 1000 years back exist in the districts along the perennial ~~river~~ Tamiraparani river and its tributaries. The river and the numerous interconnected village ponds are~~is~~ the~~a~~ lifelines for Paddy and Banana dominated agriculture supporting a ~~and athe~~ dense human population in the~~a~~ semi-arid landscape. The ancient temples ~~(>400 years)~~ are built of large granite stones and provide a habitat~~niche~~ for several bat species. There are over 4041 temples (HR&CE, 2020) in the study area with 2-3 villages having one ancient temple. The typical structure of the temples includes a tower that stands at the four entrances and one over the ~~diety~~deity, a~~. The~~ deity chamber ~~is usually~~ lit by an oil lamp and an electric bulb, ~~where only the priest can enter and devotees are not allowed, a.~~ ~~a~~A walk-way ~~corridor (hall)~~ surrounding the deity chamber ~~which is used by people~~ for walking and prayer by devotees, and several rooms including storerooms and a kitchen which are dark and rarely frequented by people. The ~~surveyed~~ temples are ~~situated in an agricultural matrix surrounded by rice paddies or bananas and~~ usually located towards the corner of a village or town.

Fig 1. Map of the study area. The filled dots represent~~s~~ temples with bats and empty ones without bats~~sampled~~.

Temple selection

We selected 59 temples from the ~~Tamrabarni~~Tamiraparani basin and ~~were visited by~~surveyed them with 2-4 personnel between 06:00-12:00 h and again between 16:00 to 17:00h to detect the presence of bats. The survey was done from May to Dec and the same temples were visited across the years 2012,2013,2014,2018,2019. ~~and~~for a total of 246 ~~temples were sampled.~~surveys

All species of both *Yinpterochiroptera* and *Yangochiroptera* bats found roosting inside the temples were recorded. The presence of bats in the temple was ~~recognized~~ identified by several signs such as droppings and smell, ~~by~~ interviewing local people, temple priests, and caretakers. Later d~~D~~ark corners, temple roof, abandoned & isolated rooms inside the temple~~,~~ and the

Formatted: Font color: Text 1

Formatted: Font color: Text 1

Formatted: Font color: Text 1

Formatted: Font: 12 pt, Font color: Text 1

Formatted: Font color: Text 1

Commented [A2]: Once in each season and each year? Why not year round?

Commented [A3R2]: Not possible to do year round for financial and logistic reasons

Commented [A4]: Was visual confirmation required for occupancy or was one of these signs? How were these signs used for abundance?

Commented [A5R4]: Yes visual confirmation was necessary to count bats after they we detected.

temple tower were all scanned for chiropteran presence with a red LED light. Once bat colonies were detected, we counted the number of individuals and in some cases, especially in closed towers, we took photographs to estimate them. ~~The survey was done from May to Dec and the same temples were visited across the years 2012,2013,2014,2018,2019. and a total of 246 temples were sampled.~~

Temple features

a. Ma.(microhabitat)

There arewere multiple locations inside temples where bats could have roosted: Closed rRooms which were usually closed structures with few small openings, with very low natural light and not frequently used by people arewere categorized as *Dark Rrooms*; stone pillars that supported the stone roof was considered a separate habitat as they provided a niche for few species that roost in the gaps created between the stone joints this was categorized as *crevices*; temple *towers* which were conical structures built over the entrance or the deity was considered as *towers* and wide walking space around the dietydeity room covered by a stone roof was termed as walk-ways*halls*. We also counted the number of independent buildings in the temple complex as bats could move between them during the day due to disturbance. In 2012 we measured temperature and humidity while sampling for bats and found no difference between temples and therefore not considered in the analysis. We could not set dataloggers for continuous monitoring because of objections by the temple authorities to placing any devices inside temples. Temple size was obtained from the visual observation;, those with deity rooms and walkways corridor considered as small, those with deity rooms, walkwayscorridor and tower considered as medium-sized, and those having deity rooms, multiple towers, and two walkwayscorridors (inside and outside) considered as large. We also recorded number of trees in the temple complex and open wells that are often used by bats. The basic structure of the temple remained the same across temple size with more walkways and towers included as they became big.

There were multiple locations inside temples where bats could roost: Closed rooms with few small openings, very low natural light and not frequently used by people were Dark Rooms; stone pillars that support the stone roof is a separate habitat as they provide a niche for few species that roost in the gaps created between the stone joints this was crevices; temple towers which were conical structures built over the entrance or the deity and wide walking space around the deity room covered by a stone roof are walkways. We also counted the number of independent buildings in the temple complex as bats could move between them during the day due to disturbance. In 2012 we measured temperature and humidity while sampling for bats and found no difference between temples and therefore not considered in the analysis. We could not set dataloggers for continuous monitoring because of objections by the temple authorities to placing any devices inside temples. Temple size *was* based on visual observation; those with deity rooms and walkways were considered as small, those with deity rooms, walkways and towers considered

Formatted: Font: 12 pt, Font color: Text 1

Formatted: Line spacing: 1.5 lines

Formatted: Font: 12 pt, Font color: Text 1

Formatted: Line spacing: 1.5 lines, Numbered + Level: 1 + Numbering Style: a, b, c, … + Start at: 1 + Alignment: Left + Aligned at: 0.07" + Indent at: 0.32"

Formatted: Line spacing: 1.5 lines

Formatted: Font color: Text 1

Formatted: Font: Italic, Font color: Text 1

Formatted: Font color: Text 1

Formatted: Font: Italic, Font color: Text 1

Formatted: Font color: Text 1

Formatted: Font: (Default) Times New Roman, 12 pt, Not Bold, Font color: Text 1

Formatted: Font: (Default) Times New Roman, 12 pt, Font color: Text 1

Formatted: Font: (Default) Times New Roman, 12 pt, Not Bold, Font color: Text 1

as medium-sized, and those having deity rooms, multiple towers, and two walkways (inside and outside) were considered as large. We also recorded the number of trees in the temple complex and open wells used by bats. The basic structure of the temple remained the same with more walkways and towers included as they became big.

b. b.Disturbance

Two factors that directly impact bats at the roost are structural impacts like renovation of the temple premises and human presence. Renovation of temples like power washing the walls, reconstructing parts of the building or any other maintenance work was on a scale from 0 to 1. Zero being no renovation activities in the temple and 1 when it was present. Information on visitors to the temple, obtained from temple authorities and scaled from 1 to 3. Three pertains to large crowds and 1 attracting few people.Ttwo factors that directly impact bats at the roost are structural impacts like renovation of the temple premises and human presence. Renovation of temples like power washing the walls, reconstructing parts of the building or any other maintenance work was graded on a scale from 0 to 1. Zero being no renovation activities in the temple and 1 when it was noticed. Human presence was accessed based on visitors to the temple obtained from temple authorities on a scale from 1 to 3. Three pertains to being a large crowds and 1 attracting being very few people.

Land--use features

Land-useHabitat features can be easily measured from satellite-based land--use classification however in this case we could not do that for each sampling period because as the region had of high levels of cloud cover during that timee sampling period from May to Dec, being the monsoon season. We, therefore, used the composite analysis available analysis of land cover available for 2015 (ESA landcover) and using high-resolution(10m) Esri data (10m) for 2020 (Karra et al.2021) (https://tiledimageservices.arcgis.com/P3ePLMYs2RVChkJx/arcgis/rest/services/Esri_2020_Land_Cover_V2/ImageServer) to identify major land uses in the region around temples. We could not obtain such information for each sampling year. The 2015 data was too coarse and we used the Esri 10m data The land uses were already are to categorized land uses into as water, scrub,- and grassland, bare ground, flooded area, crop, urban and tree tree-covered areas (Karra et al.2021). We combined scrub, bare ground and, grassland as grassland an open habitat and water and flooded area as water habitat after verifying the actual land-use on the groundbecause the area under each of these was very low and using Ggoogle Eearth. For instance, croplands

Formatted: Font color: Text 1

Formatted: Font: 12 pt, Font color: Text 1

Formatted: Space Before: 0 pt, Line spacing: 1.5 lines, Numbered + Level: 1 + Numbering Style: a, b, c, … + Start at: 1 + Alignment: Left + Aligned at: 0.07" + Indent at: 0.32"

Formatted: Font: (Default) Times New Roman, 12 pt, Font color: Text 1

Formatted: Font: 12 pt, Font color: Text 1

Formatted: Font: (Default) Times New Roman, 12 pt, Not Bold, Font color: Text 1

Formatted: Font: 12 pt, Not Bold, Font color: Text 1

Formatted: Space Before: 0 pt, Line spacing: 1.5 lines

Formatted: Font: 12 pt, Font color: Text 1

Formatted: Line spacing: 1.5 lines

Formatted: Font color: Text 1

Commented [A6]: Why only survey during monsoon season?

Commented [A7R6]: May and Sep are considered as summer but these times because of the south west monsoon in the mountains cloud cover is high over the plains. Sampling was also constrained by availability of interns and volunteers.

Formatted: Font color: Text 1

Formatted: Font color: Text 1

constituted about 90-98% of the land cover using 2015 land cover data but was 63% using Esri data. We therefore based all our analysis on the Esri land use categories relating to bat counts in the temple. Crop availability each season was obtained from Google Earth images when available and further substantiated by informal interviews with farmers around each temple. If crops were present we used the Esri calculated value of crop area in the analysis and if not we considered it as zerothat area under fallow grassland. The extent of other land uses is assumed has not to have changed much during the sampling period based on the data available with the district administration (Statistical handbook of Tirunelveli district 2009-2019).

Since the spatial scale is important for bat foraging (Bellamy and Scot 2013) we We, therefore, developed a novel approach by characterizing land use within a 5 km radius around each temple roost based on Ggoogle Eearth images when available and substantiated by informal interviews with farmers from around each temple. This was done for each year and month of sampling. Since we did not have information on how far the different bat species forage in the landscape we took used 5 km based on the study on the large bat *Megaderma lyra* (Audet et al 1991). categorized land use at 500m, 1km-,3km and 5km from the temple based on the foraging distance of bats from their roost (Audet et al 1991). We also recorded trees and water availability (open wells) which are often used by bats in the temple complex, for land-use analysis at 0km level. For the Ggoogle Eearth classification, we divided the 5km radius around the temple into 4 quadrants and recorded the presence or absence of seasonal crop type(rice paddy, banana), perennial vegetation(scrub and other natural vegetation), availability of water (ponds /rivers) and number of villages within the 5km distance. If a land use element wasis present we gave a value of 0.25 in each quadrant and calculated the frequency of each land use for the entire 5km radius area. If particular land use is found in all four quadrants it had

Formatted: Font color: Text 1, Strikethrough

a frequency 1 and 0.25 if only recorded once. This also gives an approximate extent of each land use around the roost.

Formatted: Indent: Left: 0"

Commented [A8]: Not sure this is a very effective method for land use calculation

Commented [A9R8]: We have changed our methods

Formatted: Font color: Text 1, Strikethrough

Formatted: Font color: Text 1

Formatted: Font color: Text 1, Strikethrough

Formatted: Font color: Text 1

Species identification and abundance

Bats were identified to species based on detailed photographs taken in the temple using the bat identification guide by Bates and Harrison (2001).

Bats were counted at the roost when they were least active (late morning and early evenings) using a flashlight with red filters when they were least active(late morning and early evenings). Where possible photographs were taken as soon as bat aggregations were noticed in a particular microhabitat inside the temple and later counted to estimate abundance (Carpenter, 2014). Bats were later counted in the picture. Several pictures were taken in quick succession to estimate and avoid bias in counting. Binoculars were used to see the species characteristics for identification. We could not do a roost exit count in the evening as it was not logistically possible to be in each temple at the right time for the count.

Data analysis

Formatted: Indent: Left: 0.1"

Formatted: Font: 12 pt, Font color: Text 1

Formatted: Normal, Indent: Left: 0.1", Right: 0"

We compared habitat niche breadth and overlap of species to see if there are any exclusive use of microhabitats and the level of overlap between species. Habitat nNiche breadths were compared using Levin's Iindex while nNiche overlap was calculated using Pianka's Niche Ooverlap Index (Inger et al., 1987). If more than one roost was found in a temple, we pooled roost counts and recorded total species richness for the temple. We constructed a linear model to explain the species richness and abundance of bats in the temples based on the microhabitat and landscape features. We based the variables used in our linear models on our observations made in 2012. We eliminated choose variables that had were strongly correlated (>0.70) were not correlated with other variables to reduce variable redundancy and retained ones were also that are biologically meaningful. We related the total species richness and abundance(counts) of bats at the temple with temple variables and landscape elements using mixed models. Since temples were sampled more than once and repeated measurements on the same temple are often correlated; we accounted for this by using random effects in the mixed models (Bolker et al., 2009; Bolker, 2015). We used lmer package in R for species richness as it was normally distributed, and s. Since the bat counts were over-dispersed for abundancesabundances, we used a negative binomial with glmmadmb package in R (Bolker et al 2012)https://glmmadmb.r-forge.r-project.org/glmmADMB.html). We considered temple ID and year as a random factor in the

Formatted: Font: (Default) Times New Roman, 12 pt, Font color: Text 1

Formatted: Font: 12 pt, Font color: Text 1

Formatted: Font: (Default) Times New Roman, 12 pt, Font color: Text 1

Formatted: Font: 12 pt, Font color: Text 1

Formatted: Font: Times New Roman, 12 pt, Font color: Text 1

Formatted: Font: 12 pt, Font color: Text 1

Formatted: Font: 12 pt, Font color: Text 1, Not Highlight

Formatted: Font: 12 pt, Font color: Text 1

Formatted: Font: Times New Roman, 12 pt, Font color: Text 1

Formatted: Font: 12 pt, Font color: Text 1

model and temple characteristics, disturbance and land use elements as fixed factors. We used the function *dredge* in MuMIn package(version 1.43.17) in R to obtain all possible combinations of models using the select variables. ~~If more than one roost was found in a temple, we pooled roost counts and recorded total species richness for the temple.~~ We fitted two models one for temple characteristics with~~and~~ disturbance and the other for land use elements at 5~~3~~ spatial scales; 0m,500m, 1km,3km and 5km. The models were ranked using Akaike's Information Criterion corrected for sample size and based on calculated Akaike weights. ~~The best model was selected~~

Formatted: Font: 12 pt, Italic, Font color: Text 1

Formatted: Font: 12 pt, Font color: Text 1

Formatted: Font color: Text 1

~~using.~~ Model averaging was done for closely related models following(Mazerolle 2020) and model selection criteria in MuMIn package(version 1.43.17) in R. We calculated model-averaged parameter estimates and unconditional standard errors to assess the relative importance of each variable and account for model uncertainty -(Burnham and Anderson 2002). We considered only parameters with 95% confidence intervals and not overlapping with~~including~~ 0 to be informative (Payton et al. 2003). We tested for differences in microhabitats and land-use -between species ~~We compared medians of microhabitat and land use variables~~ using Kruskal Wallis and Mann Whitney tests. ~~All~~ D~~d~~ata analysis and statistical tests were carried out using Microsoft Excel®, PAST® and R version 4.

~~This map was produced by a deep learning model trained using over 5 billion hand-labeled Sentinel-2 pixels, sampled from over 20,000 sites distributed across all major biomes of the world.~~

~~The underlying deep learning model uses 6 bands of Sentinel-2 surface reflectance data: visible blue, green, red, near infrared, and two shortwave infrared bands. To create the final map, the model is run on multiple dates of imagery throughout the year, and the outputs are composited into a final representative map of 2020.~~

~~Karra, Kontgis, et al. "Global land use/land cover with Sentinel-2 and deep learning." IGARSS 2021-2021 IEEE International Geoscience and Remote Sensing Symposium. IEEE, 2021.~~

Results

Species richness and ~~,~~ abundance~~, and seasonality~~

Forty-seven of the 59 temples surveyed housed bats and 51 of these had multiple roosts and only 8 had a single roost across years. The total number of roosts sampled was 351 across 246 ~~temples~~ surveys in 5 years. Seven species of bats were recorded of which six were Yinpterochiroptera and one *Rousettus leschenaulti* was a Yangochiroptera **(Table 1)**. Among the Yinpterochiroptera, *Hipposideros speoris* was the most frequently seen species found in 135 (38%) roosts sampled followed by *Taphozous melanopogon (58), Megaderma lyra (29) Tadarida aegyptiaca (26) Rousettus leschenaulti (22), Pipistrellus sp (9) and Rhinopoma*

Commented [A10]: Incomplete sentence

Formatted: Normal, Indent: Left: 0.1", Right: 0", Space Before: 0 pt

Formatted: Left: 0.93", Right: 0.97", Top: 1.04", Bottom: 0.19"

Formatted: Font: Times New Roman, 12 pt, Font color: Text 1

Formatted: Font: 12 pt, Font color: Text 1

Formatted: Font: Times New Roman, 12 pt, Font color: Text 1

Formatted: Font: 12 pt, Font color: Text 1

Formatted: Font: Times New Roman, 12 pt, Font color: Text 1

Formatted: Font: 12 pt, Font color: Text 1

Formatted: Font: Times New Roman, 12 pt, Font color: Text 1

Formatted: Font: 12 pt, Font color: Text 1

Formatted: Font color: Text 1

Formatted: Font color: Text 1, Highlight

Formatted: Normal, Indent: Left: 0.1", Line spacing: 1.5 lines

Formatted: Font: 12 pt, Font color: Text 1

Formatted: Font color: Text 1

Formatted: Line spacing: 1.5 lines

Formatted: Font: 12 pt, Font color: Text 1

Formatted: Font color: Text 1

Formatted: Font: 18 pt, Font color: Text 1

Formatted: Indent: Left: 0.07"

Commented [A11]: Minimum and maximum counts for each species may be helpful information here

Commented [A12R11]: Included below

Formatted: Font color: Text 1

Formatted: Font: Bold, Font color: Text 1

Formatted: Font color: Text 1

hardwickii (7). Forty-five % of the temples had only one species, 34% had two species, 15% had three species and 6% had four species of bats. In all 20,211 bats were counted of which *Hipposideros speoris(HS)* was the most common (97,15, Range 2-1150) across temples) followed by *Rousettus leschenaultia(RL)* (5,306, 1-2946), *Taphozous melanopogon* (TM) (3,196, 1-1165), *Megaderma lyra* (ML)(1,497, range 20-605) *Tadarida aegyptiaca (TE)* (303, 3-84), *Pipistrellus sp (PS)* (144) and *Rhinopoma hardwickii* (RH)(50). None of these bat species recorded is "considered threatened" as per the International Union for Conservation of Nature (IUCN) category.

Table 1. Species characteristics and IUCN status of bats recorded in temples. The breeding season in parenthesis isare based on the present studythe are observed periods in temples.

~~The most abundant *H.speioris* did not show variations across months except was high in the first summer(May June) when it was high while all other *Yinpterochiroptera* bats were both low in summer and in the monsoon(NEM)(Nov and Dec, **Fig 2**)). The overall abundance of *Yinpterochiroptera* bats, however, showed lower variations than the fruit bats *Rousettus leschenaultia* which had a sharp peak in the pre-monsoon seasons. Two other species *Pipistrellus sp* and *Rhinopoma hardwickii* were not analyzed because of fewer samples. The percentage of temples with bats drops to 34% in the NEM from 88% during summer(**Fig 3**). Contrary to the hypothesis proposed, In general monsoon months show lower bat abundance and bat occupancy than other seasonsas we had hypothesized.~~

Commented [A13]: This is confusing

Commented [A14R13]: modified

~~**Fig 2. Mean abundance(x±se) of bats across seasons in temples. NEM: Northeast monsoon(Nov-Dec), SUM1 (May-June), SWM(July-Aug) and SUM2(Sep-Oct).**~~

~~**Fig 3. Percentage of temples occupied by bats across months.**~~

Bats and Roost characteristics

We distinguished four separate microhabitats inside the temple that were used for roosting by the seven species (**Fig 24**). About 39% of the total abundance was ~~accounted by~~ in dark rooms while 51% ~~by~~ of bats were in walk-ways ~~halls and (corridors)~~ around the ~~diety~~deity room which are~~which were~~was the main microhabitats for 5 out of 7 species. *Tadarida* ~~*T.*~~ *aegyptiaca, T. melanopogon* and *Pipistrellus sp* ~~while c~~Crevices ~~in pillars were~~ preferred pillars. ~~by three *T. aegyptiaca, T. melanopogon* and *Pipistrellus sp*~~ species.

Fig 2. Percentage of microhabitats used by the different species of bats for roosting.

~~**Table 2. Species characteristics and IUCN status of bats recorded in temples. The breeding season in parenthesis are observed periods in temples.**~~

In terms of species occupancy, *H. speoris* occupied~~was seen observed in~~ three out of four microhabitats, but most commonly ~~in~~ dark rooms;~~.,~~ ~~and h~~walkways ~~H~~~~alls(corridors)~~ were used only when darkrooms were not available or disturbed. L~~, l~~ikewise *M. lyra* and *T. melanopogon*, *R.*~~*hinopoma*~~ *hardwickii* were found in two microhabitats (walkways~~hall~~ and crevices) while *T. aegyptiaca* was found only between pillars(crevices), and *Pipistrellus sp* found only in roof crevices.~~,~~ The fruit bat *R. leschenaulti* preferred walkways~~halls~~.

~~Fig 4. Percentage of microhabitats used by the different species of bats for roosting.~~

~~Seasonal changes in species habitat overlap and niche breadth~~

~~The species overlap across temples varied from 63% to 0 but is generallyis very low except between *T. melanopogon and M.lyra*(63%). However, the overlap between species varies with seasons (**Table 21**). *H.speioris* has a strong overlap with *M.lyra* and *T.melanopogon* during summer (50%) and weak (<0.02%) in other seasons. Similarly, *M.lyra* has a strong overlap with *T.melanopogon* during the southwest monsoon(63%). In general, during the northeast monsoon when heavy rains occur in the region, there is very little overlap in the species across temples~~

Formatted: Font color: Text 1

Formatted: Font: 12 pt, Font color: Text 1

Formatted: Font color: Text 1

Commented [A15]: List species here

Formatted: Font: Not Italic, Font color: Text 1

Formatted: Font color: Text 1

Formatted: Font color: Text 1

Formatted: Font color: Text 1

Formatted: Font: 12 pt, Bold, Font color: Text 1

Formatted: Font color: Text 1

Formatted: Font: Not Italic, Font color: Text 1

Formatted: Font color: Text 1

Formatted: Font: Not Italic, Font color: Text 1

Formatted: Font color: Text 1

Formatted: Font: Bold, Font color: Text 1

Formatted: Font color: Text 1

Formatted: Font: Bold, Font color: Text 1

Formatted: Font color: Text 1

while in the dry season *H.speoris* and *T. melanopogon* and *M.lyra* the three large Yinpterochiroptera bats, showed greater overlapped with each other (**Table 21**). The fruit bat *R.leschenaulti* has a negligible overlap with other species except during summer when it shares space with *T. aegyptiaca*. These results only partly support our hypothesis on habitat overlap niche hypothesis of overlap among large *Yinpterochiroptera* bats and support our hypothesis of variations across seasons.

Formatted: Font: Italic, Font color: Text 1

Formatted: Font color: Text 1

Commented [A16]: This could use reference to some of the output values

Commented [A17R16]: incorporated

Formatted: Font color: Text 1

Formatted: Font color: Text 1

Formatted: Font color: Text 1, Strikethrough

Formatted: Font color: Text 1

Similarly, niche breadth (across microhabitat) also varied with seasons and northeast

monsoon showing a contraction shorter niche breadth for the large bats than in other seasons. Niche breadth was also highest for the most abundant *H. speoris* while others had similar niche breadth (**Table 2**). These results only partly support our niche hypothesis of overlap among large Yinpterochiroptera bats and support our hypothesis of variations across seasons.

Table 21: Species overlap(Pianka's overlap index) across seasons in temples of south India. NEM: Northeast monsoon, SWM: Southwest monsoon and dry season include SUMM1 and SUMM2.

Table 2. Niche breadth of bat species in temples. NEM: Northeast monsoon, SWM: Southwest monsoon and the dry season includes SUMM1 and SUMM2.

Temple and disturbancelandscape features.

The number of buildings in the temple and Dark room availability in the temple complex significantly affected species richness and bat abundance (counts) in temples **(Fig 3,5a)**. Dark room availability positivelystrongly influenced the total abundance of bats and only affected weakly impacted species richness but strongly the total abundance of bats (**Fig 35b,** **Table 23a).** The top lmer model explained 697% of the variation in species richness of which 585% were explained by the fixed factors. Variations between temples was accounted by the random factors was high.

LanduseLand use elements did not show a very strong effect on abundances but availability of paddy(**Fig 6**) and water had a negative influence on species richness while bBanana availability and water hads a negative influence on abundance. The availability of open scrub and uncultivated areas hads a positive influence on abundance but much less on species richness (**Table 3b**).

Fig 35a. Effect plots showing the rRelationship between species richness, abundance and number of buildings and dark rooms in temples

Fig 5b. Effects plots showing the relationship between dark rooms (DR), and number of buildings on bat abundances.

Commented [A18]: This could use reference to some of the output values

Commented [A19R18]: incorporated

Formatted: Font color: Text 1

Formatted: Font color: Text 1

Formatted: Font color: Text 1, Strikethrough

Formatted: Font color: Text 1

Formatted: Font: 12 pt, Font color: Text 1

Formatted: Font color: Text 1

Formatted: Right: 0.16", Space Before: 0 pt, Line spacing: 1.5 lines

Formatted: Right: 0.16", Space Before: 0 pt

Formatted: Indent: Left: 0.07", Right: 0.16", Space Before: 0 pt, Line spacing: 1.5 lines

Fig 6. Effects plots showing the relationship between paddy availability and bat species richness. The plot is based on the most parsimonious model.

Table 23: Effects of Temple characteristics on Two GLMM models were fit separately for temple features and landscape elements for species richness (lmer model) and bat abundances(glmm-admb model) in temples. Temple id and yearmonth areis included as considered as random factors.

Renovation of temple structures ~~and scale of visitors to the temple~~ had a strong negative influence on bat species richness while the scale of visitors to the temple negatively affected ~~and~~ abundance (**Fig ~~4~~6, ~~Table 3~~**). –

Species wise response

~~Response~~ to microhabitat and disturbance ~~habitat and landscape features~~ varied ~~across species~~ (**Table ~~2~~4**). *Hiposederous speoris* abundance was positively influenced by dark rooms and~~, the~~ the number of buildings but not by any disturbance factors.

~~At the landscape level it was and negatively influenced by banana and positiveypositively by open scrub.~~ Counts of *T.melanopogon* and~~, and~~ *T.aegyptica* ~~*and M.lyra R.leschenaulti*~~ were also positively related to the number of buildings in the temple but were also affected negatively by renovation. ~~*R.leschenaulti* was also affected by buildings while~~ *M.lyra* also showed a negative response to ~~to dark rooms, presence of tower and visitors levels~~ renovation while~~and corridors and positive response to trees in the temples.~~ *R.leschenaulti* was ~~was~~ not affected by any temple characteristics or disturbance. ~~but showed negatve influence of visitor levels~~~~*or disturbance factors.*~~

Fig 4. Effects plots showing the relationship of disturbance on species richness and abundance.

Landscape

~~The proportion of landuse around the temples changed with distance. At 5km from the temple it was dominated by crop (63%) followed by urban areas(13%), scrub(12%), trees(7%) water(5%) and grass and bare areas(0.23%) and at 500m; crop (54%) continued to dominate followed by urban areas(36%), scrub(0.7%), trees(3%) water(6%), grass and fallow (0.09%). Since temples are located close to settlements urban area is high near the temple and crop, trees, scrub and grass areas are less. The effects of land use are considered influential based on non- overlapping confidence intervals to prevent ambigiuous inference of effects (Payton et al. 2003). Species richness was affected related only by to grassland around the temples especially at 3km and model estimates increase with increasing distance (TableFig). and aAbundance was related positively to scrub availability and negatively to tree and water and at. At 5km, even to~~

Formatted: Font: Bold, Font color: Text 1
Formatted: Font color: Text 1
Formatted: Font: Bold, Font color: Text 1
Formatted: Font color: Text 1
Formatted: Right: 0.16", Space Before: 0 pt
Formatted: Font: Not Italic, Font color: Text 1
Formatted: Font color: Text 1
Formatted: Font: Not Italic, Font color: Text 1
Formatted: Font color: Text 1
Formatted: Font: Not Italic, Font color: Text 1
Formatted: Font color: Text 1
Formatted: Font: Not Italic, Font color: Text 1
Formatted: Font color: Text 1
Formatted: Font: Not Italic, Font color: Text 1
Formatted: Font color: Text 1
Formatted: Font: Not Italic, Font color: Text 1
Formatted: Font color: Text 1
Formatted: Font: Bold, Font color: Text 1
Formatted: Font color: Text 1

urban areas (Table 5), water and trees had a strong negative effect.

Hiposederous speoris also showed a positive effect of scrub on its abundance across the distance interval. Grasslands also positively influenced abundance but it showed high variations. Trees had a negative effect and wasere strongest at 1km (fig 6, table). M.lyra and *T.aegyptica both were*was negatively affected by all the landuse features but strongly by grasslands and crops up to 53km while. Trees had a weak positive effect at 5km. *R.leschenaulti* had a negative response to trees in the wider landscape up to 3km while grasslands and water had a positive effect. For *T.aegyptica* water in the temple had a positive effect while further away water, crop, grassland negatively influenced its count. *T.melanopogon and R.leschenaulti* did not show any effect of land use on their roost abundanceis negatively affected by water in temples while grasslands had a positive effect further away in the landscape.The percentage of land-use around the temples changed with distance. At 5km from the temple crops (63%) dominate followed by urban areas(13%), scrub(12%), trees(7%) water(5%), grass and bare areas(0.23%) and at 500m; crop (54%) continued to dominate followed by urban areas(36%), scrub(0.7%), trees(3%) water(6%), grass and bare (0.09%). Since temples are close to settlements urban area is high near the temple and crop, trees, scrub and grass areas are less**(Fig 5)**. Grassland around the temples especially at 3km positively affected species richness and scrub availability positively affected the abundance while urban areas had a negative effect at 5km (Table 5). Trees in the temple complex had a positive effect on species richness (**Table 3**). Scrub availability also had a positive effect on *Hiposederous speoris* abundance across the distance interval. Trees had a negative effect and were strongest at 1km (**Table 3**). M.lyra and *T.aegyptica* both were negatively affected by crops up to 5km while. *T.melanopogon* and *R.leschenaulti* did not show any effect of land use on their roost abundance.

Fig 5. High resolution (10m) land-use around the temples sampled. Land-use classification obtained from Karra et al 2021. In the analysis grass and bare areas, and water and flooded areas were combined. See data analysis for more details.

Since none of the landscape elements proved statistically significant(p<0.05) we used thThe model estimates which represent the best-guess average effects on the population and are a better understanding of the variance in the effect of these elements on bat abundances and species richness. There was no significant did not show any Species richness was strong effectassociation of between species richness and landscape features on species richness and (fig x, Table) while abundance is positively influenced by

Formatted: Font: Not Italic, Font color: Text 1
Formatted: Font color: Text 1
Formatted: Font: Not Italic, Font color: Text 1
Formatted: Font color: Text 1
Formatted: Font: (Default) Times New Roman, 12 pt, Font color: Text 1
Formatted: Font: (Default) Times New Roman, 12 pt, Font color: Text 1
Formatted: Font: (Default) Times New Roman, 12 pt, Font color: Text 1
Formatted: Font: (Default) Times New Roman, 12 pt, Font color: Text 1
Formatted: Font: (Default) Times New Roman, 12 pt, Font color: Text 1
Formatted: Font: Bold
Formatted: Font: (Default) Times New Roman, 12 pt, Font color: Text 1
Formatted: Font: (Default) Times New Roman, 12 pt, Font color: Text 1
Formatted: Font: (Default) Times New Roman, 12 pt, Font color: Text 1
Formatted: Font: (Default) Times New Roman, 12 pt, Font color: Text 1
Formatted: Font: (Default) Times New Roman, 12 pt, Font color: Text 1
Formatted: Font: Bold
Formatted: Font: (Default) Times New Roman, 12 pt, Italic, Font color: Text 1
Formatted: Font: (Default) Times New Roman, 12 pt, Font color: Text 1
Formatted: Font: Bold
Formatted: Font: (Default) Times New Roman, 12 pt, Bold, Font color: Text 1
Formatted: Font: Bold
Formatted ...
Formatted ...
Formatted ...
Formatted ...
Formatted ...
Formatted ...
Formatted ...
Formatted: Font color: Text 1
Formatted: Font: Bold
Formatted: Font: Bold, Font color: Text 1

negatively affected by scrub across all distance grassland water up to 3km both in from the temple(Beta 0.017(0,0.033) and farther away at 5km(-0.072 (-00131, -0.013) but the variations become highpositively influenced by trees(model coefficient β=0.01) and water(β =0.03) in the temple complex. Away from the temple. Similarly, Ttrees had a significant positive effect at the temple but negative effect farther away but positive at the temple while urban habitats had a negative effect at 5km. *Hiposederous speoris* Hipposiderous positively responded to water till 3km and negative after that. Urban areas and trees consistently had a negative influence across the distance while open habitats and crops had a marginal positive influence. M.lyra was negatively affected by water till 3km. *R.leschenaulti* Roseatus had a negative response to trees in the wider landscape up to 3km while it preferred temples with trees. Urban areas and water have a positive influence on it at 3km. For *T.aegyptica* Tadardia water was critical at the temple but did not prefer temples surrounded by water up to 3km while trees had a negative influence on it in the landscape. *T.melanopogon* Taphazhus is negatively affected by water in temples while other factors do not show much association.

Open habitats and crops had a positive effect farther away from the temple. species richness was positively influenced related to by grass and fallow and increased from (Coeff value 0.01 at the temple (β = 0.01) with distance w(0m) to 1.07 atto 5km (β = 1.07). hile bBuilt area in the 5km distance had a negative influence(β= -0.06, (Fig 5).) while no other landscape variable had any effect.(Fig 5.Supp. tableTable x1).
Abundance was positively influenced by trees(β = 0.02) and negatively by water(-0.17) only in the temple complex. At 500m and 1km abundance it was negatively influenced by grass and fallow land (β = 0.27) but was positive at 5km. Scrub availability was and positively by availability of scrub(β = 0.24) while trees and crop had a negative effect all along the gradient and at larger scale built area had a negative effect.

No and water negatively affected by urban areas at 5km and positively by grass and fallow land at 1km from the temple. Number of trees in the temple complex had a positive effect on species richness and abundance.

Species richness was in the temple complex (0m) had a positive influence on species richness,bat abundance *At variable distances from the temples trees generally had a negative effect(table).*

Formatted: Font color: Text 1
Formatted: Font: Bold, Font color: Text 1
Formatted: Font color: Text 1
Formatted: Font: Bold, Font color: Text 1
Formatted: Font color: Text 1
Formatted: Font: Bold, Font color: Text 1
Formatted: Font color: Text 1
Formatted: Font: Bold, Font color: Text 1
Formatted: Font color: Text 1
Formatted: Font: Bold, Font color: Text 1
Formatted: Font color: Text 1
Formatted: Font: Bold, Font color: Text 1

and M.lyra, *T.melanopogon and R.leschenaulti while had no effect on others.* Species richness was negatively affected by urban areas at 5km distance and positively by grass and fallow land at 1km from the temple. Number of trees in the temple complex had a positive effect on species richness and abundance . Further away from the temple trees, trees had a significant negative effect and at 5km trees and water availability had a negative effect.

At the species level trees in the complex trees or water availability within the temple did not have any significant effect on any of the species(Fig 6). Further away *H.speoris wasere positively influenced by scrub and grass and fallow upto 500m and availability beyond, scrub was positive but grass and fallow had a negative effect. At 5km built area had a negative influence while and crop had no influence at any scale.*

For 1km while built area around the temple had a negative effect. Megaderma, lyra and R.leschenaulti all landuse showed negative influence up to 3km while grass and fallow, scrub and water became positive at 5km. *and T.aegyptica* was negatively affected by scrub closer to the temple while grass and fallow had a positive influence at larger spatial scalescrop avaibality at all scales. Roseatus and Taphazhus did not show any effect of landuse. *T.melanopogon* was largely infoenced by more grass and fallow further away from the temple (>1km) while water had a negative effect closer to the temple.

Trees around temples and presensepresence of tower had a negative influence on *H.speoris* and *T.aegyptica*. All of these are weak responses(Table 4). LanduseLand use did not have strong influence expect except banana having a negative influence on *H.speoris*. Availability of open scrub hads a positive influence across on all the species except *T.melanopogon* which also showed a negative effect of water. (Table 4).

Table 4: The top models(ΔAICc<5) of species response to microhabitat (temple model) and landuse elements (landscape model). Bld-No.buildings,DR:Dark Room,TR: Trees,HA:Hall,TW:Tower,BA:Banana,PD:paddy,SC:Scrub,VL: No. villages,WA:Water, NULL: Null model

Table 3545: Species response to microhabitat and land-usescape elements availability. Model averaged parameter estimates of top models (<25 ΔAICc) with non--overlapping confidence intervals shown in the last column*

Formatted: Font color: Text 1
Formatted: Font: Bold, Font color: Text 1
Formatted: Font: Bold, Font color: Text 1, Not
Formatted: Font: Bold, Font color: Text 1
Formatted: Font: Bold, Font color: Text 1, Not
Formatted: Font: Bold, Font color: Text 1
Formatted: Font: Bold, Font color: Text 1, Not
Formatted: Font: Bold, Font color: Text 1
Formatted: Font: Bold, Font color: Text 1, Not
Formatted: Font: Bold, Font color: Text 1
Formatted: Font: Bold, Font color: Text 1, Not
Formatted: Font: Bold, Font color: Text 1
Formatted: Font: Bold, Not Italic, Font color: Text 1
Formatted: Font: Bold, Font color: Text 1
Formatted: Font: Bold, Font color: Text 1, Not
Formatted: Font: Bold, Font color: Text 1
Formatted: Font: Bold, Not Italic, Font color: Text 1, Not Highlight
Formatted: Font: Bold, Not Italic, Font color: Text 1
Formatted: Font: Bold, Not Italic, Font color: Text 1, Not Highlight
Formatted: Font: Bold, Font color: Text 1, Not
Formatted: Font: Bold, Not Italic, Font color: Text 1, Not Highlight
Formatted: Font: Bold, Font color: Text 1
Formatted: Font: Bold, Font color: Text 1, Not
Formatted: Font: Bold, Font color: Text 1
Formatted: Font: Bold, Font color: Text 1, Highlight
Formatted: Font color: Text 1, Highlight
Formatted: Font: Bold, Font color: Text 1, Highlight
Formatted: Font color: Text 1, Highlight
Formatted: Font color: Text 1
Formatted: Font: Not Bold, Font color: Text 1
Formatted: Font color: Text 1

Though TThe availability of roost microhabitats across temples with and without bats was not significantly different (Kruskal WwallisS test; D=0.4, p=0.689), dark rooms, tower, and number of buildings were more in temples with trees (Mann-Whitney U=3917 p<0.01, Trees in the vicinity of temple was higher in bat occupied sites (Mann-Whitney U=3917 p<0.01, **Fig 6577**). Of the total temples sampled, 80% of them had bats across all the years and they were sampled. and they were specific to these temples and showed strong affinity to them. iIn only 4/57 temples we found bats abandoning the temple. Renovation levels were higher in temples with no bat (x+sd=0.34+0.48,) with 44% (15/34) compared of temples without bats were under renovation, than with temples having bats temples (x+sd=0.14+0.34) 35%(16/46) of temples similarly, visitor levels were also marginally higher in no bat (2.11+0.79) compared towith bat temples with bats(1.89+.71). Land use High-resolution l

Land use Habitat availability The models using both coarse 100m data and 1high resolution 10 m data did not showed little difference between bat occupied and not occupied temples showed no differences except for trees in the vicinity of temples which was higher in bat occupied sites (Mann-Whitney U=3917 p<0.01) any significance effect of land use on bat abundances(**Fig 767**). There was no overall difference in land use between bat vs no bat temples (paddy, Mann-Whitney, U :5037, p=0.236, water U :5159.5, p=0.73365, banana U: 4569.5, p=0.057917; coconut U: 5561.5,
p=0.960, veg U: 90 p=0.78717, **Fig 8**).

The availability of microhabitats in the temple especially the number of buildings and dark rooms is, therefore, a key determinant of bats in temples while land use elements had no or negligible only a weak effect.

Fig 657. Proportional availability of habitats in temples with and without bats.

Fig 768. Mean land-use elements value of habitat variables around temples with and without bats at various distances. * indicate significant differences at p< 0.05. 5km around the temples across bat species.

Formatted: Font: 12 pt, Font color: Text 1
Formatted: Font color: Text 1
Formatted: Font: 12 pt, Font color: Text 1
Formatted: Font color: Text 1
Formatted: Font: 12 pt, Font color: Text 1
Commented [A20]: Not sure what this means
Commented [A21]: Any tree in the 5km?
Formatted: Font color: Text 1
Formatted: Font color: Text 1
Formatted: Font: 12 pt, Font color: Text 1
Formatted: Font color: Text 1
Commented [A22]: I don't think this study allows for this conclusion
Commented [A23R22]: Agreed. Modified it to say that temples with bats were consistently occupied across years.
Formatted: Font color: Text 1, Strikethrough
Formatted: Font color: Text 1
Formatted: Font color: Text 1, Strikethrough
Formatted: Font color: Text 1
Formatted: Font color: Text 1, Not Highlight
Formatted: Font color: Text 1
Formatted: Font color: Text 1, Not Highlight
Formatted: Font color: Text 1
Formatted: Font color: Text 1, Not Highlight
Formatted: Font color: Text 1
Formatted: Font color: Text 1, Not Highlight
Formatted: Font color: Text 1
Formatted: Font: Bold, Font color: Text 1
Formatted: Font color: Text 1
Formatted: Font: Bold, Font color: Text 1
Formatted: Font color: Text 1
Formatted: Font color: Text 1, Highlight
Formatted: Font color: Text 1
Formatted: Normal, Left, Right: 0.06", Space Before: 0 pt
Formatted: Font color: Text 1
Formatted: Font color: Text 1

Discussion

Why do bats prefer some temples? Bats were found in about 80% of the temples and over 20,211 individuals were counted. Though we recorded only 7 species the most common species *Hipposideros speoris* (9715) accorded for nearly half of the population and also found in more than 50% of bat inhabited temples. *Hipposideros* ~~*H.*~~ *speoris* is a very common species across India and Srilanka and ~~is known to~~ occurs in large congregations and shows high fidelity to roost sites (Selvarathinam and Marimuthu, 2013). The other 6 species are also not rare or threatened and ~~known to~~ use a multitude of anthropogenic structures to roost (Bates and Harrison, 1997). Except for *T. aegyptiaca, Pipistrellus sp* and *Rhinopoma hardwickii* species counts of all others ranged between 450 to 200 in each temple and appear to follow the general tendency of bats that roosts in caves and manmade structures, to be highly gregarious (Gaisler, 1979). Crevice roosting *T. aegyptiaca* and *Pipistrellus sp* species may have been underestimated because they are hard to count inside the narrow crevices. ~~The overlap between large insectivorous species preferring dark rooms such as *M.lyra* and *T. melanopogon* at roost was higher than for smaller *H.speoris* and *T.aegyptica* and the frugivorous *R.leschanaulti*. This could be because *M.lyra* is a predator of larger animals including rodents and small bats (Audet et al., 1991; Bates and Harrison, 1997), and species such as *T.aegyptica* may tend to avoid it even though they occupy a different roost microhabitat. While larger *T. melanopogon* seem to be unaffected which may be due to the behavior of *T. melanopogon* which is not only large but huddles together while roosting unlike the other species and therefore may be less prone to gleaning attacks from *M. lyra*. This may also explain why we see a greater overlap between *M.lyra* and *T.melanaopogon* but much less with *H.speoris*. The greater niche breadth of *H.speoris* also facilitates it to coexist with these species in the same habitat.~~

~~Most species~~ counts ~~decline in the temples during the northeast monsoon. The NEM is also a time of high rainfall, extensive paddy cultivation, and time of high insect activity. This is also the parturition and lactation period for most bats in the region and species like *Hipposideros alter(not recorded in this survey)* take advantage of abundant Lepidoptera prey during the monsoon months in the study area (Sophia and Vanitharani 2006). But studies in Thailand on similar species have shown insect diversity is less influential than water availability (Suksai and Bumrungsri 2018). During the monsoons bat abundance falls and also the proportion of temples with bats have decreased and they seem to have dispersed probably the young ones have moved~~

Formatted: Font: 18 pt, Font color: Text 1

Formatted: Font: 20 pt, Font color: Text 1

Formatted: Font color: Text 1

Formatted: Font: Not Italic, Font color: Text 1

Formatted: Font color: Text 1

Formatted: Font: 12 pt, Font color: Text 1

Formatted: Font color: Text 1

into other habitats or even using dense foliage. This was true for the frugivore *R.leschanaulti* but also *M.lyra* and *T.aegyptica. M.lyra* breeds during November and sexes disperse after copulation leaving behind females at most roosts(Harrison and Bates 1997). This could be a reason for a fall in numbers after November but may not be true for *H.speoris* whose numbers don’t change in the monsoon as males pair with females for a year or more and do not desert the roost (Chanderasekaran 2005). *R.leschanaulti* is known to favour banana plantations as show in Kerala state(Deshpande 2012) and could be moving roosts depending on the availability of fruits even thoughhowever peak monsoon is not abundant in bat fruits except for sporadic fruiting of *Ficus sp*. (Cauglin et al 2012). Since the relationship with land use variables are weak we suspect the fall in numbers during the monsoon is more due to dispersal of young bats than the effect of any particular landuse.

Several species that roost and breed in temples across India (Bates and Harrison, 1997; Leippert et al., 2000), are cave-dwelling species that have found temples as suitable alternative habitats(Bates and Harrison, 1997; Leippert et al., 2000) to colonize. Our results show that temples offer dark, undisturbed places roosts and crevices similar to caves and these are harboursused by 6-/7 species accounting for 80% of the abundance of bats in the temples. While we have pooled different types of dark spaces available in the temples, the one behind the main door of the temple and through which the temple tower can be accessed, is the least disturbing active place and is occupied whenever there was no disturbance. These dark areas in old temples have several openings through which bats can easily fly out, a reason for their such places selectioned by most bat species. Granite stones used to build All the ancient temples are stacked together and offers crevices for some species to roost such as the free-tailed bats (*T. aegyptiaca*) which often roost at eye level between pillarsare were built with granite stones stacked together that offered crevices for some species to roost such as the free-tailed bats (*T. aegyptiaca*) which often roost at could be seen even at eye level between pillars. Walkways Corridors (halls) surrounding the temple *sanctum* where the idol of the god is kept, is often long and dark during the day with a high roof which is a preferred place for the bats like *H. speoris* that remain unnoticed and therefore undisturbed during the morning hours when there is a movement of people. A similar response ofto cave-dwelling bats fromof genus *Hipposideros* has been found in Malaysia where they roost in very dark corners and high places (Rajasegarn et al 2018). We did not take species-wise roost height measurements so it’s not evident if species segregate roost according to height but unlike in a cave, many species that roost in dark corners seem to segregate spatially and can vary between temples.

Formatted: Font color: Text 1

Formatted: Font: 12 pt, Font color: Text 1

Formatted: Font color: Text 1

This requires some additional work.

This study demonstrates that dDisturbance tof roosts is a major factor affecting roosting bats as many other similar studies have demonstrated in old buildings under temperate conditions (Kunz and Reynolds 2003, Fagan et al 2017)-. The effect was less impactful on the abundant *Though not quantified, T*the *study has shown except for H.speoris*, while other all species are negatively affected by renovation and some by the visitors levels. Large temples usually have more visitors but also more buildings and therefore the more roosts possibility of harbouring more roosts and therefore even when disturbance levels increase bats might persist by moving to other buildings in the temple temporarily. While there could be several other factors affecting bats in temples like noise levels which can be of very high decibel levels during festivals, overlighting during festivals, prayer related smoke and day-long activities during festivals we could not measure these but needs to be considered when making management decisions for bat conservation. However, in many cases, these disturbance activities are routine in temples and bats have likely got accustomed to these significant threats but to bats in the temples is coming from the recent spurt in the renovation of old temples where dark areas are lit, pressure cleaned to remove the odour of bats, painted and 'bat proofed' to keep away bats and pigeons from entering the premises is a major one and can effectively prevent bats from colonizing temples (Ganesh et al 2020). With current coronavirus and bat association being misreported by media bats have come under even greater threat and surveillance. Our surveys of bats in the same temples have shown that they have deserted some temples completely because of disturbances such as renovations and probably moved to other temples (Selvarathinam and Marimuthu 2013). Temples are in many places the only habitat for bats in the landscapes as many of the rocky hills have been destroyed. Therefore just like bats roosting in public and historical Buildings roosting bats are need to be managed , not only to promote their persistence, on the landscape, but also to mitigate human–wildlife conflict,. This is particularly true of bats roosting in buildings used by the public, where bats may prevent present a risk to human health through potential zoonotic disease transmission (e.g., histoplasmosis and rabies; Emmons et al. 1966, Constantine 2009, Streicker et al.

2013), and. Noise and artificial illumination associated with the presence of people at roost sites can also have detrimental impacts on bat behavior and survival (Mann et al. 2002, Boldogh et al. 2007, Cardiff et al. 2012, L opez-Roig and Serra-Cobo 2014). Management decisions are further complicated when bats roost in historical buildings because bat guano and urine fromcan

Formatted: Body Text, Indent: Left: 0.07", Right: 0.07"

Formatted: Font: Times New Roman, 12 pt, Font color: Text 1

Formatted: Font color: Text 1

Formatted: Font: Italic, Font color: Text 1

Formatted: Font color: Text 1

Formatted: Font: Times New Roman, 12 pt, Font color: Text 1

damaginge the historical integrity of structures (Hales 2014), temples need to develop a comprehensive plan to let bat persist on the landscape and provide the ecosystem services rather than adhoc prevention of bats in temples. Temples are in many places the only habitat for bats in the landscapes as many of the rocky hills have been destroyed.

Formatted: Font color: Text 1

~~Why no landuse effect?~~

While temple characteristics and disturbances are major drivers of bat abundance and occupancy, ~~the~~ land use ~~especially crop availability does not~~ seems to play a less~~n~~ important role across species. Several studies have shown the importance of landscape elements for the presence of bats (Ford et al., 2006; Neubaum et al., 2007, Bellamy and Scot, 2013, Treitler et al. 2016) ~~)~~ but in our case, there seems to be no single habitat effect. Fine-scale (10m) mapping of land-use around temples revealed differential use of habitat elements by species but these were either weak effects or had high variations in the effect.~~.~~ The landscape as mentioned earlier is highly homogeneous; crops and urban areas together constitute about 70-80% with scrub, ~~and~~ grasslands ~~open areas,~~ water bodies ~~wetlands~~ and trees comprising the rest in small~~equal~~ proportions. Even though c~~C~~rops were everywhere ~~and Open habitats only~~ they had a ~~very only a marginally~~ low negative ~~but positive positive~~ effect on *M.lyra* and *T.aegyptiaca* ~~bat counts~~ while trees and water availability seem to have a consistent negative influence across species. The availability of scrub and grasslands is critical for species richness and the overall abundance of bats. These are the only two natural habitats available the rest being tree plantations, ~~or~~ invasive *Prosopis juliflora* ~~(trees)~~ and artificial wetlands. Even though scrub and grasslands are less than 5~~1~~% of the area it has a dis~~s~~propor~~opri~~tionate and mostly positive effect on all the species except *M.lyra*. The radio~~-~~tracking study in south India of the insectivorous *M.lyra* showed it has a very flexible diet and can take prey both from air and ground which could enable it to use multiple habitats but ~~it~~ prefers to forage mostly within 500m from temples(Audet et al 1991). The importance of natural habitat is emphasized for many species including bats(Razgour et al 2011) and this is evident in this study. Effect of water was variable with~~but~~ species like *T. aegyptiaca* ~~Tardia~~ seen~~m~~ to prefer water points in the vicinity of the t~~t~~emple but~~and~~ this may not be for water as~~but~~ ~~nd they use the~~ large open wells in some temples are active sites for foraging~~Scrub and grass and fallow habitats had a higher effect than other elements and these often had a negative effect closer to the temple and positive at larger distances except for *H.speoris*~~. Water availability is important for bats and may drive bat movement and activity across the landscape (Geluso and Geluso, 2012; Rainho and Palmeirim, 2011) and studies in Thailand on similar species have shown insect diversity is less influential than water availability (Suksai and Bumrungsri 2018).

Formatted: Font: 12 pt, Font color: Text 1
Formatted: Font color: Text 1
Formatted: Font: (Default) Times New Roman, 12 pt, Font color: Text 1
Formatted: Font color: Text 1
Formatted: Font: Italic, Font color: Text 1
Formatted: Font color: Text 1
Formatted: Font: Italic, Font color: Text 1
Formatted: Font color: Text 1
Formatted: Font: Italic, Font color: Text 1
Formatted: Font color: Text 1
Formatted: Font: Italic, Font color: Text 1
Formatted: Font color: Text 1
Formatted: Font: Italic, Font color: Text 1
Formatted: Font color: Text 1
Formatted: Font: Not Italic, Font color: Text 1
Formatted: Font color: Text 1
Formatted: Font: Not Italic, Font color: Text 1
Formatted: Font color: Text 1
Formatted: Font: Not Italic, Font color: Text 1
Formatted: Font color: Text 1
Formatted: Font: Not Italic, Font color: Text 1
Formatted: Font color: Text 1
Formatted: Font: Italic, Font color: Text 1
Formatted: Font color: Text 1
Formatted: Font: Times New Roman, Font color: Text 1, Not Highlight
Formatted: Font color: Text 1, Not Highlight
Formatted: Font: Times New Roman, Font color: Text 1, Not Highlight
Formatted: Font color: Text 1, Not Highlight
Formatted: Font: Times New Roman, Font color: Text 1, Not Highlight
Formatted: Font color: Text 1, Not Highlight
Formatted: Font color: Text 1

However, and in this study, the effect was weak and negative for most species. This could be because there is no constraint for in water availability in the region because of the perriennial river and extensive irrigation ponds and excessive water can have a negative effect especially when the sampling was done during the cropping season. A more nualnced study and analysis will need to be done to see how water avaibiliy effects bats in the dry seasons.

Grass and fallow constitute 0.1% of the land use but appear to have a disproportionate effect on the bats. *H.speoris* is showing a negative effect of built area across different scales and Roseatus has a negative effect of trees with We found strong response to even though we tried analysis at a coarse scale of 100m and finer scale of 10m but both did not reveal any significant effect of any habitat variables.Several studies have shown the importance of landscape elements for the presence of bats (Ford et al., 2006; Neubaum et al., 2007).Scale issues are important for many species including bats(Bellamy and Scot, 2013) and in this study at 5km from the roost most of the land-use effects became negligible or the direction of effects changed from positive to negative or vice versa. The importance of natural habitat is emphasized for many species including bats(Razgour et al 2011) and this is evident in this study as well. Open habitats comprising of grasslands, scrub and bare ground comprise about 5% of the land around temples which is significant because the models do not show negative effect of these on bat abundances across species. In this study however, we did not find such a strong response even though we tried analysis at a coarse scale of 100m and finer scale of 10m but both did not reveal any significant effect of any habitat variables.. The extent of natural habitat increases with distance and this may influence bats to forage at larger spatial scales. Temples are located mostly close to human habitations and rivers while natural open dry habitats are further away and bats may be making regular forays as observed in UK bats (Razgour et al 2011). This corroborates with less preference offor water and urban areas with distance when more suitable habitats are available.

This study only provides a very broad and therefore limited view of land-use effect on bats. MA more nualnced study and analysis will need usingto use radio telemetry and acoustic sampling to be done to see how land use elemensts influence water avaibiliy effects bats. Foraging bats are most strongly associated with variables measured at small spatial scales and distance measures (Bellamy and Scot 2013). For instance, the only study on radio telemetry of temple bats shows 41% of the teime the bats foraged 500m from the temples but ranged up to 4km over the night during the summer months. This may be different in rainy months when the general productivity

Formatted: Font color: Text 1, Not Highlight
Formatted: Font color: Text 1
Formatted: Font color: Text 1, Not Highlight
Formatted: Font color: Text 1
Formatted: Font color: Text 1, Not Highlight
Formatted: Font: Times New Roman, 12 pt, Font color: Text 1, Not Highlight
Formatted: Font: Times New Roman, 12 pt, Font color: Text 1
Formatted: Font: Times New Roman, 12 pt, Font color: Text 1, Not Highlight
Formatted: Font: Times New Roman, 12 pt, Font color: Text 1
Formatted: Font: Times New Roman, 12 pt, Font color: Text 1, Not Highlight
Formatted: Font: Times New Roman, 12 pt, Font color: Text 1
Formatted: Font: Times New Roman, 12 pt, Font color: Text 1, Not Highlight
Formatted: Font: Times New Roman, 12 pt, Font color: Text 1
Formatted: Font color: Text 1
Formatted: Font: Italic, Font color: Text 1
Formatted: Font color: Text 1
Formatted: Font color: Text 1, Not Highlight
Formatted: Font color: Text 1
Formatted: Font color: Text 1, Not Highlight
Formatted: Font color: Text 1
Formatted: Font color: Text 1, Not Highlight
Formatted: Font color: Text 1
Commented [A24]: I think this had to do with the very course grain analysis of land use.
Commented [A25R24]: We have addressed the issue of ...
Formatted: Right: 0.08", Space Before: 10.3 pt
Formatted ...
Formatted: Font color: Text 1
Formatted ...
Formatted: Font color: Text 1
Formatted ...
Formatted: Font color: Text 1
Formatted ...
Formatted: Font color: Text 1
Formatted ...

of the habitat is more and larger use of the landscape may happen as scrub and grassland habitats also become ~~more~~ richer in insect fauna during the rains. Further, the land-use change has to be included along with seasonal changes in bat occurrence which haves not been entirely possible ~~been difficult~~ in this study but with more advanced remote sensining tools this is ~~may be~~ possible. A recent study in the same study area using radar images ~~infrared remote sensing~~ has shown a greater grassland area ~~availability~~ thant by conventional means used here(Samrat~~Abhishek~~ et al 2021). Such an approach ~~This~~ will also enable mapping across seasons irrespective of cloud cover. ~~in the dry seasons.~~

The current study relies on the most recent high--resolution land-use analysis (Karra et al.20210) but the study was dfone in 2012-2019. This mismatch as mentioned earlier may not be very high given the land-use has remained largely the same over this period. However, for small--sized land-use such as natural habitats with less than 5% of the area and which are critical for bats, even small changes can result in large differences for a foraging animal. Further, tThe dynamics of wetlands has not been incorporated in this study but since it was mostly in the wet seasons we assume it to be ~~very~~ stable across years but a more nuanced classification of thisese dynamics around each temple might be important as irrigation systems and water availability are driven not entirely by monsoon patterns ~~complex~~ in the region (Nikil 2020).

~~Razgour, O., Hanmer, J. & Jones, G. (2011) Using multi-scale modeling to predict habitat suitability for species of conservation concern: the grey long-eared bat. Biological Conservation, 144, 2922–2930.~~ ~~The landscape was fairly homogeneous around the temples with paddy or banana grown around almost all temples across years.~~

~~Mismatch in temporal scale~~

~~Classification issues- landscape characterization is less precise with global datasets~~

~~Finer scale needed: Foraging bats are most strongly associated with variables measured at small spatial scales and distance measures (Bellamy and Scot 2013).~~

Formatted: Font color: Text 1
Formatted: Font: Times New Roman, 12 pt, Font color: Text 1
Formatted: Font color: Text 1
Formatted: Font: Times New Roman, 12 pt, Font color: Text 1
Formatted: Font color: Text 1
Formatted: Font: Times New Roman, 12 pt, Font color: Text 1
Formatted: Font color: Text 1
Formatted: Font: Times New Roman, 12 pt, Font color: Text 1
Formatted: Font color: Text 1
Formatted: Font: Times New Roman, 12 pt, Font color: Text 1
Formatted: Font color: Text 1
Formatted ...
Formatted: Font color: Text 1
Formatted ...
Formatted: Font color: Text 1
Formatted ...
Formatted: Font color: Text 1
Formatted ...
Formatted: Font color: Text 1
Formatted ...
Formatted: Font color: Text 1
Formatted ...
Formatted: Font color: Text 1
Formatted ...
Formatted: Font color: Text 1
Formatted ...
Formatted: Font color: Text 1
Formatted ...
Formatted: Font color: Text 1
Formatted ...
Formatted: Font color: Text 1
Formatted ...
Formatted: Font color: Text 1
Formatted ...
Formatted: Font color: Text 1

While bats were missing in many temples that had dark rooms, crevices, and halls, this could be more due to other disturbances such as festivals or renovation (Ganesh et al 2020). Temples with less trees and towers where preferred by *Hipposideros speoris* and *Tadardia aegyptiaca* which can be expected as both species are also dry area species and prefer caves and buildings(Bates and Harrison, 1997) and therefore not associated with wooded area and hence may prefer temples devoid of tree cover. The importance of open uncultivated natural areas such as scrub, grasslands and lightly wooded areas are important for almost all the species of bats and this can have significant conservation importance as natural areas are rapidly diminishing.

The presence of bats is generally linked to microhabitat or stand-level structural conditions, however, Lewis (1995) also found that roost-site fidelity for bats varied depending on the availability and permanency of the roosts used and suggested that fidelity was high at buildings for these reasons. Supporting this a study on banded *H.speoris* bats from the study area showed 82% roost fidelity throughout the year (Selvarathinam, G Marimuthu 2013). Therefore the temples that have remained unchanged for centuries provide conditions for high roost fidelity despite changes in the landscape and therefore are more important for bats to persist in the area.

The importance of open uncultivated natural areas such as scrub, grasslands and lightly wooded areas are important for almost all the species of bats and this can have significant conservation importance as natural areas are rapidly diminishing.

Conservation of bats in temples

The study has shown that microhabitats within temples such as dark corners that are relatively less disturbed and rooms that were rarely used are essential for their persistence of bats. Temples with numerous buildings increase the possibility of having dark rooms. The negative effect of disturbance on bats is strong and can override the availability of microhabitats with temples having similar microhabitats not harbouring bats because of disturbance. This effect of disturbance is species--specific with the frugivores r.leschenaulti and insectivorous H.speoris are not affected by them which is due to their overall ability to return to roost sites after disturbance while others are more sensitive. The landscape factors were surprisingly less

Formatted: Font: 12 pt, Font color: Text 1

Formatted: Font color: Text 1

Formatted: Font: 12 pt, Font color: Text 1

Formatted: Font color: Text 1

~~important though availability of open natural areas around temples are important for the abundance of bat species.~~ Therefore conservation of bats would mean not just protecting the microhabitat in the temples from excessive disturbance but also allowing bats to use such spaces and not bat-proofing the temples~~getting destroyed and retaining natural areas~~. Most of t~~bats roosting in public and historical Buildings are managed to promote their persistence, mitigate human-wildlife conflict, prevent potential zoonotic disease transmission (Emmons et al. 1966, Constantine 2009, Streicker et al. 2013), and bat guano and urine from damaging the historical integrity of structures (Hales 2014), temples need to develop a comprehensive plan to let bat persist on the landscape and provide the ecosystem services rather than adhoc prevention of bats in temples. T~~he landscape factors were surprisingly not~~less~~ important though the availability of natural areas around temples are crucial~~important~~ but are also limited. While bats are known to provide important pest control services in paddy agriculture the role of paddy in sustaining the bat populations is not clear as crops did not have any significant effect on bat species richness and abundance. This calls for a more nuanced study using radio telemetry and acoustic sampling to understand the use of the landscape by bats since natural areas are likely to be converted in the future with a greater push for irrigated agriculture in the area. To make bat conservation a win-win situation bats roosting in public and historical buildings need to be managed to promote their persistence, mitigate human-wildlife conflict, prevent potential zoonotic disease transmission and prevent bat guano and urine from damaging the historical integrity of structures like temples. This can be done with faith groups, temple management and by building awareness among the public to develop a comprehensive plan to let bat persist on the landscape and continue to benefit from the ecosystem services they provide rather than ad-hoc elimination~~prevention~~ of bats in temples.

Formatted: Font color: Text 1

Formatted: Font color: Text 1

Formatted: Font color: Text 1

Formatted: Font color: Text 1

around. Though not quantified, the significant threat to bats in the temples is coming from the recent spurt in the renovation of old temples where dark areas are lit, pressure cleaned to remove the odour of bats, painted and 'bat proofed' to keep away bats and pigeons from entering the premises. With current coronavirus and bat association being misreported bats have come under even greater threat and surveillance. Our surveys of bats in the same temples have shown that they have deserted some temples completely because of disturbances such as renovations and probably moved to other temples. Temples are in many places the only habitat for bats in the landscapes as many of the rocky hills have been destroyed. However, a metapopulation of bat can exist in the landscape which will help colonize temples depending on the temple characteristics and disturbance levels. More studies are needed to understand these dynamics.

Therefore just like bats roosting in public and historical Buildings are managed to promote their persistence, mitigate human-wildlife conflict, prevent potential zoonotic disease transmission (Emmons et al. 1966, Constantine 2009, Streicker et al. 2013), and bat guano and urine from damaging the historical integrity of structures (Hales 2014), temples need to develop a comprehensive plan to let bat persist on the landscape and provide the ecosystem services rather than adhoc prevention of bats in temples. Temples are in many places the only habitat for bats in the landscapes as many of the rocky hills have been destroyed.

Acknowledgement

The work was partly supported by a grant from Bat Conservation International Global Grassroots Conservation Fund. We thank Maria,Lise and K.S.Seshadri for help in the field and M.B.Prashanth for making the map and general support. We are grateful to Dr.R.Ganesan, Dr.M.Soubadra Devy for their help during the initial stages of the work. We also tank Rajkamal, Seshadri, Prashanth and Soubadra for their comments on the MS. We thank Thamizhazhagan for helping in capturing bats and Muthupandi for assistance in the field. Abhishek helped us with landuse analysis and Thalavaipandi drew the map. The Temple authorities and temple staffs in both Tirunelveli and Thoothukudi districts permitted us to do the study. Jermiah Rajanesan, director Dhonavur Fellowship and Maria Joseph, Head Master, Christuraja Hr., Sec., School, Palayamkottai helped us with logistics and we are grateful to them for their help.

Literature cited

Addline, E.P.D., J .Vanitharani., S.Rajkumar, and R.T.Sundara. 2004. Government, law and temple bat management, pp-297-301. In: Muthuchelion,K (eds). Biodiversity Resources Management and Sustainable use. Centre for biodiversity and forest studies, school of energy, environment and natural resources, Madurai Kamaraj University, Madurai.

Audet, D., D.Krull, G. Marimuthu, S. Sumithran, and J. B. Singh. 1991. Foraging Behaviour of the Indian False Vampire Bat, *Megaderma lyra* (Chiroptera: Megadermatidae). Biotropica, 23: 63-67.

Formatted: Font color: Text 1

Formatted: Font color: Text 1, Strikethrough

Commented [A26]: Not sure this is the best way to end, stronger statement can be made here.

Commented [A27R26]: modified

Formatted: Font: 12 pt, Font color: Text 1, Strikethrough

Formatted: Font color: Text 1, Strikethrough

Formatted: Font color: Text 1, Strikethrough

Formatted: Font color: Text 1

Formatted: Font: Times New Roman, 12 pt, Font color: Text 1

Formatted: Font color: Text 1

Formatted: Font: 12 pt, Font color: Text 1

Formatted: Font color: Text 1

Formatted: Font: 12 pt, Font color: Text 1

Barragan, F., C. Lorenzo, A.Moron, M.A. Briones-Salas, and S.Lopez. 2010. Bat and rodent diversity in a fragmented landscape on the Isthmus of Tehuantepec, Oaxaca, Mexico. *Tropical Conservation Science*, *3*(1), pp.1-16.

Formatted: Font color: Text 1

Bates, P.J.J, and D.L.Harrison. 1997. Bats of the Indian Subcontinent. Harrison Zoological Museum Press, England.

Bolker,B.M, H.Skaug, A. Magnusson, A.Nielsen 2012. Getting started with the glmmADMB package. http://admb-project.org.

Bolker, B.M, M. E. Brooks, C. J. Clark, S. W. Geange, J. R. Poulsen, M. Henry, H. Stevens, J.S S. White. 2009.Generalized linear mixed models: a practical guide for ecology and evolution, Trends in Ecology & Evolution, Volume 24, Issue 3,Pages 127-135, https://doi.org/10.1016/j.tree.2008.10.008.

Chandrasekaran, M. K. 2005. Time in the living world. Universities Press, Hyderabad, 197 pp.

Carpenter,E.R Gomez,D.L.Waldein and R.E.Sherwin. 2014. Photographic estimation of roosting density of Geoffroys Rousette Fruit Bat *Rousettus amplexi caudatus* (Chiroptera: Pteropodidae) at Monfort Bat Cave, Philippines. Journal of Threatened Taxa, *6*: 5838-5844.

Cauglin,T, T.Ganesh and M.Lowman. 2012. Sacred fig trees promote frugivore visitation and tree seedling abundance in South India. Current science.102:918-922

Clem, P. D. 1993. Foraging patterns and the use of temporary roosts in female evening bats, *Nycticeius humeralis*, at an Indian maternity colony. Proceedings of the Indian Academy of Science, 102:201- 206.

Deshpande, K. 2012. Assessing diversity and distribution of bats in relation to land-use and anthropogenic threats in the southern Western Ghats, India. *Final Report Submitted to the Rufford Small Grants for Nature Conservation, 30pp*.

Estrada, A., and R. Coates- Estrada. 2002. Bats in continuous forest, forest fragments and in an agricultural mosaic habitat-island at Los Tuxtlas, Mexico. *Biological Conservation*, *103*(2), pp.237-245

Fischer, J., J. Stott, B.S. Law, M.D. Adams, and R.I. Forrester. 2009. Designing effective habitat studies: quantifying multiple sources of variability in bat activity. *Acta Chiropterologica*, *11*(1), pp.127-137.

Ford, W. M., J.M. Menzel, M.A. Menzel, J.W. Edwards, and J.C. Kilgo. 2006. Presence and absence of bats across habitat scales in the upper coastal plain of South Carolina. Journal of Wildlife Management, *70*: 1200-1209.

Furey, N.M. & P.A. Racey. 2016. Conservation ecology of cave bats. In *Bats in the Anthropocene: Conservation of Bats in a Changing World*. C. Voigt & T. Kingston, Eds.: 463–500. Springer International Publishing)

Gaisler. J. 1979. Ecology of Bats. In Ecology of Small Mammals (D M Stoddard.ed.) *Chapman and Hall*, London, Pp 2B1-342. *386* pp.

Ganesh, T., A.Saravanan and M.Mathivanan. 2020. Let’s Protect the Bats Where They Live –

Formatted: Font color: Text 1
Formatted: Font: 12 pt, Not Bold, Font color: Text 1
Formatted: Font color: Text 1
Formatted: Font: 12 pt, Not Bold, Font color: Text 1
Formatted: Font: (Default) Times New Roman, 12 pt, Not Bold, Font color: Text 1
Formatted: Font: 12 pt, Not Bold, Font color: Text 1
Formatted: Font: (Default) Times New Roman, 12 pt, Not Bold, Font color: Text 1
Formatted: Font color: Text 1
Formatted: Font: Not Bold, Font color: Text 1
Formatted: Heading 2, Left
Formatted: Font: 12 pt, Font color: Text 1
Formatted: Font color: Text 1
Formatted: Font: 12 pt, Font color: Text 1
Formatted: Font color: Text 1
Formatted: Font: 12 pt, Font color: Text 1
Formatted: Font color: Text 1
Formatted: Font: 12 pt, Font color: Text 1
Formatted: Font color: Text 1
Formatted: Line spacing: single
Formatted: Font: Times New Roman, 12 pt, Font color: Text 1
Formatted: Font color: Text 1
Formatted: Font: 12 pt, Font color: Text 1
Formatted: Font color: Text 1
Formatted: Line spacing: single

Including in Our Temples (https://science.thewire.in/environment/bats-covid-19-temples-south-india/)

Geluso, K.N., Geluso, K., 2012. Effects of environmental factors on capture rates of 579 insectivorous bats, 1971–2005. J. Mammal. 93, 161–169. doi:10.1644/11-MAMM-A-107.1

Harvey, C.A., A. Medina, D.M. Sanchez, S. Vilchez, B. Hernandez, J.C. Saenz, J.M. Maes, F. Casanoves, and F.L. Sinclair. 2006. Patterns of animal diversity in different forms of tree cover in agricultural landscapes. *Ecological applications*, *16*(5), pp.1986-1999.

Ho, Y.Y., and L.L. Lee. 2003. Roost selection by Formosan leaf-nosed bats (Hipposideros armiger terasensis). *Zoological Science*, *20*(8), pp.1017-1024.

Formatted: Font: Times New Roman, Font color: Text 1

Formatted: Font: Times New Roman, 12 pt, Font color: Text 1

Formatted: Indent: Left: 0.07"

Formatted: Font: Times New Roman, Font color: Text 1

Formatted: Font color: Text 1

Formatted: Font: 12 pt, Font color: Text 1

Formatted: Font color: Text 1

Formatted: Line spacing: single

Humphrey, S.R. 1975. Nursery roosts and community diversity of Nearctic bats. *Journal of mammalogy*, *56*(2), pp.321-346

HR&CE 2020 https://tnhrce.gov.in/hrcehome/temple_list.php

Inger, R. F., H. B. Shaffer, M. Koshy, and R. Bakde. 1987. Ecological structure of a herpetological assemblage in South India. Amphibia-Reptilia, 8: 189–202.

Jenkins, E. V., T. Laine, S. E. Morgan, K. R. Cole, and J. R. Speakman. 1998. Roost selection in the pipistrelle bat, *Pipistrellus pipistrellus* (Chiroptera: Vespertilionidae), in northeast Scotland. Animal Behaviour, 56:909-917

Jones, G., D.S. Jacobs, T.H. Kunz, M.R. Willig, and P.A. Recay. 2009. Carpe noctem: the importance of bats as bioindicators. *Endangered species research*, *8*(1-2), pp.93-115.

Karra,K, C.Kontgis, -Z. Statman-Weil, J.Mazzariello, M. Mathis, S. Brumby 2021~~et al. ~~“. Global land use/land cover with Sentinel-2 and deep learning.” IGARSS 2021-2021 IEEE International Geoscience and Remote Sensing Symposium. IEEE, 2021.

Kerth, G., M. Wagner, and B. Konig. 2001. Roosting together, foraging apart: information transfer about food is unlikely to explain sociality in female Bechstein's bats (Myotis bechsteinii). *Behavioral Ecology and Sociobiology*, *50*(3), pp.283-291.

Korad, V., K. Yardi, and R. Raut. 2007. Diversity and distribution of bats in the Western Ghats of India. *Zoos' Print Journal*, *22*(7), pp.2752-2758.

Kunz, T. H., E. Braun, De Torrez, D. Bauer, T. Lobova, and T.H. Fleming. 2011. Ecosystem services provided by bats. Annals of the New York Academy of Sciences, 1223: 1-38.

Leippert, D., W. Goymann, H. Hofer, G. Marimuthu, and J. Balasingh. 2000. Roost-mate communication in adult Indian false vampire bats (*Megaderma lyra*): an indication of individuality in temporal and spectral pattern. Animal Cognition, 3: 99-106.

Lewis, S.E. 1995. Roost fidelity of bats: a review. Journal of Mammal, 76, 481–496.

Lianne C. Ball 2002.A Strategy for Describing and Monitoring Bat Habitat. The Journal of Wildlife Management~~-~~, Vol. 66(4): 1148-1153.

McNab, B.K. 1982. Evolutionary alternatives in the physiological ecology of bats. In *Ecology of bats* (pp. 151-200). Springer, Boston, MA.

Medellin, R.A., M. Equihua, and M.A. Amin. 2000. Bat diversity and abundance as indicators of disturbance in Neotropical rainforests. *Conservation biology*, *14*(6), pp.1666-1675.

Medina, A., C.A. Harvey, D.S. Merlo, S. Vilchez, and B. Hernandez. 2007. Bat diversity and movement in an agricultural landscape in Matiguás, Nicaragua. *Biotropica*, *39*(1), pp.120-128

Meyer, C.F., M.J. Struebig, and M.R. Willig. 2016. Responses of tropical bats to habitat fragmentation, logging, and deforestation. In *Bats in the ~~anthropocene~~Anthropocene: Conservation of bats in a changing world* (pp. 63-103). Springer, Cham.

Moreno, C.E, and G. Halffter. 2001. On the measure of sampling effort used in species accumulation curves. *Journal of Applied Ecology*, pp.487-490.

Formatted: Font color: Text 1

Formatted: Font: 12 pt, Font color: Text 1

Formatted: Font color: Text 1

Formatted: Line spacing: single

Formatted: Font: (Default) Times New Roman, Font color: Text 1

Formatted: Indent: Left: 0.07", Line spacing: single

Formatted: Font: (Default) Times New Roman, Font color: Text 1

Formatted: Font: (Default) Times New Roman, Font color: Text 1

Formatted: Font: (Default) Times New Roman, Font color: Text 1

Formatted: Font: (Default) Times New Roman, Font color: Text 1

Formatted: Font color: Text 1

Formatted: Line spacing: single

Formatted: Font: 12 pt, Font color: Text 1

Formatted: Font color: Text 1

Formatted: Line spacing: single

Formatted: Font: Times New Roman, 12 pt, Font color: Text 1

Formatted: Font color: Text 1

Formatted: Line spacing: single

Formatted: Line spacing: single

Mahandran, V.,C. M. Murugan and P. T. Nathan, 2015. An unusual roosting behaviour of Schneider's leaf-nosed bat, *Hipposideros speoris* at a cave-temple roost in Tamil Nadu. *Current Science*,109(5), pp 858:859.

Nikil.K.2020. Assessment of seasonal changes in total open water extent of tanks of Tirunelveli and Thoothukudi districts of Tamil Nadu using geomatics. Masters dissertation thesis, Pondicherry Central University, India. pp 44.

Payton, M. E., M. H. Greenstone, and N. Schenker. 2003. Overlapping confidence intervals or standard error intervals: what do they mean in terms of statistical significance? Journal of Insect Science 3:1–6.

Rainho, A., Palmeirim, J.M., 2011. The importance of distance to resources in the spatial 701 modelling of bat foraging habitat. PLoS One 6, e19227. doi:10.1371/journal.pone.0019227

Rajasegarn, P., N. Shazali, and F.A.A. Khan. 2018. Microclimate and Physiological Effects in the Roosts of Cave Dwelling Bats: Implications in Roost Selection and Conservation in Sarawak, Malaysian Borneo. *Zoological science*, *35*(6), pp.521-527.

Razgour, O., Hanmer, J. & Jones, G. ~~(~~2011.~~)~~ Using multi-scale modeling to predict habitat suitability for species of conservation concern: the grey log-eared bat. Biological Conservation, 144, 2922–2930.

Samrat.A, M. S. Devy & **T. Ganesh.** 2021. Delineating fragmented grassland patches in the tropical region using multi-seasonal synthetic aperture radar (SAR) and optical satellite images, International Journal of Remote Sensing, 42:10, 3938-3954, DOI: 10.1080/01431161.2021.1881181, IF:2.976

Selvarathinam, T, and G. Marimuthu. 2013. Roost selection and fidelity of leaf-nosed bat, *Hipposideros speoris*. International Journal Current Trends in Research, 2: 62-65

Sophia,E. 2010.Foraging behaviour of the microchiropteran bat, *Hipposideros ater* on chosen insect pests. Journal of Biopesticides 3(1 Special Issue) 068 – 073.

Sophia,E, and J.Vanitharani.2006. *Hipposideros ater* in the ecosystem of Tirunelveli, Tamilnadu. Journal of Theoretical and Experimental Biology. Vol. 3 Pp: 27-34 ISSN: 0972-9720.

Stebbings, R.E. 1995. Why should bats be protected? A challenge for conservation. *Biological Journal of the Linnean Society*, *56*(suppl_1), pp.103-118.

Suksai, P., and S. Bumrungsri. 2019. Water bodies are a critical foraging habitat for insectivorous bats in tropical agricultural landscapes of ce~~a~~ntral Thailand. *Songklanakarin Journal of Science and Technology*.

Swamidoss, P.D., M.R. Sudhakaran, and P. Parvathiraj. 2012. Habitat preference of microchiropteran bats in three districts of Tamilnadu, South India. *International Research Journal of Biological Sciences*, *1*(5), pp.24-30.

Thet.T and K. M. Mya 2015. Harvesting the guano of insectivorous bats: is it sustainable? Journal of Threatened Taxa 7(6): 7296–7297.

Treitler, J.T., Heim, O., Tschapka, M. and Jung, K. (2016), The effect of local land use and loss of forests on bats and nocturnal insects. Ecol Evol, 6: 4289-4297. https://doi.org/10.1002/ece3.2160).

Vanitharani, J., 2004. The Emerging Trends in the Bio-Diversity of Bats in Tamil Nadu. *Mapana Journal of Sciences*, *2*(2), pp.14-27.

Voigt, C.C., and T. Kingston. 2016. *Bats in the Anthropocene: conservation of bats in a changing world* (p. 606). Springer Nature.

Vonhof, M.R., and Barclay. 1996. Roost-site selection and roosting ecology of forest-dwelling bats in southern British Columbia. Canadian Journal of Zoology, 74: 1797-1805.

Williams-Guillen, K., E. Olimpi, B. Mass, P.J. Taylor, and R. Arlettaz. 2016. Bats in the anthropogenic matrix: challenges and opportunities for the conservation of Chiroptera and their ecosystem services in agricultural landscapes. In *Bats in the Anthropocene: Conservation of bats in a changing world* (pp. 151-186). Springer, Cham.

Wanger, T.C., K. Darras, S. Bumrungsri, T. Tscharntke & A.M. Klein (2014). Bat pest control contributes to food security in Thailand.Biological Conservation 171: 220–223; http://dx.doi.org/10.1016/j.biocon.2014.01.030

Formatted: Font: (Default) Times New Roman, 12 pt

Field Code Changed

Formatted: Font: (Default) Times New Roman, 12 pt

Field Code Changed

Figure

Click here to access/download;Figure;Figure 1.jpg

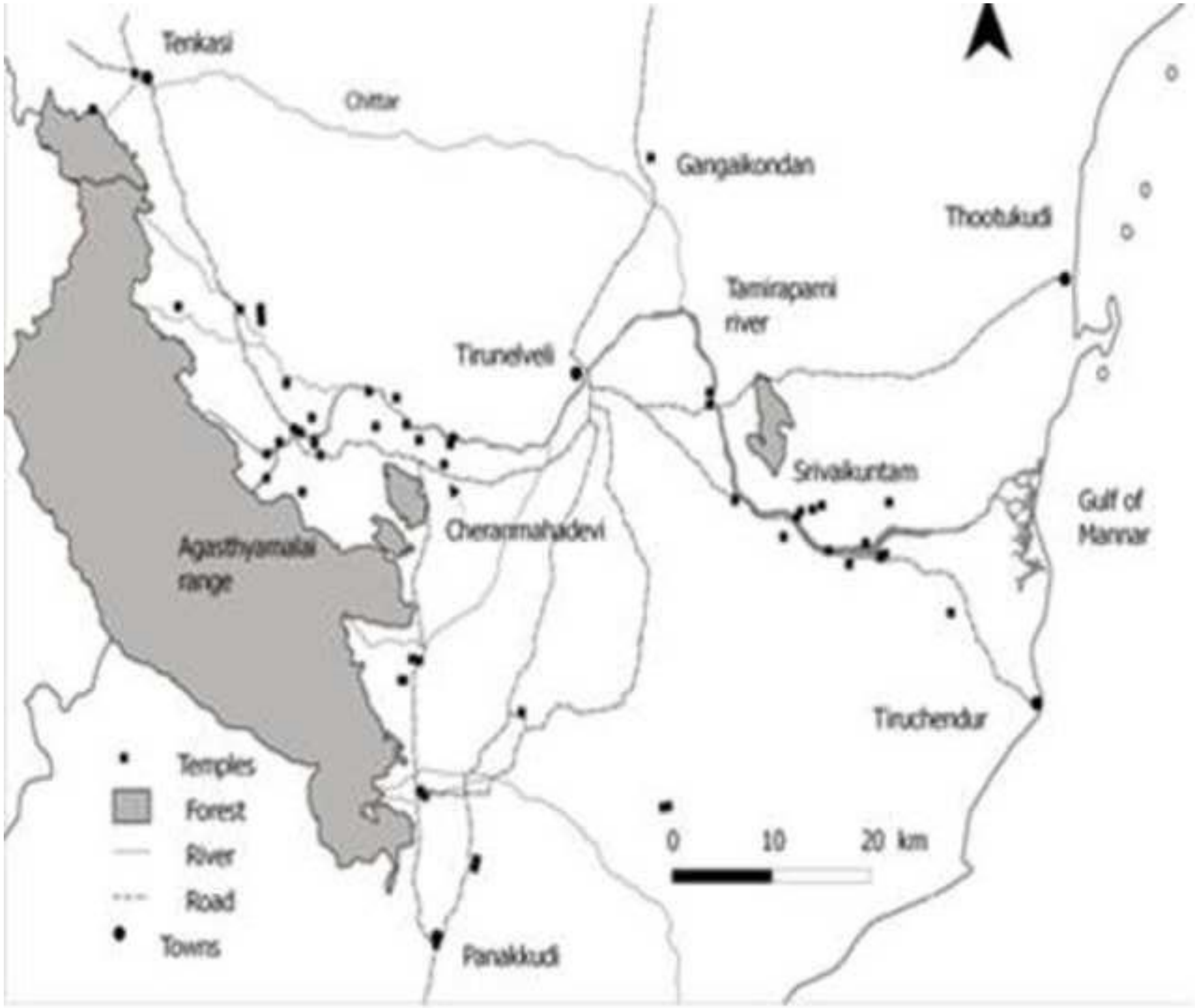

Figure2

Click here to access/download;Figure;Figure2.jpg

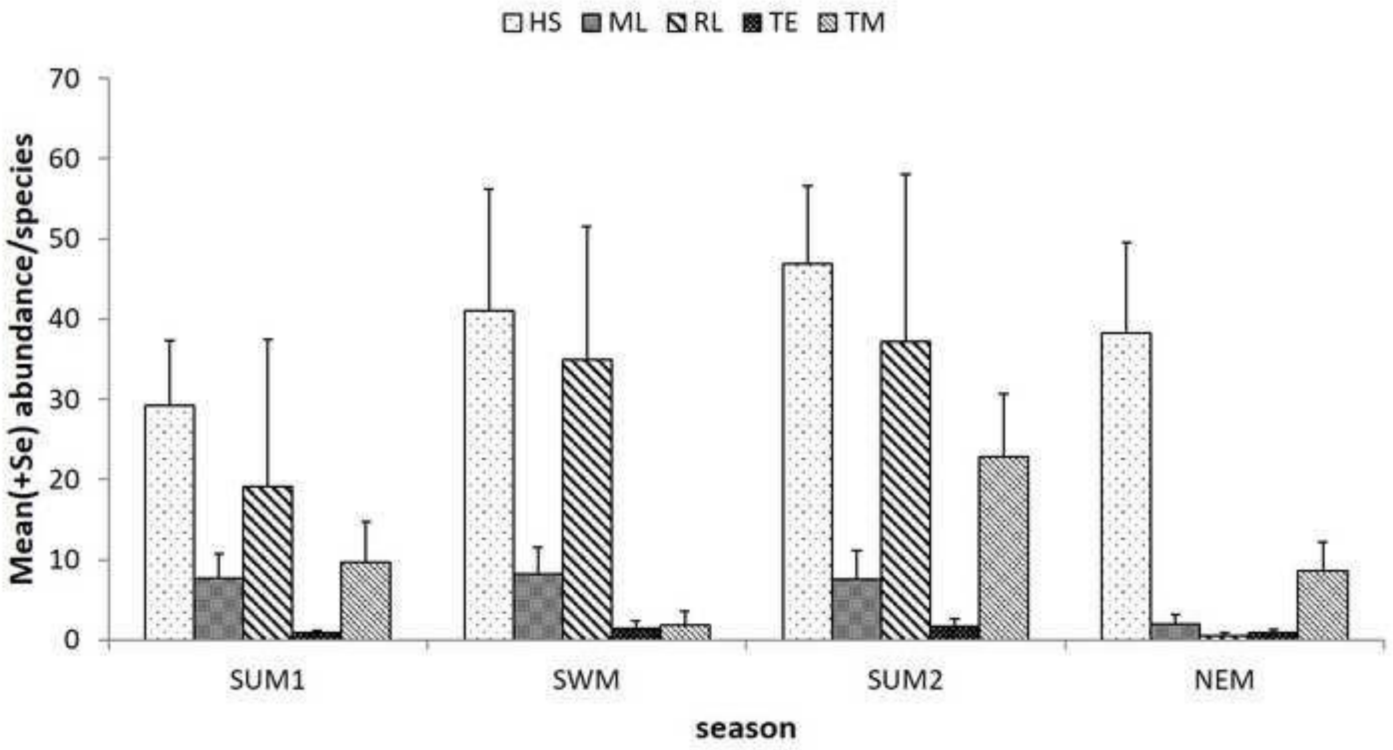

Figure3

Click here to access/download;Figure;Figure 3.jpg

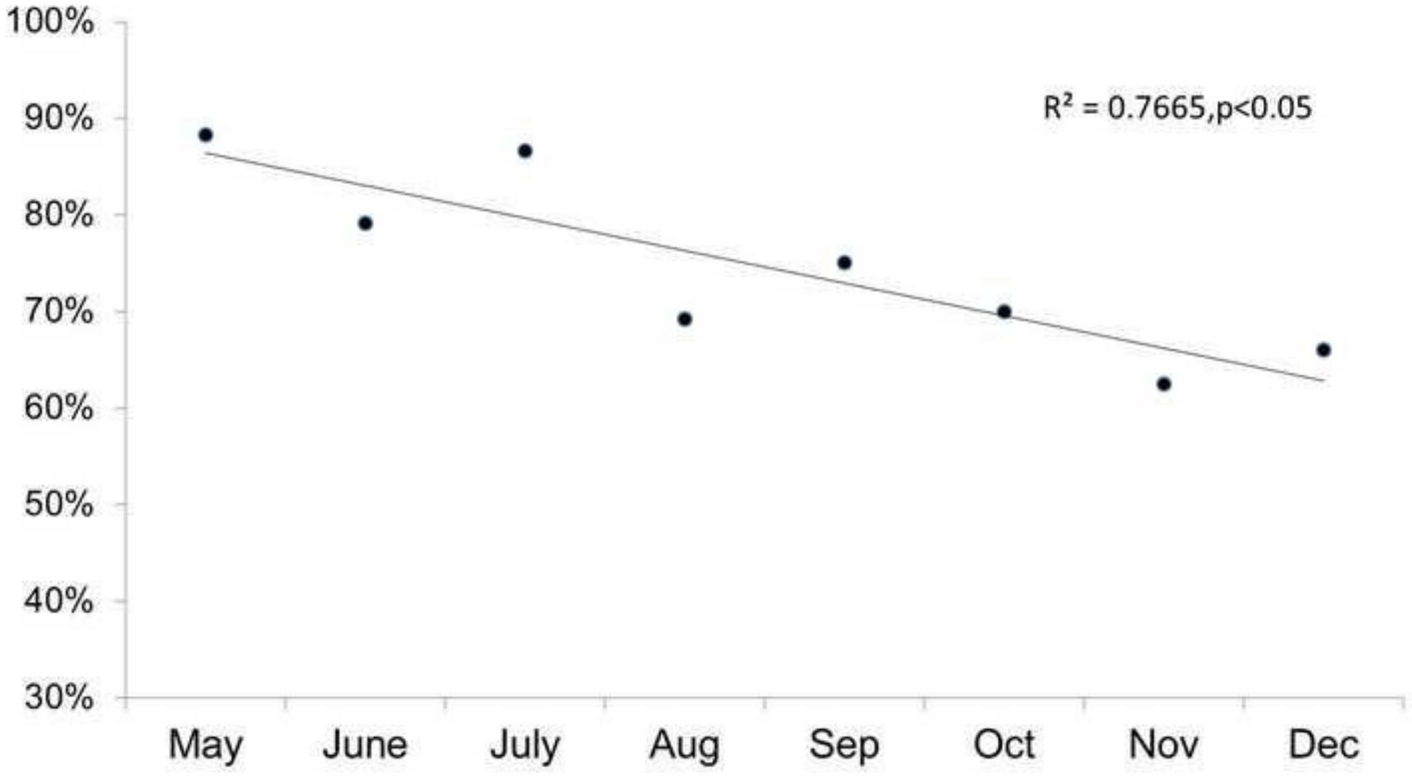

Figure4

Click here to access/download;Figure;Figure4.jpg

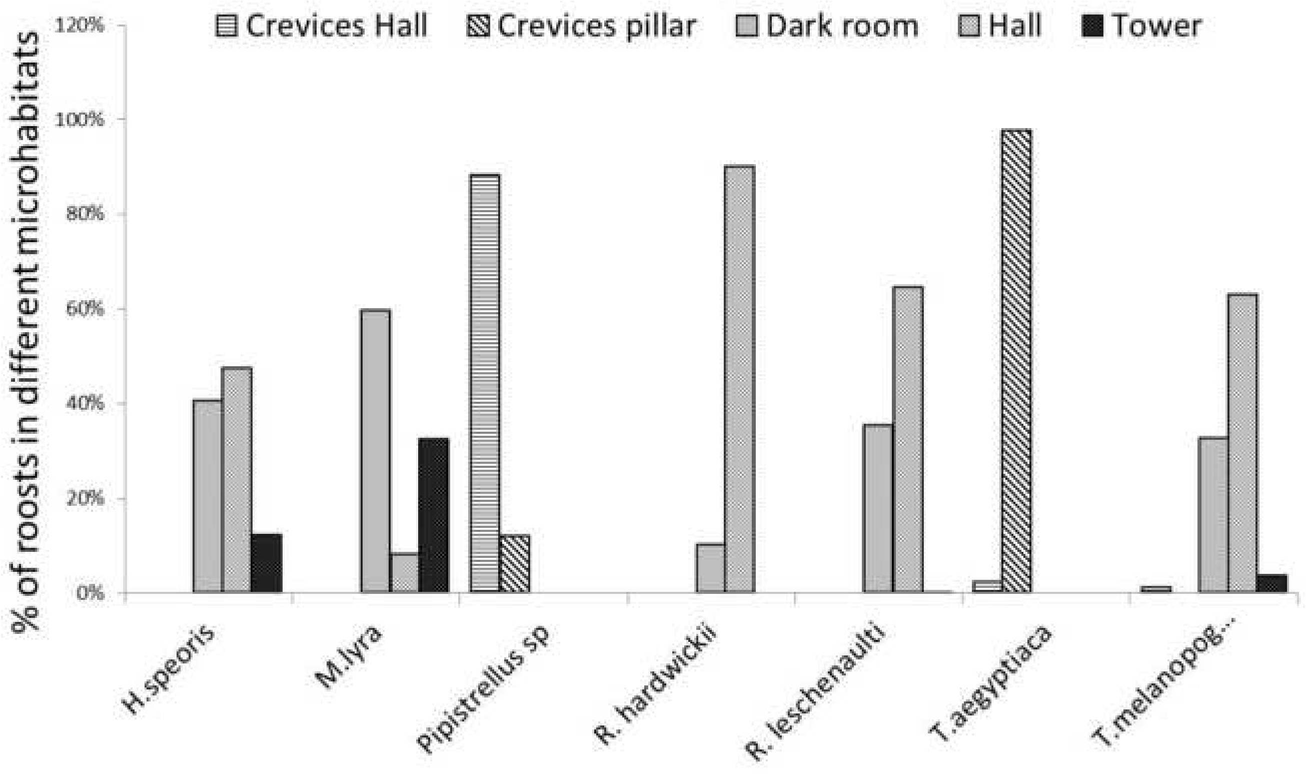

Figure5

Click here to access/download;Figure;Figure 5a.tiff

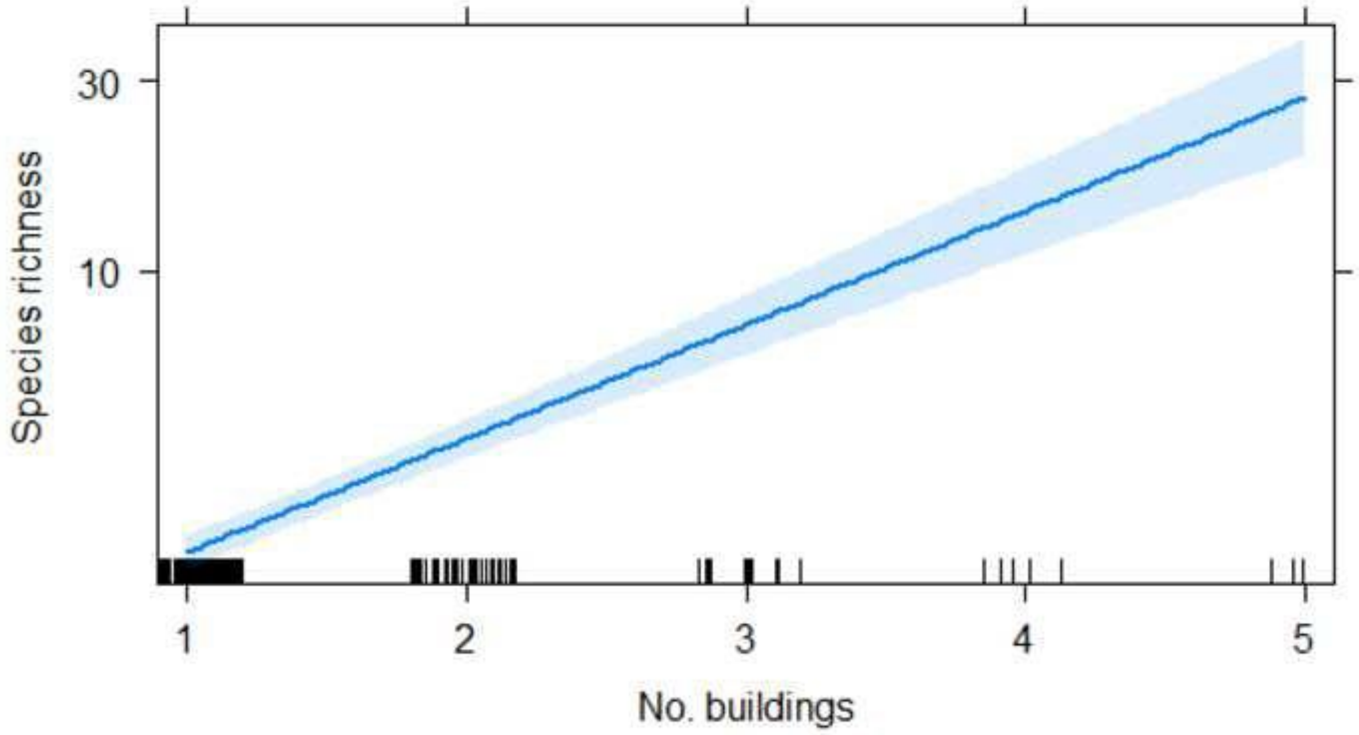

Figure5

Click here to access/download;Figure;Figure 5b.jpg

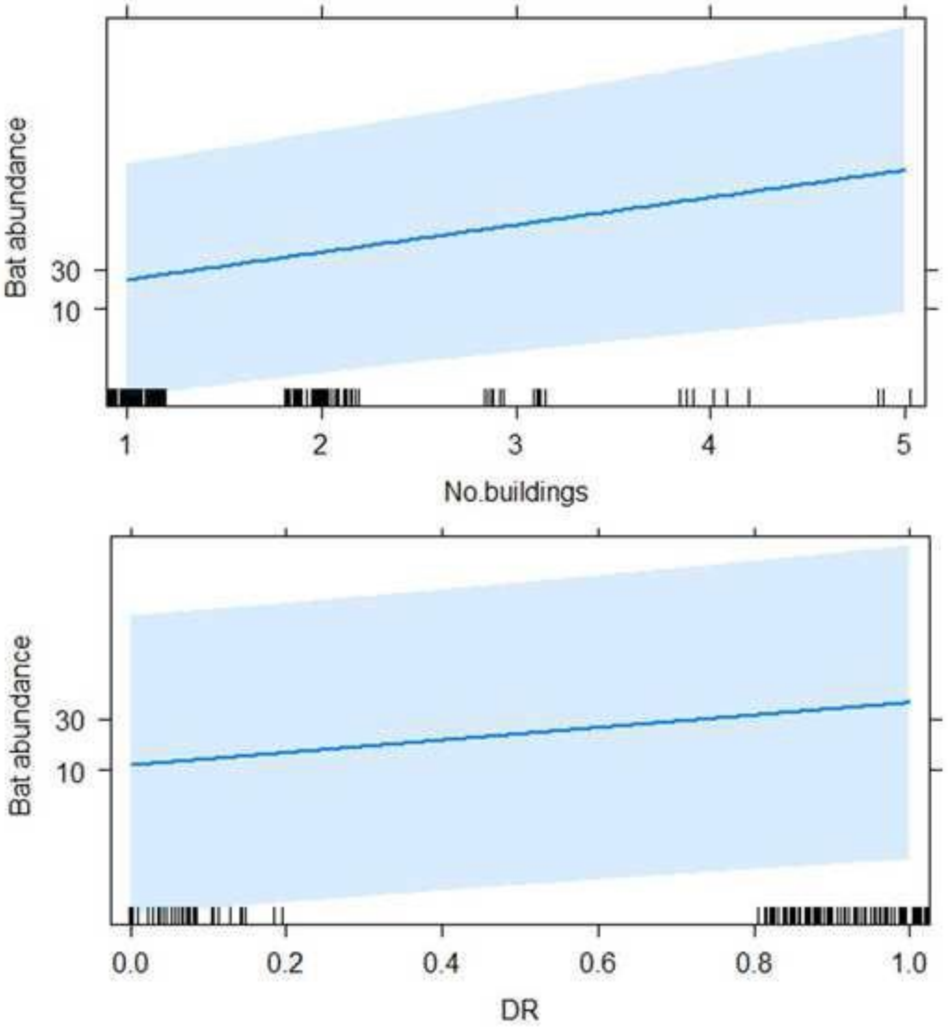

Figure6

Click here to access/download;Figure;Figure 6.tiff

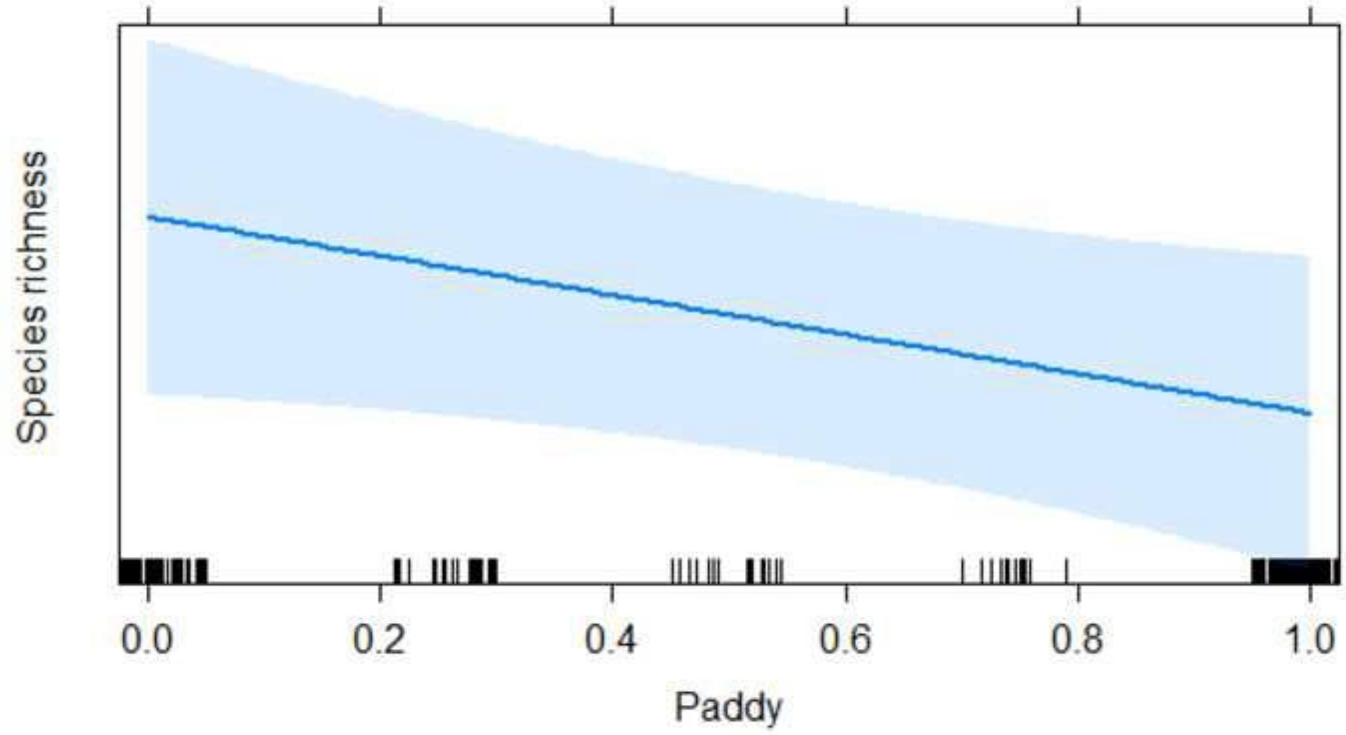

Figure7

Click here to access/download;Figure;Figure7.jpg

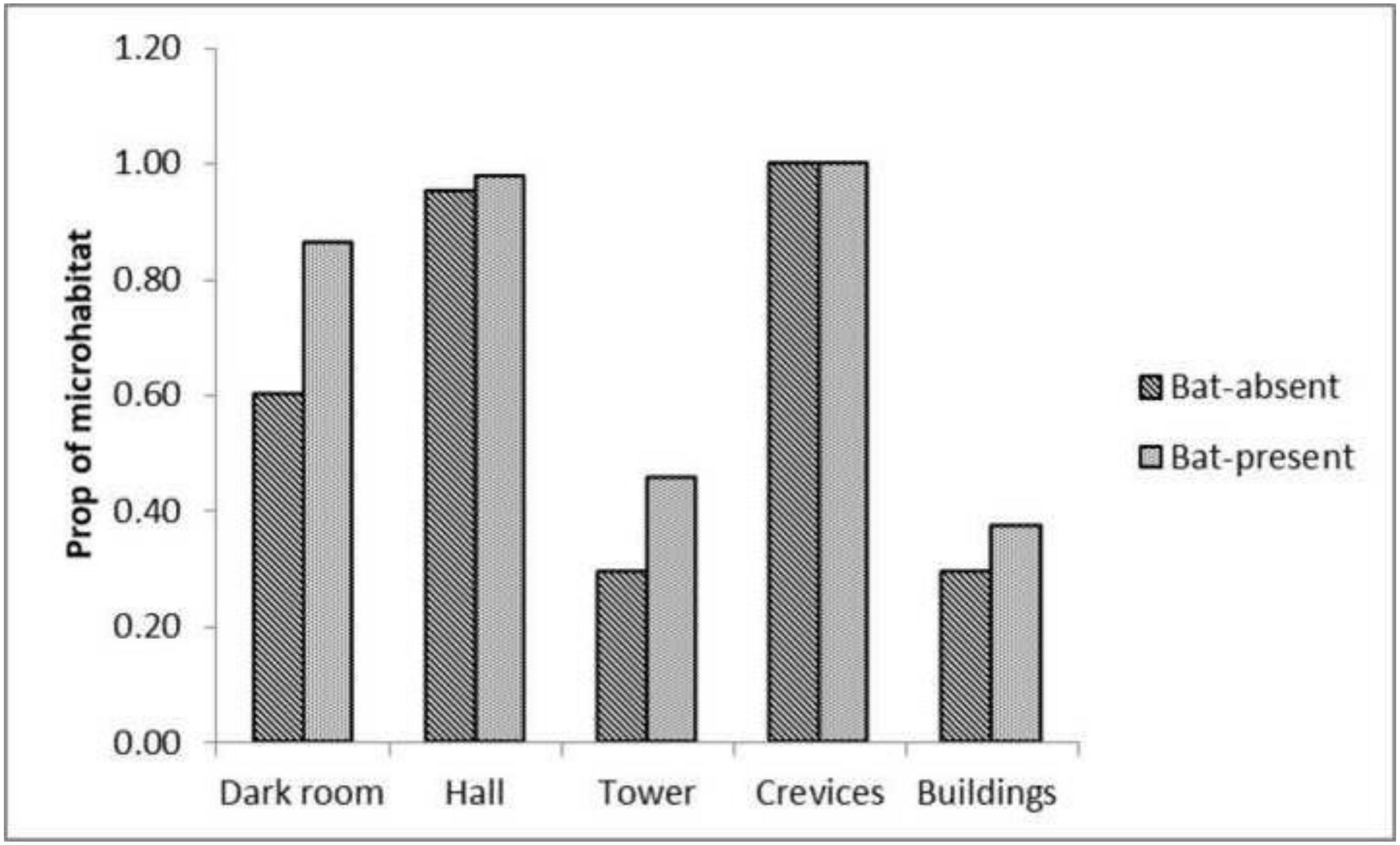

Figure8

Click here to access/download;Figure;Figure 8.jpg

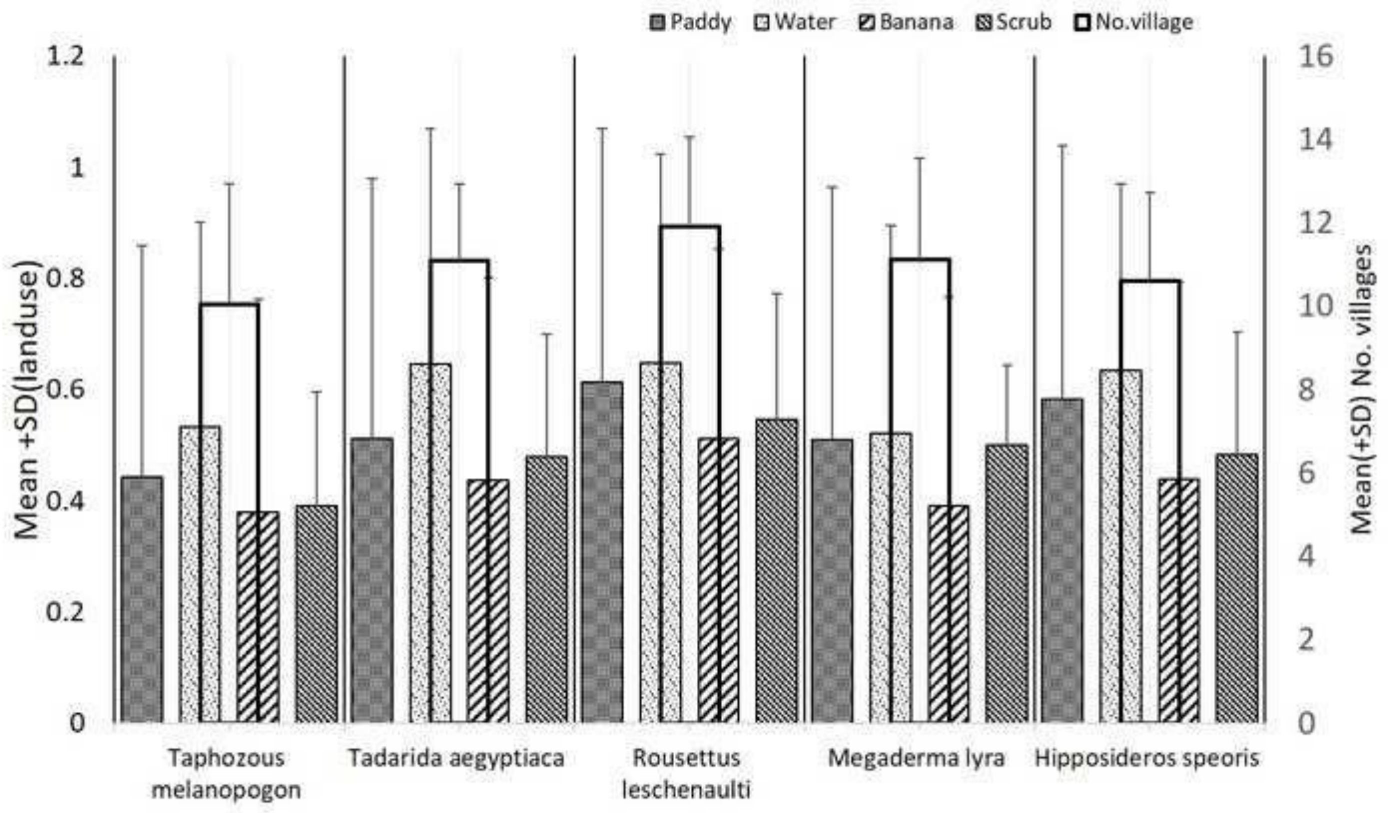

Figure legend Click here to access/download;Figure;Figure-captions.docx

Figure legends

Figure 1. Map of the study area. The filled dots represent temples with bats and empty ones without bats.

Figure 2. Percentage of microhabitats used by the different species of bats for roosting.

Figure 3. Effect plots showing the relationship between species richness, abundance and number of buildings and dark rooms in temples

Figure 4. Effects plots showing the relationship of disturbance on species richness and abundance.

Figure 5. High resolution (10m) land-use around the temples sampled. Land-use classification obtained from Karra et al 2021. In the analysis grass and bare areas, and water and flooded areas were combined. See data analysis for more details.

Figure 6.Proportional availability of habitats in temples with and without bats.

Figure 7. Mean land-use elements around temples with and without bats at various distances. * indicate significant differences at $p < 0.05$.

Figure 1. Map of the study area. The dots represents temples sampled with and without bats.

Formatted: Space Before: 4.5 pt

Figure 2. Mean abundance(x±se) of bats across seasons in temples. NEM: Northeast monsoon(Nov-Dec), SUM1 (May-June), SWM(July-Aug) and SUM2(Sep-Oct).

Figure 3. Percentage of temples occupied by bats across months.

Formatted: Indent: Left: 1", Space Before: 4.5 pt

Fig 4. Percentage of microhabitats used by the different species of bats for roosting.

Formatted: Space Before: 4.5 pt

Fig 5a. Relationship between species richness and number of buildings in temples

Fig 5b. Effects plots showing the relationship between dark rooms and number of buildings on species richness and bat abundances.

Fig 6: Effect plot of paddy availability and number of species.

Fig 7.Proportional availability of habitats in temples with and without bats.

Figure 8. Mean value of habitat variables 5km around the temples across bat species.

All responses to reviewers are marked as authors response(AR) in red.

Editor

The reviewers agree and the AE concurs that there is a degree of opacity in the analytical approaches that require elucidation before the manuscript can be fully vetted (at least with respect to the analyses, their results, and the inferences drawn from them). The reviewers identify a number of areas in the analytical methods that require additional detail and clarification.

AR: We have re written the Material and methods section giving clarity in the methods used and the data used in the analysis

the manuscript requires substantial editing to correct considerable errors in grammar and syntax.

AR: This has been addressed to the extent possible

Reviewer 1.

Review: Temples the last abode for bats in a homogeneous agriculture matrix: Importance of microhabitat availability and land use factors for bat conservation.

Ganesh et al studies historic temples as roosting habitat for bats in India. This work highlights the importance of anthropogenic structures as essential roosts, particularly as temples are places where humans are actively working to remove bats. While focusing on one region in India, the authors conducted roost counts and identified species of bats over several years while also assessing each temple for several microhabitat and landscape factors. They conducted linear models to compare species richness and abundance to the landscape and microhabitat variables and calculated niche breadth and overlap. The authors found seven species of bat across forty seven out of fifty-four temples that they surveyed. Dark rooms and corridors were found to be the most important microhabitats for roosting, while landscape effects had little overall effect albeit with some species-specific instances. Bat abundance varied by species in season, but overall was lowest during the monsoon season. There was little species overlap in temples and little niche overlap. The authors attributed a lot of the microhabitat preferences to species-specific life history and roosting preferences. The seasonality differences correlated with the parturition and lactation of many of the species. While the landscape effect had a weak response, the importance of scrub and open landscape is still noted. Overall, this paper lays the foundation for further studies and emphasizes the importance of an anthropogenic roost for bats. This paper certainly merits publication with additional clarification and editing.

Formatted: Font: Bold

Formatted: Font color: Red

Formatted: Font: Not Bold

Formatted: Normal

Formatted: Font: (Default) +Body (Calibri), 12 pt

Formatted: Font: (Default) +Body (Calibri), 12 pt, Not Bold

Formatted: Font: Bold

Formatted: Indent: Left: 0", First line: 0", Space After: 8 pt

My first concern overall for the paper is some grammatical and formatting issues. I have listed specific examples of reoccurring problems throughout at the end of this review, however there are additional grammar edits that need to be fixed.

My second concern is with the landscape ecology analysis and discussion throughout the paper. The analysis of the landscape features was measuring presence/absence of landscape features over a significantly sized area. This broad measuring does not get at some of the heterogeneity of the landscape and may explain some of the lack of effect of the landscape features in the model. A more rigorous measurement of the landscape types could be better such as using the total amount or percentage of land cover type in the 5km buffer. As the remote sensing data may be unable to provide this level of detail (as mentioned in the methods), a mention of this drawback in the discussion would be warranted. Additionally, the discussion section touched on the landscape feature briefly but the middle of the paragraph still discussed temple characteristics such as festivals and renovations and the ending sentences focused on site fidelity. The importance of open spaces was stated but never explained or elaborated on. A deeper discussion with a paragraph focused solely on landscape characteristics while moving the still-relevant topics to more appropriate places would improve the analysis especially since the third hypothesis on landscape features was disproven.

Overall, this paper has significant importance for publishing by emphasizing the importance of temples as habitat worthy of conservation especially at a time when bats are at a high risk of exclusion from places because of the pandemic. Some modifications are necessary to clarify the paper and its findings, however they do not detract from the importance of the study.

Authors response(AR):

The landscape analysis has been completely revised. We have used recently released ESA landcover classes at 10m resolution for the analysis (https://tiledimageservices.arcgis.com/P3ePLMYs2RVChkJx/arcgis/rest/services/Esri_2020_Land_Cover_V2/ImageServer). However, this approach allows us to estimate the landcover class for a particular period and not across seasons because of cloud cover as mentioned in the MS earlier. We have therefore assumed, based on government literature that landcover has not drastically changed during the study period which is also substaintiated by authors observations. We analysis the % landcover under each class to species richness and abundance of bats at 3 spatial scales;500m, 1km and 3km. The variable in the landcover class is crop which we determine based on google earth images as earlier and interviews with farmers around temples. Some periods of the sampling May and Sep do not have any crops and there fore we have considered crop area in such times as zero and considered it as fallow agriculture. More details in the MS.

Reviewers: A deeper discussion with a paragraph focused solely on landscape characteristics while moving the still-relevant topics to more appropriate places would improve the analysis especially since the third hypothesis on landscape features was disproven.

AR: Our discussion has changed and improved given the changes in the analysis. We have as suggested given more space for discussing landscape characterstics.

Formatted: Not Highlight

Formatted: Not Highlight

Formatted: Font color: Red

Formatted: Font color: Red

Formatted: Font color: Red

Formatted: Font color: Red

Formatted: Not Highlight

Formatted: Not Highlight

Formatted: Font color: Red

Grammatical and Formatting Comments:

Check the spacing throughout the article. There should be a space in between the last word and opening parentheses. On the last sentence of page 11, there should be a space after the comma and between each year. The same issues occur in the caption for Table 4 with the list of symbols. Lastly, there should be a space in between genus and species in the species names in the results - I.e. M. lyra, not M.lyra.

In the first two paragraphs of the introduction, there are a few words and several citations where the year is in blue. They should all be in black since they are not hyperlinks.

A w is bolded in the word "with" in the beginning of the full paragraph on page 10.

Throughout the entire paper, halls is followed by (corridors) or vice versa each time. I think saying "halls and corridors" is appropriate the first time and then use one consistently for the rest of the paper.

AR: We agree on the English and have tried our best to improve. Some of the issues have also arised due to formatting.

We have modified halls ~~it~~ to a more appropriate word 'walkway'

Formatted: Font color: Red

Formatted: Font color: Red

Figure 1 appears blurry, so I would recommend the resolution to be larger. Partially because of the resolution, the temples and town dots are hard to tell apart. It would be easier to read if one of the dots was hollow.

AR: We have redone the figure

Formatted: Font color: Red

Figure 3 does not have axis labels which helps the reader.

Corrected

Formatted: Font color: Red

In Figure 4, the dark room and hall patterns are very hard to tell apart between the bars. A different pattern would make the graph easier to read.

We have redone the figure

Formatted: Font color: Red

Figure 5 has extra ticks around the outside of the graph.

We have redone the figure

Formatted: Font color: Red

In Figure 8, the no-village bar is distracting and confusing as it overlaps with the error bars.

Changed the fig as we are using landcover maps

Formatted: Font color: Red

Reviewer 2

Reviewer Summary of Paper: This is a study of factors important for bats using ancient temples and overlap of roost use in bats using ancient temples. The authors investigate roost characteristics of temples (microhabitat) and surrounding land use (landscape-scale habitat at 5 km scale). They determine that roost characteristics (mainly presence of dark rooms and halls) influence bat richness/abundance in temples. Landscape variables at the 5 km scale were not important for bat abundance and bat presence.

Overall impression: The subject of managing historic/ancient buildings and bat conservation will appeal to many readers. The study would add to the literature of managing ancient/historic buildings for bats. However, the manuscript would need major revision before publication in PLoS One. The discussion is the strongest section of this paper, and other sections could be improved by incorporating more literature into the introduction to set up the hypotheses, clarifying the hypotheses, organizing the data analysis section by hypothesis, adding more information about which data were used in which analysis, and clarifying some of the figures.

My specific comments to the authors to improve the manuscript are outlined below:

Title:

1. The term "matrix" implies a heterogenous landscape of multiple land use/cover types not a homogenous one (only one cover type). I suggest rewording or clarifying the title.

   Author response AR: replaced "matrix" with "landscape"

Formatted: Font: Bold

Formatted: Heading 2

Formatted: Indent: Left: 0.75", No bullets or numbering, Tab stops: Not at 0.5"

Abstract:

2. Please specify which pandemic.
3. AR: Mentioned as COVID19 pandemic

4. "*We sampled 59 temples repeatedly across 5 years which yielded a sample of 256 temple*." I believe that 59 temples sampled repeatedly is a sample size of 59 temples. I suggest saying "which yielded 256 survey events" or rephrasing another way. Was every temple sampled 5 times?

   AR:Yes sample size is 59 temples and theses were repeatedly sampled. Suitably modified in the abstract. The number of times each temple was sampled varied between 1 to 5. Have mentioned the methods.

Formatted: Font: (Default) Times New Roman, 12 pt

Formatted: Indent: Left: 0.5", No bullets or numbering, Tab stops: Not at 0.5"

Formatted: Indent: Left: 0.75", No bullets or numbering, Tab stops: Not at 0.5"

Introduction:

The introduction should be supported with more literature to justify the research question. I suggest the authors incorporate the literature on bats using anthropogenic structures and introduce and explain the issues here for bats, humans managing structures, and bat-human conflicts. A good paper to read is Fagan et al. 2018. "Roost selection by bats in buildings, Great

Smoky Mountains National Park” in the Journal of Wildlife Management 82: 424-434, as they evaluated bat roost selection of historic buildings in a non-urbanized area and also measured landscape variables. If the literature is limited for bats in buildings, the authors could draw on the literature of bats using bridges or other anthropogenic transportation structures that humans have to manage. One example is Meierhofer et al. 2019. “Structural and environmental predictors of presence and abundance of tri-colored bats in Texas culverts” in the Journal of Mammalogy 100: 1274-1281. One of the more interesting aspects of this subject is that bats using historical buildings or other human infrastructure makes it difficult to balance bat conservation with human-bat conflicts. And this could be applied beyond ancient temples.

More information on the types of bat species that use these temples, why we should care about them, and current conservation status would greatly improve this manuscript. For example, the authors could mention the importance of bats to agricultural pest suppression (if these bats are insectivores) or seed dispersal (if these bats are frugivores) or providing other examples of ecosystem services. The authors should explain what they mean by the pandemic negatively affecting bats. Future readers may not know which pandemic the authors are referring to, and why it affected bats. If any species are “data deficient” or of conservation concern on the IUCN Red List of Threatened Species (https://www.iucnredlist.org/), this should also be mentioned in this introduction. Additionally, if there have been previous studies involving these species, i.e., what they usually use to roost if not temples, what they eat, where they forage, etc. this would be helpful to include some basic background information to set up the hypotheses. Are they mostly fruit bats or are they insectivores? Some species background information is provided in the discussion but is really needed in the introduction to support the justification of these research questions.

Similarly, more information on temple structure, e.g., What materials are they made out of? How do bats get into the temples (through doors or cracks in the walls or unknown?) Are they air conditioned or are they about the same temperature/hotter/cooler than outside?) Additionally, introduce readers to the monsoon seasons briefly in the introduction and more in the study area section.

AR: More information on temple structure given in the MS itself

The authors should be careful about using the term “niche” when only referring to roosting habitat. A niche encompasses all needs of a species (roosting habitat, foraging habitat, all biotic and abiotic factors (climate), etc.). In some parts of this manuscript, I believe “niche” should be replaced with “roost habitat”.

Formatted: Not Highlight

The objectives of this paper need to be clarified if they are investigating what microhabitat and landscape factors determine roost use and bat abundance in order to inform bat conservation in ancient temples, and/or if they are investigating which bats have roost overlap needs (what I think the authors are referring to when they say “niche overlap and breadth”). What is important about quantifying niche overlap/breadth?

AR: we have removed niche analysis from the results.

Formatted: Font color: Red

5. "*These are situated in a human-dominated agricultural matrix that is changing due to several economic reasons and climate change effects (Fischer et al. 2009, 2010; Jones et al. 2009).*" Please describe the climate change effects affecting bats in this area. Do you mean the monsoons? I also suggest including literature on anthropogenic disturbance, agriculture, and habitat/fragmentation threats to support your justification for this research. I suggest developing those ideas more with Frick et al. 2019 "A review of the major threats and challenges to global bat conservation" paper (available at: https://www.researchgate.net/publication/332146982_A_review_of_the_major_threats_and_challenges_to_global_bat_conservation) or other studies

AR: The role of climate and habitat transformation in the region has been stated in the context of Frick et al 2019 paper. We have also added some available literature for the region.

6. "*The recent pandemic has further made them more vulnerable to disturbance and direct persecution.*" Please describe which pandemic and why this is an issue for bats.
   AR: COVID 19, Done

7. " 1. Given that the temple bats are mostly cave-dwelling species we expect larger Yinpterochiroptera occupying similar micro-habitat, show greater niche overlap and similar niche breadth." Please introduce the species before stating the hypotheses. This is the first time the bats and some traits are mentioned. Please describe niche breadth and overlap in this context. Does this mean the authors are trying to determine overlap in roost needs/preferences of species using the temples and that they expect Yinpterochiroptera to have overlapping needs with each other? Why does this matter? Please clarify this hypothesis. Are Yinopterochiroptera the only group that use the temples? Are they all cave-dwelling?

AR: we have modified this hypothesis. We have removed niche overlap from the paper. Mention of species is done earlier and most of the species are insectivorous expect for one

8. "2. Microclimate conditions inside the temples are very stable and any influence on bat abundances could be a reflection of food availability or disturbance at roost and therefore we expect bats to show seasonal changes in niche overlap and breadth due to variability in crop and insect availability."

This hypothesis is confusing to me. I'm not sure what you are hypothesizing. Would it not be better to test abundance changes seasonally rather than niche overlap and breadth? The climate/monsoon seasons could also be described before introducing this hypothesis. Additionally, it is unclear how disturbance at roost and food availability were measured.

AR: we have modified this hypothesis and have made a separate result for roost disturbance which is mentioned in the introduction and methods.

9. *"3. Since the structure of ancient temples are all similar, we do not expect any change in microhabitat availability in temples with and without bats, instead we expect bat abundances driven by the availability of landscape features especially seasonal crops around the temples."* Please describe how the temples differ or are similar to each other and the seasonality of crops before introducing this hypothesis. Also, is it not true that the temples are structurally different? Some are smaller/larger and more complex than others?

AR:We have described the temples in detail and the type of crop and their availability in the section. Temple did vary in size but structurally they were more or less similar.

Materials and methods:

"The ancient temples(>400 years) are built of large granite stones and provide a niche for several bat species." I think the authors mean that the temple structures provide roost habitat, not a full niche.

AR: Agreed and we have clarified in the text

"The surveyed temples are situated in an agricultural matrix surrounded by rice paddies or bananas" Are the temples completely surrounding in the whole 5 km radius landscape or just mostly surrounded in the immediate area around the temple? What is the extent of the surrounding agriculture? An agricultural matrix implies mixed cover types so this sentence is somewhat confusing.

AR: The entire area even beyond 5km are dominated by paddy. We have given more information about the landscape in the introduction and study area section

"stone pillars that supported the stone roof was considered a separate habitat as they provided a niche for few species that roost in the gaps created between the stone joints this was categorized as crevices" This sentence is a little confusing as it reads. Were the stone joints in the pillars categorized as crevices? The stone pillars provided a roost, not a niche.

AR: Yes, they provide crevices and therefore a roost habitat

"In 2012 we measured temperature and humidity while sampling for bats and found no difference between temples and therefore not considered in the analysis." What are the temperature and humidity conditions in these temples and is it similar to cave conditions used by these bats?

AR: We are not aware of temperature and humidity conditions inside caves in the region as caves are non-existent because the rocky hills have been quarried. Conditions inside the temples are mentioned though, as stated in the methods section, we could not monitor these continuously

"Since we did not have information on how far the different bat species forage in the landscape we took 5 km based on the study on the large bat Megaderma lyra (Audet et al 1991)." Did the authors consider testing multiple spatial scales? Many bat studies have

determined that landscape scale factors are important at different scales. See the discussion in Bellamy et al. 2013 "Multiscale, presence-only habitat suitability models: fine resolution maps for eight bat species" in the Journal of Applied Ecology 50: 892-901 (available at: https://besjournals.onlinelibrary.wiley.com/doi/full/10.1111/1365-2664.12117) about importance of factors at multiple landscape scales. In the case of not knowing foraging distance for all species, it would improve this study to quantify landscape availability at more than one scale to determine if it is important (For example, maybe test 500 m, 1 km, and 5 km or landscape scales that are relevant to bats in other parts of the world). What kind of bat is the *Megaderma lyra*? Is it an insectivorous species?

AR: Thank you for the paper link. We have used one-time remote sensing classification of the land use available at ESA website and reanalyzed the data at 4 different spatial scales as suggested. Since such information was available only at one particular year for the entire region we considered the land use not to have changed during the sampling period and when the image was taken. This we justify based on government records of area under agriculture. We also tried to do a fine level classification by gridding the area around temples and finding the land use within the grid cells using google earth. This was a herculean task as the grid cells for each temple at 3km was over 1000 and was not possible to do that for each temple across years and is the reason we resorted to remote sensing analysis for fine scale analysis

.

AR: Megaderma lyra is an insectivorous species and we have mentioned the details of all the species in a separate Table 1..

"If a land use element is present we gave a value of 0.25 in each quadrant and calculated the frequency of each land use for the entire 5km radius area." Is 25% (i.e, 0.25) sensitive enough to detect variation? Why 25% and not 10%? If a 5 km radius landscape only has a little bit of banana, but it is present, then it would still get a value comprising 25% of the landscape? This may be a limitation of the study to mention in your discussion. Would the results be different if the 5 km landscape was divided by 10% increments?

AR: This is a very valid point and we agree on the bias and as mentioned above we have done our best under the current conditions. Keeping all the comments related to landuse we have changed the analysis as mentioned before.

Did land use around each temple differ or was it correlated among the 5 sampling years?

AR: Yes, the land use did not change much across years

"The best model was selected using." Please finish this sentence and indicate what AICc weights and delta AIC value criteria were used to determine plausible models.

AR: done

"We constructed a linear model to explain the species richness and abundance of bats in the temples based on the microhabitat and landscape features. We based the variables used in our linear models on our observations made in 2012." Does this mean that only the landscape features from 2012 and the bat counts from 2012 were used? I think it would be statistically incorrect if all survey years at the same temples were treated as independent temples in the models.

AR:We have use mixed model approached to repeated sampling of temples across years following Bolker et al. 2009 wherein they say "Repeated measurements on the same individual, the same location, or observations taken at the same point in time are often correlated; this correlation can be accounted for by using random effects in generalized linear mixed models". Since there were variations both within the sampled temples and across years, making temple and years as random factors accounts for this variation and gives a more robust relationship between the response variable (species richness and abundance of bats) with explanatory variables like temple features and land use elements.

Please organize the data analysis section to be more clear which analysis you are doing to answer which question/hypothesis and clarify which data were used to examine microhabitat and landscape factor influence on abundance and richness. In other words, did you use only data from 2012 or did you treat each repeated survey as a separate "temple"? If only used 2012 data, what were the monsoon conditions at that time?

AR: Temples were sampled for its features in 2012 and if any new temples were added in later years their features was recorded then. Temple features are subjected to modifications and therefore we have included renovation of temples and visitors to the temple as variables that could affect bats. Renovation is a dynamic variable that can differ between years and therefore we have measured it in all sampled years in all temples compared to more static ones like number of buildings, tower etc which do not change across years.
We have now tried to bring in more clarity in the data analysis section as suggested

Results:

"The total number of roosts sampled was 351 across 246 temples in 5 years." This sentence is a little confusing. There were only 59 temples. Were any of the roosts that were repeatedly sampled the same roosts as previous years?

AR: We have modified the sentence and yes, same roosts were sampled across years and some temples also had multiple roosts

"None of these bat species is threatened as per IUCN category." Are any of them considered "data deficient"? Please move this species information to the introduction to introduce the species that use the temples.
AR: Done. No bat was data deficient

"The lmer model explained 67% of the variation in species richness of which 55% were explained by the fixed factors." Do you mean the model averaged mixed model comprising all models with AICc <5 or the top model or something else?
AR: The % values pertain to the top model. The models have changed after reanalysis. The delta AICc was kept at <2 unless mentioned specifically. We have corrected for any discrepancies

Discussion:

The authors are effective at relating the results to the previous literature and this is the strongest section of the paper.

Mention the limitations of the study (e.g., temples were chosen based on logistics rather than randomly, only one landscape scale was considered, land use in the surrounding 5 km radius was considered at a course resolution (0.25))

AR: The landscape section has been reanalyzed completely as mentioned earlier

From your results, are their certain species that may be more impacted by temple renovation/disturbance?

AR: We have now made renovation as an objective as mentioned earlier and identify species that are affected, most species are.

Figures:

Fig 1 map is a bit blurry. I suggest increasing dpi/resolution. Did there appear to be any spatial pattern of temples that did not have bats vs. those that did? If so, mention in discussion.

AR: The fig has been redone. We did not test for spatial correlations

Fig 4 bar graph gray colors are difficult to differentiate between dark room and hall.

AR: Corrected

Fig 5 is this a bar graph of all temples, including the ones that were resampled? If so, perhaps plot the average species richness across the 5 years against the no. of buildings in each temple. It is difficult to interpret as is.

AR: These are not bar graphs, they are effect plots from the mixed model showing the relationship between number of buildings and species richness keeping other factors constant in the model. Such effect plots are routinely used to plot the results of mixed models and are considered valid to predict the relationships between variables.

We have corrected the graph and included datapoints along with the prediction line.

Same comment as last figure (which is also labeled Fig 5. Is that intentional?) What is DR? Define in figure or in parentheses in caption.

AR: expanded and clarified

Fig 6 I suggest the axis title say "Proportion of rice paddy in 5 km radius" or similar

AR: After the new analysis using remote sensing categorization we could not do the analysis at individual crop level so this is excluded

Fig 8 This figure is difficult to read with No. villages plotted on top of the land use classes.

AR: Changed completely

Table 1: Nicely presented. Please indicate in the caption at what values are there considered an overlap? Higher values mean more overlap?

AR: We have removed this analysis

Table 4: Missing a variable after the "+" under Hiposederous speoris? Did you mention that you also had species-specific models in the main text?

AR: Modified

Reviewer 3

Made comments in the MS itself which is addressed therein

Formatted: Tab stops: 1.65", Left
