# Supplementary material for: Temples and bats in a homogeneous agriculture landscape: Importance of microhabitat availability, disturbance and land use for bat conservation
Source: PLoS One. 2022 Jul 13;17(7):e0251771. doi: 10.1371/journal.pone.0251771 (PMC9278754; doi:10.1371/journal.pone.0251771)
Supplement: Attachment — Submitted filename: Review_bats_temples.docx [file pone.0251771.s003.docx]

**Reviewer Summary of Paper:** This is a study of factors important for bats using ancient temples and overlap of roost use in bats using ancient temples. The authors investigate roost characteristics of temples (microhabitat) and surrounding land use (landscape-scale habitat at 5 km scale). They determine that roost characteristics (mainly presence of dark rooms and halls) influence bat richness/abundance in temples. Landscape variables at the 5 km scale were not important for bat abundance and bat presence.

**Overall impression:** The subject of managing historic/ancient buildings and bat conservation will appeal to many readers. The study would add to the literature of managing ancient/historic buildings for bats. However, the manuscript would need major revision before publication in PLoS One. The discussion is the strongest section of this paper, and other sections could be improved by incorporating more literature into the introduction to set up the hypotheses, clarifying the hypotheses, organizing the data analysis section by hypothesis, adding more information about which data were used in which analysis, and clarifying some of the figures.

My specific comments to the authors to improve the manuscript are outlined below:

**Title:**

1. The term “matrix” implies a heterogenous landscape of multiple land use/cover types not a homogenous one (only one cover type). I suggest rewording or clarifying the title.

**Abstract:**

2. Please specify which pandemic.
3. “*We sampled 59 temples repeatedly across 5 years which yielded a sample of 256 temple*.” I believe that 59 temples sampled repeatedly is a sample size of 59 temples. I suggest saying “which yielded 256 survey events” or rephrasing another way. Was every temple sampled 5 times?

**Introduction:**

The introduction should be supported with more literature to justify the research question. I suggest the authors incorporate the literature on bats using anthropogenic structures and introduce and explain the issues here for bats, humans managing structures, and bat-human conflicts. A good paper to read is Fagan et al. 2018. “Roost selection by bats in buildings, Great Smoky Mountains National Park” in the Journal of Wildlife Management 82: 424-434, as they evaluated bat roost selection of historic buildings in a non-urbanized area and also measured landscape variables. If the literature is limited for bats in buildings, the authors could draw on the literature of bats using bridges or other anthropogenic transportation structures that humans have to manage. One example is Meierhofer et al. 2019. “Structural and environmental predictors of presence and abundance of tri-colored bats in Texas culverts” in the Journal of Mammalogy 100: 1274-1281. One of the more interesting aspects of this subject is that bats using historical buildings or other human infrastructure makes it difficult to balance bat conservation with human-bat conflicts. And this could be applied beyond ancient temples.

More information on the types of bat species that use these temples, why we should care about them, and current conservation status would greatly improve this manuscript. For example, the authors could mention the importance of bats to agricultural pest suppression (if these bats are insectivores) or seed dispersal (if these bats are frugivores) or providing other examples of ecosystem services. The authors should explain what they mean by the pandemic negatively affecting bats. Future readers may not know which pandemic the authors are referring to, and why it affected bats. If any species are “data deficient” or of conservation concern on the IUCN Red List of Threatened Species (https://www.iucnredlist.org/), this should also be mentioned in this introduction. Additionally, if there have been previous studies involving these species, i.e., what they usually use to roost if not temples, what they eat, where they forage, etc. this would be helpful to include some basic background information to set up the hypotheses. Are they mostly fruit bats or are they insectivores? Some species background information is provided in the discussion but is really needed in the introduction to support the justification of these research questions. Similarly, more information on temple structure, e.g., What materials are they made out of? How do bats get into the temples (through doors or cracks in the walls or unknown?) Are they air conditioned or are they about the same temperature/hotter/cooler than outside?) Additionally, introduce readers to the monsoon seasons briefly in the introduction and more in the study area section. The authors should be careful about using the term “niche” when only referring to roosting habitat. A niche encompasses all needs of a species (roosting habitat, foraging habitat, all biotic and abiotic factors (climate), etc.). In some parts of this manuscript, I believe “niche” should be replaced with “roost habitat”. The objectives of this paper need to be clarified if they are investigating what microhabitat and landscape factors determine roost use and bat abundance in order to inform bat conservation in ancient temples, and/or if they are investigating which bats have roost overlap needs (what I think the authors are referring to when they say “niche overlap and breadth”). What is important about quantifying niche overlap/breadth?

4. “*These are situated in a human-dominated agricultural matrix that is changing due to several economic reasons and climate change effects (Fischer et al. 2009, 2010; Jones et al. 2009).”* Please describe the climate change effects affecting bats in this area. Do you mean the monsoons? I also suggest including literature on anthropogenic disturbance, agriculture, and habitat/fragmentation threats to support your justification for this research. I suggest developing those ideas more with Frick et al. 2019 “A review of the major threats and challenges to global bat conservation” paper (available at: <https://www.researchgate.net/publication/332146982_A_review_of_the_major_threats_and_challenges_to_global_bat_conservation> ) or other studies
5. “*The recent pandemic has further made them more vulnerable to disturbance and direct persecution.”* Please describe which pandemic and why this is an issue for bats.
6. *“ 1. Given that the temple bats are mostly cave-dwelling species we expect larger*

*Yinpterochiroptera occupying similar micro-habitat, show greater niche overlap and similar niche breadth.”*  Please introduce the species before stating the hypotheses. This is the first time the bats and some traits are mentioned. Please describe niche breadth and overlap in this context. Does this mean the authors are trying to determine overlap in roost needs/preferences of species using the temples and that they expect Yinpterochiroptera to have overlapping needs with each other? Why does this matter? Please clarify this hypothesis. Are Yinopterochiroptera the only group that use the temples? Are they all cave-dwelling?

7. *“2. Microclimate conditions inside the temples are very stable and any influence on*

*bat abundances could be a reflection of food availability or disturbance at roost and therefore we expect bats to show seasonal changes in niche overlap and breadth due to variability in crop and insect availability.”* This hypothesis is confusing to me. I’m not sure what you are hypothesizing. Would it not be better to test abundance changes seasonally rather than niche overlap and breadth? The climate/monsoon seasons could also be described before introducing this hypothesis. Additionally, it is unclear how disturbance at roost and food availability were measured.

8. *“3. Since the structure of ancient temples are all similar, we do not expect any*

*change in microhabitat availability in temples with and without bats, instead we expect bat abundances driven by the availability of landscape features especially seasonal crops around the temples.”* Please describe how the temples differ or are similar to each other and the seasonality of crops before introducing this hypothesis. Also, is it not true that the temples are structurally different? Some are smaller/larger and more complex than others?

Materials and methods:

9. *“The ancient temples(>400 years) are built of large granite stones and provide a niche for several bat species.”* I think the authors mean that the temple structures provide roost habitat, not a full niche.
10. *“The surveyed temples are situated in an agricultural matrix surrounded by rice paddies or bananas”* Are the temples completely surrounding in the whole 5 km radius landscape or just mostly surrounded in the immediate area around the temple? What is the extent of the surrounding agriculture? An agricultural matrix implies mixed cover types so this sentence is somewhat confusing.
11. *“stone pillars that supported the stone roof was considered a separate habitat as they provided a niche for few species that roost in the gaps created between the stone joints this was categorized as crevices”* This sentence is a little confusing as it reads. Were the stone joints in the pillars categorized as crevices? The stone pillars provided a roost, not a niche.
12. *“In 2012 we measured temperature and humidity while sampling for bats and found no difference between temples and therefore not considered in the analysis.”* What are the temperature and humidity conditions in these temples and is it similar to cave conditions used by these bats?
13. *“Since we did not have information on how far the different bat species forage in the landscape we took 5 km based on the study on the large bat Megaderma lyra (Audet et al 1991).”* Did the authors consider testing multiple spatial scales? Many bat studies have determined that landscape scale factors are important at different scales. See the discussion in Bellamy et al. 2013 “Multiscale, presence-only habitat suitability models: fine resolution maps for eight bat species” in the Journal of Applied Ecology 50: 892-901 (available at: <https://besjournals.onlinelibrary.wiley.com/doi/full/10.1111/1365-2664.12117>) about importance of factors at multiple landscape scales. In the case of not knowing foraging distance for all species, it would improve this study to quantify landscape availability at more than one scale to determine if it is important (For example, maybe test 500 m, 1 km, and 5 km or landscape scales that are relevant to bats in other parts of the world). What kind of bat is the *Megaderma lyra*? Is it an insectivorous species?
14. *“If a land use element is present we gave a value of 0.25 in each quadrant and calculated the frequency of each land use for the entire 5km radius area.”* Is 25% (i.e, 0.25) sensitive enough to detect variation? Why 25% and not 10%? If a 5 km radius landscape only has a little bit of banana, but it is present, then it would still get a value comprising 25% of the landscape? This may be a limitation of the study to mention in your discussion. Would the results be different if the 5 km landscape was divided by 10% increments?
15. Did land use around each temple differ or was it correlated among the 5 sampling years?
16. *“The best model was selected using.”* Please finish this sentence and indicate what AICc weights and delta AIC value criteria were used to determine plausible models.
17. *“We constructed a linear model to explain the species richness and abundance of bats in the temples based on the microhabitat and landscape features. We based the variables used in our linear models on our observations made in 2012.”* Does this mean that only the landscape features from 2012 and the bat counts from 2012 were used? I think it would be statistically incorrect if all survey years at the same temples were treated as independent temples in the models.
18. Please organize the data analysis section to be more clear which analysis you are doing to answer which question/hypothesis and clarify which data were used to examine microhabitat and landscape factor influence on abundance and richness. In other words, did you use only data from 2012 or did you treat each repeated survey as a separate “temple”? If only used 2012 data, what were the monsoon conditions at that time?

Results:

19. *“The total number of roosts sampled was 351 across 246 temples in 5 years.”* This sentence is a little confusing. There were only 59 temples. Were any of the roosts that were repeatedly sampled the same roosts as previous years?
20. *“None of these bat species is threatened as per IUCN category.”* Are any of them considered “data deficient”? Please move this species information to the introduction to introduce the species that use the temples.
21. *“The lmer model explained 67% of the variation in species richness of which 55% were explained by the fixed factors.”* Do you mean the model averaged mixed model comprising all models with AICc <5 or the top model or something else?

Discussion:

The authors are effective at relating the results to the previous literature and this is the strongest section of the paper.

22. Mention the limitations of the study (e.g., temples were chosen based on logistics rather than randomly, only one landscape scale was considered, land use in the surrounding 5 km radius was considered at a course resolution (0.25))
23. From your results, are their certain species that may be more impacted by temple renovation/disturbance?

Figures:

24. Fig 1 map is a bit blurry. I suggest increasing dpi/resolution. Did there appear to be any spatial pattern of temples that did not have bats vs. those that did? If so, mention in discussion.
25. Fig 4 bar graph gray colors are difficult to differentiate between dark room and hall.
26. Fig 5 is this a bar graph of all temples, including the ones that were resampled? If so, perhaps plot the average species richness across the 5 years against the no. of buildings in each temple. It is difficult to interpret as is.
27. Same comment as last figure (which is also labeled Fig 5. Is that intentional?) What is DR? Define in figure or in parentheses in caption.
28. Fig 6 I suggest the axis title say “Proportion of rice paddy in 5 km radius” or similar
29. Fig 8 This figure is difficult to read with No. villages plotted on top of the land use classes.
30. Table 1: Nicely presented. Please indicate in the caption at what values are there considered an overlap? Higher values mean more overlap?
31. Table 4: Missing a variable after the “+” under Hiposederous speoris? Did you mention that you also had species-specific models in the main text?
