# Supplementary material for: Temples and bats in a homogeneous agriculture landscape: Importance of microhabitat availability, disturbance and land use for bat conservation
Source: PLoS One. 2022 Jul 13;17(7):e0251771. doi: 10.1371/journal.pone.0251771 (PMC9278754; doi:10.1371/journal.pone.0251771)
Supplement: Attachment — Submitted filename: Review.docx [file pone.0251771.s004.docx]

Review: Temples the last abode for bats in a homogeneous agriculture matrix: Importance of

microhabitat availability and land use factors for bat conservation.

Ganesh et al studies historic temples as roosting habitat for bats in India. This work highlights the importance of anthropogenic structures as essential roosts, particularly as temples are places where humans are actively working to remove bats. While focusing on one region in India, the authors conducted roost counts and identified species of bats over several years while also assessing each temple for several microhabitat and landscape factors. They conducted linear models to compare species richness and abundance to the landscape and microhabitat variables and calculated niche breadth and overlap. The authors found seven species of bat across forty seven out of fifty-four temples that they surveyed. Dark rooms and corridors were found to be the most important microhabitats for roosting, while landscape effects had little overall effect albeit with some species-specific instances. Bat abundance varied by species in season, but overall was lowest during the monsoon season. There was little species overlap in temples and little niche overlap. The authors attributed a lot of the microhabitat preferences to species-specific life history and roosting preferences. The seasonality differences correlated with the parturition and lactation of many of the species. While the landscape effect had a weak response, the importance of scrub and open landscape is still noted. Overall, this paper lays the foundation for further studies and emphasizes the importance of an anthropogenic roost for bats. This paper certainly merits publication with additional clarification and editing.

My first concern overall for the paper is some grammatical and formatting issues. I have listed specific examples of reoccurring problems throughout at the end of this review, however there are additional grammar edits that need to be fixed.

My second concern is with the landscape ecology analysis and discussion throughout the paper. The analysis of the landscape features was measuring presence/absence of landscape features over a significantly sized area. This broad measuring does not get at some of the heterogeneity of the landscape and may explain some of the lack of effect of the landscape features in the model. A more rigorous measurement of the landscape types could be better such as using the total amount or percentage of land cover type in the 5km buffer. As the remote sensing data may be unable to provide this level of detail (as mentioned in the methods), a mention of this drawback in the discussion would be warranted. Additionally, the discussion section touched on the landscape feature briefly but the middle of the paragraph still discussed temple characteristics such as festivals and renovations and the ending sentences focused on site fidelity. The importance of open spaces was stated but never explained or elaborated on. A deeper discussion with a paragraph focused solely on landscape characteristics while moving the still-relevant topics to more appropriate places would improve the analysis especially since the third hypothesis on landscape features was disproven.

Overall, this paper has significant importance for publishing by emphasizing the importance of temples as habitat worthy of conservation especially at a time when bats are at a high risk of exclusion from places because of the pandemic. Some modifications are necessary to clarify the paper and its findings, however they do not detract from the importance of the study.

Grammatical and Formatting Comments:

Check the spacing throughout the article. There should be a space in between the last word and opening parentheses. On the last sentence of page 11, there should be a space after the comma and between each year. The same issues occur in the caption for Table 4 with the list of symbols. Lastly, there should be a space in between genus and species in the species names in the results - I.e. M. lyra, not M.lyra.

In the first two paragraphs of the introduction, there are a few words and several citations where the year is in blue. They should all be in black since they are not hyperlinks.

A w is bolded in the word “with” in the beginning of the full paragraph on page 10.

Throughout the entire paper, halls is followed by (corridors) or vice versa each time. I think saying “halls and corridors” is appropriate the first time and then use one consistently for the rest of the paper.

Figure 1 appears blurry, so I would recommend the resolution to be larger. Partially because of the resolution, the temples and town dots are hard to tell apart. It would be easier to read if one of the dots was hollow.

Figure 3 does not have axis labels which helps the reader.

In Figure 4, the dark room and hall patterns are very hard to tell apart between the bars. A different pattern would make the graph easier to read.

Figure 5 has extra ticks around the outside of the graph.

In Figure 8, the no-village bar is distracting and confusing as it overlaps with the error bars.
