# Supplementary material for: Temples and bats in a homogeneous agriculture landscape: Importance of microhabitat availability, disturbance and land use for bat conservation
Source: PLoS One. 2022 Jul 13;17(7):e0251771. doi: 10.1371/journal.pone.0251771 (PMC9278754; doi:10.1371/journal.pone.0251771)
Supplement: Attachment — Submitted filename: PONE-D-21-13889_reviewer (2).docx [file pone.0251771.s005.docx]

Manuscript	[Click here to access/download;Manuscript;Bats in ancient temples of South India.docx](https://www.editorialmanager.com/pone/download.aspx?id=28905772&amp;guid=11b36e29-028b-48a7-870a-5e204ebe715a&amp;scheme=1)

### Temples the last abode for bats in a homogeneous agriculture matrix: Importance of microhabitat availability and land use factors for bat conservation.

**Authors: T.Ganesh, A.Saravanan and M.Mathivanan**

Ashoka Trust for Research in Ecology and the Environment (ATREE), Royal Enclave, Sriramapura, Jakkur Post, Bangalore 560064, Karnataka state, India

### ABSTRACT

Anthropogenic structures such as temples in south Asia are widely used by bats as roosting and nursery sites. Temples are being renovated and bats are removed from such places on a regular basis even more so after the pandemic. However, there is no detailed understanding of what makes temples favorable for bats and what factors are important for their conservation. Here we relate temple microhabitat characteristics and land use around ancient temples(>400 years) to bat species richness and abundance in the Tamiraparani river basin of south India. Temples were selected along the basin based on logistics and permission to access them. Bats were counted at the roost in the morning and late afternoon inside the temples. Temple characteristics such as dark rooms, corridors, crevices and presence of tower were recorded after the countWe also recorded land use such as crop, plantations, scrub and water availability within 5km radius of the temple. We used GLMM to relate the counts in temple with microhabitat and land use. We sampled 59 temples repeatedly across 5 years which yielded a sample of 256 surveys.

The total number of bats counted was 20,211 of which *Hipposideros speoris* was the most common (9,715) followed by *Rousettus leschenaulti* (5,306), *Taphozous melanopogon* (3,196), *Megaderma lyra* (1,497) *Tadarida aegyptiaca* (303), *Pipistrellus sp* (144) and *Rhinopoma hardwickii* (50). About 39% of the abundance was accounted by dark rooms while 51% by halls(corridors) around the deity room. Species richness and total abundance was related to number of building in the temple and availability of dark rooms. Land use elements only had a weak effect but open natural habitat were important for most species. The bats were also seasonal with the monsoons having the lowest number of bats which we attribute to the breeding behavior of the species. We conclude that retaining undisturbed dark rooms with small exits in temples and other dimly lit areas and having natural areas around temples are critical for bats conservation in the area.

Key words: Bats; microhabitat; Hindu temples; India; landscape,

# Introduction

Many studies suggest that human-modified landscapes with a heterogeneous mosaic of different land-uses can preserve species-rich bat assemblages (Medellín et al. 2000; Moreno and Halffter 2001; Estrada and Coates-Estrada 2002; Harvey et al. 2006; Medina et al. 2007; Barragan et al. 2010; Mendenhall et al. 2014). This is possible with the concurrent availability of microhabitats that bats use as roosting sites ranging from caves, crevices, tree cavities, foliage, and several types of anthropogenic structures (Voigt and Kingston 2016). These microhabitats being important features of a bat's environment, the selection of specific roost sites can strongly impact the survival and fitness of bats (Vonhof and Barclay, 1996, Humphrey, 1975; Stebbings, 1995). However, emerging challenges such as changing agricultural practices and climate change pose major threats to bat assemblages and populations in human-dominated landscapes (Jones et al.

2009) and could negatively impact human populations by altering the ecosystem services that bats provide (Kunz et al. 2011, Williams-Guillén et al 2016).

Most studies on the use of anthropogenic structures by bats have come from urban areas and under temperate conditions (Kunz et al., 2011; Jenkins et al., 1998; Clem, 1993). While it is accepted that bats have been poorly studied in the Paleotropics (Meyer et al 2016), the selection of microhabitats and landscape-level influences on their population remains poorly known (McNab, 1982; Ho and Lee, 2003, Kerth et al., 2001). In tropical South Asia, several species of both insectivorous and frugivorous bats use temples as roosting sites (Bates and Harrison, 1997, Korad 2007). In south India numerous very old (>400y) temples dot the landscape and form important bat habitats (Swamidoss et al. 2012). These are situated in a human-dominated agricultural matrix that is changing due to several economic reasons and climate change effects (Fischer et al. 2009, 2010; Jones et al. 2009). Populations of bats in many temples have dwindled (Vanitharani 2004, Mahandran et al.2015) and many are also directly killed during temple renovation (Addline et al 2004). The changing attitude of people towards bats has also led many temples to be made “bat-proof” by placing nets and power washing roost areas with chlorinated chemicals to prevent bats from colonizing. The recent pandemic has further made them

more vulnerable to disturbance and direct persecution. It’s therefore essential to understand what attributes of temples are important for bats and such information can help articulate bat conservation efforts in such temple landscapes.

Despite the numerous threats and the importance of bats in providing key ecosystem services in human-dominated landscapes (Wagnet et al. 2014, Thet and Mya 2015), there is no systematic study on the ecology of bats in temples while behavioural studies have been carried out on species that occupy temples in South India (Audet et al. 1991, Leippert et al.2000). Moreover**, w**ith populations of bats dwindling no attempt to monitor bats in the temples or how important are temples in an agricultural matrix has not been investigated. We initiated a bat monitoring program in 2012 in temples of south India to understand why bats use temples and how the population of species is responding to disturbance and land use change. In this study, we identify temples used by bats for roosting in the rice paddy-dominated agricultural landscape in South India. We record the species composition, rarity, and abundance of bats in temples and identify factors that promote bat colonization such as microhabitat availability in temples and landscape features surrounding the temples. While much of the bat diversity in human-dominated landscapes can be attributed to a combination of habitat diversity and roost site availability in an area, in tropical monoculture areas with rice paddies and banana with low habitat heterogeneity, it would be interesting to compare the role of land use elements and availability of microhabitats on bat species richness and abundances. In this paper, we test three major hypotheses 1. Given that the temple bats are mostly cave-dwelling species we expect larger Yinpterochiroptera occupying similar micro-habitat, show greater niche overlap and similar niche breadth. 2. Microclimate conditions inside the temples are very stable and any influence on bat abundances could be a reflection of food availability or disturbance at roost and therefore we expect bats to show seasonal changes in niche overlap and breadth due to variability in crop and insect availability. 3. Since the structure of ancient temples are all similar, we do not expect any change in microhabitat availability in temples with and without bats, instead we expect bat abundances driven by the availability of landscape features, especially seasonal crops around the temples.

## Materials and methods

**Study area**

The study was carried out in Tirunelveli & Thoothukudi Districts of Tamil Nadu state in South India (**Fig 1**.). Several ancient temples exist in the districts along the perennial river Tamiraparani and its tributaries. The river is a lifeline for agriculture and the dense human population in a semi-arid landscape. The ancient temples(>400 years) are built of large granite stones and provide a niche for several bat species. There are over 4041 temples (HR&CE, 2020) in the study area with 2-3 villages having one ancient temple. The typical structure of the temples includes a tower that stands at the four entrances and one over the deity. The deity chamber is usually lit by an oil lamp and an electric bulb where only the priest can enter and devotees are not allowed. A corridor (hall) surrounding the deity chamber which is used by people for walking, and several rooms including storerooms and a kitchen which are dark and rarely frequented by people. The surveyed temples are situated in an agricultural matrix surrounded by rice paddies or bananas and usually located towards the corner of a village or town.

### Fig 1. Map of the study area. The dots represents temple sampled.

**Temple selection**

We selected 59 temples from the Tamrabarni basin and surveyed them with 2-4 personnel between 06:00-12:00 h and again between 16:00 to 17:00h to detect the presence of bats. The survey was done from May to Dec and the same temples were visited across the years 2012,2013,2014,2018,2019. for a total of 246 surveys

All species of both *Yinpterochiroptera* and *Yangochiroptera* bats found roosting inside the temples were recorded. The presence of bats in the temple was identified by several signs such as droppings and smell, interviewing local people, temple priests, and caretakers. Dark corners, temple roof, abandoned & isolated rooms inside the temple and temple tower were all scanned for chiropteran presence with a red LED light. Once bat colonies were detected, we counted the number of individuals and in some cases, especially in closed towers, we took photographs to estimate them.

## Temple features (microhabitat)

There were multiple locations inside temples where bats could have roosted: Rooms which were usually closed structures with few small openings, very low natural light and not frequently used by people were categorized as *Dark* rooms; stone pillars that supported the stone roof was considered a separate habitat as they provided a niche for few species that roost in the gaps created between the stone joints this was categorized as *crevices*; temple *towers* which were conical structures built over the entrance or the deity and wide walking space around the deity room covered by a stone roof was termed as *halls.* We also counted the number of independent buildings in the temple complex. In 2012 we measured temperature and humidity while sampling for bats and found no difference between temples and therefore not considered in the analysis. We could not set dataloggers for continuous monitoring because of objection by temple authorities to placing any devices inside temples. Temple size was obtained from the visual observation, those with deity rooms and corridor considered as small, those with deity rooms, corridor and tower considered as medium-sized, and those having deity rooms, multiple towers, and two corridors (inside and outside) considered as large.

## Land use features

Habitat features can be easily measured from satellite-based land use classification however in this case we could not do that as the region had high levels of cloud cover during the sampling period from May to Dec, being the monsoon season. We therefore, developed a novel approach by characterizing land use within a 5 km radius around each temple roost based on Google Earth images when available and substantiated by informal interviews with farmers from around each temple. This was done for each year and month of sampling. Since we did not have information on how far the different bat species forage in the landscape we used 5km based on the study on the large bat *Megaderma lyra* (Audet et al 1991). For the Google Earth classification, we divided the 5km radius around the temple into 4 quadrants and recorded the presence or absence of seasonal crop type(rice paddy, banana), perennial vegetation(scrub and other natural vegetation), availability of water (ponds /rivers) and number of villages within the 5km distance. If a land use element was present we gave a value of 0.25 in each quadrant and calculated the frequency of each land use for the entire 5km radius area. If particular land use is found in all four quadrants it had

a frequency 1 and 0.25 if only recorded once. This also gives an approximate extent of each land use around the roost.

## Species identification and abundance

Bats were identified to species based on detailed photographs taken in the temple using the bat identification guide by Bates and Harrison (2001).

Bats were counted at the roost when they were least active (late morning and early evenings) using a flashlight with red filters. Where possible photographs were taken as soon as bat aggregations were noticed in a particular microhabitat inside the temple and later counted to estimate abundance (Carpenter, 2014). Several pictures were taken in quick succession to estimate and avoid bias in counting. Binoculars were used to see the species characteristics for identification. We could not do a roost exit count in the evening as it was not logistically possible to be in each temple at the right time for the count.

## Data analysis

We compared niche breadth and overlap of species to see if there are any exclusive use of microhabitats and the level of overlap between species. Niche breadths were compared using Levin’s Index while niche overlap was calculated using Pianka’s Niche Overlap Index (Inger et al., 1987). We constructed a linear model to explain the species richness and abundance of bats in the temples based on the microhabitat and landscape features. We based the variables used in our linear models on our observations made in 2012. We choose variables that were not correlated with other variables to reduce variable redundancy and were also biologically meaningful. We related total species richness and abundance(counts) of bats at the temple with temple variables and landscape elements using mixed models. We used lmer pakage in R for species richness as it was normally distributed. Since the counts were over dispersed for abundances, we used a negative binomial with glmmadmb package in R. We considered temple ID as a random factor in the model and temple characteristics and land use elements as fixed factors. If more than one roost was found in a temple, we pooled roost counts and recorded total species richness for the temple. The models were ranked using Akaike's Information Criterion corrected for sample size and based on calculated Akaike weights. The best model was selected

using. Model averaging was done for closely related models following(Mazerolle 2020) and model selection criteria in MuMIn package(version 1.43.17) in R. We compared medians of microhabitat and landuse variables using Kruskal Wallis and Mann Whitney tests. All data analysis and statistical tests were carried out using Microsoft Excel®, PAST® and R version 4.

# Results

## Species richness, abundance, and seasonality

Forty-seven of the 59 temples surveyed housed bats and 51 of these had multiple roosts and only 8 had a single roost across years. The total number of roosts sampled was 351 across 246 surveys in 5 years. Seven species of bats were recorded of which six were Yinpterochiroptera and one *Rousettus leschenaulti* was a Yangochiroptera. Among the Yinpterochiroptera, *Hipposideros speoris* was the most frequently seen species found in 135 (38%) roosts sampled followed by *Taphozous melanopogon (58), Megaderma lyra (29) Tadarida aegyptiaca (26) Rousettus leschenaulti (22), Pipistrellus sp (9) and Rhinopoma hardwickii (7)*. Forty-five % of the temples had only one species, 34% had two species, 15% had three species and 6% had four species of bats. In all 20,211 bats were counted of which *Hipposideros speoris(HS)* was the most common (97,15) followed by *Rousettus leschenaultia(RL)* (5,306), *Taphozous melanopogon* (TM)(3,196), *Megaderma lyra* (ML)(1,497) *Tadarida aegyptiaca (TE)* (303), *Pipistrellus sp (PS)* (144) and *Rhinopoma hardwickii* (RH)(50). None of these bat species is considered threatened as per the International Union for Conservation of Nature (IUCN).

The most abundant *H.speioris* did not show variations across months except in the first summer(May-June) when it was high while all other *Yinpterochiroptera* bats were both low in summer and in the monsoon(NEM)(Nov and Dec, **Fig 2**)). The overall abundance of *Yinpterochiroptera* bats, however, showed lower variations than the fruit bats *Rousettus leschenaultia* which had a sharp peak in the pre-monsoon seasons. Two other species *Pipistrellus sp* and *Rhinopoma hardwickii* were not analyzed because of fewer samples. The percentage of temples with bats drops to 34% in the NEM from 88% during summer(**Fig 3**). In general monsoon months show lower bat abundance and bat occupancy as we had hypothesized.

### Fig 2. Mean abundance(x±se) of bats across seasons in temples. NEM: Northeast monsoon(Nov-Dec), SUM1 (May-June), SWM(July-Aug) and SUM2(Sep-Oct).

**Fig 3. Percentage of temples occupied by bats across months.**

**Bats and Roost characteristics**

We distinguished four separate microhabitats inside the temple that were used for roosting by the seven species (**Fig 4**). About 39% of the total abundance was in dark rooms while 51% of bats were in halls (corridors) around the deity room was the main microhabitat for 5 out of 7 species. Crevices in pillars were preferred by three species .

In terms of species occupancy, *H. speoris* was observed in three out of four microhabitats, but most commonly in dark rooms. Halls(corridors) were used only when darkrooms were not available or disturbed. Likewise *M. lyra* and *T. melanopogon*, *Rhinopoma hardwickii* were found in two microhabitats (hall and crevices) while *T. aegyptiaca* was found only between pillars(crevices), and *Pipistrellus sp* found only in roof crevices. The fruit bat *R. leschenaulti* preferred halls.

### Fig 4. Percentage of microhabitats used by the different species of bats for roosting.

**Seasonal changes in species overlap and niche breadth**

The species overlap across temples is very low except between *T. melanopogon and M.lyra.* However, the overlap between species varies with seasons. *H.speioris* has a strong overlap with *M.lyra* and *T.melanopogon* during summer and weak in other seasons. Similarly, *M.lyra* has a strong overlap with *T.melanopogon* during the southwest monsoon. In general, during the northeast monsoon when heavy rains occur in the region, there is very little overlap in the species across temples while in the dry season *H.speoris* and *T. melanopogon* and *M.lyra* the three large Yinpterochiroptera bats overlapped with each other (**Table 1**). The fruit bat *R.leschenaulti* has a negligible overlap with other species except during summer when it shares space with *T. aegyptiaca*. Similarly, niche breadth also varied with season and northeast

monsoon showing shorter niche breadth for the large bats than in other seasons. Niche breadth was also highest for the most abundant *H. speoris* while others had similar niche breadth (**Table 2**). These results only partly support our niche hypothesis of overlap among large Yinpterochiroptera bats and support our hypothesis of variations across seasons.

### Table 1: Species overlap(Pianka’s overlap index) across seasons in temples of south India. NEM: Northeast monsoon, SWM: Southwest monsoon and dry season include SUMM1 and SUMM2.

**Table 2. Niche breadth of bat species in temples. NEM: Northeast monsoon, SWM: Southwest monsoon and the dry season includes SUMM1 and SUMM2.**

**Temple and landscape features**.

The number of buildings in the temple complex significantly affected species richness and bat abundance (counts) in temples **(Fig 5a)**. Dark room availability strongly influenced the total abundance of bats and only weakly impacted species richness(**Fig 5b, Table 3a).** The lmer model explained 67% of the variation in species richness of which 55% were explained by the fixed factors.

Land use elements did not show a very strong effect on abundances but availability of paddy(**Fig6**) and water had a negative influence on species richness while banana availability and water had a negative influence on abundance. The availability of open scrub and uncultivated areas had a positive influence on abundance but much less on species richness (**Table 3b**).

### Fig 5a. Relationship between species richness and number of buildings in temples

**Fig 5b. Effects plots showing the relationship between dark rooms(DR) and number of buildings on bat abundances.**

**Fig 6. Effects plots showing the relationship between paddy availability and bat species richness.The plot is based on the most parsimonious model.**

**Table 3: Two GLMM models were fit separately for temple and landscape for species richness (lmer model) and bat abundances( glmm.admb model) in temples. Temple id and month is considered as random factors.**

Response to habitat and landscape features varied across species (**Table 4**). *Hiposederous speoris* abundance was positively influenced by dark rooms, the number of buildings and negatively influenced by banana and positively by open scrub. Counts of *T.melanopogon, T.aegyptica and R.leschenaulti* were also positively related to the number of buildings in the temple. *M.lyra* showed a negative response to dark rooms and corridors and positive response to trees in temples. Trees around temples and presence of tower had a negative influence on *H.speoris* and *T.aegyptica*. All of these are weak responses(**Table 4**). Land use did not have strong influence except banana having a negative influence on *H.speoris*. Availability of open scrub had a positive influence across on all the species except *T.melanopogon* which also showed a negative effect of water*.* **(Table 4).**

### Table 4: The top models(∆AICc<5) of species response to microhabitat (temple model) and landuse elements (landscape model). Bld-No.buildings,DR:Dark Room,TR: Trees,HA:Hall,TW:Tower,BA:Banana,PD:paddy,SC:Scrub,VL: No. villages,WA:Water, NULL: Null model

**Table 5:Species response to microhabitat and landscape elements availability. Model averaged parameter estimates of top models (<5 ΔAICc)**

Though the availability of roost microhabitats across temples with and without bats was not significantly different (KS test; D=0.4, p=0.689), dark rooms, tower, and number of buildings were more in temples with trees (Mann-Whitney U=3917 p<0.01, **Fig 7**). Of the total temples sampled, 80% of them had bats and they were specific to these temples and showed strong affinity to them. There was no overall difference in land use between bat vs no bat temples (paddy, Mann-Whitney, U :5037, p=0.236, water U :5159.5, p=0.73365, banana U: 4569.5, p=0.057917; coconut U: 5561.5,

p=0.960, veg U: 90 p=0.78717,**Fig 8**). The availability of microhabitats in the temple especially the number of buildings and dark rooms is, therefore, a key determinant of bats in temples while land use elements had only a weak effect.

### Fig 7.Proportional availability of habitats in temples with and without bats.

**Fig 8. Mean value of habitat variables 5km around the temples across bat species.**

**DISCUSSION**

Why do bats prefer some temples? Bats were found in about 80% of the temples and over 20,211 individuals were counted. Though we recorded only 7 species the most common species *Hipposideros speoris* (9715) accorded for nearly half of the population and also found in more than 50% of bat inhabited temples. *H. speoris* is a very common species across India and Srilanka and known to occur in large congregations and shows high fidelity to roost sites (Selvarathinam and Marimuthu, 2013). The other 6 species are also not rare or threatened and known to use a multitude of anthropogenic structures to roost (Bates and Harrison, 1997). Except for *T. aegyptiaca, Pipistrellus sp* and *Rhinopoma hardwickii* species counts of all others ranged between 450 to 200 in each temple and appear to follow the general tendency of bats that roosts in caves and manmade structures to be highly gregarious (Gaisler, 1979). The overlap between large insectivorous species preferring dark rooms such as *M.lyra* and *T. melanopogon* at roost was higher than for smaller *H.speoris* and *T.aegyptica* and the frugivorous *R.leschanaulti*. This could be because *M.lyra* is a predator of larger animals including rodents and small bats (Audet et al., 1991; Bates and Harrison, 1997), and species such as *T.aegyptica* may tend to avoid it even though they occupy a different roost microhabitat. While larger *T. melanopogon* seem to be unaffected which may be due to the behavior of *T. melanopogon* which is not only large but huddles together while roosting unlike the other species and therefore may be less prone to gleaning attacks from *M. lyra.* This may also explain why we see a greater overlap between *M.lyra* and *T.melanaopogon* but much less with *H.speoris.* The greater niche breadth of *H.speoris* also facilitates it to coexist with these species in the same habitat.

Most species counts decline in the temples during the northeast monsoon. The NEM is also a time of high rainfall, extensive paddy cultivation, and time of high insect activity. This is also the parturition and lactation period for most bats in the region and species like *Hipposideros alter(not recorded in this survey)* take advantage of abundant Lepidoptera prey during the monsoon months in the study area (Sophia and Vanitharani 2006). But studies in Thailand on similar species have shown insect diversity is less influential than water availability (Suksai and Bumrungsri 2018). During the monsoons bat abundance falls and also the proportion of temples with bats have decreased and they seem to have dispersed probably the young ones have moved

into other habitats or even using dense foliage. This was true for the frugivore *R.leschanaulti* but also *M.lyra* and *T.aegyptica. M.lyra* breeds during November and sexes disperse after copulation leaving behind females at most roosts(Harrison and Bates 1997). This could be a reason for a fall in numbers after November but may not be true for *H.speoris* whose numbers don’t change in the monsoon as males pair with females for a year or more and do not desert the roost (Chanderasekaran 2005). *R.leschanaulti* is known to favour banana plantations as show in Kerala state(Deshpande 2012) and could be moving roosts depending on the availability of fruits however peak monsoon is not abundant in bat fruits except for sporadic fruiting of *Ficus sp*. (Cauglin et al 2012). Since the relationship with land use variables are weak we suspect the fall in numbers during the monsoon is more due to dispersal of young bats than the effect of any particular landuse.

Several species that roost and breed in temples across India (Bates and Harrison, 1997; Leippert et al., 2000), are cave-dwelling species that have found temples as suitable alternative habitats. Our results show that temples offer dark, undisturbed roosts and crevices similar to caves and these are used by 6/7 species accounting for 80% of the abundance of bats in the temple. While we have pooled different types of dark spaces available in the temples, the one behind the main door of the temple and through which the temple tower can be accessed, is the least active place and occupied whenever there was no disturbance. These areas in old temples have several openings through which bat can easily fly out, a reason for such places selected by most bat species. All the ancient temples were built with granite stones stacked together that offered crevices for some species to roost such as the free-tailed bats (*T. aegyptiaca*) which could be seen even at eye level between pillars. Corridors (halls) surrounding the temple *sanctum* where the idol of the god is kept, is often long and dark during the day with a high roof which is a preferred place for the bats like *H. speoris* that remain unnoticed and therefore undisturbed during the morning hours when there is a movement of people. A similar response of cave-dwelling bats from genus *Hipposideros* has been found in Malaysia where they roost in very dark corners and high places (Rajasegarn et al 2018). We did not take species-wise roost height measurements so it’s not evident if species segregate roost according to height but unlike in a cave, many species that roost in dark corners seem to segregate spatially and can vary between temples.

While temple characteristics are major drivers of bat abundance and occupancy, the land use especially crop availability does not seem to play an important role across species. Several studies have shown the importance of landscape elements for the presence of bats (Ford et al., 2006; Neubaum et al., 2007). In this study however, we did not find such a strong response. The landscape was fairly homogeneous around the temples with paddy or banana grown around almost all temples across years. While bats were missing in many temples that had dark rooms, crevices, and halls, this could be more due to other disturbances such as festivals or renovation(Ganesh et al 2020). Temples with less trees and towers where preferred by *Hipposideros speoris* and *Tadardia aegyptiaca* which can be expected as both species are also dry area species and prefer caves and buildings(Bates and Harrison, 1997) and therefore not associated with wooded area and hence may prefer temples devoid of tree cover. The importance of open uncultivated natural areas such as scrub, grasslands and lightly wooded areas are important for almost all the species of bats and this can have significant conservation importance as natural areas are rapidly diminishing. The presence of bats is generally linked to microhabitat or stand-level structural conditions, however, Lewis (1995) also found that roost-site fidelity for bats varied depending on the availability and permanency of the roosts used and suggested that fidelity was high at buildings for these reasons. Supporting this a study on banded *H.speoris* bats from the study area showed 82% roost fidelity throughout the year (Selvarathinam, G Marimuthu 2013). Therefore the temples that have remained unchanged for centuries provide conditions for high roost fidelity despite changes in the landscape and therefore are more important for bats to persist in the area.

### Conservation of bats in temples

The study has shown that microhabitat within temples such as dark corners that are relatively less disturbed and rooms that were rarely used are essential for their persistence. Temples with numerous buildings increase the possibility of having dark rooms. The landscape factors were surprisingly less important though availability of open natural areas around temples are important for the abundance of bat species. Therefore conservation of bats would mean protecting the microhabitat in the temples from getting destroyed and retaining natural areas

around. Though not quantified, the significant threat to bats in the temples is coming from the recent spurt in the renovation of old temples where dark areas are lit, pressure cleaned to remove the odour of bats, painted and ‘bat proofed’ to keep away bats and pigeons from entering the premises. With current coronavirus and bat association being misreported bats have come under even greater threat and surveillance. Our surveys of bats in the same temples have shown that they have deserted some temples completely because of disturbances such as renovations and probably moved to other temples. Temples are in many places the only habitat for bats in the landscapes as many of the rocky hills have been destroyed. However, a metapopulation of bat can exist in the landscape which will help colonize temples depending on the temple characteristics and disturbance levels. More studies are needed to understand these dynamics.

### Acknowledgement

The work was partly supported by a grant from Bat Conservation International Global Grassroots Conservation Fund. We thank Maria,Lise and K.S.Seshadri for help in the field and M.B.Prashanth for making the map and general support. We are grateful to Dr.R.Ganesan, Dr.M.Soubadra Devy for their help during the initial stages of the work. We also tank Rajkamal, Seshadri, Prashanth and Soubadra for their comments on the MS.We thank Thamizhazhagan for helping in capturing bats and Muthupandi for assistance in the field. The Temple authorities and temple staffs in both Tirunelveli and Thoothukudi districts permitted us to do the study. Jermiah Rajanesan, director Dhonavur Fellowship and Maria Joseph, Head Master, Christuraja Hr., Sec., School, Palayamkottai helped us with logistics and we are grateful to them for their help.

### Literature cited

Addline, E.P.D., J.Vanitharani., S.Rajkumar, and R.T.Sundara. 2004. Government, law and temple bat management, pp-297-301. In: Muthuchelion,K (eds). Biodiversity Resources Management and Sustainable use. Centre for biodiversity and forest studies, school of energy, environment and natural resources, Madurai Kamaraj University, Madurai.

Audet, D., D.Krull, G. Marimuthu, S. Sumithran, and J. B. Singh. 1991. Foraging Behaviour of the Indian False Vampire Bat, *Megaderma lyra* (Chiroptera: Megadermatidae). Biotropica, 23: 63-67.

Barragan, F., C. Lorenzo, A.Moron, M.A. Briones-Salas, and S.Lopez. 2010. Bat and rodent diversity in a fragmented landscape on the Isthmus of Tehuantepec, Oaxaca, Mexico. *Tropical Conservation Science*, *3*(1), pp.1-16.

Bates, P.J.J, and D.L.Harrison. 1997. Bats of the Indian Subcontinent. Harrison Zoological Museum Press, England.

Chandrasekaran, M. K. 2005. Time in the living world. Universities Press,Hyderabad, 197pp.

Carpenter,E.R Gomez,D.L.Waldein and R.E.Sherwin. 2014. Photographic estimation of roosting density of Geoffroys Rousette Fruit Bat *Rousettus amplexi caudatus* (Chiroptera: Pteropodidae) at Monfort Bat Cave, Philippines. Journal of Threatened Taxa*, 6*: 5838-5844.

Cauglin,T, T.Ganesh and M.Lowman. 2012. Sacred fig trees promote frugivore visitation and tree seedling abundance in South India. Current science.102:918-922

Clem, P. D. 1993. Foraging patterns and the use of temporary roosts in female evening bats, *Nycticeius humeralis*, at an Indian maternity colony. Proceedings of the Indian Academy of Science, 102:201- 206.

Deshpande, K. 2012. Assessing diversity and distribution of bats in relation to land-use and anthropogenic threats in the southern Western Ghats, India. *Final Report Submitted to the Rufford Small Grants for Nature Conservation, 30pp*.

Estrada, A., and R. Coates- Estrada. 2002. Bats in continuous forest, forest fragments and in an agricultural mosaic habitat-island at Los Tuxtlas, Mexico. *Biological Conservation*, *103*(2), pp.237-245

Fischer, J., J. Stott, B.S. Law, M.D. Adams, and R.I. Forrester. 2009. Designing effective habitat studies: quantifying multiple sources of variability in bat activity. *Acta Chiropterologica*, *11*(1), pp.127-137.

Ford, W. M., J.M. Menzel, M.A. Menzel, J.W. Edwards, and J.C. Kilgo. 2006. Presence and absence of bats across habitat scales in the upper coastal plain of South Carolina. Journal of Wildlife Management*, 70*: 1200-1209.

Gaisler. J. 1979. Ecology of Bats. In Ecology of Small Mammals (D M Stoddard.ed.) *Chapman and Hall,* London, Pp 2B1-342. *386* pp.

Ganesh, T., A.Saravanan and M.Mathivanan. 2020. Let’s Protect the Bats Where They Live – Including in Our Temples (https://science.thewire.in/environment/bats-covid-19-temples-south- india/)

Harvey, C.A., A. Medina, D.M. Sanchez, S. Vilchez, B. Hernandez, J.C. Saenz, J.M. Maes, F. Casanoves, and F.L. Sinclair. 2006. Patterns of animal diversity in different forms of tree cover in agricultural landscapes. *Ecological applications*, *16*(5), pp.1986-1999.

Ho, Y.Y., and L.L. Lee. 2003. Roost selection by Formosan leaf-nosed bats (Hipposideros armiger terasensis). *Zoological Science*, *20*(8), pp.1017-1024.

Humphrey, S.R. 1975. Nursery roosts and community diversity of Nearctic bats. *Journal of mammalogy*, *56*(2), pp.321-346

HR&CE 2020 https://tnhrce.gov.in/hrcehome/temple_list.php

Inger, R. F., H. B. Shaffer, M. Koshy, and R. Bakde. 1987. Ecological structure of a herpetological assemblage in South India. Amphibia-Reptilia, 8: 189–202.

Jenkins, E. V., T. Laine, S. E. Morgan, K. R. Cole, and J. R. Speakman. 1998. Roost selection in the pipistrelle bat, *Pipistrellus pipistrellus* (Chiroptera: Vespertilionidae), in northeast Scotland. Animal Behaviour, 56:909-917

Jones, G., D.S. Jacobs, T.H. Kunz, M.R. Willig, and P.A. Recay. 2009. Carpe noctem: the importance of bats as bioindicators. *Endangered species research*, *8*(1-2), pp.93-115.

Kerth, G., M. Wagner, and B. Konig. 2001. Roosting together, foraging apart: information transfer about food is unlikely to explain sociality in female Bechstein's bats (Myotis bechsteinii). *Behavioral Ecology and Sociobiology*, *50*(3), pp.283-291.

Korad, V., K. Yardi, and R. Raut. 2007. Diversity and distribution of bats in the Western Ghats of India. *Zoos’ Print Journal*, *22*(7), pp.2752-2758.

Kunz, T. H., E. Braun, De Torrez, D. Bauer, T. Lobova, and T.H. Fleming. 2011. Ecosystem services provided by bats. Annals of the New York Academy of Sciences*,* 1223: 1-38.

Leippert, D., W. Goymann, H. Hofer, G. Marimuthu, and J. Balasingh. 2000. Roost-mate communication in adult Indian false vampire bats (*Megaderma lyra*): an indication of individuality in temporal and spectral pattern. Animal Cognition*,* 3: 99-106.

Lewis, S.E. 1995. Roost fidelity of bats: a review. Journal of Mammal, 76, 481–496.

Lianne C. Ball 2002.A Strategy for Describing and Monitoring Bat Habitat. The Journal of Wildlife Management , Vol. 66( 4): 1148-1153.

McNab, B.K. 1982. Evolutionary alternatives in the physiological ecology of bats. In *Ecology of bats* (pp. 151-200). Springer, Boston, MA.

Medellin, R.A., M. Equihua, and M.A. Amin. 2000. Bat diversity and abundance as indicators of disturbance in Neotropical rainforests. *Conservation biology*, *14*(6), pp.1666-1675.

Medina, A., C.A. Harvey, D.S. Merlo, S. Vilchez, and B. Hernandez. 2007. Bat diversity and movement in an agricultural landscape in Matiguás, Nicaragua. *Biotropica*, *39*(1), pp.120-128

Meyer, C.F., M.J. Struebig, and M.R. Willig. 2016. Responses of tropical bats to habitat fragmentation, logging, and deforestation. In *Bats in the anthropocene: Conservation of bats in a changing world* (pp. 63-103). Springer, Cham.

Moreno, C.E, and G. Halffter. 2001. On the measure of sampling effort used in species accumulation curves. *Journal of Applied Ecology*, pp.487-490.

Mahandran, V.,C. M. Murugan and P. T. Nathan, 2015. An unusual roosting behaviour of Schneider’s leaf-nosed bat, *Hipposideros speoris* at a cave-temple roost in Tamil Nadu. *Current Science*,109(5), pp 858:859.

Rajasegarn, P., N. Shazali, and F.A.A. Khan. 2018. Microclimate and Physiological Effects in the Roosts of Cave Dwelling Bats: Implications in Roost Selection and Conservation in Sarawak, Malaysian Borneo. *Zoological science*, *35*(6), pp.521-527.

Selvarathinam, T, and G. Marimuthu. 2013. Roost selection and fidelity of leaf-nosed bat,

*Hipposideros speoris*. International Journal Current Trends in Research, 2: 62-65

Sophia,E, and J.Vanitharani.2006. *Hipposideros ater* in the ecosystem of Tirunelveli, Tamilnadu. Journal of Theoretical and Experimental Biology. Vol. 3 Pp: 27-34 ISSN: 0972-9720.

Stebbings, R.E. 1995. Why should bats be protected? A challenge for conservation. *Biological Journal of the Linnean Society*, *56*(suppl_1), pp.103-118.

Suksai, P., and S. Bumrungsri. 2019. Water bodies are a critical foraging habitat for insectivorous bats in tropical agricultural landscapes of cantral Thailand. *Songklanakarin Journal of Science and Technology*.

Swamidoss, P.D., M.R. Sudhakaran, and P. Parvathiraj. 2012. Habitat preference of microchiropteran bats in three districts of Tamilnadu, South India. *International Research Journal of Biological Sciences*, *1*(5), pp.24-30.

Thet.T and K. M. Mya 2015. Harvesting the guano of insectivorous bats: is it sustainable? Journal of Threatened Taxa 7(6): 7296–7297

Vanitharani, J., 2004. The Emerging Trends in the Bio-Diversity of Bats in Tamil Nadu. *Mapana Journal of Sciences*, *2*(2), pp.14-27.

Voigt, C.C., and T. Kingston. 2016. *Bats in the Anthropocene: conservation of bats in a changing world* (p. 606). Springer Nature.

Vonhof, M.R., and Barclay. 1996. Roost-site selection and roosting ecology of forest-dwelling bats in southern British Columbia. Canadian Journal of Zoology, 74: 1797-1805.

Williams-Guillen, K., E. Olimpi, B. Mass, P.J. Taylor, and R. Arlettaz. 2016. Bats in the anthropogenic matrix: challenges and opportunities for the conservation of Chiroptera and their ecosystem services in agricultural landscapes. In *Bats in the Anthropocene: Conservation of bats in a changing world* (pp. 151-186). Springer, Cham.

Wanger, T.C., K. Darras, S. Bumrungsri, T. Tscharntke & A.M. Klein (2014). Bat pest control contributes to food security in Thailand.Biological Conservation 171: 220–223; <http://dx.doi.org/10.1016/j.biocon.2014.01.030>

Figure	[Click here to access/download;Figure;Figure 1.jpg](https://www.editorialmanager.com/pone/download.aspx?id=28905773&amp;guid=0199f7c2-22e0-4331-925d-e0ef75b09825&amp;scheme=1)

Figure2	[Click here to access/download;Figure;Figure2.jpg](https://www.editorialmanager.com/pone/download.aspx?id=28905774&amp;guid=f2cc2ae3-926b-45c0-8f8c-28d7a8841005&amp;scheme=1)

Figure3	[Click here to access/download;Figure;Figure 3.jpg](https://www.editorialmanager.com/pone/download.aspx?id=28905781&amp;guid=28264017-50d1-4f9a-8973-d1d1d46ea1c9&amp;scheme=1)

Figure4	[Click here to access/download;Figure;Figure4.jpg](https://www.editorialmanager.com/pone/download.aspx?id=28905782&amp;guid=6f9ea7dd-392e-454a-add3-7a4271dbbc5f&amp;scheme=1)

Figure5	[Click here to access/download;Figure;Figure 5a.tiff](https://www.editorialmanager.com/pone/download.aspx?id=28905784&amp;guid=d741b5be-f1c9-41f3-9d40-e2d6b272faae&amp;scheme=1)

Figure5	[Click here to access/download;Figure;Figure 5b.jpg](https://www.editorialmanager.com/pone/download.aspx?id=28905785&amp;guid=43bf04f1-71fc-4062-be0b-232ce6fb7964&amp;scheme=1)

Figure6	[Click here to access/download;Figure;Figure 6.tiff](https://www.editorialmanager.com/pone/download.aspx?id=28905786&amp;guid=f8ac9242-7f05-425f-9097-a35ce9f8111b&amp;scheme=1)

Figure7	[Click here to access/download;Figure;Figure7.jpg](https://www.editorialmanager.com/pone/download.aspx?id=28905787&amp;guid=a0c57d21-aaea-4486-b198-6cba5ccb823f&amp;scheme=1)

Figure8	[Click here to access/download;Figure;Figure 8.jpg](https://www.editorialmanager.com/pone/download.aspx?id=28905788&amp;guid=0cb93ea9-db39-4268-93f7-6fffb94f059a&amp;scheme=1)

Figure legend	[Click here to access/download;Figure;Figure-captions.docx](https://www.editorialmanager.com/pone/download.aspx?id=28905802&amp;guid=e3e3af0b-8842-4ec1-ad1d-a23664a2cdd5&amp;scheme=1)

Figure 1. Map of the study area. The dots represents temple sampled.

Figure 2. Mean abundance(x±se) of bats across seasons in temples. NEM: Northeast monsoon(Nov-Dec), SUM1 (May-June), SWM(July-Aug) and SUM2(Sep-Oct).

Figure 3. Percentage of temples occupied by bats across months.

Fig 4. Percentage of microhabitats used by the different species of bats for roosting. Fig 5a. Relationship between species richness and number of buildings in temples

Fig 5b. Effects plots showing the relationship between dark rooms and number of buildings on species richness and bat abundances.

Fig 6: Effect plot of paddy availability and number of species.

Fig 7.Proportional availability of habitats in temples with and without bats.

Figure 8. Mean value of habitat variables 5km around the temples across bat species.
