# Supplementary material for: Temples and bats in a homogeneous agriculture landscape: Importance of microhabitat availability, disturbance and land use for bat conservation
Source: PLoS One. 2022 Jul 13;17(7):e0251771. doi: 10.1371/journal.pone.0251771 (PMC9278754; doi:10.1371/journal.pone.0251771)
Supplement: Attachment — Submitted filename: PONE-D-21-13889_R1_Feb2022.docx [file pone.0251771.s007.docx]

MMaannuussccrriipptt

[Click h](https://www.editorialmanager.com/pone/download.aspx?id=28905772&amp;amp%3Bguid=11b36e29-028b-48a7-870a-5e204ebe715a&amp;amp%3Bscheme=1)[Celircekthoeareccteosasc/dcoewssn/ldooawd;nMloaandu;Mscarinput;sBcaritpst;iMn aanncuisecnrtipt.docx](https://www.editorialmanager.com/pone/download.aspx?id=30281355&amp;guid=1b397e18-95fc-46fa-9db4-934c2051fbc8&amp;scheme=1) [temples of South India.docx](https://www.editorialmanager.com/pone/download.aspx?id=28905772&amp;amp%3Bguid=11b36e29-028b-48a7-870a-5e204ebe715a&amp;amp%3Bscheme=1)

**Temples the last abode for bats in a homogeneous agriculture landscape: Importance of microhabitat availability, disturbance and land use factors for bat conservation.**

**Authors: T.Ganesh, A.Saravanan and M.Mathivanan**

Ashoka Trust for Research in Ecology and the Environment (ATREE), Royal Enclave, Sriramapura, Jakkur Post, Bangalore 560064, Karnataka state, India

**Abstract**

Anthropogenic structures such as temples in south Asia are widely used by cave-dwelling bats as roosting and nursery sites. These roosts are constantly under threat as temples are being renovated and bats removed from such places even more so after the COVID-19 pandemic. No detailed understanding exists on what makes temples favorable for bats and what factors are important for their persistence. Here we relate temple microhabitat characteristics and land use around ancient temples (>400 years) to bat species richness and abundance in the Tamiraparani river basin of south India. Temples were selected for sampling along the basin based on logistics and permission to access them. Bats were counted at the roost in the mornings and late afternoons from inside the temples. Temple characteristics such as dark rooms, corridors, crevices and presence of tower were recorded after the count along with disturbances to the roost. We also recorded land use such as crop, trees, scrub, grassland, urban areas and water availability within 5 km radius of the temple based on ESA landuse classifications. We used GLMM to relate the counts in temples with microhabitat and land use. We sampled 59 temples repeatedly across 5 years which yielded a sample of 256 survey events. The total number of bats counted was 20,211 of which *Hipposideros speoris* was the most common (9,715) followed by *Rousettus leschenaulti* (5,306), *Taphozous melanopogon* (3,196), *Megaderma lyra* (1,497)

*Tadarida aegyptiaca* (303), *Pipistrellus sp* (144) and *Rhinopoma hardwickii* (50). About 39% of the abundance was found in dark rooms while 51% by walkways around the deity room.

##### Species richness and total abundance were related to the number of buildings in the temple and the availability of dark rooms. Land use elements only had a weak effect, but scrub and grassland, even though were few, are important for bats. We conclude that retaining undisturbed dark rooms with small exits in temples and other dimly lit areas and having natural areas around temples are critical for bat conservation in the area.

Key words: Bats; microhabitat; Hindu temples; India; landscape,

**Introduction**

Many studies suggest that human-modified landscapes with a heterogeneous mosaic of different land-uses can preserve species-rich bat assemblages (Medellín et al. 2000; Moreno and Halffter 2001; Estrada and Coates-Estrada 2002; Harvey et al. 2006; Medina et al. 2007; Barragan et al. 2010; Mendenhall et al. 2014). This is possible with the concurrent availability of microhabitats that bats use as roosting sites ranging from caves, crevices, tree cavities, foliage, and several types of anthropogenic structures including road bridges and buildings (Voigt and Kingston 2016, Meierhofer et al. 2019). These microhabitats being important features of a bat's environment, the selection of specific roost sites can strongly impact the survival and fitness of bats (Vonhof and Barclay, 1996, Humphrey, 1975; Stebbings, 1995). However, emerging challenges such as changing agricultural practices and climate change pose major threats to bat assemblages and populations in human-dominated landscapes (Jones et al. 2009, Frick et al 2019 ) and could negatively impact human populations by altering the ecosystem services that bats provide (Kunz et al. 2011, Williams-Guillén et al 2016). Moreover, bats that roost in anthropogenic structures are considered vulnerable to disturbance, since just like cave roosting species, they often form large, concentrated aggregations attracting attention (Furey et al. 2016).

Most studies on the use of anthropogenic structures by bats have come from urban areas and under temperate conditions (Kunz et al., 2011; Jenkins et al., 1998; Clem, 1993, Fagan et al 2017, Meierhofer et al. 2019). While it is accepted that bats have been poorly studied in the Paleotropics (Meyer et al 2016), the selection of microhabitats and landscape-level influences on their population remains poorly known (McNab, 1982; Ho and Lee, 2003, Kerth et al., 2001). Most studies on bat–habitat relationships are done at fixed spatial scales (Rainho & Palmeirim 2011) but recent studies have shown the importance of scale for bats (Razgour, Hanmer & Jones 2011, Bellamy et al 2013). In tropical South Asia, several species of both insectivorous and frugivorous bats use temples as roosting sites (Bates and Harrison, 1997, Korad 2007). In south India numerous very old (>400y) temples dot the landscape and form important bat habitats (Swamidoss et al. 2012). These temples are made of granite stones and the entrance of the temple has towers (*Gopuram*) usually made of bricks. The temples are not air-conditioned and the temperature inside is stable and warm (320 - 330 C) and fairly constant across the day (personal obs T.Ganesh). Bats enter and leave the roost through numerous vents that are left open for air to circulate inside the

temple.

Bat use of anthropogenic structures, such as temples, also makes it difficult to balance bat conservation with human-bat conflicts. The temples are situated in a human-dominated agricultural matrix where intensive paddy and banana cultivation is done. Agriculture is considered a major threat to bats (Frick et al. 2019) and with irrigated agriculture spreading to dry areas in the region, threats to bat population can be expected to rise in the future along with a predicted increase in rains (Tamil Nadu State Action Plan for Climate Change). Populations of bats in many temples have dwindled (Vanitharani 2004, Mahandran et al.2015) and many are also directly killed during temple renovation (Addline et al 2004). The changing attitude of people towards bats has led many temples to be made “bat-proof” by placing nets and power washing roost areas with chlorinated chemicals to prevent bats from colonizing. The recent COVID-19 pandemic has further made them more vulnerable to disturbance and direct persecution. In India, only two out of 120 species of bats comes under the strict Wildlife Protection Act 1972, while others even though are threatened under IUCN category, remain unprotected.

Management decisions for bats and buildings, including the timing of maintenance activities, restriction of human use, and bat deterrence or exclusion, require information on the roosting habits of temple-roosting bats. Studies in temperate regions have shown how features of bridges and buildings can help identify sites for conservation and thereby reduce bat-human conflict (Fagan et al 2017, Meierhofer et 2019) and such efforts are needed for conserving bats in tropical regions. It’s therefore essential to understand what attributes of temples are important for bats and how such information can help bat conservation efforts in temple landscapes.

More than 50% of the 35 bat species found in Tamil Nadu state dwell in caves and old buildings (Bates and Harrison 1997). All are insectivores, except *Rousettus leschenaultia,* a frugivore. In the paddy agriculture dominated landscape of south India, ancient temples are often the only habitat for several bat species. They provide critical ecosystem services for agriculture and in south Asia can save up to US$1.2 million per year from insect depredation (Wagner et al. 2014) and consume large quantities of mosquitoes, thus providing human health benefits (Sophia 2010). Despite the numerous threats and the importance of bats in providing key ecosystem services in human-dominated landscapes (Wagner et al. 2014, Thet and Mya 2015), there is no systematic study on the ecology of bats in temples while behavioural studies have been carried out on them in South India (Audet et al. 1991, Leippert et al.2000).

Moreover, with populations of bats dwindling, no attempt to monitor bats in the temples or how important temples are in an agricultural matrix to bats has been investigated. We initiated a bat monitoring program in 2012 in temples of south India to understand why bats use temples and how the population is responding to disturbance and land-use change. In this study, we identify temples used by bats for roosting in a semi-arid region characterized by the winter monsoon (Oct to Dec) and agriculture dominated by irrigated rice paddy and banana for 6-8 months a year. The rest of the time the land is fallow in most places. We recorded the species composition, rarity, and abundance of bats in temples and identified factors that promote or dissuade bat colonization such as microhabitat availability, disturbance in temples and landscape features surrounding the temples. While much of the bat diversity in human-dominated landscapes can be attributed to a combination of habitat diversity and roost site availability in a tropical monoculture areas with rice paddies and banana and low habitat heterogeneity, we compared the role of land use elements and availability of microhabitats on bat species richness and abundances. In this paper, we test three major hypotheses: 1.Disturbance at the roost negatively affects species richness and abundance of bats, 2. Roost characteristics are more important than landscape features for bats to colonize temples and 3. In a homogeneous monoculture cropping, species occurrence and abundance are independent of spatial availability of habitats around roosts.

**Materials and Methods**

**Study area**

The study was carried out in Tirunelveli & Thoothukudi Districts of Tamil Nadu state in South India. The region is semi-arid and experiences the northeast winter monsoons between Oct-Dec, which bring in 70% of the annual rainfall. The southwest monsoon which is obstructed by the Western Ghats mountains brings in strong winds and light rain during June – Sep. The rest of the months are dry. Several ancient temples built during the Chola and Pandya dynasties 500 to 1000 years back exist in the districts along the perennial Tamiraparani river and its tributaries. The river and numerous interconnected village ponds are the lifelines for paddy and banana dominated agriculture, supporting a dense human population in the semi-arid landscape. The ancient temples are built of large granite stones and provide a habitat for several bat species.

There are over 4041 temples (HR&CE, 2020) in the study area with 2-3 villages having one ancient temple. The typical structure of the temples includes a tower that stands at the four entrances and one over the deity, a deity chamber lit by an oil lamp and an electric bulb, a walkway surrounding the deity chamber for walking and prayer by devotees, and several rooms including storerooms and a kitchen, which are dark and rarely frequented by people. The temples are usually located towards the corner of a village or town.

**Temple selection**

We selected 59 temples from the Tamiraparani basin and surveyed them with 2-4 personnel between 06:00-12:00 h and again between 16:00 to 17:00h to detect the presence of bats. The survey was done from May to Dec and the same temples were visited in 2012, 2013, 2014, 2018, 2019, for a total of 246 surveys.

All species of both *Yinpterochiroptera* and *Yangochiroptera* bats found roosting inside the temples were recorded. The presence of bats in the temple was identified by several signs such as droppings and smell, interviewing local people, temple priests, and caretakers. Later dark corners, temple roof, abandoned & isolated rooms inside the temple and the temple tower were all scanned for chiropteran presence with a red LED light. Once bat colonies were detected, we counted the number of individuals and in some cases, especially in closed towers, we took photographs to estimate them.

**Temple features**

a. **Microhabitat**

There were multiple locations inside temples where bats could roost: closed rooms with few small openings, very low natural light and not frequently used by people were considered dark rooms; stone pillars that support the stone roof with gaps created between the stone joints was crevices; temple towers, which were conical structures built over the entrance or the deity and wide walking space around the deity room covered by a stone roof are walkways. We also counted the number of independent buildings in the temple complex, as bats could move between them during the day due to disturbance. In 2012 we measured temperature and humidity while sampling for bats and found no difference between temples and therefore did not consider in the analysis. We could not set dataloggers for continuous monitoring because of objections by the temple authorities to placing any devices inside temples. Temple size estimated based on visual observation; those with deity rooms and walkways were considered as small, those with deity rooms, walkways and towers considered as medium-sized, and those having deity rooms, multiple towers, and two walkways (inside and outside) were considered large. We also recorded the number of trees in the temple complex and open wells used by bats.

b. **Disturbance**

Two types of disturbance that directly impact bats at the roost are structural impacts like renovation of the temple premises and human presence. Renovation of temples like power washing the walls, reconstructing parts of the building or any other maintenance work was recorded on a scale from 0 to 1:zero being no renovation activities in the temple and 1 when it was present. Information on visitors to the temple, obtained from temple authorities and scaled from 1 to 3:three pertains to large crowds and 1 attracting few people.

**Land-use features**

Land-use features can be easily measured from satellite-based land-use classification, however in this case we could not do that for each sampling period because of high cloud cover during that time. We, therefore, used the composite analysis of land cover available using high- resolution (10m) Esri data for 2020 (Karra et al.2021) to identify major land uses in the region

around temples. We could not obtain such information for each sampling year. The land use was categorized as water, scrub, grassland, bare ground, flooded area, crop, urban and tree-covered areas (Karra et al.2021). We combined bare ground and grassland as grassland habitat and water and flooded area as water habitat after verifying the actual land-use on the ground and using Google Earth. Crop availability each season was obtained from Google Earth images when available and further substantiated by informal interviews with farmers around each temple. If crops were present, we used the Esri calculated value of crop area in the analysis and if not, we considered it as zero. The extent of other land uses has not changed much during the sampling period based on the data available with the district administration (Statistical handbook of Tirunelveli district 2009-2019).

Since the spatial scale is important for bat foraging (Bellamy and Scot 2013) we categorized land use at 500m, 1km, 3km and 5km from the temple based on the foraging distance of bats from their roost (Audet et al 1991). We also recorded trees and water availability (open wells) which are often used by bats in the temple complex, for land-use analysis at 0km level.

**Species identification and abundance**

Bats were identified to species based on detailed photographs taken in the temple using the bat identification guide by Bates and Harrison (2001).

Bats were counted at the roost when they were least active (late morning and early evenings) using a flashlight with red filters. Where possible, photographs were taken as soon as bat aggregations were noticed in a particular microhabitat inside the temple and later counted to estimate abundance (Carpenter, 2014). Several pictures were taken in quick succession to estimate and avoid bias in counting. Binoculars were used to see the species characteristics for identification. We could not do a roost exit count in the evening as it was not logistically possible to be in each temple at the right time for the count.

**Data analysis**

If more than one roost was found in a temple, we pooled roost counts and recorded total species richness for the temple. We constructed a linear model to explain the species richness and abundance of bats in the temples based on the microhabitat and landscape features. We based the variables used in our linear models on our observations made in 2012. We eliminated variables that had were strongly correlated (>0.70) with other variables to reduce variable redundancy and

retained ones that are biologically meaningful. We related the total species richness and abundance (counts) of bats at the temple with temple variables and landscape elements using mixed models. Since temples were sampled more than once and repeated measurements on the same temple are often correlated, we accounted for this by using random effects in the mixed models (Bolker et al., 2009). We used lmer package in R for species richness as it was normally distributed and since the bat counts were overdispersed, we used a negative binomial with glmmadmb package in R (Bolker et al 2012).We considered temple ID and year as a random factor in the model and temple characteristics, disturbance and land use elements as fixed factors. We used the function *dredge* in MuMIn package (version 1.43.17) in R to obtain all possible combinations of models using the select variables. We fitted two models, one for temple characteristics with disturbance and the other for land use elements at 5 spatial scales; 0m, 500m, 1km, 3km and 5km. The models were ranked using Akaike's Information Criterion corrected for sample size and model weights. Model averaging was done for closely related models (Mazerolle 2020) using model selection criteria in MuMIn package (version 1.43.17) in R. We calculated model-averaged parameter estimates and unconditional standard errors to assess the relative importance of each variable and account for model uncertainty (Burnham and Anderson 2002). We considered only parameters with 95% confidence intervals not overlapping with 0 to be informative (Payton et al. 2003). We tested for differences in microhabitats and land-use between species using Kruskal Wallis and Mann Whitney tests. Data analysis and statistical tests were carried out using Microsoft Excel®, PAST® and R version 4.

**Results**

**Species richness and abundance**

Forty-seven of the 59 temples surveyed housed bats and 51 of these had multiple roosts and only 8 had a single roost across years. The total number of roosts sampled was 351 across 246 surveys in 5 years. Seven species of bats were recorded of which six were Yinpterochiroptera and one *Rousettus leschenaulti* was a Yangochiroptera **(Table 1)**. Among the Yinpterochiroptera, *Hipposideros speoris* was the most frequently seen species found in 135 (38%) roosts sampled followed by *Taphozous melanopogon (58), Megaderma lyra (29) Tadarida aegyptiaca (26) Rousettus leschenaulti (22), Pipistrellus sp (9) and Rhinopoma hardwickii (7)*. Forty-five percent of the temples had only one species, 34% had two species, 15% had three species and 6% had four species of bats. In all 20,211 bats were counted of which *Hipposideros speoris* (HS) was the most common (9,715, Range 2-1150) across temples followed by *Rousettus leschenaultia* (RL; 5,306, 1-2946), *Taphozous melanopogon* (TM; 3,196, 1-1165), *Megaderma lyra* (ML; 1,497, range 20-605), *Tadarida aegyptiaca* (TE; 303, 3-84), *Pipistrellus sp* (PS; 144) and *Rhinopoma hardwickii* (RH;50). None of the bat species recorded is “threatened” as per the International Union for Conservation of Nature (IUCN).

**Table 1. Species characteristics and IUCN status of bats recorded in temples. The breeding season in parenthesis is based on the present study.**

Species name

**IUCN	Diet	Breeding**

**season**

**Roosting habitat**

***Rousettus***	LC	Flowers and fruits	Nov-March	Temples, old buildings, caves

***leschenaultii***

***Rhinopoma hardwickii***

LC	Moths, neuropteran insects and beetles	Feb-Mar	Crevices, roof, houses, temples,

boulders

***Hipposideros speoris***

LC	Beetles, termites, low flying insects and flies	Dec-Mar

(Dec)

crevices in hills, caves, caverns, disused buildings, tunnels and temples

***Megaderma lyra***

LC	Insects, reptiles, fishes and birds	Nov-Apr	caves, temples, forts, dilapidated old

buildings, underground tunnels, old cow sheds, grain godowns, shallow

soap stone mines and attics of houses

***Taphozous melanopogon***

LC	Insects	Jan-June

(Dec-June)

ruins temples and caves, dark dungeons in the old fort

***Tadarida aegyptiaca***

LC	I Caterpillar, spider, water beetle, ground- dwelling insects

June-Sep	Crevices in cliff faces, crevices in large

piles of rocks and boulders, narrow spaces formed by slabs of stone leanings against walls, the expansion joints at the top of supporting pillars in modern grain store, narrow cracks in a pillar of old mosques and crevices

in buildings and old forts

***Pipistrellus tenuis***

LC	Small insects, beetle, cockroach, termite, grasshopper

Feb-march, July-Aug (Dec)

Roofs of bungalows, holes and crevices in walls, hollow branches of trees, dead leaves of trees

#### Bats and Roost characteristics

##### Four microhabitats inside the temple were used for roosting by the seven species (**Fig 1**). About 39% of the total abundance was in dark rooms while 51% of bats were in walkways around the deity room, which are the main microhabitats for 5 out of 7 species. *Tadarida aegyptiaca, T. melanopogon* and *Pipistrellus sp* Crevices preferred pillars.

**Fig 1. Percentage of microhabitats used by the different species of bats for roosting.**

In terms of species occupancy, *H. speoris* occupied three out of four microhabitats, but most commonly dark rooms; walkways were used only when darkrooms were not available or disturbed. Likewise, *M. lyra*, *T. melanopogon* and *R. hardwickii* were found in two microhabitats (walkways and crevices) while *T. aegyptiaca* was found only between pillars(crevices), and *Pipistrellus sp* found only in roof crevices. The fruit bat *R. leschenaulti* preferred walkways.

#### Temple and disturbance.

##### The number of buildings and dark room availability in the temple complex significantly affected species richness and bat abundance (counts) in temples **(Fig 2, Table 2).** The top lmer model explained 69% of the variation in species richness of which 58% were explained by the fixed factors.

**Fig 2. Effect plots showing the relationship between species richness, abundance and number of buildings and dark rooms in temples**

**Table 2: Effects of Temple characteristics on species richness (lmer model) and bat abundances( glmmadmb model). Temple id and year are included as random factors.**

Temple model with disturbance

| **Species richness** |  | | | | | | | | | |
| --- | --- | --- | --- | --- | --- | --- | --- | --- | --- | --- |
|  | Estimate | 2.500 | 97.500 | Std. | Models | d | logLik | AICc | delta | weigh |
|  |  |  |  | Error |  | f |  |  |  | t |
| **(Intercept)** | -0.052 | -0.291 | 0.187 | 0.122 | BLD+DR+RE | 7 | -171.90 | 358.27 | 0.000 | 0.600 |
|  |  |  |  |  | N |  |  |  |  |  |
| **No. buildings** | 0.674 | **0.587** | **0.761** | 0.044 | BLD+REN | 6 | -173.38 | 359.11 | 0.850 | 0.400 |
| **Dark rooms** | 0.246 | **0.044** | **0.448** | 0.103 |  |  |  |  |  |  |
| **Renovation** | -0.364 | **-0.524** | **-0.204** | 0.081 |  |  |  |  |  |  |
|  |  |  |  |  |  |  |  |  |  |  |
| **Bat Abundance** |  |  |  |  |  |  |  |  |  |  |
| **(Intercept)** | 1.817 | -0.498 | 4.132 | 1.177 | BLD+DR+RE | 8 | -1118.67 | 2253.94 | 0.000 | 0.570 |
|  |  |  |  |  | N+TWR+WA |  |  |  |  |  |
|  |  |  |  |  | Y |  |  |  |  |  |
| **No. buildings** | 0.802 | **0.397** | **1.206** | 0.206 | BLD+DR+RE | 9 | -1117.88 | 2254.52 | 0.570 | 0.430 |
|  |  |  |  |  | N+VIS+WAY |  |  |  |  |  |
| **Dark rooms** | 1.597 | **0.541** | **2.654** | 0.536 |  |  |  |  |  |  |
| **walkway** | 1.678 | -0.404 | 3.761 | 1.057 |  |  |  |  |  |  |
| **Renovation** | -0.319 | -1.016 | 0.378 | 0.354 |  |  |  |  |  |  |
| **Visitors** | -0.869 | **-1.473** | **-0.265** | 0.307 |  |  |  |  |  |  |
| **Tower** | 0.349 | -0.532 | 1.230 | 0.447 |  |  |  |  |  |  |
| ***Hiposederous speoris*** | | | | | | | | | | |
| **(Intercept)** | -0.923 | -5.439 | 3.594 | 2.296 | BLD+DR+RE | 8 | -766.21 | 1549.02 | 0.000 | 0.400 |
|  |  |  |  |  | N+VIS |  |  |  |  |  |
| **No. buildings** | 0.769 | **0.090** | **1.448** | 0.345 | DR+VIS | 6 | -768.88 | 1550.12 | 1.090 | 0.230 |
| **Dark rooms** | 2.731 | **0.367** | **5.095** | 1.200 | BLD+DR+RE | 1 | -764.79 | 1550.51 | 1.480 | 0.190 |
|  |  |  |  |  | N+TWR+VIS+ | 0 |  |  |  |  |
|  |  |  |  |  | WAY |  |  |  |  |  |
| **Renovation** | -0.323 | -1.509 | 0.863 | 0.602 | BLD+DR+RE N+TWR+VIS | 9 | -765.92 | 1550.60 | 1.580 | 0.180 |
| **Visitors** | -0.559 | -1.877 | 0.759 | 0.669 |  |  |  |  |  |  |
| **walkway** | 3.631 | -0.948 | 8.209 | 2.324 |  |  |  |  |  |  |
| **Tower** | -0.748 | -2.566 | 1.069 | 0.922 |  |  |  |  |  |  |
| ***Megaderma lyra*** |  |  |  |  |  |  |  |  |  |  |
| **(Intercept)** | -10.230 | -16.610 | -3.849 | 3.243 | BLD+REN | 6 | -176.56 | 365.46 | 0.000 | 0.660 |
| **No. buildings** | 0.982 | -0.162 | 2.126 | 0.581 | BLD+VIS+RE | 7 | -176.16 | 366.78 | 1.320 | 0.340 |
|  |  |  |  |  | N |  |  |  |  |  |
| **Renovation** | -3.462 | **-6.074** | **-0.850** | 1.326 |  |  |  |  |  |  |
| **Visitors** | -1.769 | -6.113 | 2.575 | 2.205 |  |  |  |  |  |  |

***Tadarida aegyptiaca***

**(Intercept)**	-6.086	-12.775	0.603	3.413	BLD+REN+VI

S

7	-129.49	273.50	0.000	0.567

**Renovation**	-3.723	**-7.216	-0.230**	1.782

**visitors**	-2.231	-6.036	1.575	1.942

**No. buildings**	1.083	**0.145	2.021**	0.479

***Taphozous melanopogon***

**(Intercept)**	-14.690	-25.594	-3.786	5.538	BLD+REN+VI

7	-265.48	545.42	0.000	0.680

S

**No. buildings**	0.758	**0.132	1.384**	0.318	BLD+DR+RE

N+VIS

8	-265.14	546.89	1.460	0.320

**Renovation**	-1.181	-2.659	0.297	0.750

**visitors**	2.312	-1.594	6.217	1.983

**Dark rooms**	2.576	-4.351	9.503	3.516

***Rousettus leschenaulti***

| **(Intercept)** | -9.636 | -16.172 | -3.100 | 3.318 | BLD+VIS | 6 | -190.46 | 393.27 | 0.000 | 0.440 |
| --- | --- | --- | --- | --- | --- | --- | --- | --- | --- | --- |
| **No. buildings** | 1.163 | -1.296 | 3.622 | 1.248 | DR+REN | 6 | -191.10 | 394.55 | 1.290 | 0.230 |
| **visitors** | -0.913 | -3.988 | 2.161 | 1.561 | BLD+REN+VI S | 7 | -190.36 | 395.18 | 1.920 | 0.340 |
| **Dark rooms** | 0.687 | -4.871 | 6.245 | 2.822 |  |  |  |  |  |  |
| **Renovation** | 0.667 | -2.645 | 3.979 | 1.681 |  |  |  |  |  |  |

Renovation of temple structures had a strong negative influence on bat species richness while the scale of visitors to the temple negatively affected abundance (**Fig 3**). Species response to microhabitat and disturbance varied (**Table 2**). *Hiposederous speoris* abundance was positively influenced by dark rooms and the number of buildings but not by any disturbance factors.

Counts of *T.melanopogon* and *T.aegyptica* were also positively related to the number of buildings in the temple but were affected negatively by renovation. *M.lyra* showed a negative response to renovation while *R.leschenaulti* was not affected by any temple characteristics or disturbance.

**Fig 3. Effects plots showing the relationship of disturbance on species richness and abundance.**

**Landscape**

The percentage of land-use around the temples changed with distance. At 5km from the temple crops (63%) dominate followed by urban areas (13%), scrub (12%), trees (7%), water (5%), grass and bare areas (0.23%). At 500m crops (54%) continued to dominate followed by urban areas (36%), scrub (0.7%), trees (3%), water (6%) grass and bare (0.09%). Since temples are close to settlements, urban area is high near the temple and crop, trees, scrub and grass areas are less **(Fig 5)**. Grassland around the temples at 3km positively affected species richness. Scrub availability positively affected the abundance while urban areas had a negative effect at 5km. Trees in the temple complex had a positive effect on species richness (**Table 3**). Scrub availability also had a positive effect on *Hiposederous speoris* abundance across the distance interval. Trees had a negative effect and were strongest at 1km (**Table 3**). *M. lyra* and *T. aegyptica* both were negatively affected by crops up to 5km while. *T. melanopogon* and *R. leschenaulti* did not show any effect of land use on their roost abundance.

**Fig 4. High resolution (10m) land-use around the temples sampled. Land-use classification obtained from Karra et al 2021. In the analysis grass and bare areas, and water and flooded areas were combined. See data analysis for more details.**

**Table 3: Species response to land-use elements. Model averaged parameter estimates of top models (<2 ΔAICc) with non-overlapping confidence intervals shown in the last column***

| **Species/Abun dance** | **Distance (m)** | **Parameters** | **Estimate** | **2.50%** | **97.50**  **%** | **Std.Err** | **Strong association** |
| --- | --- | --- | --- | --- | --- | --- | --- |
| Species richness | 0 | Intercept | 0.829 | 0.503 | 1.155 | 0.166 |  |
|  |  | Water | 0.029 | -0.144 | 0.202 | 0.088 |  |
|  |  | Trees | 0.006 | **0.002** | **0.010** | 0.002 | * |
|  | 500 | Intercept | 0.937 | 0.694 | 1.180 | 0.123 |  |
|  |  | Grassland | -0.071 | -0.398 | 0.255 | 0.166 |  |
|  | 1000 | Intercept | 0.940 | 0.695 | 1.184 | 0.124 |  |
|  |  | Grassland | -0.197 | -0.982 | 0.588 | 0.399 |  |
|  | 3000 | Intercept | 0.793 | 0.541 | 1.071 | 0.128 |  |
|  |  | Grassland | 0.653 | **0.190** | **1.120** | 0.235 | * |
|  | 5000 | Intercept | 0.861 | 0.548 | 1.173 | 0.159 |  |
|  |  | Grassland | 0.618 | -0.205 | 1.441 | 0.418 |  |
| Abundance | 0 | Intercept | 2.917 | 1.963 | 3.870 | 0.484 |  |
|  |  | Trees | 0.017 | **0.000** | **0.033** | 0.008 | * |
|  |  | Water | -0.174 | -0.804 | 0.456 | 0.320 |  |
|  | 500 | Intercept | 3.307 | 2.587 | 4.027 | 0.366 |  |
|  |  | Trees | -0.032 | -0.097 | 0.032 | 0.033 |  |
|  |  | Grassland | 0.184 | -1.330 | 1.698 | 0.769 |  |
|  |  | Water | -0.001 | -0.061 | 0.059 | 0.030 |  |
|  |  | Scrub | 0.142 | -0.088 | 0.371 | 0.116 |  |
|  | 1000 | Intercept | 3.229 | 1.964 | 4.495 | 0.643 |  |
|  |  | Scrub | 0.176 | 0.057 | 0.294 | 0.060 |  |
|  |  | Crop | 0.002 | -0.007 | 0.011 | 0.005 |  |
|  |  | Trees | -0.144 | -0.237 | -0.051 | 0.047 | * |
|  |  | Urban | 0.016 | -0.018 | 0.051 | 0.018 |  |
|  |  | Water | -0.019 | -0.089 | 0.051 | 0.035 |  |
|  |  | Grassland | -0.094 | -3.954 | 3.767 | 1.960 |  |
|  | 3000 | Intercept | 3.282 | 2.007 | 4.557 | 0.648 |  |
|  |  | Scrub | 0.066 | -0.004 | 0.136 | 0.036 |  |
|  |  | Trees | -0.064 | -0.133 | 0.004 | 0.035 |  |
|  |  | Grassland | 0.291 | -1.387 | 1.968 | 0.851 |  |
|  |  | Water | -0.084 | -0.289 | 0.120 | 0.104 |  |
|  |  | Urban | 0.035 | -0.059 | 0.129 | 0.048 |  |
|  | 5000 | Intercept | 7.065 | 3.306 | 10.824 | 1.913 |  |
|  |  | Water | -0.498 | -0.864 | -0.132 | 0.186 | * |
|  |  | Urban | -0.139 | -0.267 | -0.010 | 0.065 | * |
|  |  | Trees | -0.077 | -0.137 | -0.017 | 0.030 | * |
|  |  | Crop | 0.001 | -0.008 | 0.011 | 0.005 |  |
|  |  | Grassland | -0.023 | -3.133 | 3.087 | 1.579 |  |
|  |  | Scrub | 0.069 | 0.002 | 0.137 | 0.034 | * |
| *Hiposederous speoris* | 0 | Intercept | 1.591 | 0.171 | 3.011 | 0.724 |  |
|  |  | Trees | -0.004 | -0.032 | 0.025 | 0.015 |  |
|  | 500 | Intercept | 1.681 | -0.070 | 3.431 | 0.890 |  |
|  |  | Grassland | 1.091 | -1.123 | 3.304 | 1.124 |  |

|  |  | Urban | -0.036 | -0.080 | 0.008 | 0.022 |  |
| --- | --- | --- | --- | --- | --- | --- | --- |
|  |  | Scrub | 0.246 | -0.128 | 0.621 | 0.190 |  |
|  |  | Water | 0.017 | -0.085 | 0.119 | 0.052 |  |
|  |  | Trees | -0.005 | -0.112 | 0.102 | 0.054 |  |
|  | 1000 | Intercept | 1.583 | -0.295 | 3.460 | 0.954 |  |
|  |  | Scrub | 0.275 | 0.059 | 0.492 | 0.110 | * |
|  |  | Crop | 0.001 | -0.014 | 0.016 | 0.007 |  |
|  |  | Trees | -0.173 | -0.333 | -0.013 | 0.081 | * |
|  |  | Urban | -0.026 | -0.089 | 0.037 | 0.032 |  |
|  |  | Water | 0.015 | -0.106 | 0.135 | 0.061 |  |
|  |  | Grassland | 0.444 | -5.734 | 6.622 | 3.136 |  |
|  | 3000 | Intercept | 0.672 | -0.668 | 2.012 | 0.684 |  |
|  |  | Scrub | 0.124 | 0.009 | 0.240 | 0.059 | * |
|  | 5000 | Intercept | 2.098 | -2.593 | 6.789 | 2.389 |  |
|  |  | Grassland | -0.595 | -6.040 | 4.850 | 2.765 |  |
|  |  | Scrub | 0.112 | -0.007 | 0.231 | 0.060 |  |
|  |  | Water | -0.275 | -0.972 | 0.423 | 0.354 |  |
|  |  | Urban | -0.197 | -0.446 | 0.051 | 0.126 |  |
|  |  | Trees | -0.002 | -0.101 | 0.097 | 0.050 |  |
| *Megaderma lyra* | 0 | Intercept | -10.086 | -14.399 | -6.069 | 2.219 |  |
|  |  | Trees | 0.006 | -0.057 | 0.068 | 0.032 |  |
|  |  | Water | -0.064 | -1.373 | 1.648 | 1.252 |  |
|  | 500 | Intercept | -9.165 | -12.658 | -5.673 | 1.773 |  |
|  |  | Grassland | -1.701 | -22.185 | 18.783 | 10.399 |  |
|  |  | Crop | -0.038 | -0.074 | -0.002 | 0.018 |  |
|  |  | Water | -0.053 | -0.387 | 0.282 | 0.170 |  |
|  | 1000 | Intercept | -8.910 | -12.676 | -5.143 | 1.912 |  |
|  |  | Grassland | -2.381 | -29.684 | 24.921 | 13.860 |  |
|  |  | Crop | -0.031 | -0.060 | -0.001 | 0.015 |  |
|  |  | Water | -0.130 | -0.769 | 0.509 | 0.324 |  |
|  | 3000 | Intercept | -9.571 | -13.736 | -5.407 | 2.115 |  |
|  |  | Grassland | -0.325 | -9.084 | 8.435 | 4.447 |  |
|  |  | Crop | -0.028 | -0.059 | 0.003 | 0.016 |  |
|  |  | Water | -0.168 | -1.223 | 0.887 | 0.536 |  |
|  | 5000 | Intercept | -9.432 | -14.681 | -4.182 | 2.666 |  |
|  |  | Grassland | -1.791 | -16.318 | 12.736 | 7.375 |  |
|  |  | Crop | -0.027 | -0.061 | 0.006 | 0.017 |  |
|  |  | Water | -0.103 | -1.593 | 1.386 | 0.756 |  |
|  |  | Trees | 0.008 | -0.220 | 0.236 | 0.116 |  |
| *Rousettus leschenaulti* | 0 | Intercept | -10.086 | -14.457 | -5.715 | 2.219 |  |
|  |  | Trees | 0.006 | -0.057 | 0.068 | 0.032 |  |
|  |  | Water | -0.064 | -2.531 | 2.403 | 1.252 |  |
|  | 500 | Intercept | -9.441 | -13.064 | -5.819 | 1.839 |  |
|  |  | Trees | -0.354 | -1.823 | 1.115 | 0.746 |  |
|  |  | Grassland | -2.717 | -28.261 | 22.827 | 12.968 |  |

|  |  | Water | 0.029 | -0.167 | 0.224 | 0.099 |  |
| --- | --- | --- | --- | --- | --- | --- | --- |
|  | 1000 | Intercept | -9.219 | -13.146 | -5.291 | 1.994 |  |
|  |  | Trees | -0.381 | -1.805 | 1.043 | 0.723 |  |
|  |  | Water | 0.013 | -0.251 | 0.277 | 0.134 |  |
|  | 3000 | Intercept | -9.805 | -14.066 | -5.543 | 2.164 |  |
|  |  | Trees | -0.159 | -0.928 | 0.610 | 0.391 |  |
|  |  | Urban | 0.078 | -0.220 | 0.375 | 0.151 |  |
|  |  | Grassland | 1.390 | -4.757 | 7.536 | 3.120 |  |
|  | 5000 | Intercept | -10.148 | -15.311 | -4.984 | 2.622 |  |
|  |  | Water | 0.241 | -0.913 | 1.395 | 0.586 |  |
|  |  | Grassland | 2.023 | -8.862 | 12.908 | 5.526 |  |
|  |  | Trees | -0.049 | -0.391 | 0.294 | 0.174 |  |
|  |  | Crop | -0.010 | -0.054 | 0.035 | 0.023 |  |
|  |  | Scrub | 0.054 | -0.207 | 0.315 | 0.132 |  |
| *Tadarida aegyptiaca* | 0 | Intercept | -9.608 | -14.070 | -5.146 | 2.266 |  |
|  |  | Water | 0.555 | -1.653 | 2.763 | 1.121 |  |
|  |  | Trees | 0.001 | -0.057 | 0.059 | 0.030 |  |
|  | 500 | Intercept | -8.367 | -12.152 | -4.581 | 1.922 | * |
|  |  | Grassland | -1.819 | -19.858 | 16.221 | 9.158 |  |
|  |  | Crop | -0.023 | -0.046 | 0.000 | 0.012 | * |
|  |  | Urban | -0.010 | -0.109 | 0.088 | 0.050 |  |
|  |  | Trees | -0.317 | -1.849 | 1.215 | 0.778 |  |
|  |  | Water | -0.085 | -0.484 | 0.314 | 0.203 |  |
|  | 1000 | Intercept | -7.652 | -11.239 | -4.065 | 1.821 |  |
|  |  | Grassland | -24.772 | - 149.99  6 | 100.45  3 | 63.572 |  |
|  |  | Crop | -0.022 | -0.043 | -0.001 | 0.010 |  |
|  |  | Water | -0.081 | -0.506 | 0.344 | 0.216 |  |
|  | 3000 | Intercept | -8.235 | -13.475 | -2.995 | 2.663 |  |
|  |  | Grassland | 0.812 | -5.570 | 7.193 | 3.240 |  |
|  |  | Crop | -0.022 | -0.040 | -0.004 | 0.009 |  |
|  |  | Water | -0.487 | -1.728 | 0.755 | 0.630 |  |
|  |  | Urban | 0.061 | -0.228 | 0.350 | 0.147 |  |
|  |  | Trees | -0.031 | -0.344 | 0.283 | 0.159 |  |
|  | 5000 | Intercept | -8.291 | -14.453 | -2.129 | 3.130 |  |
|  |  | Grassland | -0.488 | -12.427 | 11.452 | 6.061 |  |
|  |  | Crop | -0.024 | -0.044 | -0.004 | 0.010 | * |
|  |  | Water | -0.368 | -1.794 | 1.058 | 0.724 |  |
|  |  | Urban | 0.117 | -0.338 | 0.572 | 0.231 |  |
|  |  | Trees | -0.022 | -0.264 | 0.220 | 0.123 |  |
| *Taphozous melanopogon* |  | Intercept | -8.476 | -12.411 | -4.542 | 1.998 |  |
|  |  | Water | -0.997 | -3.967 | 1.973 | 1.508 |  |
|  |  | Trees | 0.010 | -0.051 | 0.071 | 0.031 |  |
|  | 500 | Intercept | -9.273 | -12.846 | -5.699 | 1.815 |  |

|  |  | Trees | -0.096 | -0.837 | 0.645 | 0.376 |  |
| --- | --- | --- | --- | --- | --- | --- | --- |
|  |  | Water | 0.029 | -0.176 | 0.233 | 0.104 |  |
|  |  | Grassland | 0.322 | -3.117 | 3.760 | 1.746 |  |
|  |  | Urban | 0.047 | -0.052 | 0.147 | 0.051 |  |
|  |  | Crop | -0.007 | -0.036 | 0.021 | 0.015 |  |
|  | 1000 | Intercept | -9.137 | -12.374 | -5.901 | 1.644 |  |
|  |  | Grassland | 1.637 | -5.136 | 8.410 | 3.438 |  |
|  |  | Water | 0.056 | -0.195 | 0.308 | 0.128 |  |
|  |  | Trees | -0.032 | -0.425 | 0.362 | 0.200 |  |
|  |  | Crop | -0.012 | -0.030 | 0.006 | 0.009 |  |
|  |  | Urban | 0.029 | -0.100 | 0.157 | 0.065 |  |
|  | 3000 | Intercept | -8.635 | -13.813 | -3.456 | 2.633 |  |
|  |  | Grassland | 7.892 | -1.886 | 17.670 | 4.964 |  |
|  |  | Urban | -0.208 | -0.693 | 0.276 | 0.246 |  |
|  |  | Water | 0.302 | -0.337 | 0.940 | 0.324 |  |
|  |  | Scrub | 0.050 | -0.162 | 0.261 | 0.107 |  |
|  |  | Trees | -0.059 | -0.502 | 0.384 | 0.225 |  |
|  | 5000 | Intercept | -9.437 | -15.329 | -3.546 | 2.994 |  |
|  |  | Grassland | 5.918 | -7.637 | 19.473 | 6.883 |  |
|  |  | Trees | -0.091 | -0.514 | 0.332 | 0.215 |  |
|  |  | Water | 0.198 | -0.856 | 1.252 | 0.535 |  |
|  |  | Crop | -0.009 | -0.025 | 0.006 | 0.008 |  |
|  |  | Urban | -0.262 | -0.930 | 0.405 | 0.339 |  |
|  |  | Scrub | 0.081 | -0.171 | 0.333 | 0.128 |  |

The availability of roost microhabitats across temples with and without bats was not significantly different (Kruskal Wallis test; D=0.4, p=0.689), **Fig 5**). Of the total temples sampled, 80% of them had bats across all the years and in only 4 of 57 temples we found bats abandoning the temple. Renovation levels were higher in temples with no bats 44% (15/34) compared with temples having bats 35% (16/46). Similarly, visitor levels were also marginally higher in no bat (2.11+0.79) compared to temples with bats (1.89+.71). Land use between bat occupied and not occupied temples showed no differences except for trees in the vicinity of temples was higher in bat occupied sites (Mann-Whitney U=3917 p<0.01) (**Fig 6**).

**Fig 5.Proportional availability of habitats in temples with and without bats.**

**Fig 6. Mean land-use elements around temples with and without bats at various distances. * indicate significant differences at p< 0.05.**

**Discussion**

Why do bats prefer some temples? Bats were found in about 80% of the temples and over 20,211 individuals were counted. Though we recorded only 7 species, the most common species *Hipposideros speoris* (9,715) accorded for nearly half of the population and was found in more than 50% of bat inhabited temples. *Hipposideros speoris* is a very common species across India and Srilanka and occurs in large congregations with high fidelity to roost sites (Selvarathinam and Marimuthu, 2013). The other 6 species are also not rare or threatened and use a multitude of anthropogenic structures to roost (Bates and Harrison, 1997). Except for *T. aegyptiaca, Pipistrellus sp* and *Rhinopoma hardwickii,* species counts of all others ranged between 200 to 450 in each temple and appear to follow the general tendency of bats that roosts in caves and manmade structures, to be highly gregarious (Gaisler, 1979). Crevice roosting *T. aegyptiaca* and *Pipistrellus sp* species may have been underestimated because they are hard to count inside the narrow crevices.

Several species that roost and breed in temples across India are cave-dwelling species that have found temples as suitable alternative habitats (Bates and Harrison, 1997; Leippert et al., 2000). Our results show that temples offer dark, undisturbed roosts and crevices similar to caves and these harbor 6-7 species accounting for 80% of the abundance of bats in the temples. While we have pooled different types of dark spaces available in the temples, the one behind the main door of the temple and through which the temple tower can be accessed is occupied whenever there was no disturbance. These dark areas in old temples have several openings through which bats can easily fly out, a likely reason for their selection by most bat species. Granite stones used to build the ancient temples are stacked together and offers crevices for some species to roost such as the free-tailed bats (*T. aegyptiaca*) which often roost at eye level between pillars. Walkways surrounding the temple *sanctum* where the idol of the god is often are long and dark during the day with a high roof which is a preferred place for the bats like *H. speoris* to remain unnoticed and therefore undisturbed during the morning hours when there is a movement of people. A similar response of cave-dwelling bats from genus *Hipposideros* has been found in Malaysia where they roost in very dark corners and high places (Rajasegarn et al 2018). We did not take species roost height measurements so it’s not evident if species segregate roost according to height but unlike in a cave, many species that roost in dark corners seem to segregate spatially and can vary between temples.

This study demonstrates that disturbance to roosts is a major factor affecting roosting bats as many other similar studies have demonstrated in old buildings under temperate conditions (Kunz and Reynolds 2003, Fagan et al 2017). The effect was less impactful on the abundant *H.speoris* while other species are negatively affected by renovation and visitor levels. Large temples usually have more visitors but also more buildings with more potential roosts and therefore even when disturbance levels increase, bats might persist by moving to other buildings in the temple temporarily. While there could be several other factors affecting bats in temples especially around festivals which can increase noise, lighting, human activity and prayer-related smoke. We did not measure these but should be considered when making management decisions for bat conservation. However, in many cases, these disturbance activities are routine in temples and bats have likely got accustomed to these threats. But the recent i in the renovation of old temples where dark areas are lit, pressure cleaned to remove the odor of bats, painted and ‘bat proofed’ to keep away bats and pigeons from entering the premises is a major one and can effectively prevent bats from colonizing temples (Ganesh et al 2020). With current coronavirus and bat association being misreported by media bats have come under even greater threat and surveillance. Our surveys of bats in the same temples have shown that they have deserted some temples completely because of disturbances such as renovations (Selvarathinam and Marimuthu 2013).While temple characteristics and disturbances are major drivers of bat abundance and occupancy, land use seems to play a less important role across species. Several studies have shown the importance of landscape elements for the presence of bats (Ford et al., 2006; Neubaum et al., 2007, Bellamy and Scot, 2013, Treitler et al. 2016) but in our case, there seems to be no single habitat effect. Fine-scale (10m) mapping of land-use around temples revealed differential use of habitat elements by species but these were either weak effects or had high variations in the effect. The landscape as mentioned earlier is highly homogeneous; crops and urban areas together constitute about 70-80% with scrub, grasslands, water bodies and trees comprising the rest in small proportions. Even though crops were everywhere they had a low negative effect on *M. lyra* and *T. aegyptiaca,* while trees and water availability seem to have a consistent negative influence across species. The availability of scrub and grasslands is critical for species richness and the overall abundance of bats. These are the only two natural habitats available, the rest being tree plantations, invasive *Prosopis juliflora* and artificial wetlands. Even though scrub and grasslands are less than 5% of the area it has a disproportionate and mostly positive effect on all the species except *M. lyra.* The radio-tracking study in south India of the insectivorous *M. lyra* showed it has a very flexible diet and can take prey both from air and ground which could enable it to use multiple habitats but prefers to forage mostly within 500m from temples (Audet et al 1991)*.* The importance of natural habitat is emphasized for many species, including bats (Razgour et al 2011) and this is evident in this study. Effect of water was variable with species like *T. aegyptiaca* seen to prefer water points in the vicinity of the temple but this may not be for water as large open wells in some temples are active sites for foraging. Water availability is important for many species of bats and may drive bat movement and activity in some landscapes ([Geluso and Geluso, 2012](https://www.biorxiv.org/content/10.1101/613943v1.full#ref-40); [Rainho and Palmeirim, 2011](https://www.biorxiv.org/content/10.1101/613943v1.full#ref-84)) Studies in Thailand on similar species have shown insect diversity is less influential than water availability (Suksai and Bumrungsri 2018).

However, in this study, the effect was weak and negative for most species. This could be because there is no constraint for water availability in the region because of the perennial river and extensive irrigation ponds and excessive water can have a negative effect, especially when the sampling was done during the cropping season.

Scale issues are important for many species including bats (Bellamy and Scot, 2013) and in this study at 5km from the roost most of the land-use effects became negligible or the direction of effects changed from positive to negative or vice versa. The extent of natural habitat increases with distance and this may influence bats to forage at larger spatial scales. Temples are located mostly close to human habitations and rivers while natural open dry habitats are further away and bats may be making regular forays as observed in UK bats (Razgour et al 2011). This corroborates with less preference for water and urban areas with distance when more suitable habitats are available.

This study provides a very broad and therefore limited view of land-use effect on bats. More nuanced study and analysis will need to use radio telemetry and acoustic sampling to see how land use elements influence bats. Foraging bats are most strongly associated with variables measured at small spatial scales and distance measures (Bellamy and Scot 2013). For instance, the only study on radio telemetry of temple bats shows 41% of the time the bats foraged 500m from the temples but ranged up to 4km over the night during the summer months. This may be different in rainy months when the general productivity of the habitat is higher and larger use of the landscape may happen as scrub and grassland habitats also become richer in insect fauna during the rains. Further, with more advanced remote sensing tools the land-use change could be included with seasonal changes in bat occurrence. A recent study in the same area using radar images has shown greater grassland area than by conventional means used here (Samrat et al 2021),such an approach would also enable mapping across seasons irrespective of cloud cover.

The current study relies on the most recent high-resolution land-use analysis (Karra et al. 2021) but the study was done in 2012-2019. This mismatch as mentioned earlier may not be very high given the land-use has remained largely the same over this period. However, for small-sized land-use such as natural habitats with less than 5% of the area and which are critical for bats, even small changes can result in large differences for a foraging animal. Further, the dynamics of wetlands has not been incorporated in this study but since it was mostly in the wet season, we assume it to be stable across years but a more nuanced classification of these dynamics around each temple might be important as irrigation systems and water availability are driven not entirely by monsoon patterns in the region (Nikil 2020).

The presence of bats is generally linked to microhabitat or stand-level structural conditions, however, Lewis (1995) also found that roost-site fidelity for bats varied depending on the availability and permanency of the roosts used and suggested that fidelity was high at buildings for these reasons. Supporting this a study on banded *H. speoris* bats from the study area showed 82% roost fidelity throughout the year (Selvarathinam, G Marimuthu 2013). Therefore, the temples that have remained unchanged for centuries provide conditions for high roost fidelity despite changes in the landscape and therefore are more important for bats to persist in the area.

The importance of open uncultivated natural areas such as scrub, grasslands and lightly wooded areas are important for almost all the species of bats and this can have significant conservation importance as natural areas are rapidly diminishing.

**Conservation of bats in temples**

The study has shown that microhabitats within temples such as dark corners that are relatively less disturbed and rooms that were rarely used are essential for the persistence of bats.

Temples with numerous buildings increase the possibility of having dark rooms. The negative effect of disturbance on bats is strong and can override the availability of microhabitats with temples having similar microhabitats not harboring bats because of disturbance. This effect of disturbance is species-specific with the frugivores *R.vleschenaulti* and insectivorous *H.vspeoris* not affected by themMost of the landscape factors were surprisingly not important though the availability of natural areas around temples are crucial but are also limited. While bats are known to provide important pest control services in paddy agriculture the role of paddy in sustaining the bat populations is not clear as crops did not have any significant effect on bat species richness and abundance. This calls for a more nuanced study using radio telemetry and acoustic sampling to understand the use of the landscape by bats since natural areas are likely to be converted in the future with a greater push for irrigated agriculture in the area. To make bat conservation a win-win situation, bats roosting in public

and historical buildings need to be managed to promote their persistence, mitigate human- wildlife conflict, prevent potential zoonotic disease transmission and prevent bat guano and urine from damaging the historical integrity of structures like temples. This can be done with faith groups, temple management and building awareness among the public to develop a comprehensive plan to let bats persist on the landscape and continue to benefit from the ecosystem services they provide rather than ad-hoc elimination of bats in temples.

**Acknowledgement**

We thank Maria,Lise and K.S.Seshadri for help in the field. We are grateful to Dr.R.Ganesan, Dr.M.Soubadra Devy for their help during the initial stages of the work. We also tank Rajkamal, Seshadri, Prashanth and Soubadra for their comments on the MS. We thank Thamizhazhagan for helping in capturing bats and Muthupandi for assistance in the field. Abhishek helped us with landuse analysis and Thalavaipandi drew the map. The Temple authorities and temple staffs in both Tirunelveli and Thoothukudi districts permitted us to do the study. Jermiah Rajanesan, director Dhonavur Fellowship and Maria Joseph, Head Master, Christuraja Hr., Sec., School, Palayamkottai helped us with logistics and we are grateful to them for their help.

**Literature cited**

Addline, EPD, J Vanitharani., S.Rajkumar, and R.T.Sundara. 2004. Government, law and temple bat management, pp-297-301. In: Muthuchelion,K (eds). Biodiversity Resources Management and Sustainable use. Centre for biodiversity and forest studies, school of energy, environment and natural resources, Madurai Kamaraj University, Madurai.

Audet, D., D.Krull, G. Marimuthu, S. Sumithran, and J. B. Singh. 1991. Foraging Behaviour of the Indian False Vampire Bat, *Megaderma lyra* (Chiroptera: Megadermatidae). Biotropica, 23: 63-67.

Barragan, F., C. Lorenzo, A.Moron, M.A. Briones-Salas, and S.Lopez. 2010. Bat and rodent diversity in a fragmented landscape on the Isthmus of Tehuantepec, Oaxaca, Mexico. *Tropical Conservation Science*, *3*(1), pp.1-16.

Bates, P.J.J, and D.L.Harrison. 1997. Bats of the Indian Subcontinent. Harrison Zoological Museum Press, England.

Bolker,B.M, H.Skaug, A. Magnusson, A.Nielsen 2012. Getting started with the glmmADMB package. [http://admb-project.org.](http://admb-project.org/)

Bolker, B.M, M. E. Brooks, C. J. Clark, S. W. Geange, J. R. Poulsen, M. Henry, H. Stevens, J.S

R. White. 2009.Generalized linear mixed models: a practical guide for ecology and evolution, Trends in Ecology & Evolution, Volume 24, Issue 3,Pages 127-135, https://doi.org/10.1016/j.tree.2008.10.008.

Chandrasekaran, M. K. 2005. Time in the living world. Universities Press, Hyderabad, 197 pp.

Carpenter,E.R Gomez,D.L.Waldein and R.E.Sherwin. 2014. Photographic estimation of roosting density of Geoffroys Rousette Fruit Bat *Rousettus amplexi caudatus* (Chiroptera: Pteropodidae) at Monfort Bat Cave, Philippines. Journal of Threatened Taxa*, 6*: 5838-5844.

Cauglin,T, T.Ganesh and M.Lowman. 2012. Sacred fig trees promote frugivore visitation and tree seedling abundance in South India. Current science.102:918-922

Clem, P. D. 1993. Foraging patterns and the use of temporary roosts in female evening bats, *Nycticeius humeralis*, at an Indian maternity colony. Proceedings of the Indian Academy of Science, 102:201- 206.

Deshpande, K. 2012. Assessing diversity and distribution of bats in relation to land-use and anthropogenic threats in the southern Western Ghats, India. *Final Report Submitted to the Rufford Small Grants for Nature Conservation, 30pp*.

##### Estrada, A., and R. Coates- Estrada. 2002. Bats in continuous forest, forest fragments and in an agricultural mosaic habitat-island at Los Tuxtlas, Mexico. *Biological Conservation*, *103*(2), pp.237-245

Fischer, J., J. Stott, B.S. Law, M.D. Adams, and R.I. Forrester. 2009. Designing effective habitat studies: quantifying multiple sources of variability in bat activity. *Acta Chiropterologica*, *11*(1), pp.127-137.

Ford, W. M., J.M. Menzel, M.A. Menzel, J.W. Edwards, and J.C. Kilgo. 2006. Presence and absence of bats across habitat scales in the upper coastal plain of South Carolina. Journal of Wildlife Management*, 70*: 1200-1209.

Furey, N.M. & P.A. Racey. 2016. Conservation ecology of cave bats. In *Bats in the Anthropocene: Conservation of Bats*

*in a Changing World*. C. Voigt & T. Kingston, Eds.: 463–500. Springer International Publishing)

##### Gaisler. J. 1979. Ecology of Bats. In Ecology of Small Mammals (D M Stoddard.ed.) *Chapman and Hall,* London, Pp 2B1-342. *386* pp.

Ganesh, T., A.Saravanan and M.Mathivanan. 2020. Let’s Protect the Bats Where They Live – Including in Our Temples (https://science.thewire.in/environment/bats-covid-19-temples-south- india/)

Geluso, K.N., Geluso, K., 2012. Effects of environmental factors on capture rates of

579 insectivorous bats, 1971–2005. J. Mammal. 93, 161–169. doi:10.1644/11-MAMM-A-107.1

Harvey, C.A., A. Medina, D.M. Sanchez, S. Vilchez, B. Hernandez, J.C. Saenz, J.M. Maes, F. Casanoves, and F.L. Sinclair. 2006. Patterns of animal diversity in different forms of tree cover in agricultural landscapes. *Ecological applications*, *16*(5), pp.1986-1999.

Ho, Y.Y., and L.L. Lee. 2003. Roost selection by Formosan leaf-nosed bats (Hipposideros armiger terasensis). *Zoological Science*, *20*(8), pp.1017-1024.

Humphrey, S.R. 1975. Nursery roosts and community diversity of Nearctic bats. *Journal of mammalogy*, *56*(2), pp.321-346

HR&CE 2020 https://tnhrce.gov.in/hrcehome/temple_list.php

Inger, R. F., H. B. Shaffer, M. Koshy, and R. Bakde. 1987. Ecological structure of a herpetological assemblage in South India. Amphibia-Reptilia, 8: 189–202.

Jenkins, E. V., T. Laine, S. E. Morgan, K. R. Cole, and J. R. Speakman. 1998. Roost selection in the pipistrelle bat, *Pipistrellus pipistrellus* (Chiroptera: Vespertilionidae), in northeast Scotland. Animal Behaviour, 56:909-917

Jones, G., D.S. Jacobs, T.H. Kunz, M.R. Willig, and P.A. Recay. 2009. Carpe noctem: the importance of bats as bioindicators. *Endangered species research*, *8*(1-2), pp.93-115.

Karra,K, C.Kontgis, Z. Statman-Weil, J.Mazzariello, M. Mathis, S. Brumby 2021. Global land use/land cover with Sentinel-2 and deep learning.” IGARSS 2021-2021 IEEE International Geoscience and Remote Sensing Symposium. IEEE, 2021.

Kerth, G., M. Wagner, and B. Konig. 2001. Roosting together, foraging apart: information transfer about food is unlikely to explain sociality in female Bechstein's bats (Myotis bechsteinii). *Behavioral Ecology and Sociobiology*, *50*(3), pp.283-291.

Korad, V., K. Yardi, and R. Raut. 2007. Diversity and distribution of bats in the Western Ghats of India. *Zoos’ Print Journal*, *22*(7), pp.2752-2758.

Kunz, T. H., E. Braun, De Torrez, D. Bauer, T. Lobova, and T.H. Fleming. 2011. Ecosystem services provided by bats. Annals of the New York Academy of Sciences*,* 1223: 1-38.

Leippert, D., W. Goymann, H. Hofer, G. Marimuthu, and J. Balasingh. 2000. Roost-mate communication in adult Indian false vampire bats (*Megaderma lyra*): an indication of individuality in temporal and spectral pattern. Animal Cognition*,* 3: 99-106.

Lewis, S.E. 1995. Roost fidelity of bats: a review. Journal of Mammal, 76, 481–496.

Lianne C. Ball 2002.A Strategy for Describing and Monitoring Bat Habitat. The Journal of Wildlife Management, Vol. 66( 4): 1148-1153.

Mazerolle, MJ (2011). AlCcmodavg: Model selection and multimodel inference based on (Q) AIC (c). *R package version*, *1*.

McNab, B.K. 1982. Evolutionary alternatives in the physiological ecology of bats. In *Ecology of bats* (pp. 151-200). Springer, Boston, MA.

Medellin, R.A., M. Equihua, and M.A. Amin. 2000. Bat diversity and abundance as indicators of disturbance in Neotropical rainforests. *Conservation biology*, *14*(6), pp.1666-1675.

Medina, A., C.A. Harvey, D.S. Merlo, S. Vilchez, and B. Hernandez. 2007. Bat diversity and movement in an agricultural landscape in Matiguás, Nicaragua. *Biotropica*, *39*(1), pp.120-128

Meyer, C.F., M.J. Struebig, and M.R. Willig. 2016. Responses of tropical bats to habitat fragmentation, logging, and deforestation. In *Bats in the Anthropocene: Conservation of bats in a changing world* (pp. 63-103). Springer, Cham.

##### Moreno, C.E, and G. Halffter. 2001. On the measure of sampling effort used in species accumulation curves. *Journal of Applied Ecology*, pp.487-490.

Mahandran, V.,C. M. Murugan and P. T. Nathan, 2015. An unusual roosting behaviour of Schneider’s leaf-nosed bat, *Hipposideros speoris* at a cave-temple roost in Tamil Nadu. *Current Science*,109(5), pp 858:859.

Nikil.K.2020. Assessment of seasonal changes in total open water extent of tanks of Tirunelveli and Thoothukudi districts of Tamil Nadu using geomatics. Masters dissertation thesis, Pondicherry Central University, India. pp 44.

Payton, M. E., M. H. Greenstone, and N. Schenker. 2003. Overlapping confidence intervals or standard error intervals: what do they mean in terms of statistical significance? Journal of Insect Science 3:1–6.

Rainho, A., Palmeirim, J.M., 2011. The importance of distance to resources in the spatial

701 modelling of bat foraging habitat. PLoS One 6, e19227. doi:10.1371/journal.pone.0019227

Rajasegarn, P., N. Shazali, and F.A.A. Khan. 2018. Microclimate and Physiological Effects in the Roosts of Cave Dwelling Bats: Implications in Roost Selection and Conservation in Sarawak, Malaysian Borneo. *Zoological science*, *35*(6), pp.521-527.

Razgour, O., Hanmer, J. & Jones, G. 2011. Using multi-scale modeling to predict habitat suitability for species of conservation concern: the grey log-eared bat. Biological Conservation, 144, 2922–2930.

Samrat.A, M. S. Devy & T. Ganesh. 2021. Delineating fragmented grassland patches in the tropical region using multi-seasonal synthetic aperture radar (SAR) and optical satellite images, International Journal of Remote Sensing, 42:10, 3938-

3954, DOI: [10.1080/01431161.2021.1881181](https://doi.org/10.1080/01431161.2021.1881181), IF:2.976

Selvarathinam, T, and G. Marimuthu. 2013. Roost selection and fidelity of leaf-nosed bat,

*Hipposideros speoris*. International Journal Current Trends in Research, 2: 62-65

##### Sophia,E. 2010.Foraging behaviour of the microchiropteran bat, *Hipposideros ater* on chosen insect pests. Journal of Biopesticides 3(1 Special Issue) 068 – 073.

Sophia,E, and J.Vanitharani.2006. *Hipposideros ater* in the ecosystem of Tirunelveli, Tamilnadu. Journal of Theoretical and Experimental Biology. Vol. 3 Pp: 27-34 ISSN: 0972-9720.

Stebbings, R.E. 1995. Why should bats be protected? A challenge for conservation. *Biological Journal of the Linnean Society*, *56*(suppl_1), pp.103-118.

Suksai, P., and S. Bumrungsri. 2019. Water bodies are a critical foraging habitat for insectivorous bats in tropical agricultural landscapes of central Thailand. *Songklanakarin Journal of Science and Technology*.

Swamidoss, P.D., M.R. Sudhakaran, and P. Parvathiraj. 2012. Habitat preference of microchiropteran bats in three districts of Tamilnadu, South India. *International Research Journal of Biological Sciences*, *1*(5), pp.24-30.

##### Thet.T and K. M. Mya 2015. Harvesting the guano of insectivorous bats: is it sustainable? Journal of Threatened Taxa 7(6): 7296–7297.

Treitler, J.T., Heim, O., Tschapka, M. and Jung, K. (2016), The effect of local land use and loss of forests on bats and nocturnal insects. Ecol Evol, 6: 4289-

4297. <https://doi.org/10.1002/ece3.2160>).

Vanitharani, J., 2004. The Emerging Trends in the Bio-Diversity of Bats in Tamil Nadu. *Mapana Journal of Sciences*, *2*(2), pp.14-27.

Voigt, C.C., and T. Kingston. 2016. *Bats in the Anthropocene: conservation of bats in a changing world* (p. 606). Springer Nature.

##### Vonhof, M.R., and Barclay. 1996. Roost-site selection and roosting ecology of forest-dwelling bats in southern British Columbia. Canadian Journal of Zoology, 74: 1797-1805.

Williams-Guillen, K., E. Olimpi, B. Mass, P.J. Taylor, and R. Arlettaz. 2016. Bats in the anthropogenic matrix: challenges and opportunities for the conservation of Chiroptera and their ecosystem services in agricultural landscapes. In *Bats in the Anthropocene: Conservation of bats in a changing world* (pp. 151-186). Springer, Cham.

##### Wanger, T.C., K. Darras, S. Bumrungsri, T. Tscharntke & A.M. Klein (2014). Bat pest control contributes to food security in Thailand.Biological Conservation 171: 220–223; <http://dx.doi.org/10.1016/j.biocon.2014.01.030>

Figure 1	[Click here to access/download;Figure;Figure 1.jpg](https://www.editorialmanager.com/pone/download.aspx?id=30281356&amp;guid=87bd2f39-33f6-4768-92ec-b8c782386d67&amp;scheme=1)

Figure 2	[Click here to access/download;Figure;Figure 2.tif](https://www.editorialmanager.com/pone/download.aspx?id=30281359&amp;guid=3fd5f319-9e35-4e83-a4bd-bcd500073d8f&amp;scheme=1)

Figure 3	[Click here to access/download;Figure;Figure 3.tif](https://www.editorialmanager.com/pone/download.aspx?id=30281360&amp;guid=ca16cd42-6a25-4bb9-8fd2-0198f236e027&amp;scheme=1)

Figure 4	[Click here to access/download;Figure;Figure 4.jpeg](https://www.editorialmanager.com/pone/download.aspx?id=30281361&amp;guid=bb93f188-d4e4-4b89-bae1-4691400016e2&amp;scheme=1)

Figure 5	[Click here to access/download;Figure;Figure 5.jpg](https://www.editorialmanager.com/pone/download.aspx?id=30281362&amp;guid=5edcb93d-b071-458e-86c3-1702c801a4c1&amp;scheme=1)

Figure 6	[Click here to access/download;Figure;Figure 6.tif](https://www.editorialmanager.com/pone/download.aspx?id=30281363&amp;guid=a5f474b0-d472-4033-88b7-b3690ef826f2&amp;scheme=1)
