# Supplementary material for: Temples and bats in a homogeneous agriculture landscape: Importance of microhabitat availability, disturbance and land use for bat conservation
Source: PLoS One. 2022 Jul 13;17(7):e0251771. doi: 10.1371/journal.pone.0251771 (PMC9278754; doi:10.1371/journal.pone.0251771)
Supplement: Attachment — Submitted filename: author-responses.docx [file pone.0251771.s009.docx]

6. Review Comments to the Author

Please use the space provided to explain your answers to the questions above. You may also include additional comments for the author, including concerns about dual publication, research ethics, or publication ethics. (Please upload your review as an attachment if it exceeds 20,000 characters)

Reviewer #1: It is obvious that the authors have spent a lot of time and effort in revising this manuscript. The details on the analyses have been provided and the methods are sound. The information presented is very interesting. There is not a data availability statement in the manuscript, but the authors do indicate in the submission form that they are available upon request. There are still many grammatical errors, but I have provided specific suggestions for the authors to improve the grammar and readability. My suggestions are included in the attached PDF and the authors can use Adobe Acrobat Reader DC to view the edits.

**Authors response (AR)**

**Thank you for the reviews and edits on the manuscript. We have incorporated your edits and have gone through the MS thoroughly to correct for grammatical errors.**

Reviewer #2: Review: Temples the last abode for bats in a homogeneous agriculture landscape: Importance of microhabitat availability, disturbance and land use factors for bat conservation.

The edits to this article made the methods much more robust and clarified many of my previous concerns, especially the updated methods for landcover classification. There remain some spacing and grammatical issues some of which I lined out below. With minor grammatical and editorial revisions, this paper will meet publication criteria.

Revisions
Ensure there is a space between every genus and species- M. lyra, not M.lyra
Ensure there is a space between each paratheses and last word in each instance- i.e. temples (Audet et al 1991) or trees (3%)

**AR:We have corrected for space**

Figure 6’s font is quite hard to read. I think the resolution of the image needs to be increased.
**AR:We have changed the resolution. The actual image is clearer than the embedded one.**

Double check the style of your citations. The format differs between citations with respect to spacing and comma use between the last name and year.
In the last paragraph, two species mentioned need to have the Genus capitalized and the species italicized.
**AR:We have used the Plos One reference format**

The last sentence in the Bats and Roost Characteristics paragraph is incomplete or contains an extra word.
The list of years- 2012,2013,2014,2018,2019. – should be 2012, 2013, 2014, 2018, 2019 with spaces in between and no period at the end. Similar spacing should be maintained in the list of distances surveyed in the Data Analysis paragraph.

**AR: Rectified**

I would recommend rephrasing your title and your introductory sentence of the Conservation of bats in temples paragraph. You’ve shown that many bats consistently utilize temples as roosts and that they have preferred microhabitats, but I don’t think you can infer that temples are essential for the persistence of bats. To do this, you would need telemetry data/population-level data for species to show that temples are the majority of roosting locations and reducing temples reduces bat populations because of limited roosting which may not be true for common species. There may be enough other buildings nearby to sustain bat populations without temples.

**AR: We agree. Accordingly have modified the title****“Temples and bats in a homogeneous agriculture landscape: Importance of microhabitat availability, disturbance and land use for bat conservation”.**

Reviewer #3: There are still many grammatical corrections to make for publication (attached Word document with track changes). Also the in-text citations are not consistent with regard to semicolons and commas between citations and periods after et al. There are still species abbreviations missing the space between genus and species as mentioned by reviewer 1 previously.

**AR: We have now addressed these issues. We have removed species abbreviations.**
